# Welcome to 310 Environmental Working Group! A Group Project That Places Students in the Role of Consultants Helping Businesses Choose the Most Climate Friendly Fluorinated Gas

**DOI:** 10.1021/acs.jchemed.4c00479

**Published:** 2024-09-06

**Authors:** Jessica C. D’eon, Sivani Baskaran, Jennifer A. Faust, Mima Staikova, Cora J. Young

**Affiliations:** † Department of Chemistry, University of Toronto, 80 St. George Street, Toronto, Ontario M5S 3H6, Canada; ‡ Department of Environmental Engineering, Norwegian Geotechnical Institute (NGI), P .O. Box 3930, Ullevål Stadion, NO-0806 Oslo, Norway; ¶ Department of Chemistry, College of Wooster, 943 College Mall, Wooster, Ohio 44691, United States; § Department of Chemistry, York University, 4700 Keele Street, Toronto M3J 1P3, Ontario, Canada

**Keywords:** Upper-Division Undergraduate, Environmental Chemistry, Collaborative/Cooperative Learning, Communication/Writing, Problem Solving/Decision Making: Applications of Chemistry, Atmospheric Chemistry, Computational Chemistry, Green Chemistry, IR Spectroscopy, Physical Properties, Rate Law

## Abstract

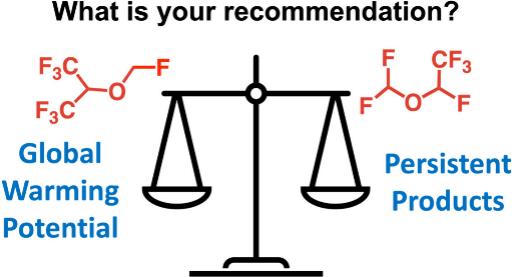

The Montreal Protocol is an international treaty that controls substances that deplete the ozone layer. Through the control of halogenated gases, it has been one of the most successful climate legislations to date. This success is driven by the interplay between chemical regulation and smart chemical design, demonstrating the positive impact chemistry can have on the world. This Article describes a group project that includes four assignments, a group presentation, and a writing task where students take on the role of consultants to assess the environmental friendliness of two fluorinated gases. Through the assignments students determine the global warming potential of two chemicals and pair this assessment with an evaluation of their potential to produce persistent products, such as trifluoroacetic acid, via atmospheric oxidation. Students worked together to take these, sometimes conflicting, pieces of evidence to make a final recommendation to their client as to the most “environmentally friendly” option in a mock Board of Directors meeting and then individually through a written recommendation. The project effectively addressed the learning goals of a third-year environmental chemistry class and was well received by students as a means of contextualizing the course material and providing students with a clear peer network in the class. This project is an effective application of fundamental chemistry topics (e.g., spectroscopy and the relationship between structure and reactivity) within a real-world context that emphasizes the ability of chemistry to have a positive impact on important environmental issues such as climate.

Most university students are interested in sustainability and how it relates to the topics they are learning.^1^ In the chemistry context this has been accomplished by framing content through the UN sustainable development goals^2−4^ or the principles of green chemistry.^5−8^ The project presented here uses the chemistry of fluorinated gases to explore how smart chemical design has produced commercially viable materials with decreased environmental impact. This is a green chemistry success story and an example of chemical regulation outside toxicity.

Chlorofluorocarbons (CFCs) were first-generation refrigerants and aerosol propellants with extremely long atmospheric lifetimes and the ability to deplete stratospheric ozone through the production of chlorine radicals.^9^ The carbon–fluorine bond does not cleave in the stratosphere, and so fluorinated gases do not deplete stratospheric ozone.^10^ As a result, fluorinated gases have been used as CFC replacements since the signing of the Montreal Protocol that controls ozone depleting substances in 1987.^11−13^ However, many fluorinated gases are potent greenhouse gases^14−16^ and so are still controlled by the Montreal Protocol through the Kigali amendment, which expanded the scope of this legislation to include substances with an effect on climate.^11−13^ By controlling the emission of these potent greenhouse gases, the Montreal Protocol has been an incredibly effective international climate policy.^17,18^

In this project students use the climate metrics of radiative efficiency (RE), radiative forcing (RF), and global warming potential (GWP) to assess the climate impact of fluorinated gases.^19^ RE has units of W m^–2^ ppb^–1^ and is a measure of how efficiently a chemical absorbs photons that are important to Earth’s energy balance. Earth emits in the infrared (IR) region with strong emission between 800 and 1250 cm^–1^ (Figure 1), a region referred to as the “atmospheric window”.^19^ The carbon–fluorine bond stretch often falls in the atmospheric window (Figure 1), explaining why many fluorinated gases are potent greenhouse gases.^14−16^ RF is a measure of a chemical’s instantaneous radiative warming; it has units of W m^–2^ and is the product of RE and concentration. GWP considers both RE and atmospheric lifetime through a thought experiment where 1 kg of a chemical is released into the atmosphere and its cumulative effect on RF is compared to the same scenario for CO_2_ over a defined time horizon, typically 100 years:
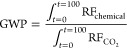
1

**Figure 1:**
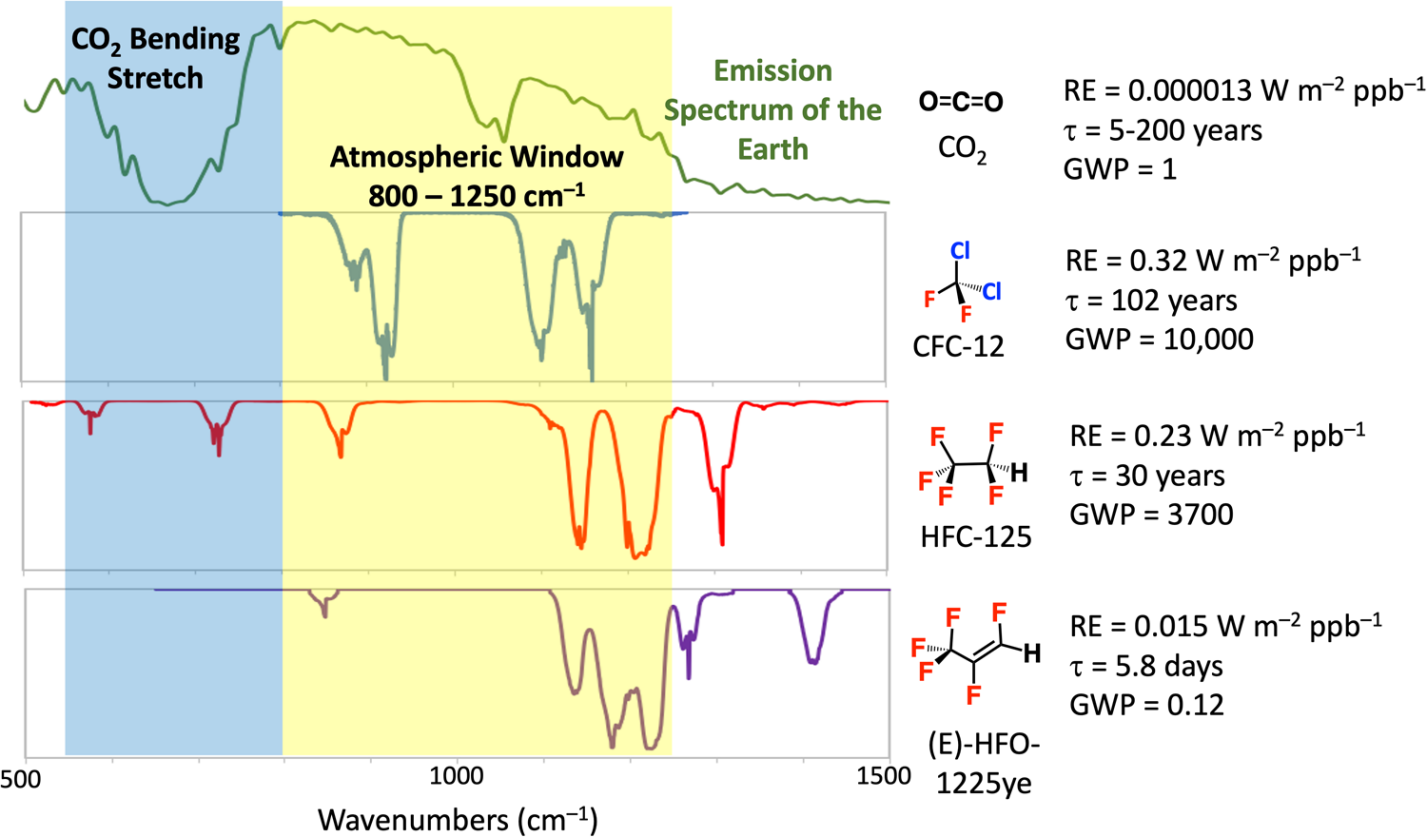
Overlay of the emission spectrum of the Earth (top), where the bending stretch from CO_2_ is identified, with the IR spectra for CFC-12, HFC-125, and (*E*)-HFO-1225ye together with relevant climate metrics of radiative efficiency (RE), atmospheric lifetime (τ), and global warming potential (GWP).^21^

Figure 1 explores the relationship between chemical structure and climate effects by overlaying the IR spectra of three commercial refrigerants with the emission spectrum of the Earth. CFC-12 has a GWP of 11,200.^20^ A first-generation replacement, HFC-125, is a hydrofluorocarbon (HFC) with a GWP of 3700,^20^ and a current use chemical, (*E*)-HFO-1225ye, is a hydrofluoroolefin (HFO) with a GWP of 0.12.^20^ This decrease in GWP from CFC to HFC to HFO is driven by increased reactivity to atmospheric oxidation. This change in structure was purposefully designed to meet both commercial requirements and regulation requirements to decrease the chemical’s impact on climate.

The newest generation of fluorinated gases, the HFOs, have limited climate impacts, but many produce trifluoroacetic acid (TFA) in almost 100% yield.^22−24^ From an industry and regulatory perspective, the production of TFA from the atmospheric oxidation of fluorinated gases with low GWP has generally been considered a reasonable trade-off.^25,26^ However, TFA has recently been found in human blood^27^ and observed to be increasing in environmental compartments including ice cores from the Arctic^28^ and plant leaves.^26^ The ethics of releasing large quantities of TFA, which is a persistent anthropogenic pollutant, are questionable,^29,30^ and proposed policy would limit the production of TFA from fluorinated gases.^31^

The project described in this Article has students looking comprehensively at the environmental fate and implications of different fluorinated gases and making recommendations, sometimes clear-cut and often not, about what trade-offs are reasonable and which gases should be considered more “environmentally friendly”. To accomplish this task students need to assume a system-wide perspective and make predictions using their fundamental chemistry knowledge (e.g., mechanistic understanding of organic reactions and spectroscopy), which is inherently a systems-thinking approach.^32−34^ Through the project students appreciate how chemistry knowledge can be used to evaluate a chemical’s impact on climate and how the interplay of legislation and commercial chemical production can result in increasingly benign commercial materials.

## Project Framing and Logistics

This project was undertaken in a third-year environmental chemistry class, CHM310 *Environmental Chemistry*, which is part of our Environmental Chemistry Major and Green Chemistry Focus^6^ but is also available to any student who meets the second-year organic chemistry prerequisite. CHM310 has two lecture hours a week. The project spanned the entirety of our 12-week semester and included four individual assignments, a group presentation, and an individual writing assignment. It was included in the winter 2016 (93 students enrolled), winter 2017 (71 students enrolled), winter 2018 (65 students enrolled), and fall 2018 (49 students enrolled) iterations of the class. For larger curricular reasons, CHM310 was rebranded as *Environmental Fate and Toxicology of Organic Contaminants* in 2020 and climate was moved to a second-year environmental chemistry course, and so the topic of the project was no longer appropriate.

**Figure 2:**
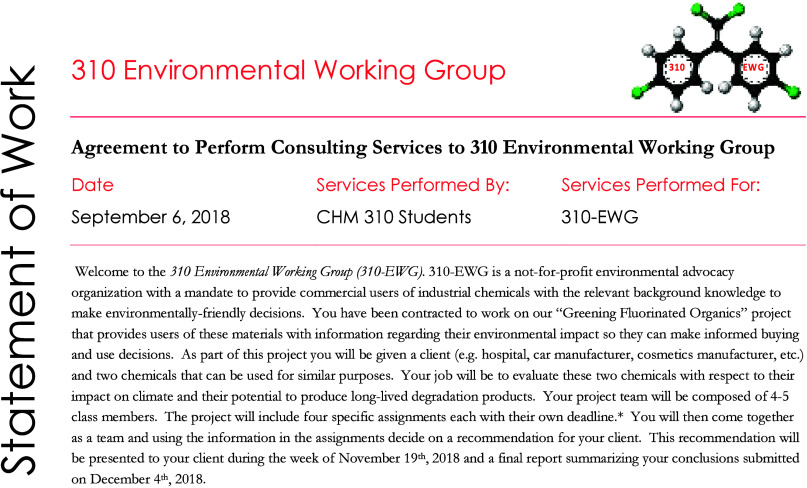
Fall 2018 Statement of Work that was provided to students at the outset of the class. All documents from the fall 2018 iteration of the project are provided in the Supporting Information.

For this project students assumed the role of scientific consultants working in groups of 3–5 for 310 Environmental Working Group (310-EWG), the consulting company for the course. Students were introduced to the project using a Statement of Work, which is shown in Figure 2, and all documents related to the project are available in the Supporting Information (SI). Each group was assigned a specific client and task that involved an evaluation of the environmental fate and climate implications for two fluorinated gases with relevant commercial applications. The three scenarios and chemicals involved in the fall 2018 iteration of the class are shown in Table 1. The four assignments served to build the scientific knowledge basis for the recommendation that the students would make, which was delivered as a group presentation at a mock Board of Directors meeting. Students also wrote individual written recommendations that included both a technical component and a lay summary.

**Table 1:** List of Clients, Applications, and Chemicals^a^

Commercial Application and Client Type		Chemical Formula, CAS Number, and Common Name	Origin of the Data	τ (years)	RE (W m^–2^ ppb^–1^)	GWP	Atmospheric Oxidation Products
Inhaled anesthetic (hospital)	A1	(CF_3_)_2_CHOCH_2_F	Literature^20^	1.9	0.31	195	TFA, CO_2_, F^–^
		28523-86-6	Class	0.78	0.46–0.49	63–220	
		Sevoflurane					
	A2	CHF_2_OCHFCF_3_	Literature^20^	14	0.46	2600	CO_2_, F^–^
		57041-67-5	Class	3.2–4.1	0.45–0.50	500–890	
		Desflurane					
Refrigerant (automotive company)	R1	CF_3_CH_2_OCHFCF_3_	Literature				TFA, CO_2_, F^–^
		55605-86-2	Class	0.90	0.50–1.5	240–500	

	R2	CHF_2_CF_2_OCH_2_CF_3_	Literature^20^	6.1	0.48	980	CO_2_, F^–^
		406-78-0	Class	2.7	0.5–0.55	510–740	
		HFE-347pcf2					
Propellant (cosmetic company)	P1	(CF_3_)_2_CFOCH_3_	Literature^20^	3.7	0.32	390	TFA, CO_2_, F^–^
		22052-84-2	Class	0.53–1.4	0.47–0.49	180–300	
		HFE-347mmy1					
	P2	CF_3_CHFCF_2_OCH_3_	Literature^35^	2.6		99	TFA, CO_2_, F^–^
		382-34-3	Class	1.3	0.33–0.43	220–250	
		HFE-356mec					

a Climate metrics of atmospheric lifetime (τ), radiative efficiency (RE), and global warming potential (GWP) are provided from the literature, when available, together with the range of values calculated by the fall 2018 CHM310 students. The range of final atmospheric oxidation products expected for each chemical is also provided.

The mock Board of Directors meetings took place outside class time over four 2-h sessions in the 10th week of the semester with three groups presenting in each time slot. Students self-selected into groups with the presentation time slots assigned to ensure students were available. Once established, each group was sent a contract that included their client and the chemicals they would compare.^18^Table 1 provides the list of the chemicals used in the fall 2018 iteration of the class. A longer list of potential fluorinated gases is provided in the SI, as well as details on how iterations of the project differed.

## Learning Goals

As a class, CHM310 had two overarching multifaceted goals articulated in the syllabus:

1. As a CHM310 student you will be able to predict *where* in the environment (air, water, soil, biota, ...) an organic chemical would be expected to be found as well as *how* it might undergo degradation using only its chemical structure.
2. Climate change is a major topic in CHM 310; upon completion of this class you will be able to assess the potency of a greenhouse gas, understand the origin of climate data, and feel comfortable discussing the science of climate change in lay terms.

The project described here touches all aspects of the course and accounts for 45% of the students’ final grade (breakdown available in the SI), and so it was designed to support all aspects of the course goals. With this in mind, upon completion of the project students will have experience in the following:

1. Comparing chemical structure to expected rates of atmospheric oxidation.
2. Predicting products of atmospheric oxidation.
3. Relating IR spectra to radiative efficiency.
4. Calculating global warming potential.
5. Predicting partitioning of chemicals in the environment.
6. Programming and running a chemical fate model.
7. Producing and delivering a group presentation to a room of peers.
8. Producing both technical and nontechnical writing.
9. Working with peers to evaluate information and come to a consensus.

Learning goals 1–6 relate to the knowledge goals of the class and were evaluated using the questions in each assignment. Learning goals 7 and 8 relate to the final presentation and writing assignment and were evaluated through those assessments. Learning goal 9 relates specifically to the group aspect of the project. All assignments, aside from the final presentation, were completed independently; however, working together as a group was encouraged through language in the framing document and in comments made by the instructor in class. Success of the collaborative learning aspect of this project was assessed using student responses to an anonymous student experience survey and feedback on the end of year course evaluations.

## Assignment Details

The project includes four individual assignments used to create the scientific basis on which students will make their recommendation. The assignments are scaffolded with the content of the class and a logical progression of the analysis. Assignment structure also took into consideration details required for subsequent calculations to ensure students received feedback on each piece before putting them together.

### Assignment 1: Atmospheric Lifetime and Fate

The hydroxyl radical (•OH) is the major oxidant in the atmosphere, and it controls the lifetime of most organic molecules.^36^ •OH reacts via two major pathways, abstraction of a hydrogen atom from an alkane or addition to a double bond.^36^ To simplify the lifetime prediction, the project only included fully saturated hydrocarbons. Students used the Kwok and Atkinson^37^ structure activity relationship (SAR) to predict the rate constant for reaction with •OH (*k*_OH_). At least one of the assigned chemicals had more than one site of potential H-abstraction, and students needed to calculate a *k*_OH_ for each site and add them together to obtain an overall rate constant (*k*_OH TOTAL_).

The reaction kinetics of organic molecules with •OH can be considered pseudo-first-order as •OH is generated photochemically with typical daytime steady-state concentrations of 1 × 10^6^ molecules cm^–3^.^36^ With *k*_OH TOTAL_ and [•OH], students can calculate an atmospheric lifetime (τ_OH_), which is the e-folding time:
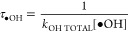
2

Chemicals in the atmosphere can be lost via wet and dry deposition and diffusion into the stratosphere. Given their high vapor pressures, deposition is of limited significance for these fluorinated gases. Loss via diffusion into the stratosphere has a lifetime of about 100 years (τ_STRAT_),^19,36^ and students determined a total lifetime of their chemicals considering both loss mechanisms using the following relationship:
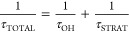
3

**Figure 3:**
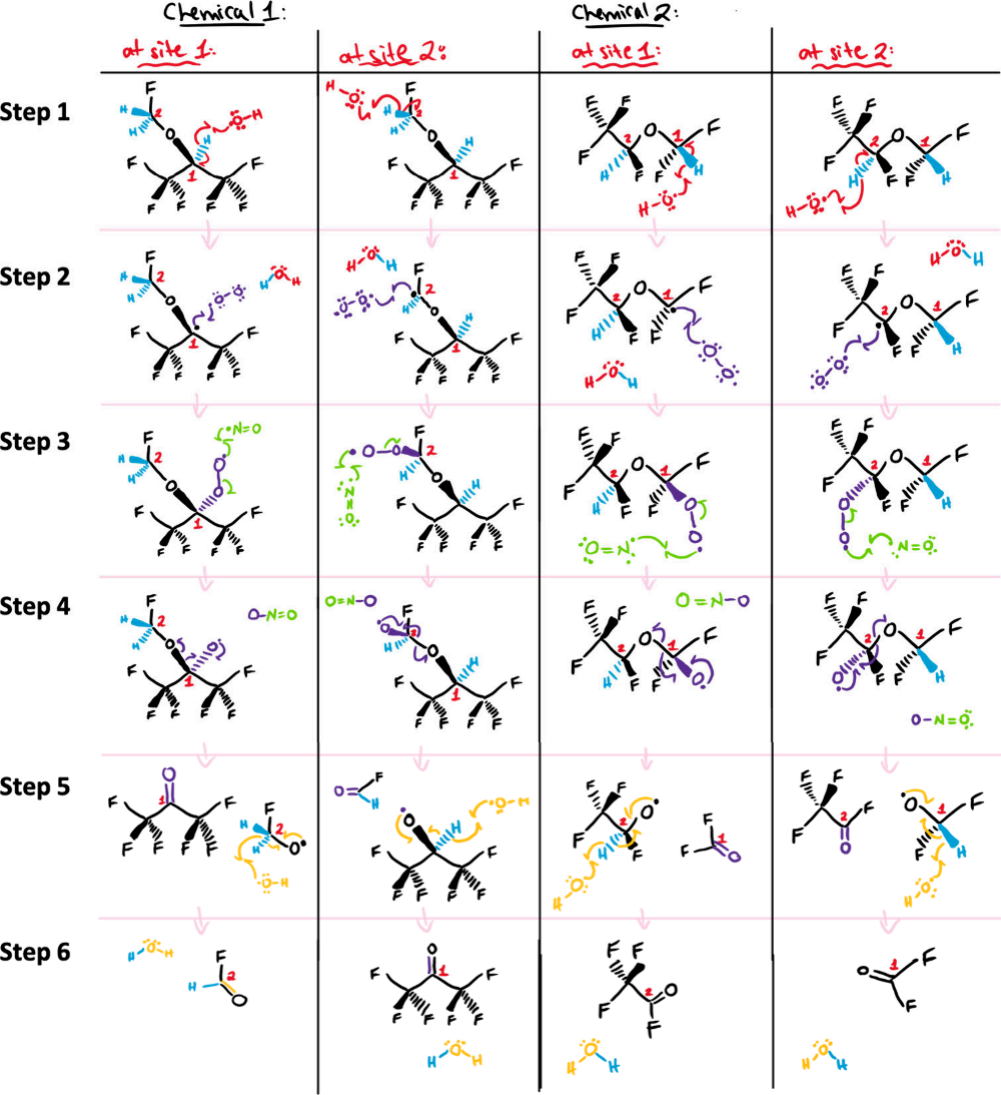
Student figure submitted for assignment 1 detailing the atmospheric oxidation of the inhaled anesthetics Sevoflurane (Chemical 1) and Desflurane (Chemical 2) in fall 2018. The figure was annotated here to indicate steps 1–6 to facilitate discussion in the text.

Students also determined the first stable nonradical product from reaction at each site and submitted a scheme outlining the steps in the mechanism. A sample scheme submitted by a student assigned the two inhaled anesthetics, Sevoflurane and Desflurane, is shown in Figure 3. Atmospheric oxidation mechanisms follow a general four-step pattern:^36^

1. H-abstraction by •OH produces a carbon-centered radical (R•).
2. O_2_ adds to this radical generating a peroxy radical (ROO•).
3. NO abstracts an O generating NO_2_ and an alkoxy radical (RO•).
4. The alkoxy radical (RO•) decomposes to generate a carbonyl; in doing so it must cleave a bond. This is a branching point where there are two possible pathways:
  a. Cleavage of one of the bonds attached to the carbon of the alkoxy radical, generating the carbonyl and a radical on the group that is lost.
  b. If the carbon of the alkoxy radical is bonded to a hydrogen, then O_2_ can abstract the H atom generating HO_2_ and the carbonyl.

The rate of mechanism 4a depends on the nature of the group that is lost. Production of a methyl radical occurs at a rate of about 2 × 10^2^ s^–1^; this increases to 5 × 10^4^ s^–1^ with the production of a primary carbon radical and to 4 × 10^5^ s^–1^ for a secondary carbon radical and 3 × 10^6^ s^–1^ for a tertiary carbon radical.^36^ Rates increase to 10^7^–10^8^ s^–1^ when the alkoxy radical is singly or doubly bonded to an oxygen.^36^ The rate of mechanism 4b is 5 × 10^4^ s^–1^,^36^ and so mechanism 4b is only favored when the radical generated in mechanism 4a is a methyl radical and the mechanisms are in close competition when a primary carbon radical is formed. These rates can be explained using trends in the bond dissociation energies (BDE) predicted when considering the expected stability of the radical that is lost, which is commensurate with carbocation stability with which students are more familiar.^36^

When students draw mechanism 4a, they need to apply a mechanistic understanding of organic chemistry which is enunciated in learning objective 2 of this project, predicting products of atmospheric oxidation. In Figure 3, all the alkoxy radicals in step 4 were adjacent to the ether oxygen, and the student chose to cleave the C–O bond over either a C–C bond or a C–F bond. This scenario of C–O bond cleavage in an ether was not part of our classroom discussion, and so this was a choice by the student. It is possible other mechanisms involving C–C bond cleavage^38,39^ or H-abstraction by O_2_ would compete with the mechanism as drawn, but this mechanism is reasonable and was given full credit.^36^

The reaction scheme generated in this assignment was used to evaluate student success with learning objective 2, predicting products of atmospheric oxidation. The calculation of *k*_OH_(37) was the first step in evaluating learning objective 1, comparing chemical structure to expected rates of atmospheric oxidation. However, students could complete the calculation without an appreciation for the underlying chemistry. To flesh out the students’ ability to explain and apply the relationship between chemical structure and rates of atmospheric oxidation, the assignment included the two qualitative questions shown in Table 2. Q4 asked students to evaluate and comment on the product distribution proposed by the SAR, and Q9 asked students to describe the effects of ether functionality on reaction rate.

**Table 2:** Two Student Responses to Assignment 1 Qualitative Questions That Probe Student Understanding of Chemical Reactivity with Respect to Reaction with OH

Question	Student Responses	
**Q4:** Choose one of your molecules that contains more than one reactive site and with reference to the chemical structure explain why one site is more reactive than another or why they are similarly reactive.	**Student 1**	
	Chemical:	(CF_3_)_2_^**1**^**CH**O^**2**^**CH**_**2**_**F**
	Product distribution:	C1–4%
		C2–96%
	Explanation:	Carbon #2 is more reactive than carbon #1 because it is more electron rich. Carbon #2 only has a fluorine and an oxygen pulling electrons away meanwhile carbon #1 has two CF_3_ and an oxygen which, together, are more electron withdrawing and hence, make carbon #1 more electron poor. The carbon that is more electron rich will form the more stable carbon radical, which is why carbon #2 is much more thermodynamically favorable.
	**Student 2**	
	Chemical:	CF_3_^**1**^**CHF**CF_2_O^**2**^**CH**_**3**_
	Product Distribution:	C1–2%
		C2–98%
	Explanation:	Although CF_3_^1^CHFCF_2_O^2^CH_3_ has two sites that can undergo H-abstraction by hydroxyl radical, the second position located at the primary carbon is more reactive and favored. The presence of the ether functional group (−O−) as the substituent bonding off the primary carbon is able to aid in stabilizing the loss of the hydrogen atom through resonance. The lone pairs of electrons on the oxygen atom are able to move about regions of the chemical adding stability by forming double bonds, in doing so the second position is to be more reactive as the product is more stable.
**Q9:** Both of your chemicals contain an ether functional group. How does this functionality affect the atmospheric lifetime of these compounds? Explain your answer.	**Student 1**	
	Ether functional groups help decrease the atmospheric lifetime of these compounds. Ethers contain oxygen that has to ability to back-donate electrons from their pi orbitals creating more stable radicals through resonance. This increase in stability creates a more stable product, favoring the reaction leading to an increase in the reaction rate which leads to a decrease in their atmospheric lifetime.	
	**Student 2**	
	The functional group of ethers has two lone pairs of electrons, which are able to add stability to chemicals by providing resonance. The ability to move electrons within a chemical can reduce the effect of a stress that has been applied to said chemical. A carbon radical for instance is highly reactive due to its single electron and the nature of radicals. However, in the presence of an ether substituent the lone pairs are able to interact with the radical electron and produce double bonds, delocalizing the position of the lone electron.	

Both responses to Q4 shown in Table 2 received full credit as they correctly identified factors involved in the predicted product distribution. Student 1 identified the electron withdrawing effects of the F atoms, the CF_3_ group, and the adjacent ether but did not comment on the ability of the oxygen to stabilize a carbon-centered radical through resonance, likely because both reactive sites were adjacent to the ether. Conversely, Student 2 focused on the ability of the ether oxygen to stabilize the resulting radical through resonance because only the C2 site was adjacent to the ether. The role of resonance stabilization of radical intermediates was often more difficult for students to identify as compared to inductive effects, and so Q9 was designed to probe the application of this topic. Both students provided clear and correct explanations in Q9, demonstrating their knowledge and the ability of this question to probe this specific topic.

### Assignment 2: Radiative Efficiency and Global Warming Potential

In assignment 2 students calculated RE using an IR spectrum. The vibrational frequencies and the resulting IR spectrum were derived from quantum mechanical (QM) computations using the Gaussian 09^40^ computational package accessed through the web-based interface WebMO^41^ (details in the SI). A model of RE per wavenumber developed by Pinnock et al.^42^ was used to translate the observed frequencies and intensities from the IR spectrum into an RE for each chemical. With RE from this assignment and the atmospheric lifetime from assignment 1, students were ready to calculate GWP. This was accomplished by calculating the atmospheric concentration of each chemical after a 1 kg bolus addition over 100 years with 1 year resolution. Multiplying by RE gives RF at each time point, which can be summed to give an approximation of the total forcing from this bolus addition over 100 years. Students then calculated the same scenario for CO_2_ using an RE value of 1.4 × 10^–5^ W m^–2^ ppb^–1^ and this relationship that models the loss kinetics of CO_2_ from the atmosphere:^43^
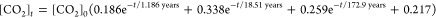
4

The ratio of total RF for the chemical in question relative to the same scenario for CO_2_ is the GWP.

Literature GWPs for each chemical and the range of values calculated by the fall 2018 CHM310 students are provided in Table 1. Exact agreement with literature is not expected given the use of calculated IR spectra and predicted •OH kinetics. These values could be tightened up using experimental values; however, there is learning value in these predictive tools. The substituent factors in the Kwok and Atkinson^37^ SAR provide insight into groups that tend to be activating or deactivating, and calculating an IR spectrum is a useful application of computational chemistry and allows students to visualize the movement of the chemical in relevant vibrational modes.

Assignment 2 addressed both learning goal 3, relating IR spectra to radiative efficiency, and learning goal 4, calculating global warming potential, as both calculations were done from first principles. Students’ ability to explain these relationships was assessed using a qualitative question that asked whether overtone frequencies, which typically fall in the range of 3000–3500 cm^–1^, are expected to be important to a chemical’s RE. The following student response demonstrated their understanding of the importance of photon frequency in relation to Earth’s emission spectrum to a chemical’s RE:

“No, they would not. The overtones are at a frequency where very little light of that frequency is emitted by Earth. Therefore, they would have very low radiative efficiencies per unit cross sectional area, leading to low radiative efficiencies.”

### Assignment 3: Environmental Fate

**Figure 4:**
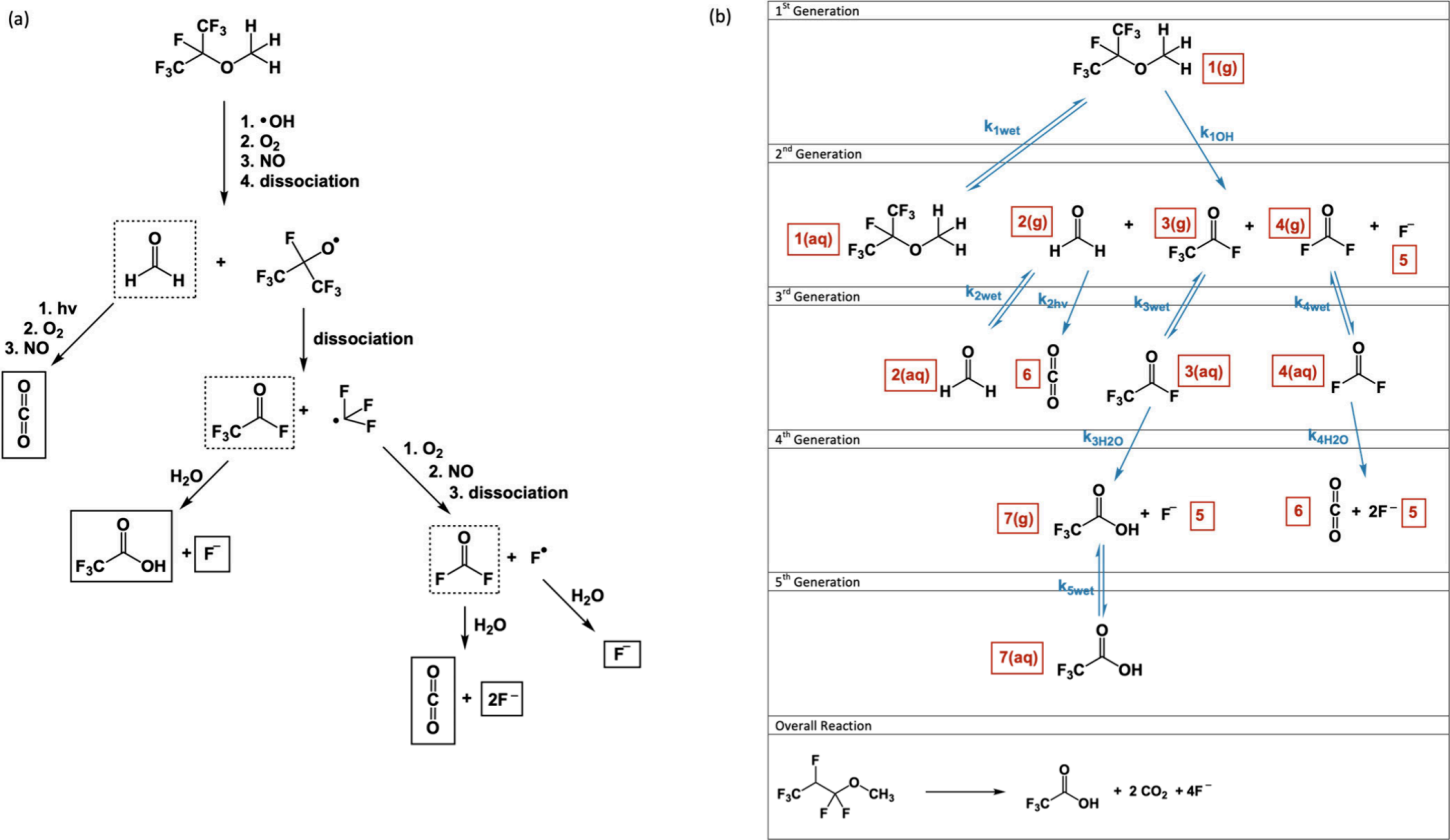
Student-generated (a) degradation scheme and (b) generational plot for the propellant P1 (HFE-347mmy1). All structures were created using ChemDraw^46^ to which the University of Toronto has a site license. ChemSketch^47^ is a similar software that is free for academic use.

Assignment 3 expanded the mechanistic work of the first assignment. Students took their schemes from assignment 1 and continued the degradation to final terminal products. At this point in the semester, the class had covered photolysis of carbonyls in the atmosphere and hydrolysis reactions of esters and acid halides in aqueous solution. With this background, students were prepared to describe the fate of the initial carbonyl products of atmospheric degradation. A typical complete mechanism is shown in Figure 4a with final degradation products of CO_2_, F^–^, and TFA. Of these products, CO_2_ and F^–^ are desirable as they are the inorganic versions of these atoms and so the organofluoride molecule has mineralized. TFA is a worrisome product as the carbon–fluorine bond is incredibly strong, making fluorinated acids generally resistant to environmental degradation, so much so they’ve been labeled “forever chemicals”.^44,45^

Building on the reaction scheme, students produced a figure we called a generational plot which is shown in Figure 4b. The purpose of this plot was to help students write the mass balance equations they would need to program the chemical fate model in the last assignment. Each arrow in the generational plot included a rate constant (*k*) for the process in question. The model included both a water and an air compartment, and so students needed to include partitioning between these phases in the generational plot and consider the phase of the chemicals when outlining proposed reactions. Each chemical in the generational plot was given a number and a phase. For example, in Figure 4b the parent chemical, (CF_3_)_2_CFOCH_3_, was given number 1(g) in the first generation where it is emitted and 1(aq) in the second generation to account for potential dissolution into water. The rate of this process is defined by *k1*_wet_ which refers to the transfer of the chemical from the gas phase into the aqueous phase as defined by its air–water partition coefficient (*K*_AW_). Certain products, like CO_2_ and F^–^, are generated in multiple different ways and so exist as products in more than one generation but are all given the same number. This assignment served to solidify learning goal 2, predicting products of atmospheric oxidation, and learning goal 5, predicting partitioning of chemicals in the environment.

### Assignment 4: Chemical Fate Model

**Figure 5:**
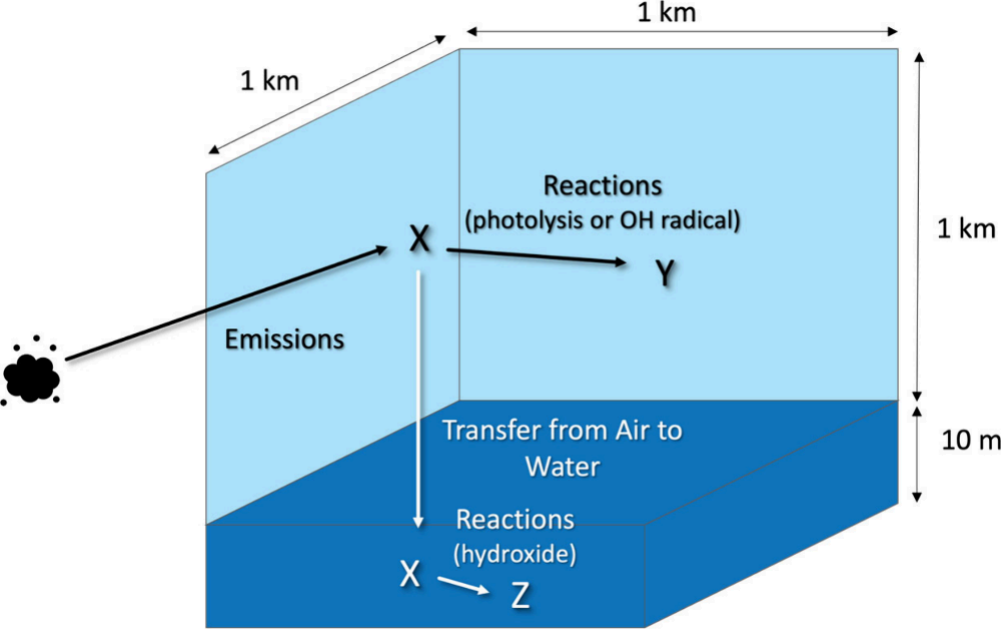
Pictorial representation of the chemical fate model used in Assignment 4.

A representation of the chemical fate model is shown in Figure 5, which includes the sizes of the air and water compartments and arrows showing the processes. The model, based on work by Mackay et al.,^48^ was first written and run in Microsoft Excel Visual Basic for Applications (VBA) and has since been updated to run using R and R Studio;^49^ both versions are available in the SI together with detailed instructions. Within the provided code students had to write mass balance equations. Once fully programmed, the model would calculate the change in concentration for each chemical in each compartment every minute over 100 years. This time resolution was necessary given the speed of many of the processes involved; however, this can be computationally intensive. The Excel VBA version took much longer to run than the R version, and students may obtain errors due to limitations in available RAM during the calculations. One simplification that was included to decrease computing power and to simplify the mass balance equations was to make the processes involved unidirectional and irreversible. This is appropriate for chemical reactions; however, it is an oversimplification for the partitioning between phases as it means chemicals that move into the aqueous phase are not able to move back into the air.

Students ran the model for each of their assigned chemicals for 100 years, with 1 kg emission per year into the air for the first 50 years followed by no emission for the final 50 years, to model an extended emission period followed by regulation and recovery. An example student output from the Excel VBA model is available in the SI. This assignment visualized the issue of persistent products, as once TFA was produced in the modeled environment it remained present even 50 years after production of the commerical product had ceased. The assignment addressed learning goal 6, programming and running a chemical fate model, as students were required to write their own mass balance equations and add them to the model before running. Several office hours were offered in a computer lab to ensure students were supported in the programming and running of the model. After this assignment was complete, students had all the data they needed to generate a final recommendation which they would deliver as a group presentation at a mock Board of Directors meeting.

## The Role of Peer-to-Peer Learning

One issue with using assignments that build on each other is the need to ensure students have the correct answers from one assignment before moving onto the next. Solutions outlining an appropriate approach to each question were posted, and students were encouraged to come discuss their results with the instructor during office hours or, better yet, to compare their results with their group members and work together to generate what they considered to be the most appropriate result. Results from a winter 2017 anonymous student experience survey confirm that students were working together on the individual assignments and that this collaboration increased as the semester proceeded. Only 36% of students reported spending time working with their group members on assignment 1, but this increased to 39% for assignment 2, 56% for assignment 3, and 66% for assignment 4. Student opinion of the group work component was overall positive with 71% of winter 2018 students either agreeing or strongly agreeing with the statement *“The group work component of the industrial consultant project improved my learning experience in CHM 310 overall.”* The idea of students working together toward consensus was solidified when preparing the final group presentation as, whether students had been working together throughout the semester or not, they were required to come together and produce one presentation with one set of information. In almost all cases this approach generated reasonable results. When the winter 2017 students were asked whether all group members participated in choosing the consensus values, 93% said yes. Although the group work was overall successful in these projects, the students would have benefitted from additional resources or reflection pieces to support their ability to work together.

In a university as large as the University of Toronto, any way of providing students with a clear peer group within the class was useful for students looking for that support. This was demonstrated in the winter 2017 survey where 75% of students either agreed or strongly agreed with the statement *“I feel as though meeting other students through the group work component of the ICP [Industrial Consultants Project] assignments helped my learning in CHM310 as a whole”* and 34% of students reported that they worked with their group members on course components unrelated to the project. The importance of peer-to-peer learning, as facilitated by this project, was also expressed in comments on the end of term course of evaluations:

The group project was also fun and helped support my learning because my group members and I taught each other concepts we did not understand. [Winter 2018]

## Board of Directors Meeting

There were three groups of students in each Board of Directors meeting. The group that was presenting played the role of the consultants speaking to the Board, and the other two groups and the instructor played the role of the Board. Students were given one bonus mark on their individual presentation grade if they asked a question of another group as a member of the Board, resulting in some lively discussions. These group presentations were a fantastic way to end the semester, as they provided an additional modality of assessment, solidified the peer learning aspect of the project, and often felt celebratory. They were also useful as a touchpoint to ensure all students had appropriate plots and values to use in their written reports as any misunderstandings that were not ironed out in the planning process were resolved through student or instructor questions.

Presentation grades were a combination of a group grade out of 20 and a personal grade out of 10, both determined by the instructor and each worth 5% of the students’ final grade. Students were also responsible for completing a peer evaluation of one other group in their timeslot, and the average of these peer evaluations was worth 3% of the students’ final grade. A rubric for these evaluations is provided the SI.

## Written Recommendation

In the final assignment, students were tasked with writing both a lay summary (300 words max) and a technical summary (700 words max). As this was the only writing assessment in the course, there was limited opportunity to iterate and help students improve their writing. However, it was useful to emphasize to students the importance of written communication skills and to provide them with a variety of assessment modalities. The technical summary was written to someone with the same background as the CHM310 students. The lay summary asked students to explain their recommendation without technical language. Here is an excerpt of the lay summary of a student explaining the difference in the production of persistent products between the two inhaled anesthetics Sevoflurane and Desflurane.

Not only will they both warm the planet but each of them will be partially transformed into a harmful chemical. These harmful chemicals will persist in the environment for very long periods of time. Sevoflurane will transform itself into a chemical called formic acid meanwhile Desfluorane will transform itself into the chemical abbreviated as TFA. While both are problematic for earth’s ecosystem, TFA’s effects are more harmful than formic acid’s. In addition, formic acid is already found naturally in small amounts meanwhile TFA is not. As such, formic acid can be eliminated by nature meanwhile it is unknown if such a process exists for TFA. However, their harmful effects in nature highly depend on their amounts that are emitted; where their effects worsen in increasing amounts. The amounts of TFA produced from Desfluorane are significant meanwhile the amounts of formic acid produced from Sevoflurane are negligible.

This student recommended the use of Sevoflurane to the hospital partner in large part because of the difference in its potential to generate the persistent product TFA. They were able to explain issues surrounding environmental contamination clearly and accurately without assumed background knowledge. The student used the technical work done in the assignments to justify the recommendation, and when some technical language was required, such as formic acid and TFA, it was defined and contextualized.

## Conclusions

This Article outlines a group project that had students using structure–activity relationships, computational chemistry methods, and a chemical fate model to investigate the climate implications and potential for environmental contamination from the use of fluorinated gases. Students self-selected into groups of 3–5 and were placed in the role of scientific consultants to a variety of businesses where they used information generated to make a recommendation to their client. This project had students explore what it means to create a commercial product that is climate and environment friendly. It also provided an important narrative structure and sense of continuity to the environmental chemistry course in which it was implemented, with 90% of the winter 2017 students either agreeing or strongly agreeing with the statement *“I feel as though the ICP [Industrial Consultants Project] assignments created a sense of continuity and purpose in CHM310”*. The group aspect of the project provided students with a peer network, which was important for the learning of some students. Although presented here as a unit, individual assignments could be tailored to a variety of chemistry courses and used as a mechanism to engage students in climate-relevant activities as an application of fundamental chemistry principles. Some ideas on how to accomplish this are provided in the SI. The context of fluorinated gases, as controlled through the Montreal Protocol, demonstrates the potential of chemical regulation paired with green chemistry and sustainable chemistry to generate useful and benign chemicals.

## Supporting Information Available

All details for the fall 2018 project, which include the statement of work, example contracts sent to student groups, assignments 1–4 including the student versions, answer keys, and report sheets completed by the students, instructions for the Gaussian 09 calculations, brief presentation of the QM concepts used in the IR spectra calculations, instructions for the chemical fate model and Excel VBA and R versions of the model, guidelines and rubrics for the presentation and report, and list of potential chemicals to include in the project (ZIP)
Discussion of the results of the chemical fate model, notes on the different iterations of the projects, ideas on how to use the material in a modular way, and the surveys implemented in winter 2017 and winter 2018 (PDF, DOCX)

## Notes

Due to updates to various R packages, the version of the R code included here uses functions that have since been deprecated or superseded (e.g., gather from tidyverse) and may produce warnings when run.
