# Supplementary material for: Welcome to 310 Environmental Working Group! A Group Project That Places Students in the Role of Consultants Helping Businesses Choose the Most Climate Friendly Fluorinated Gas
Source: J Chem Educ. 2024 Sep 6;101(10):4203–13. doi: 10.1021/acs.jchemed.4c00479 (PMC11465463; doi:10.1021/acs.jchemed.4c00479)
Supplement: Supplementary file 1 — ed4c00479_si_001.zip [file ed4c00479_si_001.zip › Supporting Information/CHM310 Fall 2018 Syllabus.pdf]

CHM310: Environmental Chemistry
Fall 2018

Schedule
Lecture: Tues/Thurs 2:00-3:00, LM159
Office hours: Wed 2:30 – 3:30, LM119

Contact Information
Prof. Jessica D'eon, LM 119, (416) 978-7283, jessica.deon@utoronto.ca

Course Overview
There is an environmental cost to our modern lifestyles. Chemicals surround us in our everyday lives (medications, personal care products, flame retardants, refrigerants…) and because of this they are present in the environment and in ourselves. In this class we will explore the fate of chemicals in the environment and the body to understand how to design chemicals without adverse effects or environmental persistence. Climate change is arguably the most pressing environmental issue we are currently facing and so changes in climate forcing need to be included within the scope of environmental effects.

Learning Objectives

1. As a CHM310 student you will be able to predict ***where*** in the environment (air, water, soil, biota…) an organic chemical would be expected to be found as well as ***how*** it might undergo degradation using only its chemical structure…
2. Climate change is a major topic in CHM 310, upon completion of this class you will be able to assess the potency of a greenhouse gas, understand the origin of climate data, and feel comfortable discussing the science of climate change in lay terms

Grading

310 Environmental Working Group Project (310-EWG)		45 %
Assignment 1: *Atmospheric Lifetime*	5 %	
Assignment 2: *Radiative Efficiency & GWP*	5 %	
Assignment 3: *Environmental Fate*	5 %	
Assignment 3: *Chemical Fate Model*	5 %	
Boardroom-Style Presentation		
Group Presentation Grade	5 %	
Individual Presentation Grade	5 %	
Peer-evaluation	3%	
Final Recommendation	12 %	
Midterm (Tuesday October 30th)		15 %
Final exam (cumulative, during finals period)		40 %

Course website
All material including presentation slides and literature resources, when provided, will be available on Quercus. You are responsible for checking this site regularly for announcements and content. I will be posting relevant slides at least 24 hours before class. I will complete these notes with you in class, and will be posting the completed notes together with lecture recordings on the course website.

Discussion Board
There will be a discussion board available on Quercus for each unit of the class. Please feel free to post questions and answers. Prof. D'eon will moderate the discussion board every Monday and Wednesday.

Lecture Schedule

Dates			Due Dates	Topics Discussed
September	6	R		Introduction
	11	T		**U1:** *Atmospheric Oxidation*
	13	R		
	18	T		
	20	R		**U2:** *Interaction between Molecules and Electromagnetic Radiation*
	25	T	310-EWG 1	
	27	R		Dr. Mima Staikova Guest Lecture on Gaussian 09 Calculations
October	2	T		**U4:** *Nucleophilic Reactions in the Aqueous Environment*
	4	R		
	9	T	310-EWG 2	**U5:** *Reduction in the Anoxic Environment*
	11	R		**U6:** *Partitioning of Organic Contaminants*
	16	T		
	18	R		
	23	T	310-EWG 3	**U7:** *What Makes a Chemical Toxic?*
	25	R		
	30	T	**Midterm**	
November	1	R		**U8:** *The Earth System and Climate Feedback Cycles*
	6	T	**Reading Week**	
	8	R		
	13	T	310-EWG 4	**U9:** *Isotopes and the Geological Record*
	15	R		
	20	T	310-EWG Presentations	
	22	R		**U10:** *The Long-term and Short-term Carbon Cycles*
	27	T		
	29	R		**U11:** *Geoengineering Techniques and Abatement Strategies*
December	4	T	310-EWG Final	Review

Academic Integrity

While I encourage you to discuss your understanding of course material with others, any material that you submit or present MUST represent your own independent work and comprehension. Information about academic integrity can be found here: http://www.artsci.utoronto.ca/osai/

Accommodations

Students with diverse learning styles and needs are welcome in this course. In particular, if you have a disability or health consideration that may require accommodations, please feel free to approach me and/or Accessibility Services at (416) 978 8060; http://accessibility.utoronto.ca

Absences

Students who miss a test or assignment deadline should contact Professor D'eon as soon as possible, and no later than one week after returning to class. A legitimate reason for an absence or missed deadline due to medical, personal, or family reasons should be documented by one of the following: 1) U of T Student Medical Certificate; 2) Student Health or Disability Related Certificate; 3) College Registrar's Letter; or 4) Accessibility Services Letter. In the absence of a legitimate reason for missing a test or assignment you will receive a grade of 0 on the test, and a deduction of 10% per day (including weekends) for assignments.
