# Supplementary material for: Welcome to 310 Environmental Working Group! A Group Project That Places Students in the Role of Consultants Helping Businesses Choose the Most Climate Friendly Fluorinated Gas
Source: J Chem Educ. 2024 Sep 6;101(10):4203–13. doi: 10.1021/acs.jchemed.4c00479 (PMC11465463; doi:10.1021/acs.jchemed.4c00479)
Supplement: Supplementary file 1 — ed4c00479_si_001.zip [file ed4c00479_si_001.zip › Supporting Information/Statement of Work and Sample Contracts/310 EWG Group 1 Hospital.docx]

| 310 Environmental Working Group |  |
| --- | --- |

Agreement to Perform Consulting Services to 310 Environmental Working Group

Contract

| Date | Services Performed By: | Services Performed For: |
| --- | --- | --- |
| September 14, 2018 | Monica Brundage  Travis Garvin  Joshua Graham  Veronica McKelvey  Erinda Moglica | 310-EWG |

Humber River Hospital is looking for an environmental assessment of two of the inhaled anesthetics used in their facility. Chemical details are given below. You will use four assignments over the next 10 weeks to assess the environmental impact of these compounds both in terms of their effect on climate and their propensity to form persistent degradation products.

You will present the results of your assessment to hospital board members from 10 am to 12 pm on Monday November 19^th^ in LM 108. A final report of these findings is also to be submitted by 2 pm on Tuesday December 4^th^.

|  | **Formula** | **CAS #** | **Molecular Weight (g/mol)** |
| --- | --- | --- | --- |
| **Chemical 1** | (CF_3_)_2_CHOCH_2_F | 28523-86-6 | 200.05 |
| **Chemical 2** | CHF_2_OCHFCF_3_ | 57041-67-5 | 168.04 |
