# Supplementary material for: Welcome to 310 Environmental Working Group! A Group Project That Places Students in the Role of Consultants Helping Businesses Choose the Most Climate Friendly Fluorinated Gas
Source: J Chem Educ. 2024 Sep 6;101(10):4203–13. doi: 10.1021/acs.jchemed.4c00479 (PMC11465463; doi:10.1021/acs.jchemed.4c00479)
Supplement: Supplementary file 1 — ed4c00479_si_001.zip [file ed4c00479_si_001.zip › Supporting Information/Statement of Work and Sample Contracts/310 EWG Statement of Work.docx]

| 310 Environmental Working Group |  |
| --- | --- |

Agreement to Perform Consulting Services to 310 Environmental Working Group

Statement of Work

| Date | Services Performed By: | Services Performed For: |
| --- | --- | --- |
| September 6, 2018 | CHM 310 Students | 310-EWG |

Welcome to the *310 Environmental Working Group (310-EWG)*. 310-EWG is a not-for-profit environmental advocacy organization with a mandate to provide commercial users of industrial chemicals with the relevant background knowledge to make environmentally-friendly decisions. You have been contracted to work on our “Greening Fluorinated Organics” project that provides users of these materials with information regarding their environmental impact so they can make informed buying and use decisions. As part of this project you will be given a client (e.g. hospital, car manufacturer, cosmetics manufacturer, etc.) and two chemicals that can be used for similar purposes. Your job will be to evaluate these two chemicals with respect to their impact on climate and their potential to produce long-lived degradation products. Your project team will be composed of 4-5 class members. The project will include four specific assignments each with their own deadline.* You will then come together as a team and using the information in the assignments decide on a recommendation for your client. This recommendation will be presented to your client during the week of November 19^th^, 2018 and a final report summarizing your conclusions submitted on December 4^th^, 2018.

*note: you are welcome to work together but each student must submit an individual assignment that represents their own work)

Deliverables

By Friday September 14^th^, you must…

- Sign up for a presentation time slot on Quercus. This will determine your consulting team and your assigned HFCs. Presentations will take place at the following times:
  - Monday November 19^th^ 10:00 am – 12:00 pm [LM 108]
  - Monday November 19^th^ 2:00 – 4:00 pm [LM 128]
  - Tuesday November 20^th^ 10:00 am – 12:00 pm [LM 108]
  - Wednesday November 21^st^ 10:00 am – 12:00 pm [LM 108]
- Complete the introductory quiz on climate change and ozone depletion on Quercus. (You will receive a 0.5% bonus on your final grade regardless of the content of your answers.)
- Read the article by Velders *et al.* posted on Blackboard: “Preserving Montreal Protocol Climate Benefits by Limiting HFCs”, *Science* **2012**, *335*, 922-923.

Assignment 1: *Atmospheric Lifetime* – Use a structure-activity relationship to predict atmospheric lifetimes and oxidation products of the HFCs. [**Tuesday September 25^th^**]

Assignment 2: *Radiative Efficiency & Global Warming Potential* – Determine radiative efficiencies using IR spectra calculated in Gaussian 09 and calculate the global warming potential (GWP) for each assigned chemical. [**Tuesday October 9^th^**]

Assignment 3: *Environmental Fate* – Predict the environmental fate of the oxidation products. [**Tuesday October 23^rd^**]

Assignment 4: *Chemical Fate Model* – Code and run a chemical fate model in Excel. [**Tuesday November 13^th^**]

Boardroom Presentation: Deliver a 15-minute team presentation on your final recommendations to your client, who will be played by the students if other groups in your time slot. Clients who ask questions will be awarded a bonus mark. [**Nov 19-21**]

Individual Report: Summarize the important findings from your assignments and explain your ultimate recommendation to the company. [**Tuesday December 4^th^**]
