# Supplementary material for: Welcome to 310 Environmental Working Group! A Group Project That Places Students in the Role of Consultants Helping Businesses Choose the Most Climate Friendly Fluorinated Gas
Source: J Chem Educ. 2024 Sep 6;101(10):4203–13. doi: 10.1021/acs.jchemed.4c00479 (PMC11465463; doi:10.1021/acs.jchemed.4c00479)
Supplement: Supplementary file 1 — ed4c00479_si_001.zip [file ed4c00479_si_001.zip › Supporting Information/Assignment 1/310 EWG Assignment 1 Fall 2018.docx]

| 310 Environmental Working Group |  |
| --- | --- |

Assignment 1

Deliverables

In this assignment, you will use the structure-activity relationship developed by Kwok and Atkinson (*Atmos. Env.* 1995, *29*, 1685-1695) to predict rate constants (*k*) for the reaction of your chemicals with hydroxyl radicals (OH). This method is a useful predictive tool, and it also demonstrates how specific molecular moieties affect reaction with OH. If a chemical has more than one possible site of reaction with OH the overall rate constant is the sum of the rates constants for each site. Let’s use the reaction of 1-chloropropane (CH_3_CH_2_CH_2_Cl) as an example. 1-chloropropane has three possible sites for H-abstraction.

CH_3_CH_2_CH_2_Cl + OH 🡪 products

Rate (overall) = Rate (CH_3_–) + Rate (–CH_2_–) + Rate (–CH_2_Cl)

If we change these rates into their corresponding rate laws we get:

*k*_overall_ [OH][CH_3_CH_2_CH_2_Cl] = *k*_-CH3_ [OH][CH_3_CH_2_CH_2_Cl] + *k*_-CH2-_ [OH][CH_3_CH_2_CH_2_Cl] + *k*_-CH2Cl_ [OH][CH_3_CH_2_CH_2_Cl]

We can see from this relationship that we can calculate the overall rate constant for the reaction of 1-chloropropane with OH by adding the rate constants for the individual sites.

*k*_overall_ = *k*_-CH3_ + *k*_-CH2-_ + *k*_-CH2Cl_

This is how the structure activity relationship works. For saturated hydrocarbons (no double bonds) the rate constant of H-abstraction from each site is first defined by assigning the carbon as primary, secondary, or tertiary as defined by Kwok and Atkinson.

1. Carbons with only one substituent (X) aside from hydrogen are called primary carbons and their rate constant is define by the following expression:

 *k*_prim_ = (1.36 x 10^-13^ cm^3^ molecule^-1^ s^-1^) (F(X))

1. Carbons with two substituents (X, Y) aside from hydrogen are called secondary carbons and their rate constant is defined by the following expression:

*k*_sec_ = (9.34 x 10^-13^ cm^3^ molecule^-1^ s^-1^) (F(X)) (F(Y))

1. Carbons with three substituents (X, Y, Z) aside from hydrogen are called tertiary carbons and their rate constant is defined by the following expression:

*k*_tert_ = (1.94 x 10^-12^ cm^3^ molecule^-1^ s^-1^) (F(X)) (F(Y)) (F(Z))

To determine the rate constant for an organic compound with OH you will need to look up the relevant substituent factors (F(X), F(Y), F(Z)), which can be found in Table 2 on page 1688 of the Kwok and Atkinson paper.

*k*_-CH3_ = *k*_prim_ (F(-CH_2_-)) = (1.36 x 10^-13^ cm^3^ molecule^-1^ s^-1^) (1.23) = 1.67 x 10^-13^ cm^3^ molecule^-1^ s^-1^

*k*_-CH2-_ = *k*_sec_ (F(-CH_3_)) (F(-CH_2_Cl)) = (9.34 x 10^-13^ cm^3^ molecule^-1^ s^-1^) (1.00) (0.36) = 3.36 x 10^-13^ cm^3^ molecule^-1^ s^-1^

*k*_-CH2Cl_ = *k*_sec_ (F(-CH_2_-)) (F(-Cl)) = (9.34 x 10^-13^ cm^3^ molecule^-1^ s^-1^) (1.23) (0.38) = 4.37 x 10^-13^ cm^3^ molecule^-1^ s^-1^

**Questions**

This assignment includes both an online submission and an in-class hard copy. All solutions will submitted online by completing the word document entitled “310 EWG Assignment 1 Fall 2018 Report Sheet.docx” and submitting you solutions online through Quercus. Your submitted report sheet should be renamed as “Surname_FirstName_310EWG_Assign1.docx”. In addition to this report sheet you must submit a hard copy of your mechanism (Question 7) for both of your chemicals. In addition to the mechanism you may also include relevant calculations in your hard copy to receive any relevant partial credit. Be sure to include your name and student number on the in-class submission.

1. Write out the molecular formula for each of your assigned chemicals. On this structure please number the carbons that contain sites that are reactive to atmospheric oxidation (e.g. hydrogenated carbons or carbons that are part of a double bond) using the following nomenclature: CF3^1^CFHCF2O^2^CH2CF3, where the CHF is labeled C1 and the CH2 is labeled C2.
2. Use the Kwok and Atkinson structure activity relationship to calculate the rate constant for H-abstraction by hydroxyl radicals (OH) from each distinct carbon environment in the molecule. (**2 marks**)
3. If there is more than one reaction site in each of your molecules, what is the product distribution between reaction at the two sites? (**2 marks**)
4. Choose one of your molecules that contains more than one reactive site and with reference to the explain why one site is more reactive than another or why they are similarly reactive. The Kwok and Atkinson structure activity relationship is not perfect and so if you do not agree with the product distribution calculated using this method feel free to say so and explain your reasoning. (**2 marks**)
5. Using the rate constant from Q2, calculate the overall atmospheric lifetime in years with respect to reaction with OH for your assigned chemicals in the presence of a mean OH concentration of 1 x 10^6^ molecules cm^-3^. (**2 marks**)
6. In addition to reaction with hydroxyl radicals both the new and legacy chemicals are lost from the troposphere via diffusion into the stratosphere. Using a lifetime of 100 years for diffusion into the stratosphere what is the overall lifetime of your compound in the troposphere? (**2 marks**)
7. For each chemical draw out the mechanism following H-abstraction by OH to the first ***closed-shell*** species (not a radical) for each site of reaction. For full credit please include ***all*** closed-shell products on the attached solution sheet for ***all*** reaction sites on your molecules as well as a handing in a hard copy of your mechanism in class. (**4 marks**)
8. A reaction that is diffusion controlled (meaning it occurs with every collision) with respect to OH has a rate constant of 3 x 10^-10^ cm^3^ molecule^-1^ s^-1^, use this information to calculate how many collisions, on average, OH makes with each of your chemicals before there is a reaction. (**2 marks**)
9. Both of your chemicals contain an ether functional group how does this functionality affect the atmospheric lifetime of these compounds? Explain your answer. (**2 marks**)
10. The calculations performed here will be used in assignment 2 to calculate global warming potential (GWP). GWP is a thought experiment to assess a chemical’s climate impact. In this thought experiment 1 Kg of a chemical is released into the atmosphere, is instantaneously well mixed, and its effects on climate are measured relative to the same scenario for carbon dioxide over a given time horizon, typically 100 years.
    1. For each of your chemicals calculate the number of moles in 1 Kg. (**1 mark**)
    2. Assuming there are 1.7 x 10^20^ moles of gas in the atmosphere, what is the concentration of each of your chemicals in units of parts per billion (ppb)? (**1 mark**)
