# Supplementary material for: Welcome to 310 Environmental Working Group! A Group Project That Places Students in the Role of Consultants Helping Businesses Choose the Most Climate Friendly Fluorinated Gas
Source: J Chem Educ. 2024 Sep 6;101(10):4203–13. doi: 10.1021/acs.jchemed.4c00479 (PMC11465463; doi:10.1021/acs.jchemed.4c00479)
Supplement: Supplementary file 1 — ed4c00479_si_001.zip [file ed4c00479_si_001.zip › Supporting Information/Assignment 1/310 EWG Assignment 1 Fall 2018 Report Sheet.docx]

**Name:** **Student Number:**

| **Presentation**  **Timeslot (highlight one):** | Mon Nov 19^th^  10 am – 12 pm | Mon Nov 19^th^  2 – 4 pm | Tues Nov 20^th^  10 am – 12 pm | Wed Nov 21^st^  10 am – 12 pm |  |
| --- | --- | --- | --- | --- | --- |

**Consulting Group (highlight one):** 1 2 3

| **Questions** | | | **Chemical 1** | **Chemical 2** |
| --- | --- | --- | --- | --- |
| **Q1 (molecular formula)** | | |  |  |
| **Q2 (cm^3^ molecules^-1^ s^-1^)** | | **C1** |  |  |
|  |  | **C2** |  |  |
| **Q3 (% distribution)** | | **C1** |  |  |
|  |  | **C2** |  |  |
| **Q4** | **Structure** | |  | |
|  | **Explanation** | |  | |
| **Q5 (years)** | | |  |  |
| **Q6 (years)** | | |  |  |
| **Q7 (chemical structure(s) + mechanism submitted as hard copy during class)** | | |  |  |
|  |  |  |  |  |
| **Q8 (# of collisions)** | | |  |  |
| **Q9 (explanation)** | | |  | |
| **Q10a (moles)** | | |  |  |
| **Q10b (ppb)** | | |  |  |
