# Supplementary material for: Welcome to 310 Environmental Working Group! A Group Project That Places Students in the Role of Consultants Helping Businesses Choose the Most Climate Friendly Fluorinated Gas
Source: J Chem Educ. 2024 Sep 6;101(10):4203–13. doi: 10.1021/acs.jchemed.4c00479 (PMC11465463; doi:10.1021/acs.jchemed.4c00479)
Supplement: Supplementary file 1 — ed4c00479_si_001.zip [file ed4c00479_si_001.zip › Supporting Information/QM Calculation Details and class presentation/Calculating IR chm310_w16 .pdf]

Calculations of IR spectra
using Gaussian09 suit of programs and WebMO interface

Summary

Each student has to perform two calculations on his/her structure:

Job 1: Build the most stable conformer and send it for calculation using:

Calculation type: Optimization
Theory: B3LYP
Basis set: Basic: 3-21G
Charge: 0
Multiplicity: singlet

Job 2: Use the optimized structure from Job 1 and send it for calculation with:

Calculation type: Optimize + Vib Freq
Theory: B3LYP
Basis set: Accurate: 6-311+G (2d,p)
Charge: 0
Multiplicity: singlet

From the results, report the vibrational frequencies (in the appropriate range), the corresponding intensities, the mode of each vibration, and the IR spectrum.

Procedures

Following the instructions provided in a separate file for the WebMO user ID and password, login to the WebMO site.

When you log in for the first time, your WebMO Job Manager will contain any jobs.

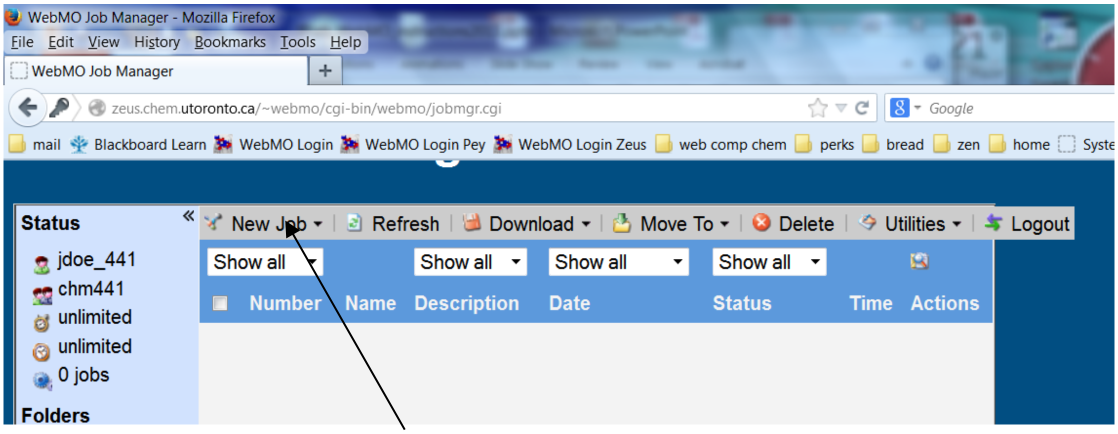

To start building your molecule with WebMO, go to “New Job”

Select the “New job” icon and the WebMO builder utility will open. Make sure the “Build” mode/ icon is active.

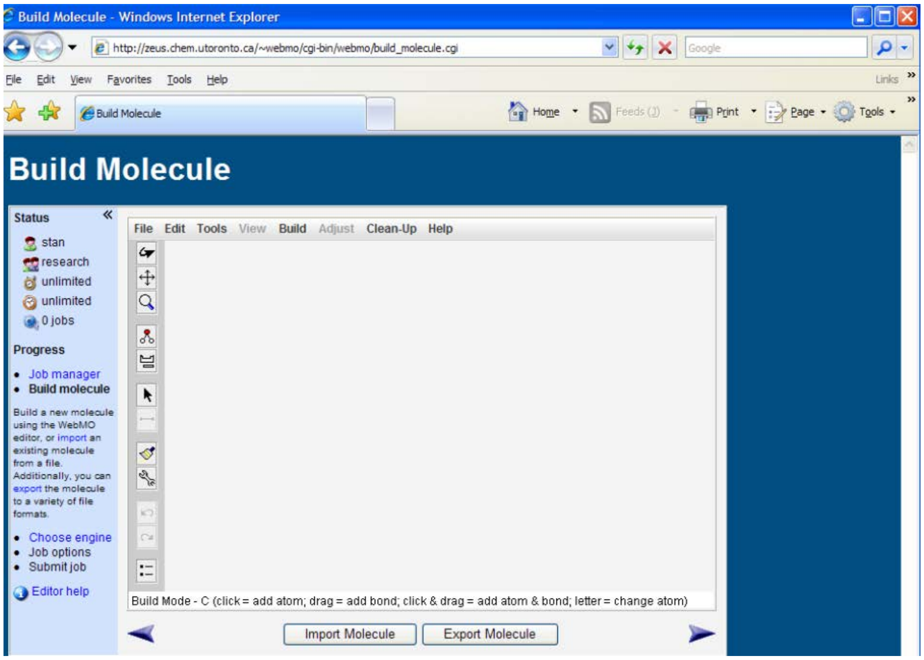

From “Build” menu select one element, as needed (C, H, O, N) or select “Other”, to open a periodic table, and select any other element from there.

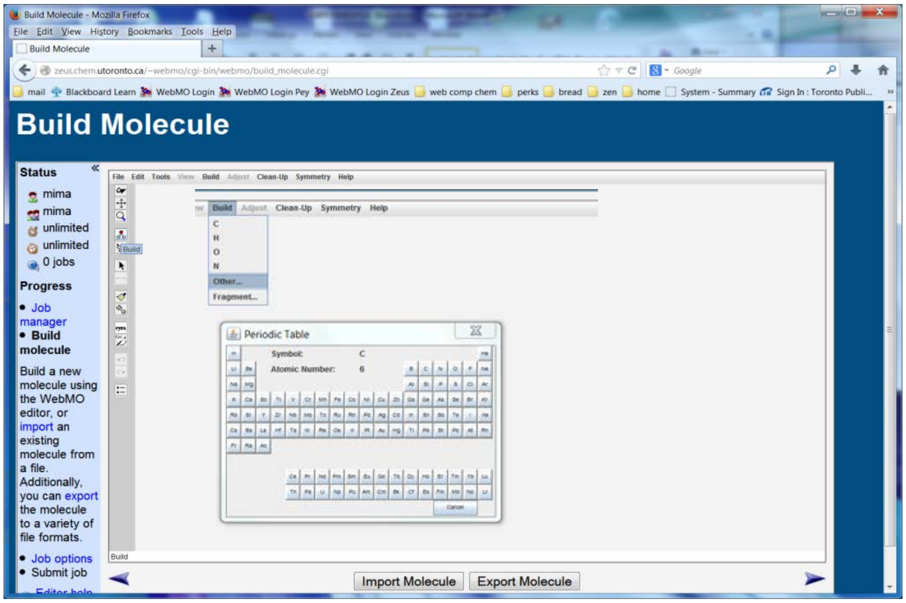

Draw your assigned structures, without adding the hydrogen atoms. To add the hydrogens, select the “Clean-Up” menu and then select “Add Hydrogens”. To adjust the bond lengths and angles of the structure you can select the “Geometry-Mechanics” from the “Clean-Up” menu, or the shortcut “Brush” or “Wrench” icon on the left panel. If you have not added the hydrogen atoms in the previous step, they will be added automatically now.

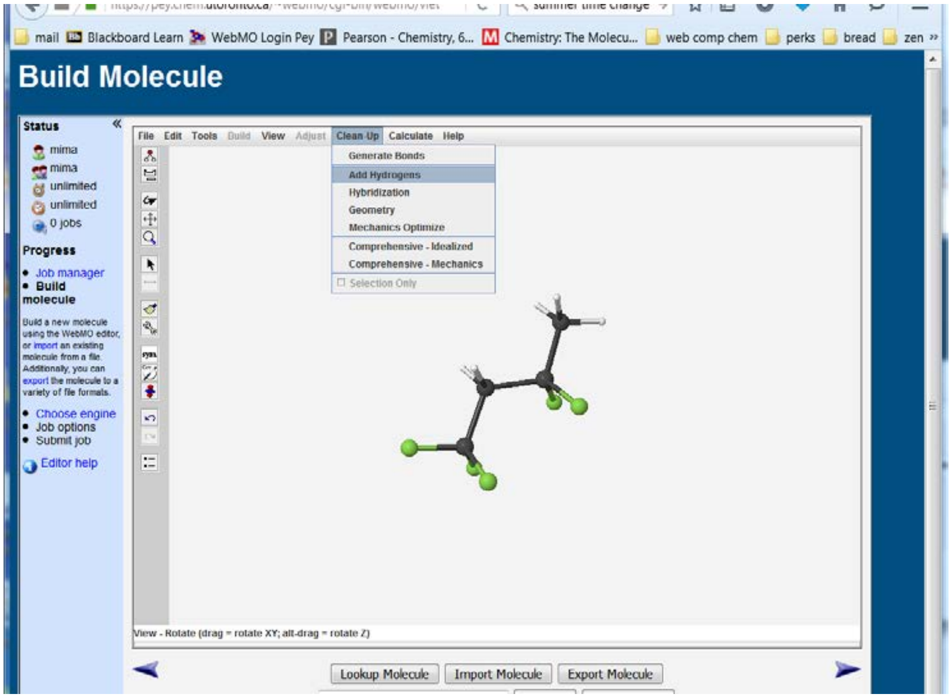

Using the bottom-right arrow in the above widow, go to the "Choose Computational Engine" window, select Gaussian 09, and "zeus-default", on the Select Queue drop box:

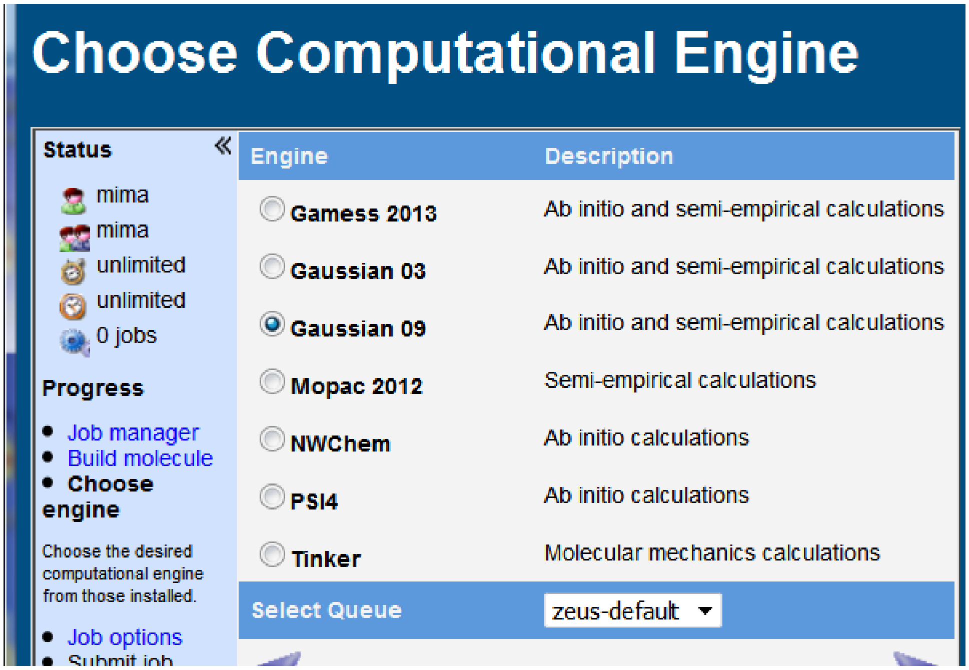

Proceed to "Configure Gaussian Job Options" window through the bottom-right arrow to define the parameters of your calculation:

For Job 1 use the parameters on the screen shot below:

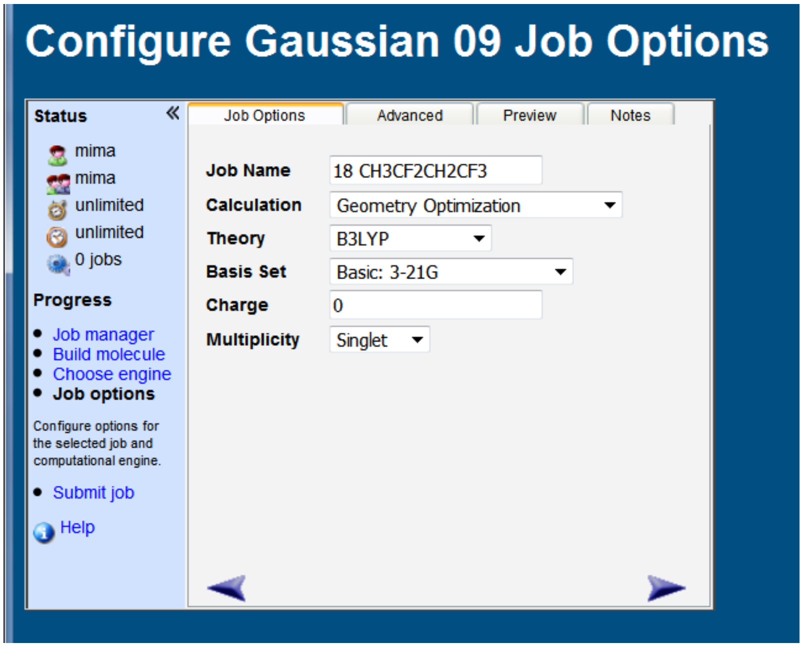

When you are satisfied that all the selections are correct, using the bottom-right arrow, send the job for calculation:

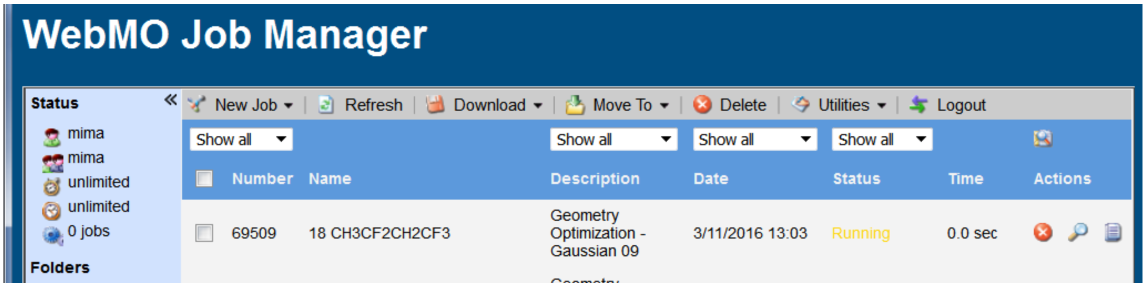

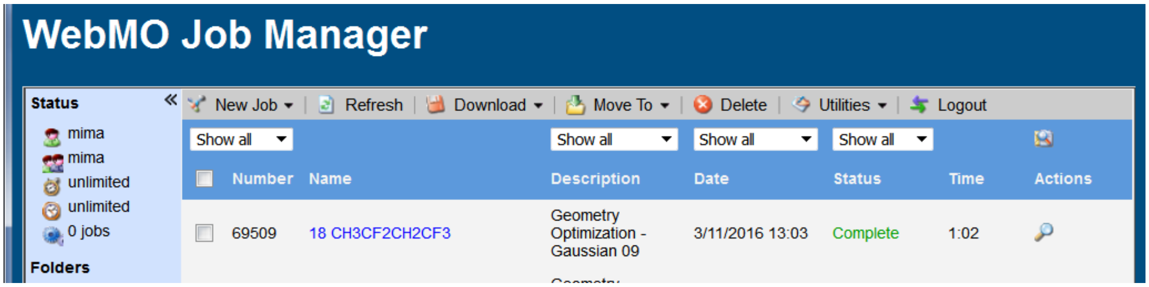

The job will appear first as "Running", and when done, will show a status of "Complete". You can log out and close your window at any time after a job is submitted; this will not affect your calculations.

To view and evaluate your results after the job is completed, you can click on the job name or the "Magnifying glass" icon.

Open the calculated Job 1 and inspect the structure to see if you have calculated indeed the most stable conformer. If you find out that the structure is not the most stable one, use "New Job

Using This Geometry" (see the screen shot below), remodel the structure and send it again at **Job 1 level** of calculation.

If you are satisfied that this is indeed the most stable conformer, proceed to sending **Job 2**.

To send your Job 2, and use the "New Job Using This Geometry":

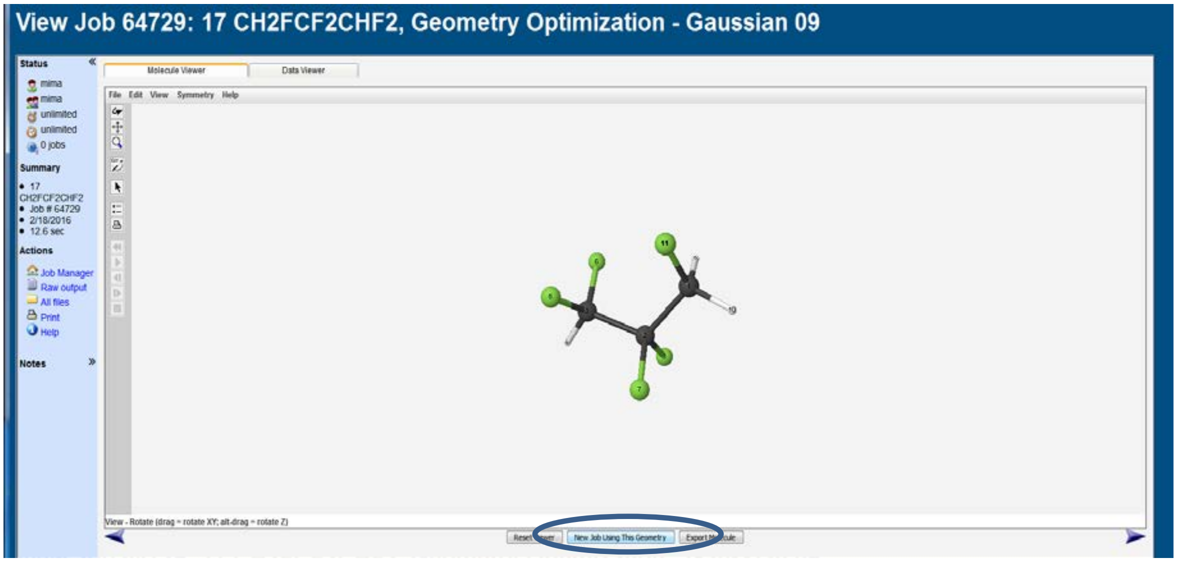

Without changing the structure, proceed to "Choose Computational Engine", and to "Configure Gaussian 09 Job Options". Select the options as shown on the screen shot below and send for calculation:

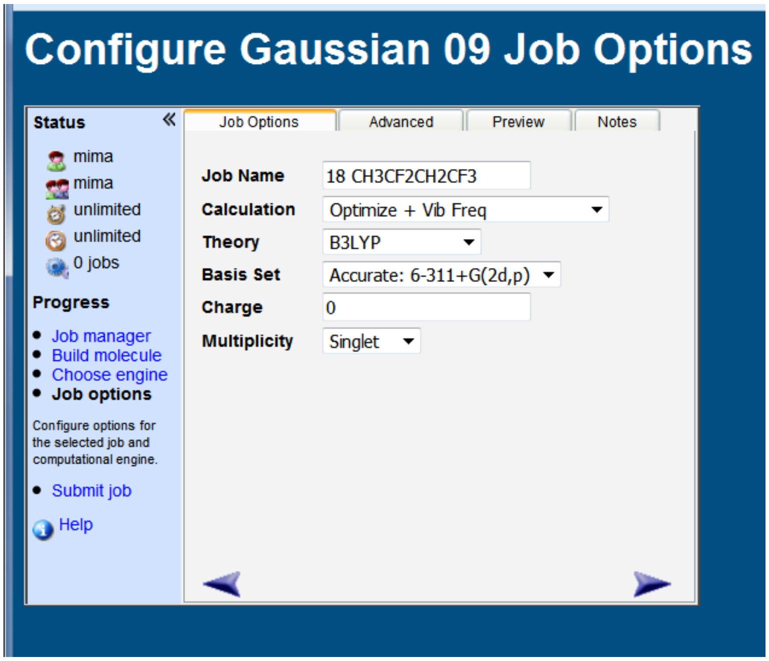

When the job is completed, the results are displayed as on the screen shoot below. Scroll down the page to reveal the calculated energies and properties of your structure:

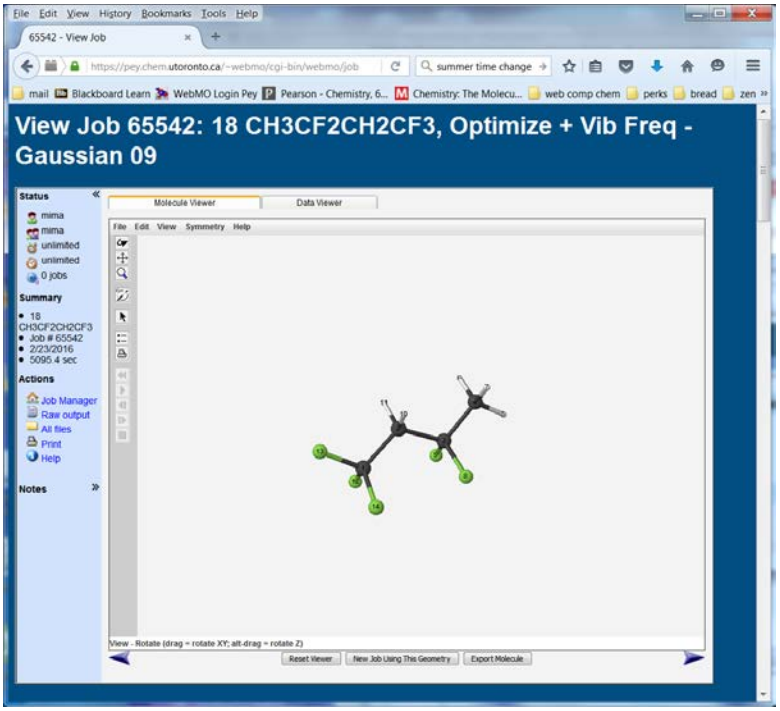

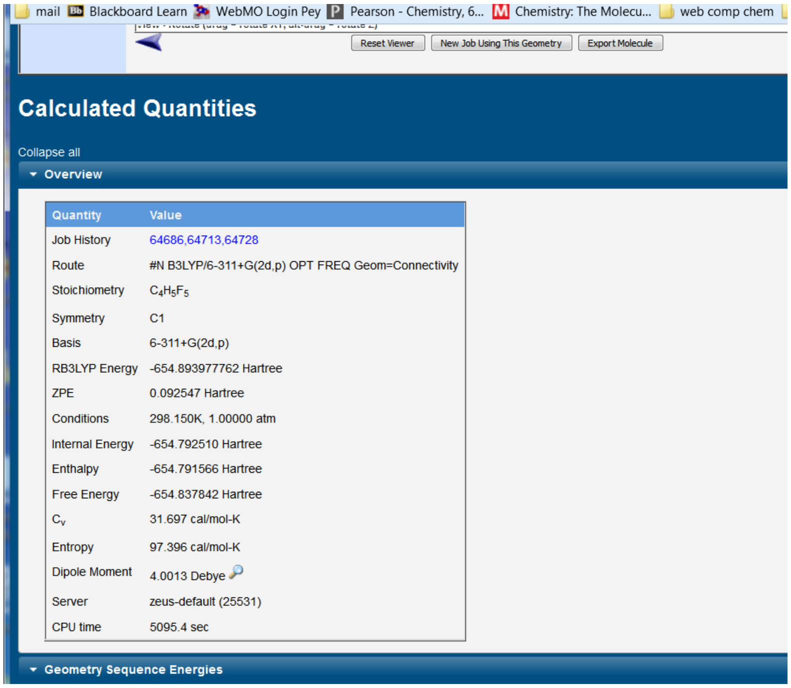

Quantity	Value
Job History	64686,64713,64728
Route	#N B3LYP/6-311+G(2d,p) OPT FREQ Geom=Connectivity
Stoichiometry	$C_4H_5F_5$
Symmetry	C1
Basis	6-311+G(2d,p)
RB3LYP Energy	-654.893977762 Hartree
ZPE	0.092547 Hartree
Conditions	298.150K, 1.00000 atm
Internal Energy	-654.792510 Hartree
Enthalpy	-654.791566 Hartree
Free Energy	-654.837842 Hartree
C_v	31.697 cal/mol-K
Entropy	97.396 cal/mol-K
Dipole Moment	4.0013 Debye
Server	zeus-default (25531)
CPU time	5095.4 sec

The last table in the output is the "Vibrational Modes"

https://pey.chem.utoronto.ca/~webmo/cgi-bin/webmo/job

mail Blackboard Learn WebMO Login Pey Pearson - Chemistry, 6... Che

Vibrational Modes

Mode	Symmetry	Frequency (cm^{-1})	IR (Raman) Intensity	Actions
1	A	4.5218	0.6227	
2	A	115.1972	0.4806	
3	A	154.2851	0.5289	
4	A	216.4681	0.0307	
5	A	280.0662	2.3437	
6	A	293.2215	0.0724	
7	A	308.6678	0.0011	
8	A	375.4458	0.3331	
9	A	424.6979	0.9310	
10	A	496.8705	10.7127	
11	A	516.5428	8.5467	
12	A	529.0150	1.3111	
13	A	578.7251	25.2345	
14	A	681.3468	6.7679	
15	A	817.0277	32.0702	
16	A	843.2054	6.9247	
17	A	889.6052	83.9885	
18	A	947.9302	0.9393	
19	A	961.9056	19.2867	
20	A	1070.4585	26.6021	
21	A	1082.8669	151.6738	
22	A	1202.0680	122.2711	
23	A	1209.9089	338.5527	
24	A	1229.7561	105.8687	
25	A	1285.4588	205.0610	
26	A	1316.4154	18.6723	
27	A	1410.5190	65.3670	
28	A	1421.9018	75.7511	
29	A	1463.2283	6.0323	
30	A	1486.0023	1.3483	
31	A	1487.1826	4.9161	
32	A	3053.9795	2.4216	
33	A	3080.1437	0.8076	
34	A	3123.6821	0.8606	
35	A	3131.3469	8.0315	
36	A	3133.6298	6.1527	
Frequency Scale Factor				1.0
Normal Mode Amplitude				1.0
Animation Speed				50
IR Spectrum				
Peak Width (cm^{-1})			40	

Quote

Data from the calculation, you need to report and use to finish your assignment:

Compound name	Calculated, B3LYP 6-311+G(2d,p)		Experimental frequency (if available)	Scaling factor	Type of vibration
	Scaled Frequency (Harmonic Frequency)	Intensity			

Copy only the frequencies in the appropriate range.
At the bottom of the "Vibrational modes" table change the "Peak Width (cm^{-1})" to 10 and click the "View" icon. The calculated IR spectrum will appear. You will need a copy of it for your assignment.

To record the intensities of the IR peaks, hover with the cursor on each of the visible peaks of the spectrum until you have the approximate value of the frequency. The value of the intensity appears next to the frequency. See the example below.

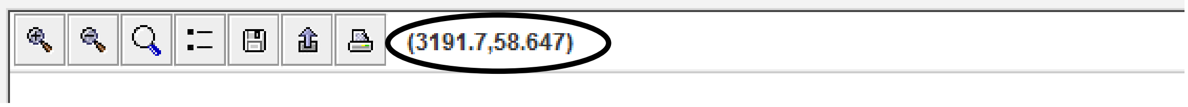

To see the vibrating atoms of each mode, use the "Animate" icon next to each frequency in the "Vibrational Modes" table. Use it to facilitate the IR peak assignments.
