# Supplementary material for: Welcome to 310 Environmental Working Group! A Group Project That Places Students in the Role of Consultants Helping Businesses Choose the Most Climate Friendly Fluorinated Gas
Source: J Chem Educ. 2024 Sep 6;101(10):4203–13. doi: 10.1021/acs.jchemed.4c00479 (PMC11465463; doi:10.1021/acs.jchemed.4c00479)
Supplement: Supplementary file 1 — ed4c00479_si_001.zip [file ed4c00479_si_001.zip › Supporting Information/QM Calculation Details and class presentation/Quantum Mechanics and Spectroscopy CHM310.pdf]

Quantum Mechanics and Spectroscopy

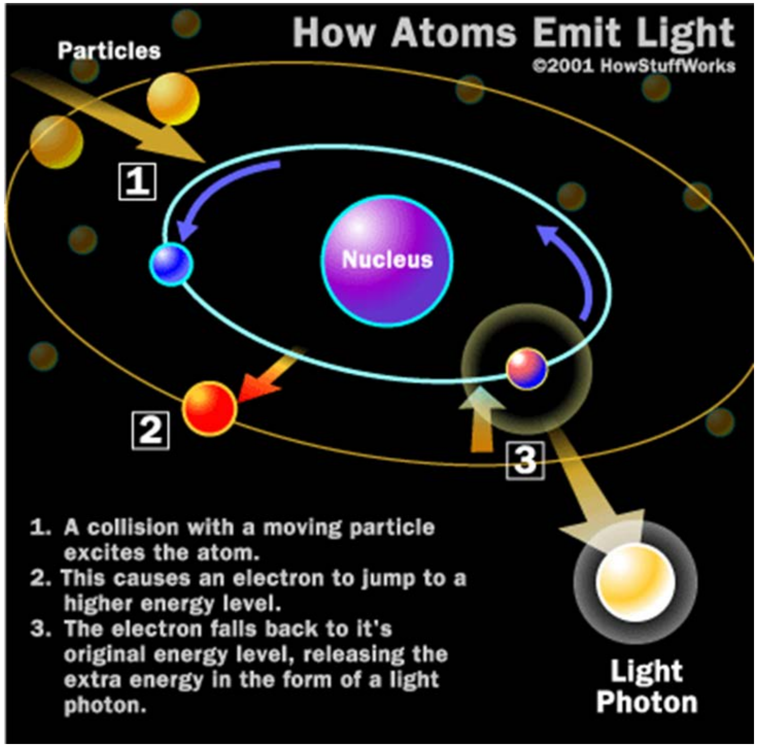

Dr. Mima Staikova

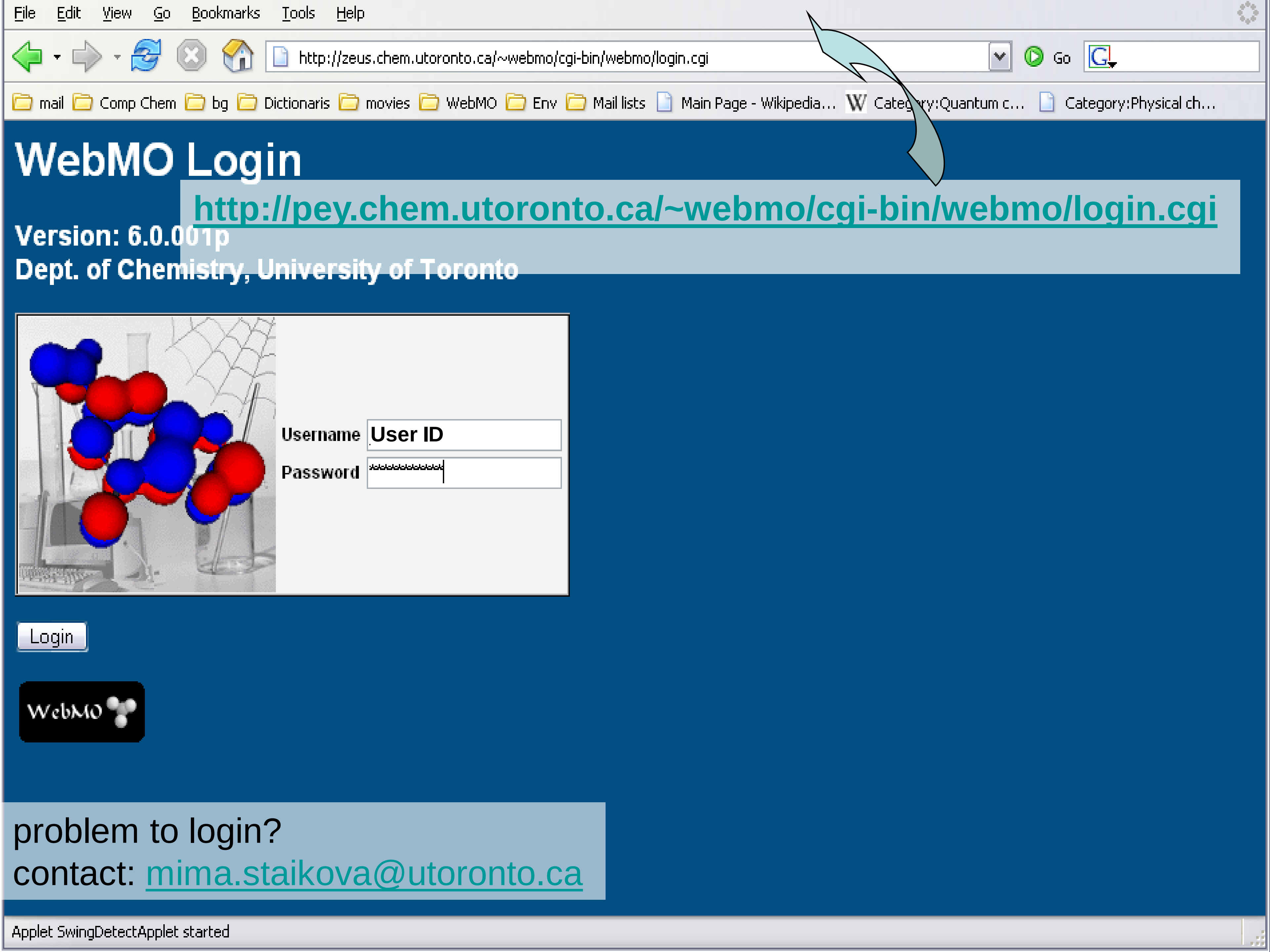

http://pey.chem.utoronto.ca/~webmo/cgi-bin/webmo/login.cgi

problem to login?

contact: mima.staikova@utoronto.ca

Molecular Energy

Molecules can have the following types of energy:

$$E_{molecule} = E_{translational} + E_{rotational} + E_{vibrational} + E_{electronic}$$

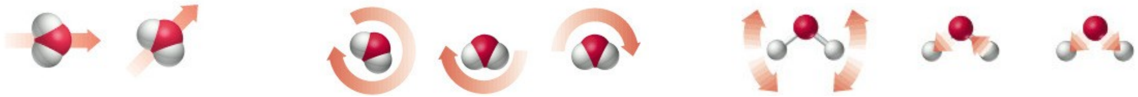

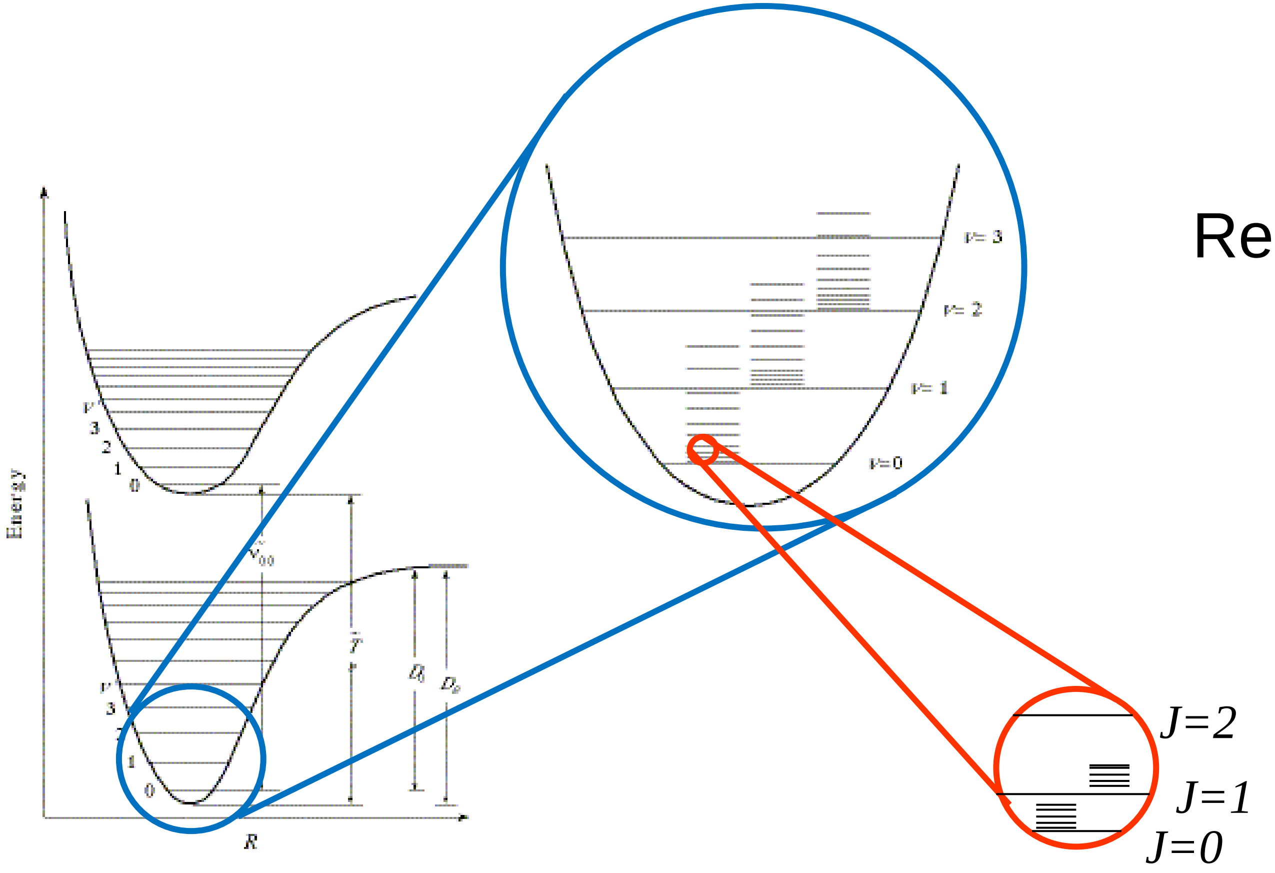

Relative energy scale

QUANTUM MECHANICS

The Schrödinger equation

$$\hat{H}\psi = E\psi$$

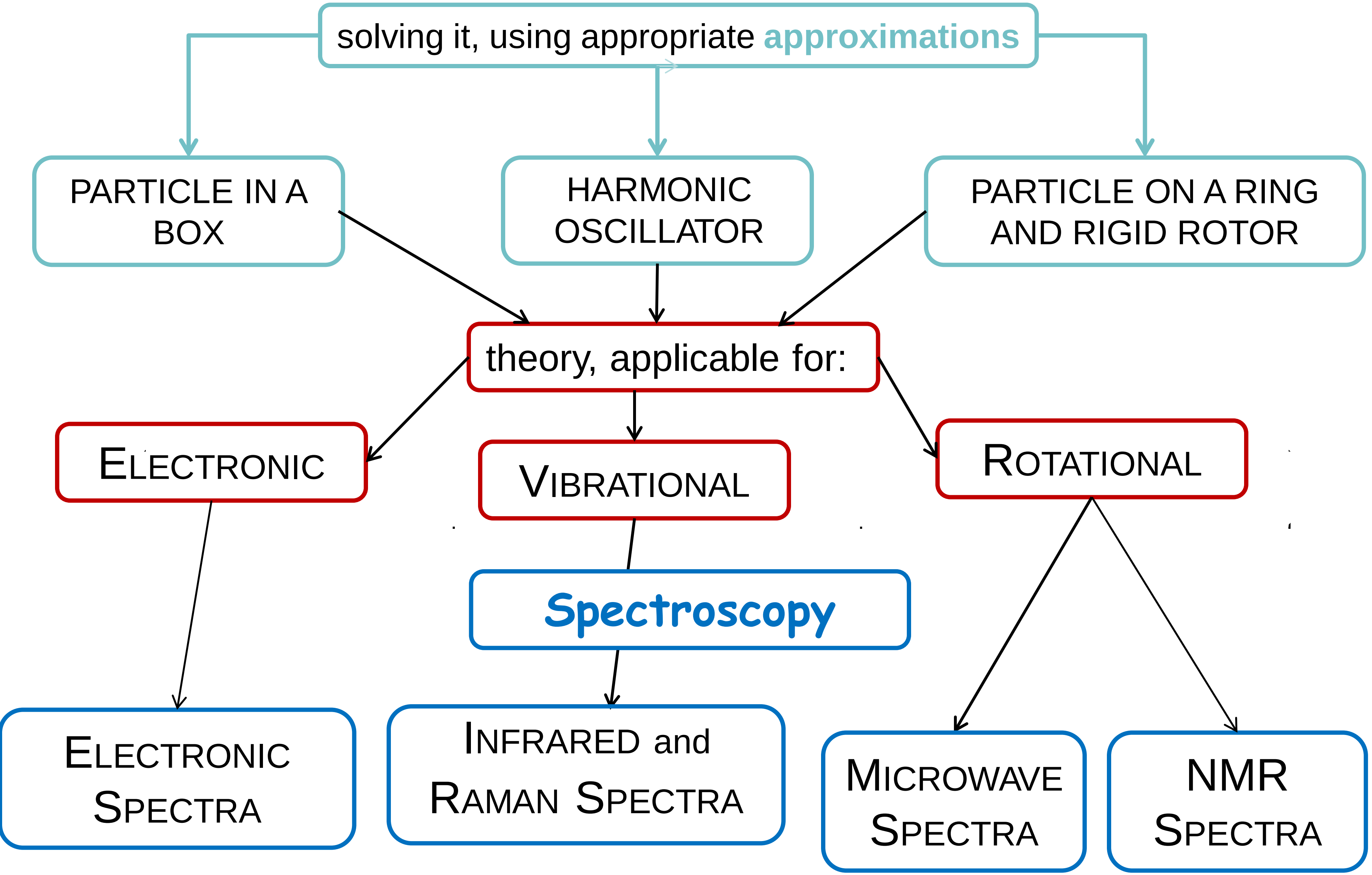

QUANTUM MECHANICS

The Schrödinger equation

$$\hat{H}\psi = E\psi$$

solving it, using appropriate **approximations**

HARMONIC OSCILLATOR

theory, applicable for:

VIBRATIONAL

Spectroscopy

INFRARED and RAMAN SPECTRA

absorption and emission

Both absorption and emission involve a transition between two quantized energy levels

$$\Delta E = E_2 - E_1 = h\nu$$

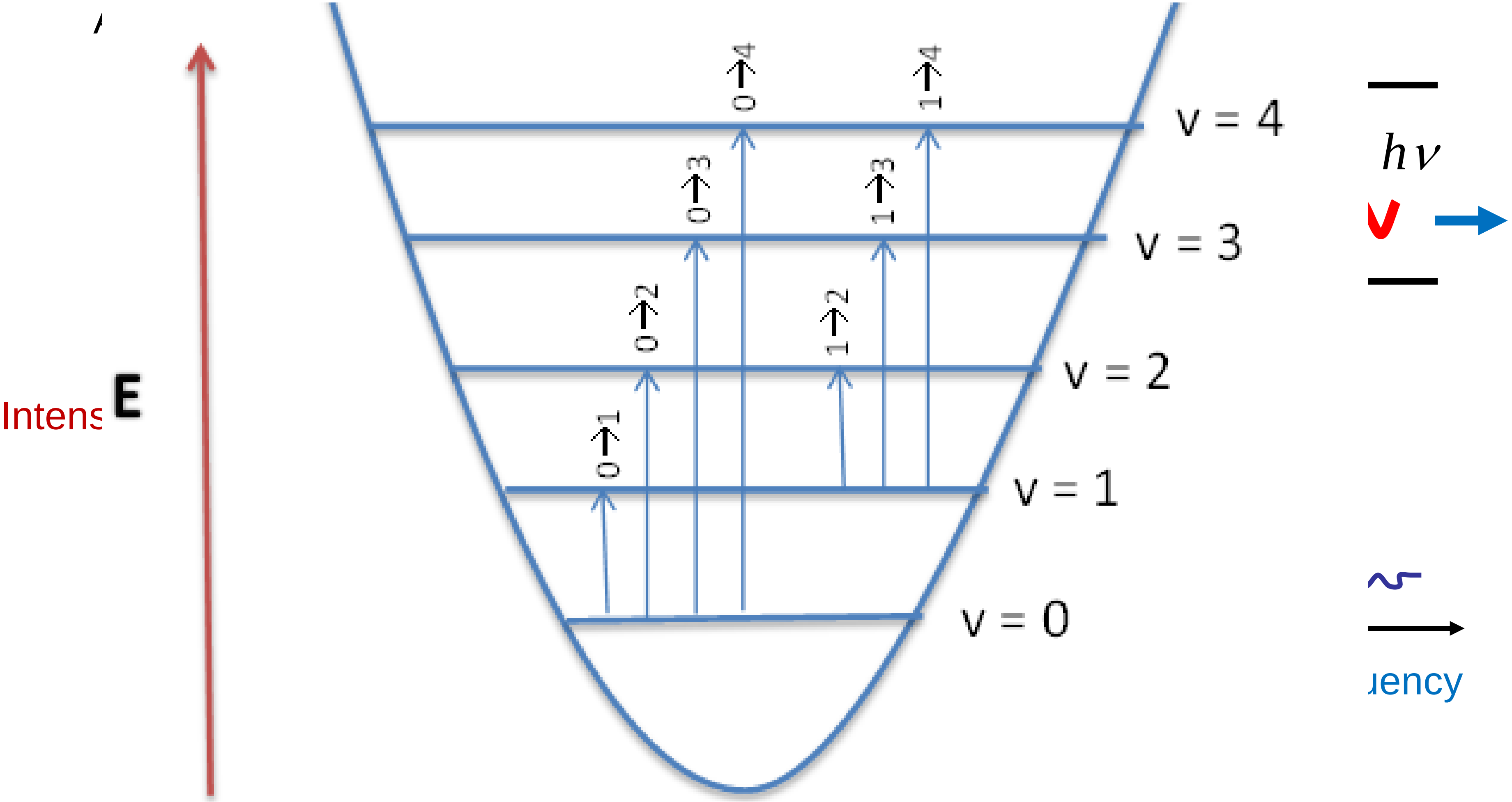

Line width and line resolution

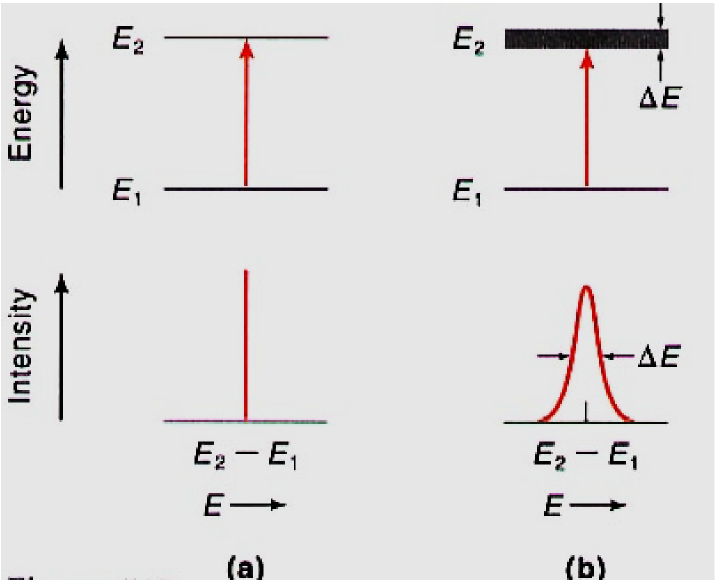

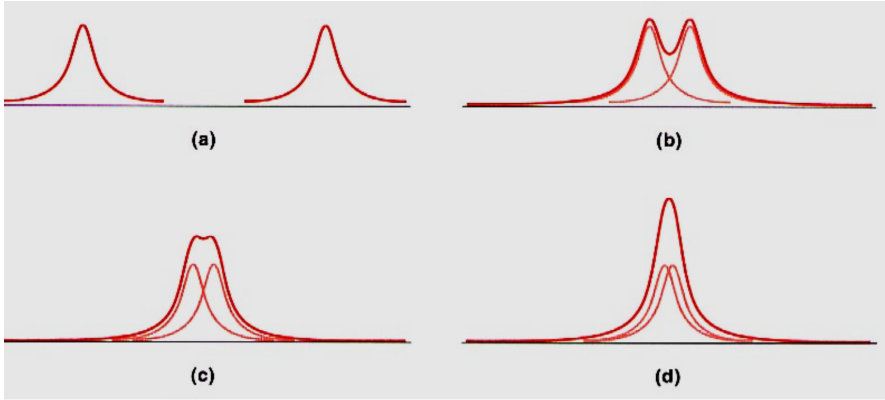

Line width

Spectral lines have a finite, nonzero width (b)

$$\Delta E \cdot \Delta t \geq \frac{h}{4\pi} \longrightarrow \Delta E \geq \frac{h}{4\pi \cdot \Delta t}$$

$$\frac{\Delta E}{h} \geq \Delta \nu = \frac{1}{4\pi \cdot \Delta t}$$

Line resolution

The separation between spectral lines

Resolving power R:

$$R = \frac{\lambda}{\Delta \lambda} = \frac{\nu}{\Delta \nu}$$

Background of infra red absorption

✓ IR radiation does not have enough energy to induce electronic transitions as seen with UV. Absorption of IR is restricted to compounds with small energy differences in the possible vibrational and rotational states.

✓ For a molecule to absorb IR, the vibrations or rotations within a molecule must cause a net change in the dipole moment of the molecule. The alternating electrical field of the radiation (remember that electromagnetic radiation consists of an oscillating electrical field and an oscillating magnetic field, perpendicular to each other) interacts with fluctuations in the dipole moment of the molecule.

✓ If the frequency of the radiation matches the vibrational frequency of the molecule then radiation will be absorbed, causing a change in the amplitude of molecular vibration.

Calculating IR Spectra

MOLECULAR VIBRATIONS: small periodic distortions form the equilibrium geometry of molecules.

IR spectroscopy concerns the vibrational motion of molecules.

A classical analogy is a simple *harmonic oscillator*

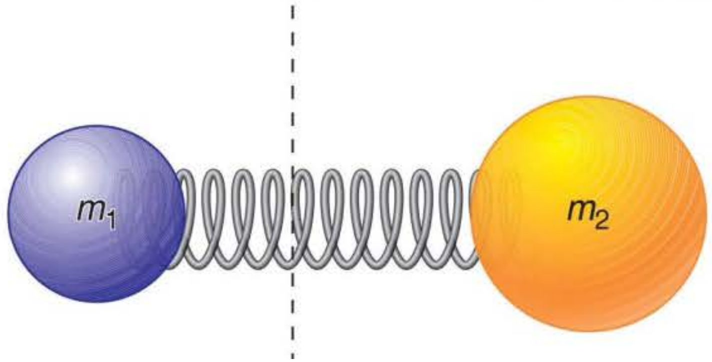

Simple Harmonic Oscillator

Potential energy of a molecule behaving like a harmonic oscillator $V = \frac{1}{2}k(r - r_e)^2$

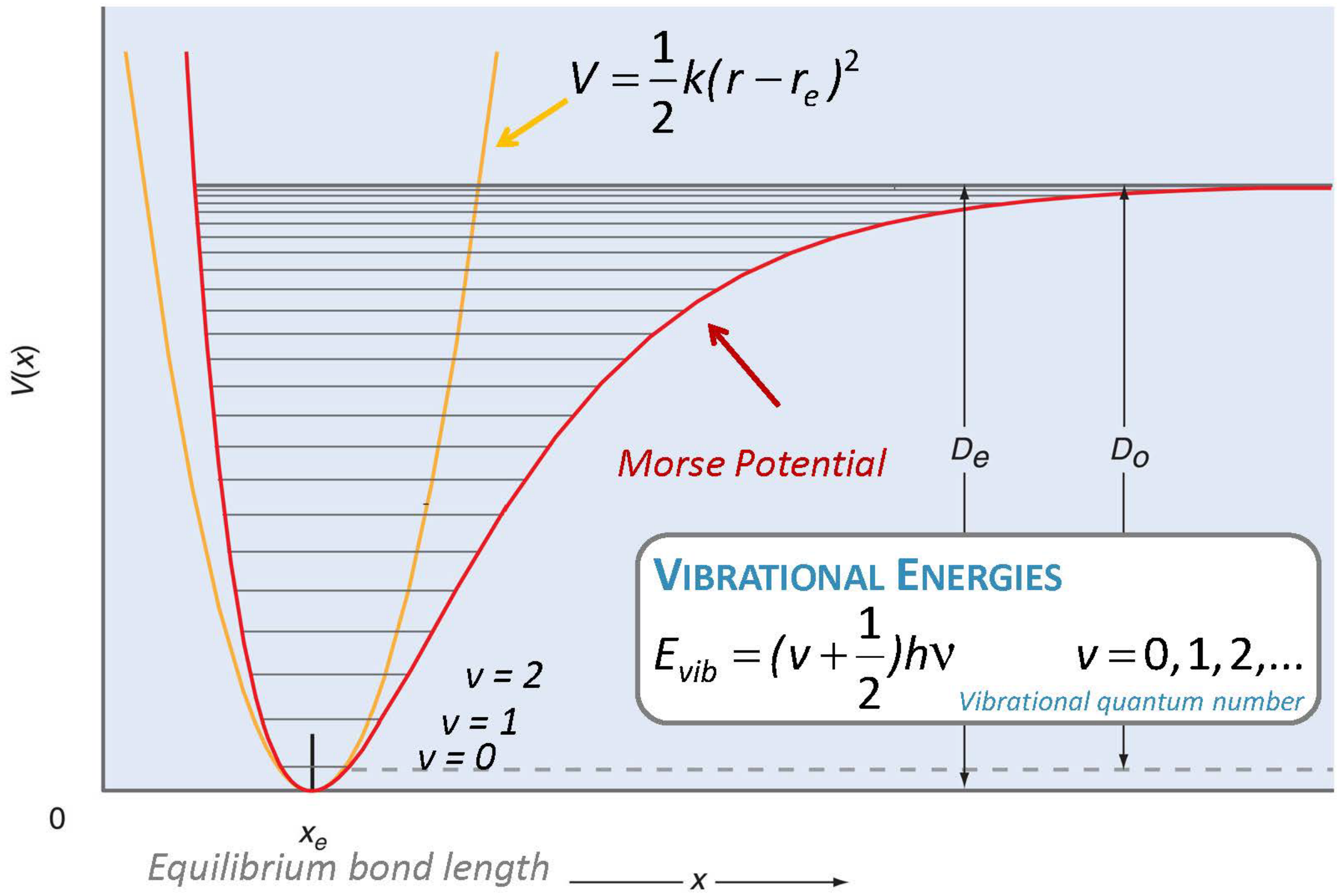

Real Molecular Potential

The real molecular potential differs form the simple harmonic potential. The spacing between successive levels decreases with increasing **v**, due to the *anharmonic* character:

$$E_{vib} = (v + \frac{1}{2})h\nu \; - x(v + \frac{1}{2})^2 h\nu$$

***x** = anharmonicity constant*
*(can be ignored except for large values of **v**)*

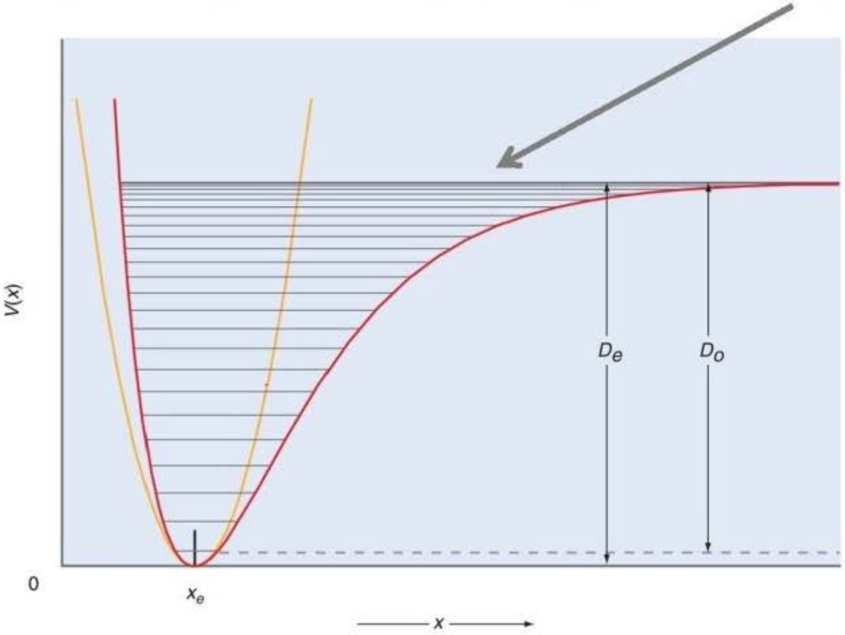

SELECTION RULE

$$\Delta v = \pm 1$$

OVERTONES

Consequence of anharmonicity

$$v = 0 \rightarrow 2, 0 \rightarrow 3$$

Comparison

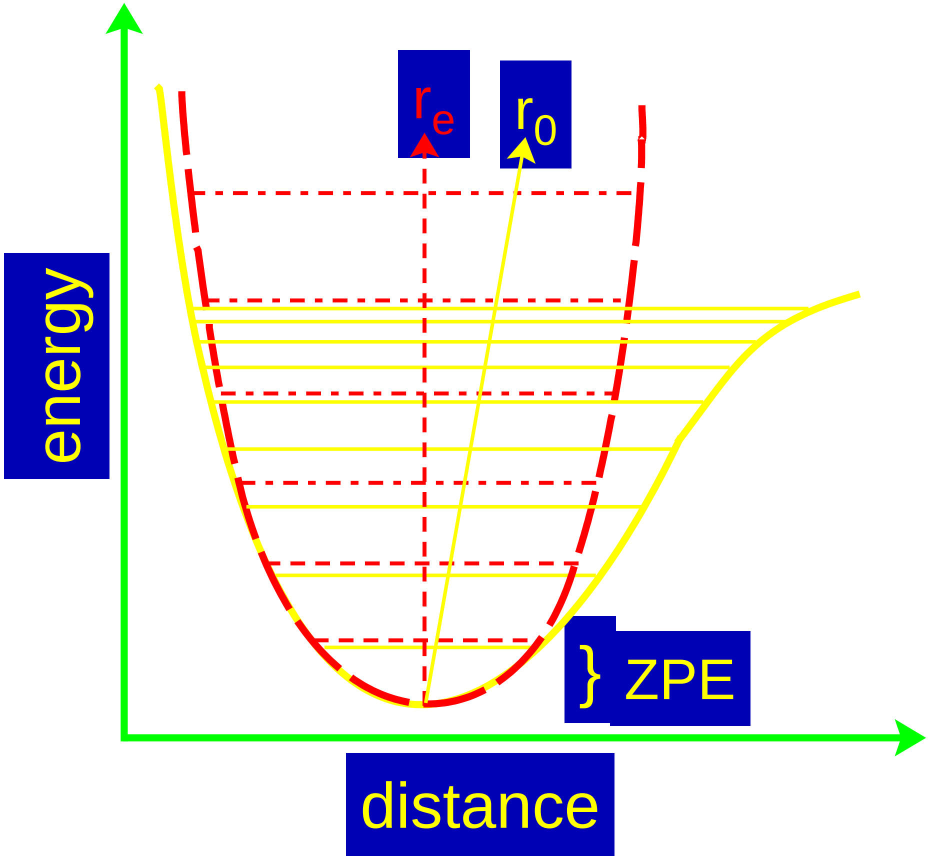

The Z-matrix : Coordinate systems as a reference points

Body fixed: fix (“embed”) axis system in molecule
3 rotational coordinates (2 for linear molecules)

3N–6 vibrational coordinates (or 3N–5)

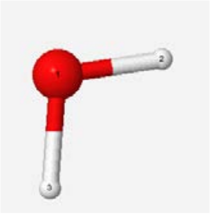

3N - 6 = 3*3 – 6 = **3 coordinates**

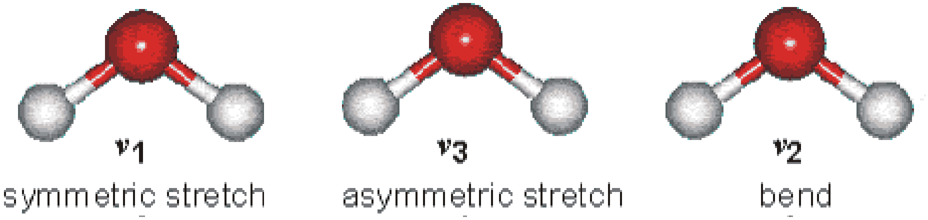

Symmetry of molecular vibrations

Three normal modes of H_2O ***3N-6*** = 3×3 – 6 = 3

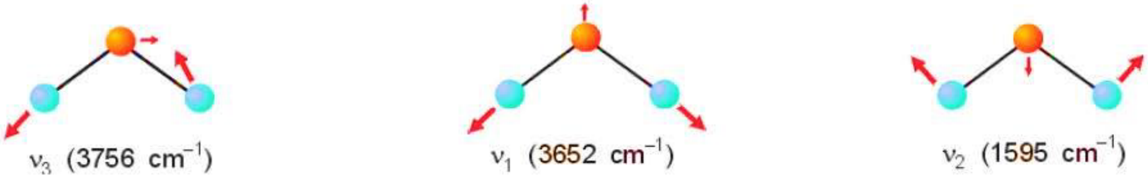

IR-ACTIVE	IR-ACTIVE	IR-ACTIVE
ASYMMETRIC STRETCH	SYMMETRIC STRETCH	BEND

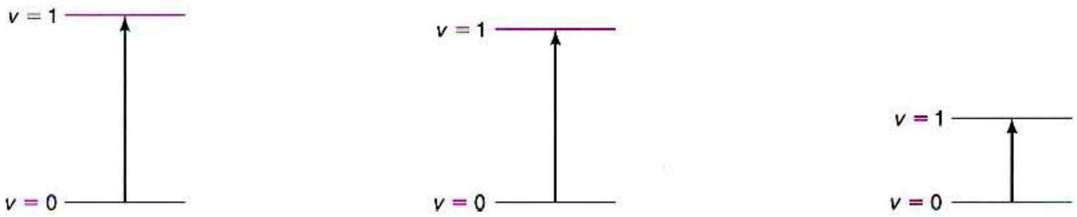

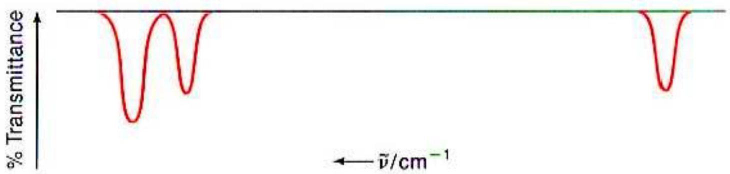

Stretching: Change in inter-atomic distance along bond axis

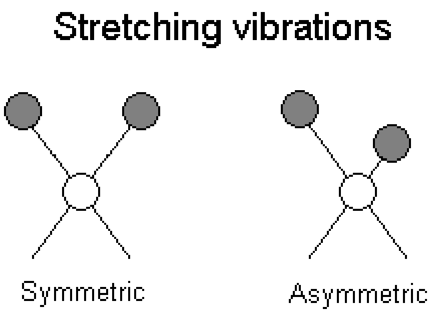

Bending: Change in angle between two bonds. There are four types of bend:

- Rocking
- Scissoring
- Wagging
- Twisting

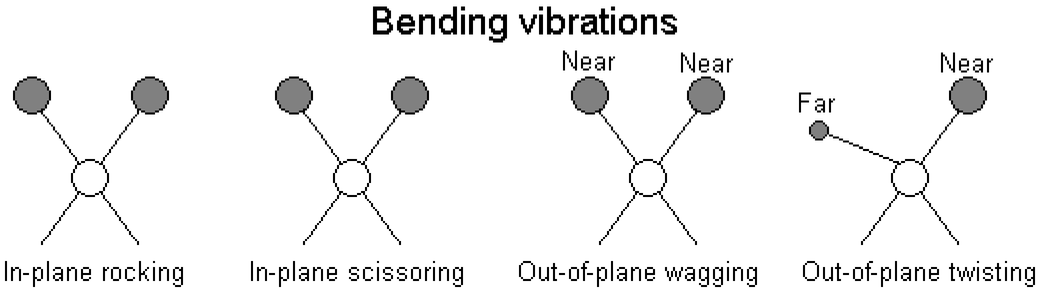

Potential Energy Surfaces

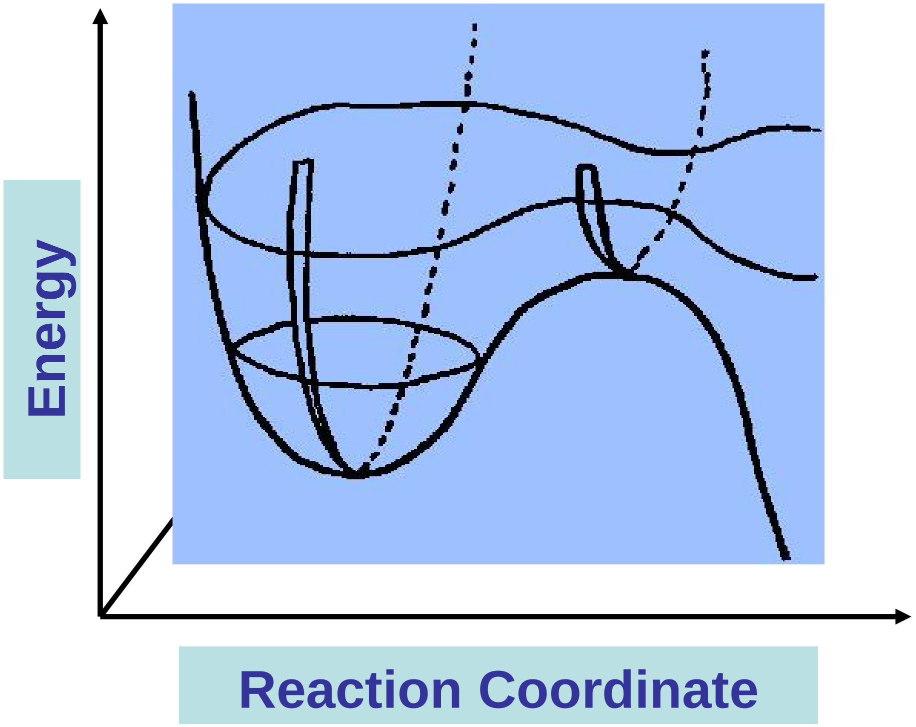

- **Minima**
  - 1st derivative (gradient) = 0
  - 2nd derivative > 0
  - Global vs. local (many)
- **Saddle Point**
  - "Transition state"
  - 2nd derivative:

    < 0 in one direction;

    > 0 in all others.

Calculation of Infrared Spectra

- **Geometry Optimization:** outputs the energy and the geometry of the molecule at the minimum (stationary point) of the PS.

- **Freq:** outputs vibrational frequencies and IR, Raman intensities
  - Frequency calculation must be done at stationary point (optimized geometry).
  - Frequency calculation must use same model and basis set as optimization
  - *Ab initio* systematically overestimates frequencies by ~10% due to electron correlation and anharmonicity

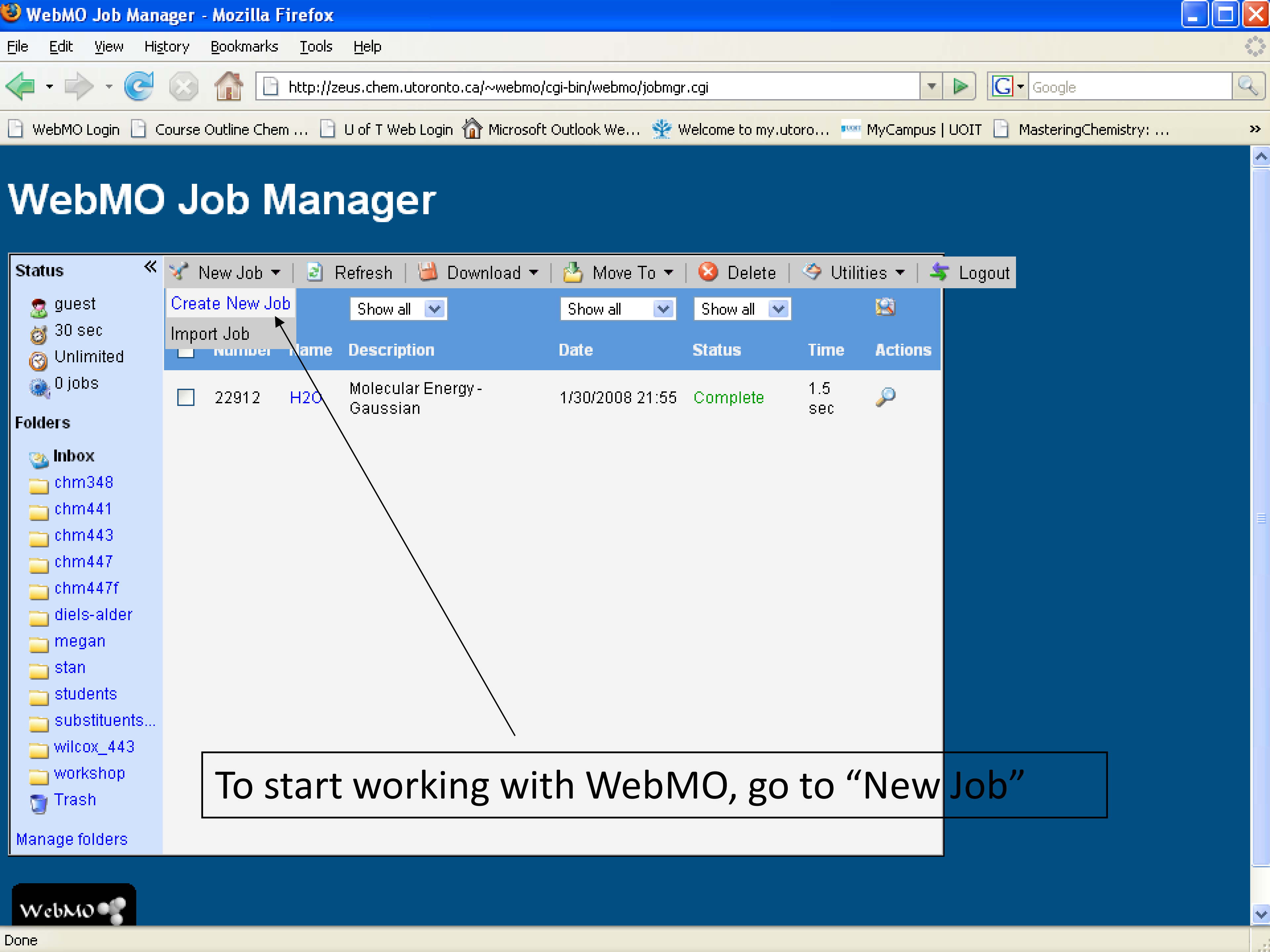

To start working with WebMO, go to “New Job”

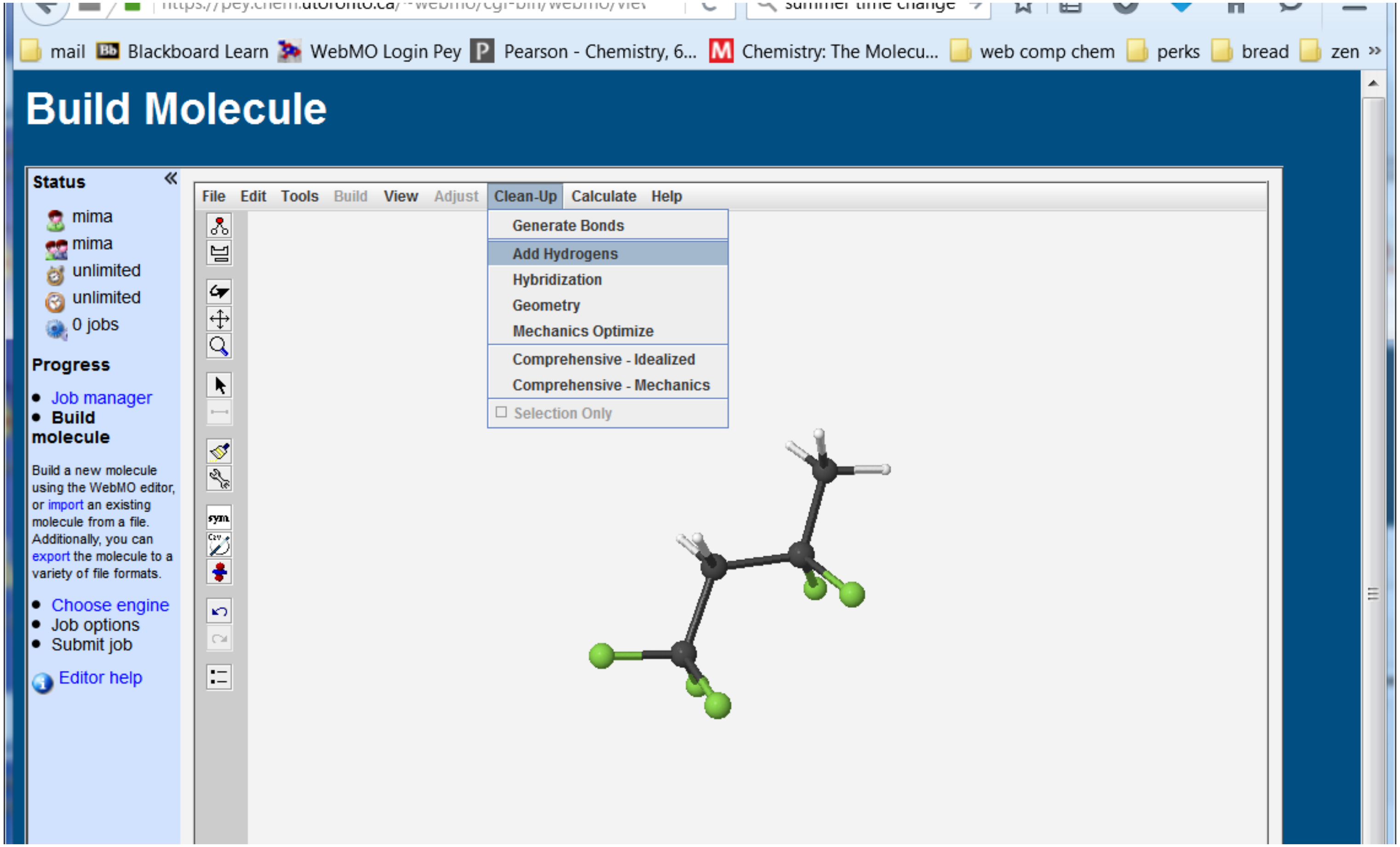
mail
Blackboard Learn
WebMO Login Pey
Pearson - Chemistry, 6...
Chemistry: The Molecu...
web comp chem
perks
bread
zen
Build Molecule
Status
mima
mima
unlimited
unlimited
0 jobs
Progress
Job manager
Build molecule
Build a new molecule using the WebMO editor, or import an existing molecule from a file. Additionally, you can export the molecule to a variety of file formats.
Choose engine
Job options
Submit job
Editor help
File
Edit
Tools
Build
View
Adjust
Clean-Up
Calculate
Help
Generate Bonds
Add Hydrogens
Hybridization
Geometry
Mechanics Optimize
Comprehensive - Idealized
Comprehensive - Mechanics
Selection Only

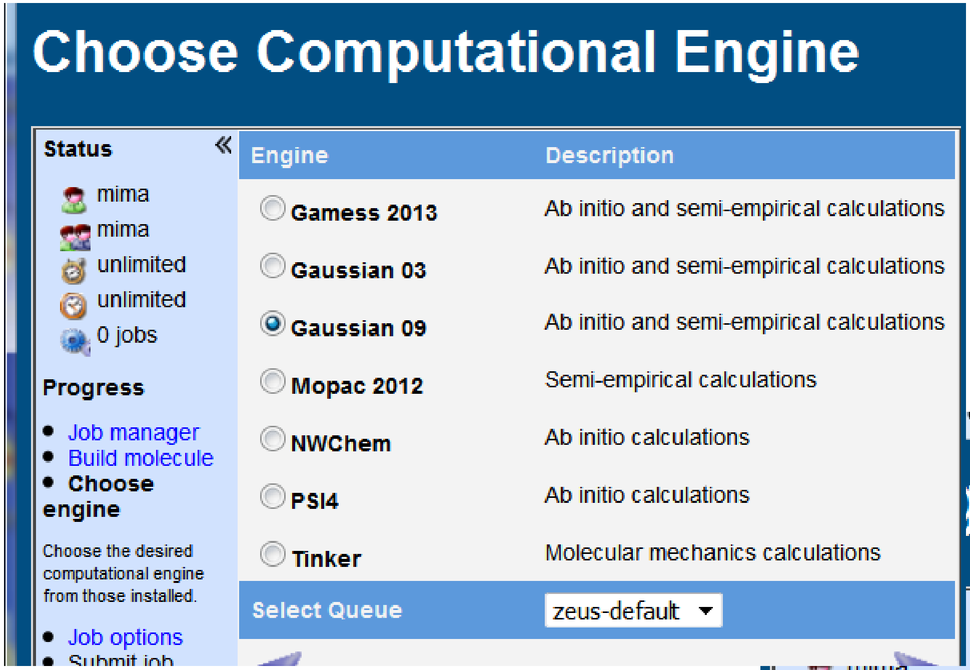
Choose Computational Engine
Status
mima
mima
unlimited
unlimited
0 jobs
Progress
Job manager
Build molecule
Choose engine
Choose the desired computational engine from those installed.
Job options
Engine
Description
Gamess 2013
Ab initio and semi-empirical calculations
Gaussian 03
Ab initio and semi-empirical calculations
Gaussian 09
Ab initio and semi-empirical calculations
Mopac 2012
Semi-empirical calculations
NWChem
Ab initio calculations
PSI4
Ab initio calculations
Tinker
Molecular mechanics calculations
Select Queue
zeus-default

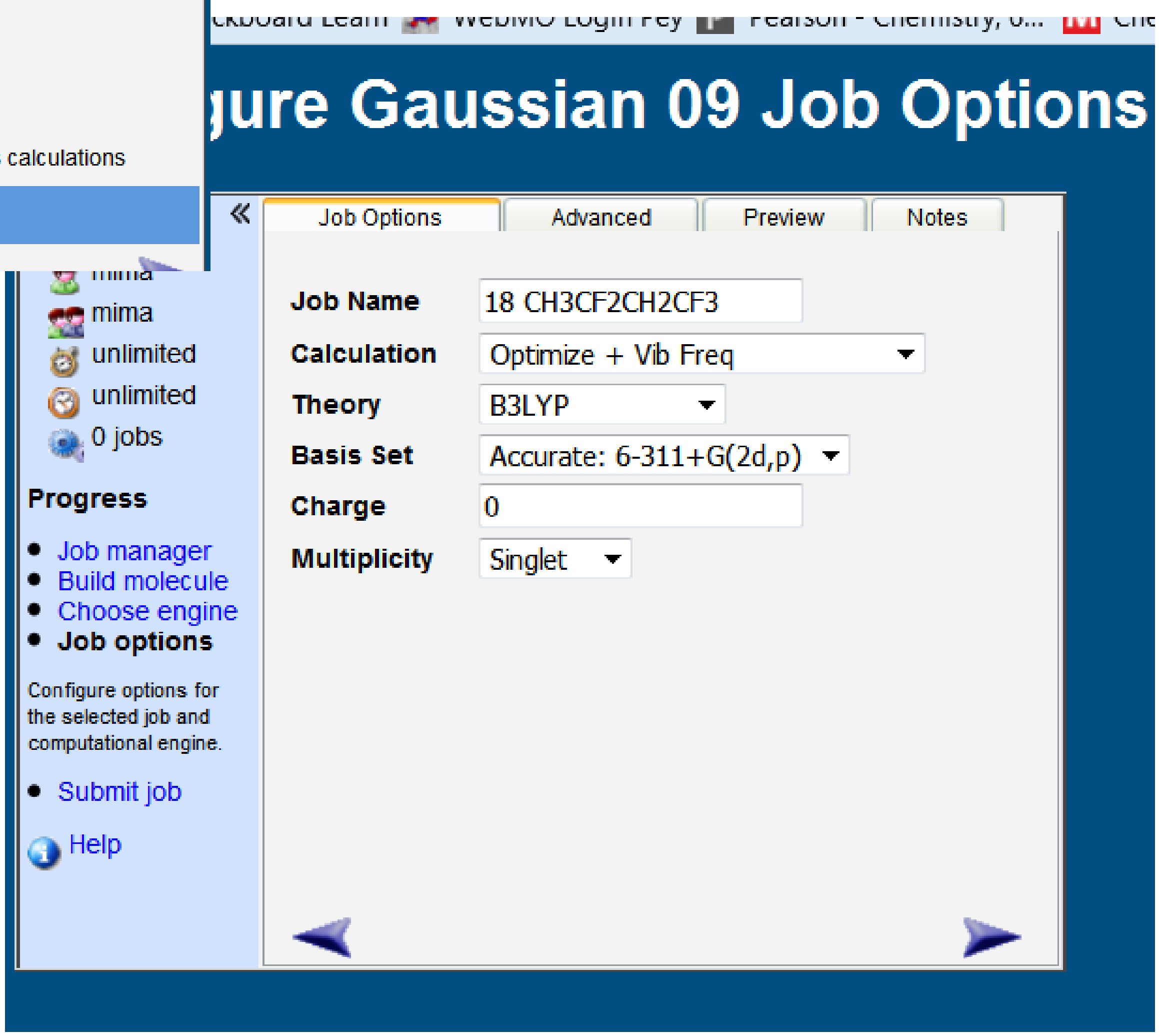
gure Gaussian 09 Job Options
Job Options
Advanced
Preview
Notes
mima
unlimited
unlimited
0 jobs
Progress
Job manager
Build molecule
Choose engine
Job options
Configure options for the selected job and computational engine.
Submit job
Help
Job Name
18 CH3CF2CH2CF3
Calculation
Optimize + Vib Freq
Theory
B3LYP
Basis Set
Accurate: 6-311+G(2d,p)
Charge
0
Multiplicity
Singlet

Evaluating Results

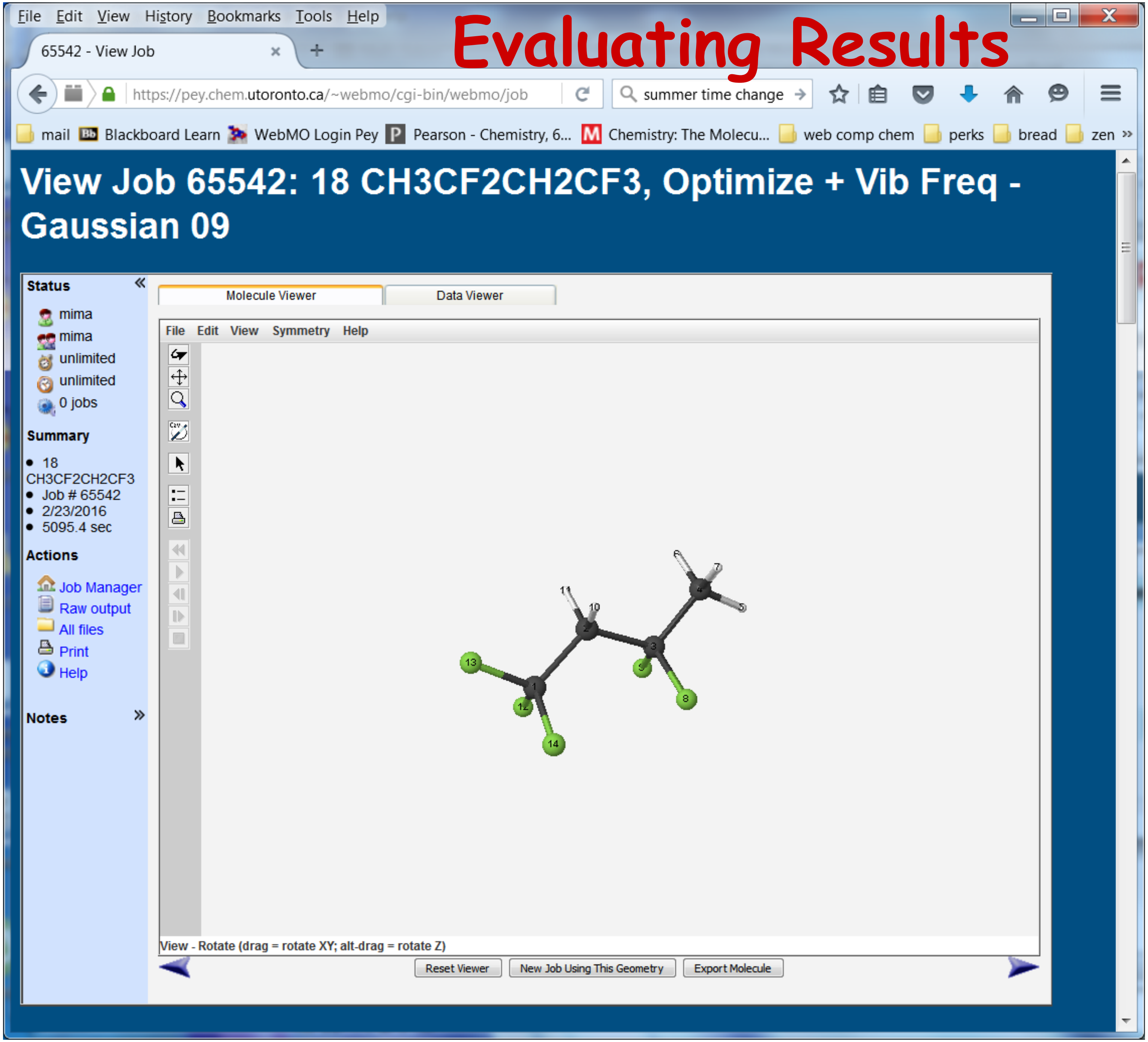

mail Blackboard Learn WebMO Login Pey Pearson - Chemistry, 6... Chemistry: The Molecu... web comp chem

Reset Viewer | New Job Using This Geometry | Export Molecule

Calculated Quantities

Collapse all

Overview

Quantity	Value
Job History	64686,64713,64728
Route	#N B3LYP/6-311+G(2d,p) OPT FREQ Geom=Connectivity
Stoichiometry	$C_4H_5F_5$
Symmetry	C1
Basis	6-311+G(2d,p)
RB3LYP Energy	-654.893977762 Hartree
ZPE	0.092547 Hartree
Conditions	298.150K, 1.00000 atm
Internal Energy	-654.792510 Hartree
Enthalpy	-654.791566 Hartree
Free Energy	-654.837842 Hartree
C_v	31.697 cal/mol-K
Entropy	97.396 cal/mol-K
Dipole Moment	4.0013 Debye
Server	zeus-default (25531)
CPU time	5095.4 sec

Geometry Sequence Energies

https://pey.chem.utoronto.ca/~webmo/cgi-bin/webmo/job

mail | Blackboard Learn | WebMO Login Pey | Pearson - Chemistry, 6... | Che

Vibrational Modes

Mode	Symmetry	Frequency (cm^{-1})	IR (Raman) Intensity	Actions
1	A	4.5218	0.6227	
2	A	115.1972	0.4806	
3	A	154.2851	0.5289	
4	A	216.4681	0.0307	
5	A	280.0662	2.3437	
6	A	293.2215	0.0724	
7	A	308.6678	0.0011	
8	A	375.4458	0.3331	
9	A	424.6979	0.9310	
10	A	496.8705	10.7127	
11	A	516.5428	8.5467	
12	A	529.0150	1.3111	
13	A	578.7251	25.2345	
14	A	681.3468	6.7679	
15	A	817.0277	32.0702	
16	A	843.2054	6.9247	
17	A	889.6052	83.9885	
18	A	947.9302	0.9393	
19	A	961.9056	19.2867	
20	A	1070.4585	26.6021	
21	A	1082.8669	151.6738	
22	A	1202.0680	122.2711	
23	A	1209.9089	338.5527	
24	A	1229.7561	105.8687	
25	A	1285.4588	205.0610	

[illegible]	[illegible]	[illegible]	[illegible]	
20	A	1070.4585	26.6021	
21	A	1082.8669	151.6738	
22	A	1202.0680	122.2711	
23	A	1209.9089	338.5527	
24	A	1229.7561	105.8687	
25	A	1285.4588	205.0610	

26	A	1316.4154	18.6723	
27	A	1410.5190	65.3670	
28	A	1421.9018	75.7511	
29	A	1463.2283	6.0323	
30	A	1486.0023	1.3483	
31	A	1487.1826	4.9161	
32	A	3053.9795	2.4216	
33	A	3080.1437	0.8076	
34	A	3123.6821	0.8606	
35	A	3131.3469	8.0315	
36	A	3133.6298	6.1527	

Frequency Scale Factor 1.0

Normal Mode Amplitude 1.0

Animation Speed 50

IR Spectrum

Peak Width (cm^{-1}) 40

Quote
