# Supplementary material for: Welcome to 310 Environmental Working Group! A Group Project That Places Students in the Role of Consultants Helping Businesses Choose the Most Climate Friendly Fluorinated Gas
Source: J Chem Educ. 2024 Sep 6;101(10):4203–13. doi: 10.1021/acs.jchemed.4c00479 (PMC11465463; doi:10.1021/acs.jchemed.4c00479)
Supplement: Supplementary file 1 — ed4c00479_si_001.zip [file ed4c00479_si_001.zip › Supporting Information/Presentation and Report guidelines and rubrics/310-EWG Report Rubric.docx]

**310-EWG Written Recommendation Rubric**

**Lay Summary**

Mentioned two criteria: 0 1 1.5 2

Climate and persistent products

Made a clear 0 1 0.5 2

Recommendation

Used non-technical 0 0.5 1

language

Under word limit 0 0.5

**Technical Summary**

Discussed and considered 0 1 1.5 2

GWP and other relevant

climate metrics

Identified and discussed 0 1 1.5 2

degradation products

Used the chemical fate model 0 1 1.5 2

output to provide context on the

lasting effects of fugitive emissions

Included required figures 0 0.5 1

Discussed and used figures 0 0.5 1

effectively

Made a clear and evidence- 0 1 1.5 2

based recommendation

Overall clarity 0 1 1.5 2

Under word limit 0 0.5

Total /18
