# Supplementary material for: Welcome to 310 Environmental Working Group! A Group Project That Places Students in the Role of Consultants Helping Businesses Choose the Most Climate Friendly Fluorinated Gas
Source: J Chem Educ. 2024 Sep 6;101(10):4203–13. doi: 10.1021/acs.jchemed.4c00479 (PMC11465463; doi:10.1021/acs.jchemed.4c00479)
Supplement: Supplementary file 1 — ed4c00479_si_001.zip [file ed4c00479_si_001.zip › Supporting Information/Presentation and Report guidelines and rubrics/310-EWG Presentation Guidelines.docx]

| 310 Environmental Working Group |  |
| --- | --- |

Client Presentation

Deliverables

Your team will deliver a presentation to the CEO of your assigned client (played by Prof. D’eon) and their board of directors (played by the students in the other groups present). When acting as the board of directors you will be ***awarded bonus marks for asking questions***, so be sure to pay attention and participate.

Your presentation needs to be persuasive and present a cohesive and fact-based argument around your recommendation. You can assume the board of directors has some scientific background, and so you can present your material at the level of a student who has completed CHM 310. The timing of your presentation should be 2-3 minutes per group member to a maximum of 15-minutes, with an additional 5-minutes for questions. All group members must participate. As mentioned above, your task is to make a final recommendation to your client as to how they can incorporate your environmental assessment into future buying decisions. You should refer to the results from the four assignments completed during the semester as data you generated in your role as an industrial consultant.

When presenting your recommendation be sure to touch on **ALL** of these discussion points:

- For each chemical provide the rate constant for reaction with OH (*k_OH_*), the radiative efficiency (RE), and global warming potential (GWP)
  - Note: Radiative efficiency (RE) calculations using data from Gaussian will vary. As a group you will need to come to a consensus with respect to what value is best, and make sure this value is used to calculate the presented GWP. Please include a brief description of how you came to a consensus in your presentation.
- For each chemical include an environmental fate diagram similar to that submitted for Assignment 3, and use this figure to describe the fate of the chemicals with emphasis on the generation of persistent products
- For each chemical include the results of the chemical fate model from Assignment 4, and explain what it tells you about the long-term implications of the chemical on the environment
