# Supplementary material for: Welcome to 310 Environmental Working Group! A Group Project That Places Students in the Role of Consultants Helping Businesses Choose the Most Climate Friendly Fluorinated Gas
Source: J Chem Educ. 2024 Sep 6;101(10):4203–13. doi: 10.1021/acs.jchemed.4c00479 (PMC11465463; doi:10.1021/acs.jchemed.4c00479)
Supplement: Supplementary file 1 — ed4c00479_si_001.zip [file ed4c00479_si_001.zip › Supporting Information/Presentation and Report guidelines and rubrics/310-EWG Report Guidelines.docx]

| 310 Environmental Working Group |  |
| --- | --- |

Written Recommendation

Deliverables

Your written assignment is a follow-up document provided to your client to summarize the recommendations presented in the client meeting. The assessment must contain a lay summary and a technical summary. It is important to note that although this is a follow-up document to the presentation it is not a problem to change your recommendation between this document and the presentation, either because of a change in perspective or because you disagree with some of your group members. Unlike the presentation, ***this is an entirely individual assignment*.**

Lay Summary (300 words max, display word count below title)

The purpose of the lay summary is to present your recommendation to a broad audience using non-technical terminology. It will be used by the client to disseminate the results of your evaluation to a wide audience both inside and outside of their organization to justify buying decisions. Although it will come at the beginning of the written assignment, the lay summary is really a non-technical summary of the technical summary and so it is likely best written last.

Technical Summary (700 words max, display word count below title)

The technical summary should present your recommendation with specific reference to the potential of chemicals in question to produce long-lived degradation products and their effect on climate. The technical summary **must contain the figure from Assignment 3 on environmental fate and the output from the chemical fate models from Assignment 4** and may contain up to two more tables or figures if they are useful in illustrating your discussion points.
