# Supplementary material for: Welcome to 310 Environmental Working Group! A Group Project That Places Students in the Role of Consultants Helping Businesses Choose the Most Climate Friendly Fluorinated Gas
Source: J Chem Educ. 2024 Sep 6;101(10):4203–13. doi: 10.1021/acs.jchemed.4c00479 (PMC11465463; doi:10.1021/acs.jchemed.4c00479)
Supplement: Supplementary file 1 — ed4c00479_si_001.zip [file ed4c00479_si_001.zip › Supporting Information/Presentation and Report guidelines and rubrics/310-EWG Presentation Rubric (used for group and peer evaluation).docx]

**310-EWG Group Presentation Evaluation**

**Total: /20**

| **Grp #: _______________** | **Chemical 1** | **Chemical 2** |
| --- | --- | --- |
| **Structure** |  |  |
| **Lifetime or rate constant** |  |  |
| **Radiative Efficiency** |  |  |
| **GWP** |  |  |
| **Persistent Products? (Y/N)** |  |  |

**/5**

***Discussion of Climate***

Did they discuss the climate implications of both compounds?

Did they discuss the climate implications of potential oxidation products that may have a significant atmospheric lifetime?

*Comments:*

**/5**

***Discussion of Environmental Fate***

Is their oxidation scheme correct? Do they identify long-lived products? Do they discuss the fate and implications of any long-lived oxidation products?

*Comments:*

**/5**

***Final Recommendation***

Did they clearly give a final recommendation? Was their recommendation consistent with evidence they presented?

*Comments:*

**/5**

***Overall Clarity***

Were their slides clear and concise? Was it easy to follow the reasoning behind their recommendation?

*Comments:*

Please include additional comments on the reverse side of the paper.
