# Supplementary material for: Welcome to 310 Environmental Working Group! A Group Project That Places Students in the Role of Consultants Helping Businesses Choose the Most Climate Friendly Fluorinated Gas
Source: J Chem Educ. 2024 Sep 6;101(10):4203–13. doi: 10.1021/acs.jchemed.4c00479 (PMC11465463; doi:10.1021/acs.jchemed.4c00479)
Supplement: Supplementary file 1 — ed4c00479_si_001.zip [file ed4c00479_si_001.zip › Supporting Information/Assignment 4/310 EWG Assignment 4 Fall 2018.docx]

| 310 Environmental Working Group |  |
| --- | --- |

Assignment 4

Deliverables

**Chemical Partitioning**

Once you have identified relevant degradation products you will need to characterize their partitioning. The easiest way to quickly access a given chemical’s partition behavior is to use the values predicted by ACS/Labs, which are provided on SciFinder. You can register and access SciFinder through the chemistry library using this link: <https://www.chem.utoronto.ca/facilities/chemlib/>.

1. Once on the SciFinder site choose *Substance Identifier* from the list of choices on the left-hand side
2. In the available text box use a chemical name, molecular formula, or CAS registry number to find the compound of interest
3. To obtain the substance details click on the appropriate structure
4. Choose the *Predicted Properties* link below
5. Choose the *Chemical* tab
6. There you will find relevant chemical properties, however they may have different names than you are used to. Here are some properties that are typically provided:

K_OC_ – The partition co-efficient between water and the organic content in soil

logD – log(K_OW_) defined at the given pH (D stands for distribution)

logP – log(K_OW_) at pH 7 (P stands for partitioning)

Mass Solubility – water solubility expressed in units of g/L and defined at a given pH

Molar Solubility – water solubility expressed in units of mol/L and defined at a given pH

pKa – only relevant if the compound has acid or base functionality

Vapor Pressure – Partial pressure of the compound above its pure liquid form, typically in units of torr

All of the properties from SciFinder are predicted using ACD Labs software. Comptox is a more sophisticated platform hosted by the U.S. Environmental Protection Agency that includes experimental data. To access Comptox click on this link: <https://comptox.epa.gov/dashboard> and search for your chemicals using their CAS number or other identifier. If your compound is found click ‘Properties’ on the left-hand menu bar to see the data available. The list of data that is shown is an average of the values available. To look at the individual values click on the property (e.g. Log P, vapour pressure…) and the data with its source is provided. If there is an experimental value provided use that one, if they are all predicted use the OPERA value is available as it is the newest prediction model and much better at predicting values for fluorinated chemicals. ***If your compound is found on Comptox use the best value there to answer the questions below. If your compound is not found on Fate Pointer use the predicted values from SciFinder to answer the questions below.***

**Questions**

For this assignment all solutions can be submitted online, however a hard copy of the calculations can be handed in class in to receive partial credit where relevant. The online submission is done through Quercus by completing the word document entitled “310 EWG Assignment 4 Fall 2018 Report Sheet.docx”. Your submitted report sheet should be renamed as “Surname_FirstName_310EWG_Assign3.docx”.

1. Draw the structure of your assigned chemicals.
2. For your two assigned chemicals provide the log(D) or log(P), molar solubility at pH 7 (C_w_), and vapour pressure (VP). Be sure to include units.
3. Indicate whether the values in Question 2 were calculated using data from Comptox or SciFinder.
4. One method of understanding and describing chemical partitioning is to use a simple thermodynamic chemical space model discussed in class and described in the paper *Displaying Multimedia Environmental Partitioning by Triangular Diagrams* by Sum Chi Lee and Donald Mackay (Environ. Toxicol. Chem. 1995, 14, 1839) that determines the mole percentage (the paper uses mass in grams, but I would like you to use moles) present in each of the three environmental phases (air, water, and organic) when 100 moles of a compound are allowed to come to equilibrium in a given scenario. By calculating the number of moles in each phase you are creating a simple level 1 model that represents a static environment at equilibrium. This model is limited in its scope but it is a simple way to provide insight into a chemical’s partitioning behavior in a variety of scenarios.

Use the values from Question 2 to calculate a K_AW_, K_OW_, K_OA_ for both of your chemicals. When performing this calculation remember that the partition coefficients are equilibrium constants for the movement between phases where the concentrations are defined in terms of molarity. (**2 marks**)

1. For the following three scenarios calculate the molar percentage in the three environmental compartments (*m*_air_, *m*_water_, *m*_organic_). (Note: these calculations are much easier if done on Excel) (**3 marks**)
   1. Dry summer day (V_AIR_ = 10^8^ m^3^, V_WATER_ = 10^3^ m^3^, V_ORGANIC_ = 1 m^3^)
   2. Thunderstorm (V_AIR_ = 10^6^ m^3^, V_WATER_ = 10^5^ m^3^, V_ORGANIC_ = 1 m^3^)
   3. Inside the lung (V_AIR_ = 10 m^3^, V_WATER_ = 1 m^3^, V_ORGANIC_ = 1 m^3^)
2. Using differences between scenarios in Question 5(a) and 5(b) describe why even relatively volatile compounds can be partially *washed out* of the atmosphere during a heavy rainstorm. (**2 marks**)
3. Are the compounds that are *washed out* during the thunderstorm permanently removed from the atmosphere? Why or why not? (**2 marks**)
4. There are many hydrofluorocarbons (HFCs) used as inhaled anesthetics (i.e. enflurane and desflurane). Hydrofluorocarbons are very volatile compounds, how it is possible for them to enter our lungs during administration? (**2 marks**)
5. In this assignment you will also model the environmental fate of each of your HFCs using a level IV model written in visual basic in Excel. Full details on how you will develop and run your model is provided in the pdf file: *CHM310 Model Instructions*. Follow the instructions in that document to answer the questions below.

You can run this model on your personal computer, however it requires the 64-bit version of Excel and the 32-bit is often the default (more information on this can be found here: <https://support.microsoft.com/en-ca/help/15056/windows-7-32-64-bit-faq>). When running the model Excel will appear frozen and non-responsive, but it’s really just busy and the calculations will complete in 5-10 minutes. To overcome these issues I am offering the times below when 12 students at a time can come to the computer lab in LM 121 to run their models there. **A spot in the computer lab needs to booked using the groups function on Quercus.** Please note that these times are for you to run the model, **you must come to the computer lab with the model already coded**. The times available are

**Monday Nov** 12:00-12:45 **Tuesday Nov 13** 12:00-12:45 **Wed Nov 14** 10:00-10:45

12:45-1:30 12:45-1:30 10:45-11:30

11:30-12:15

- 1. In the table provided for question 9(a) report the relevant rate constants and log K_AW_ for each chemical. (Note: For rate constants that were no calculated as part of the 310-EWG assignments use the data provided with Assignment 3, also remember the rate constants must be in units of min^-1^ or cm^3^ molecule^-1^ min^-1^ for reactions with hydroxyl radicals) (**1 mark**)
  2. Create and print a summary of your mass balance equations from your plain text program. (**2 marks**)
  3. Format and print your fate graphs generated by the model. Formatting may include removing some of the series that are not relevant, changing font sizes etc. (**2 marks**)
  4. Answer the following questions using the plots for both chemicals generated by the model in part (c).
     1. Did any of the chemicals in your model achieve steady-state? Explain. (**2 marks**)
     2. Did the emission of your chemical for 50 years result in the production of persistent organic pollutants? Explain. (**2 marks**)
