# Supplementary material for: Welcome to 310 Environmental Working Group! A Group Project That Places Students in the Role of Consultants Helping Businesses Choose the Most Climate Friendly Fluorinated Gas
Source: J Chem Educ. 2024 Sep 6;101(10):4203–13. doi: 10.1021/acs.jchemed.4c00479 (PMC11465463; doi:10.1021/acs.jchemed.4c00479)
Supplement: Supplementary file 1 — ed4c00479_si_001.zip [file ed4c00479_si_001.zip › Supporting Information/Assignment 4/CHM310_Model_Instructions_Excel_VBA.pptx]

## Slide 1
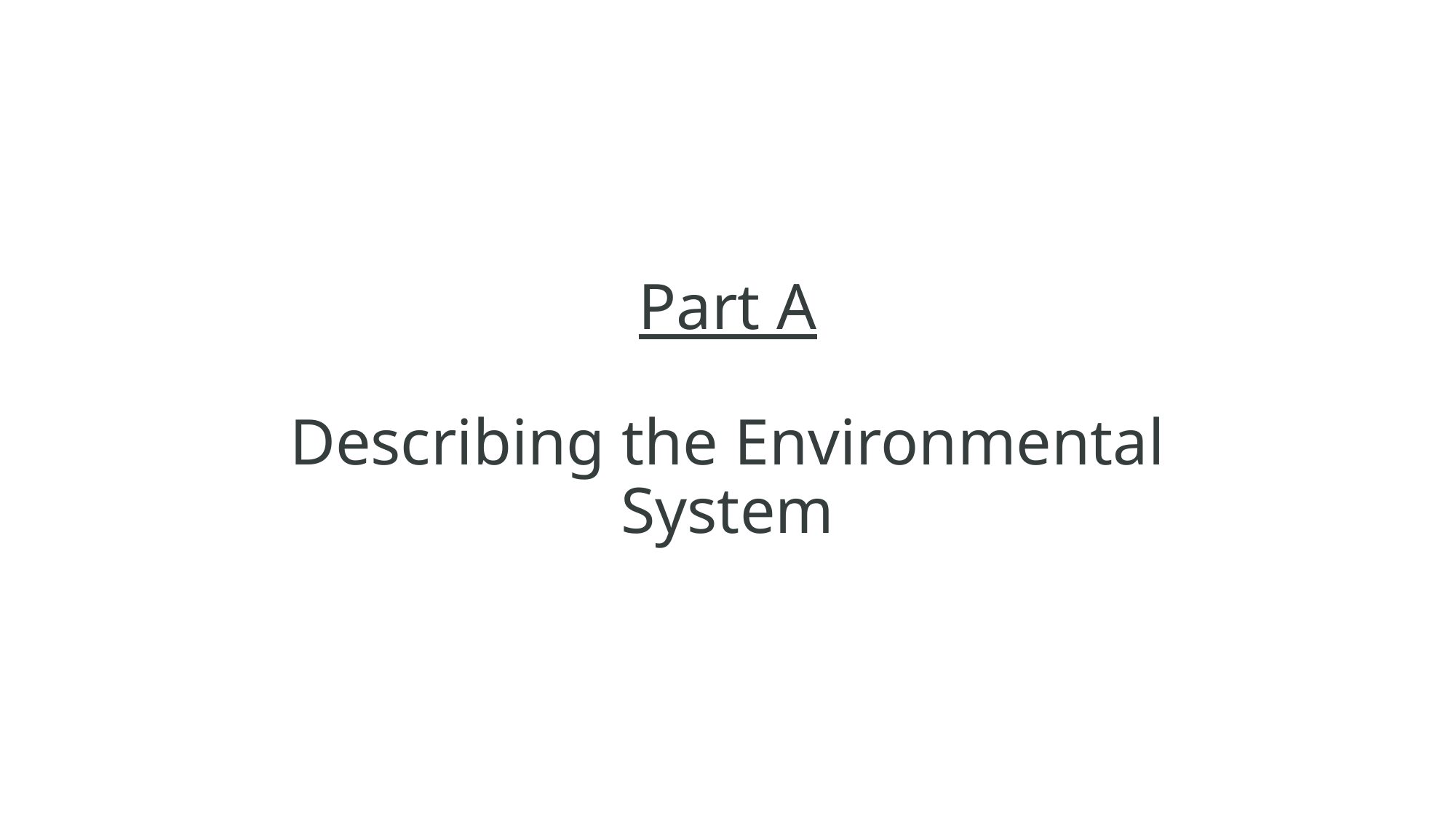

# Part A Describing the Environmental System

## Slide 2
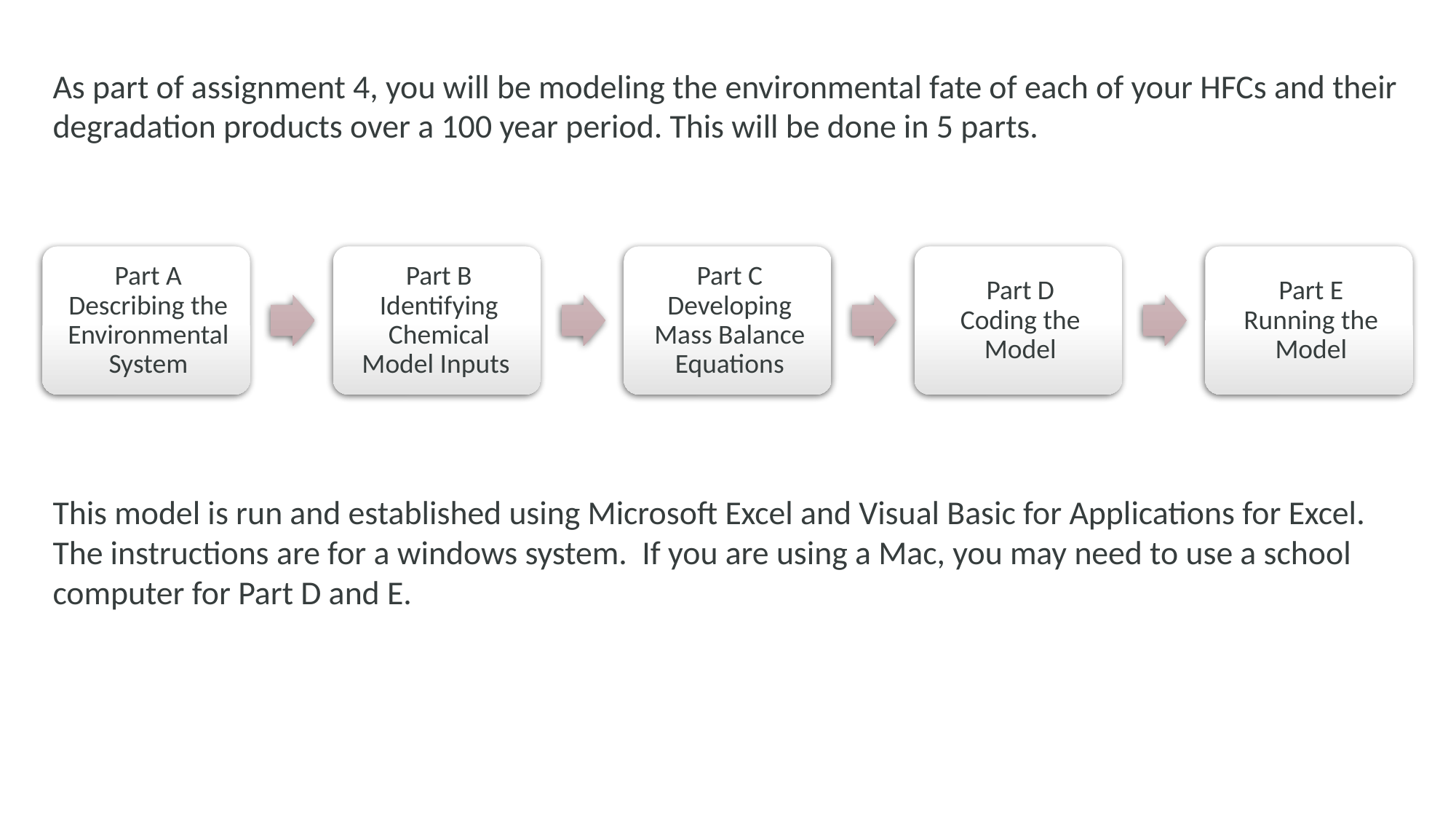

As part of assignment 4, you will be modeling the environmental fate of each of your HFCs and their degradation products over a 100 year period. This will be done in 5 parts.
This model is run and established using Microsoft Excel and Visual Basic for Applications for Excel. The instructions are for a windows system. If you are using a Mac, you may need to use a school computer for Part D and E.

## Slide 3
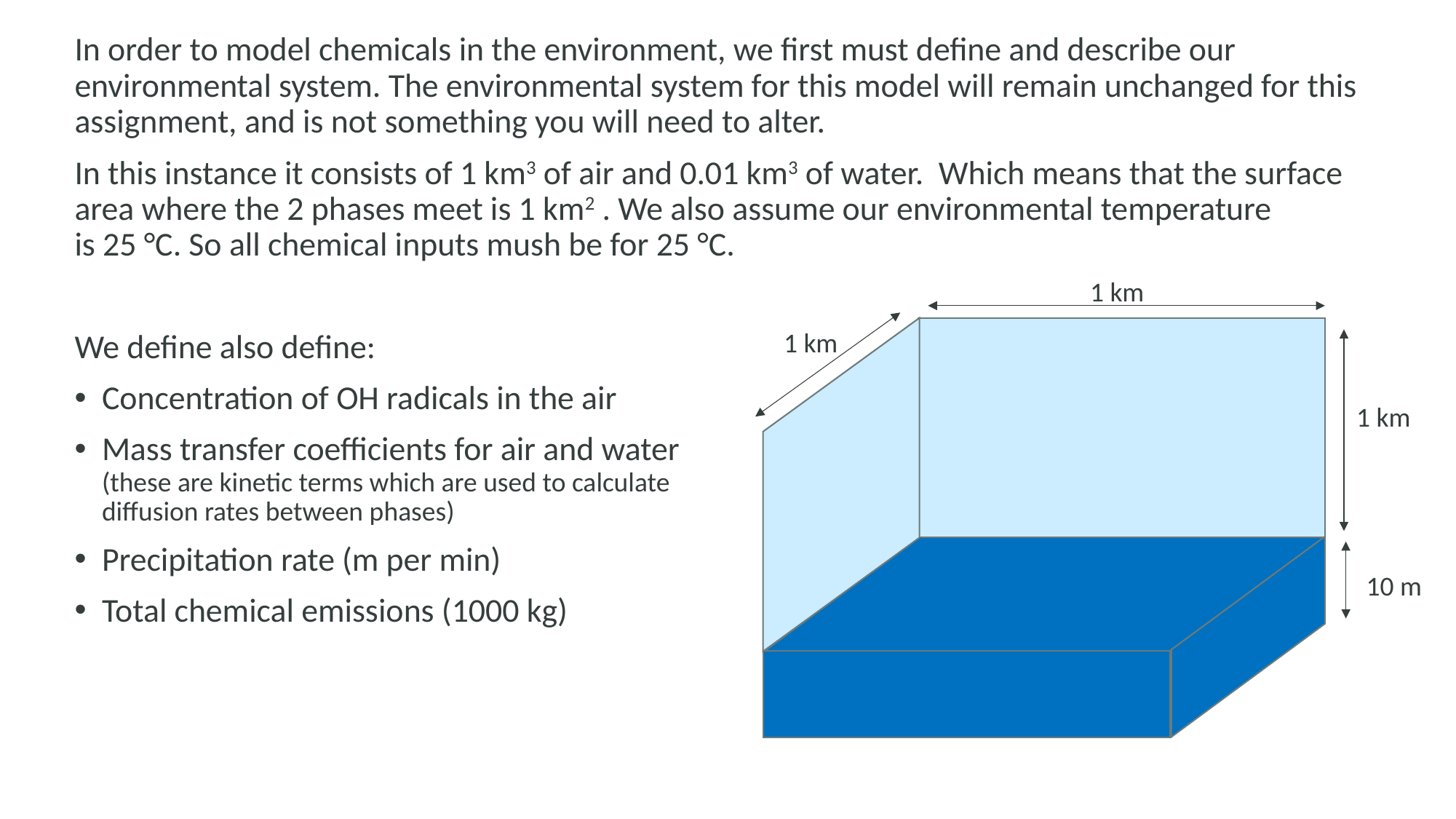

In order to model chemicals in the environment, we first must define and describe our environmental system. The environmental system for this model will remain unchanged for this assignment, and is not something you will need to alter.
In this instance it consists of 1 km3 of air and 0.01 km3 of water. Which means that the surface area where the 2 phases meet is 1 km2 . We also assume our environmental temperature
is 25 °C. So all chemical inputs mush be for 25 °C.
We define also define:
Concentration of OH radicals in the air
Mass transfer coefficients for air and water
(these are kinetic terms which are used to calculate
diffusion rates between phases)
Precipitation rate (m per min)
Total chemical emissions (1000 kg)
1 km
1 km
1 km
10 m

## Slide 4
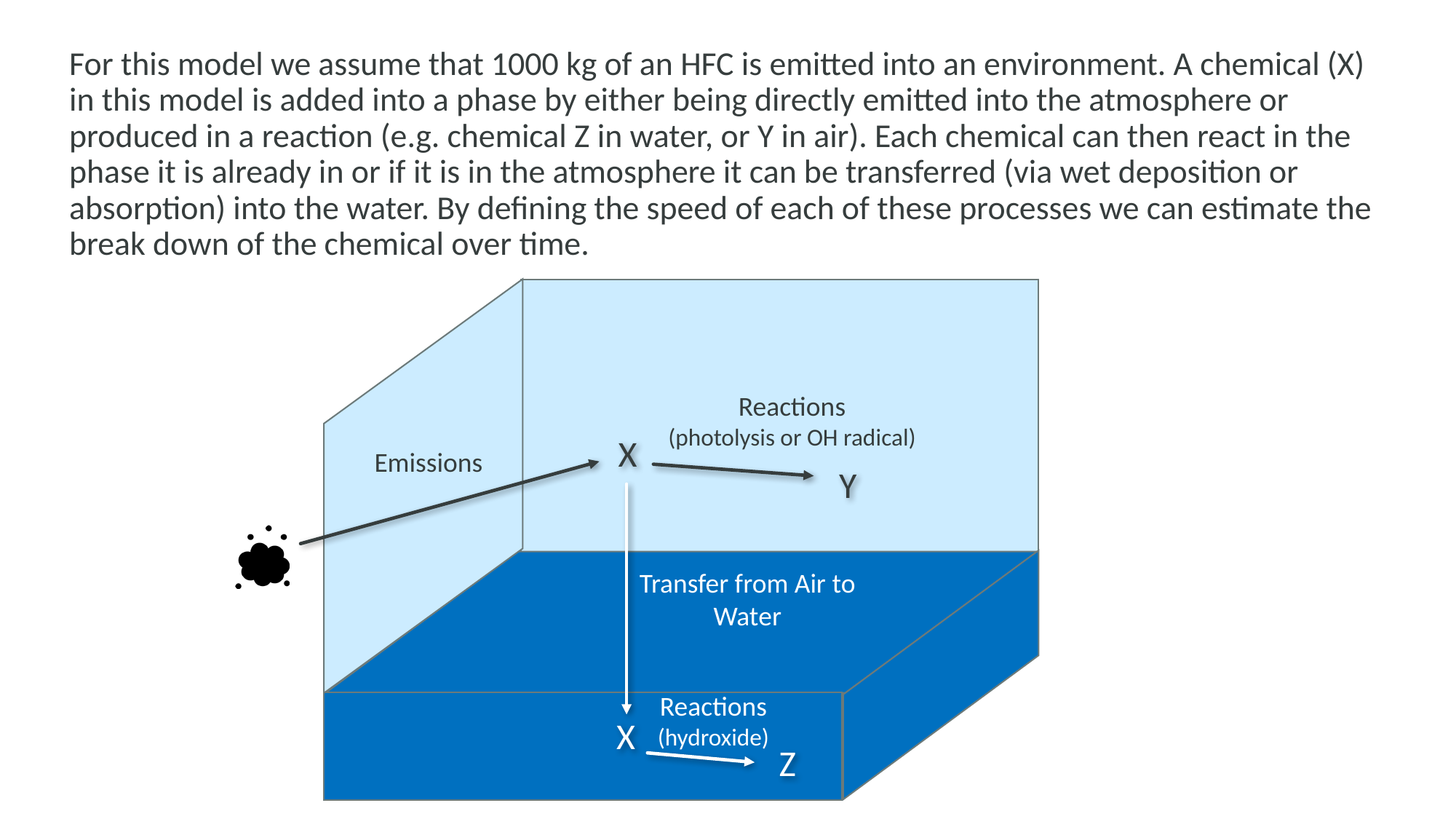

For this model we assume that 1000 kg of an HFC is emitted into an environment. A chemical (X) in this model is added into a phase by either being directly emitted into the atmosphere or produced in a reaction (e.g. chemical Z in water, or Y in air). Each chemical can then react in the phase it is already in or if it is in the atmosphere it can be transferred (via wet deposition or absorption) into the water. By defining the speed of each of these processes we can estimate the break down of the chemical over time.
Reactions
(photolysis or OH radical)
X
Emissions
Y
Transfer from Air to Water
Reactions (hydroxide)
X
Z

## Slide 5
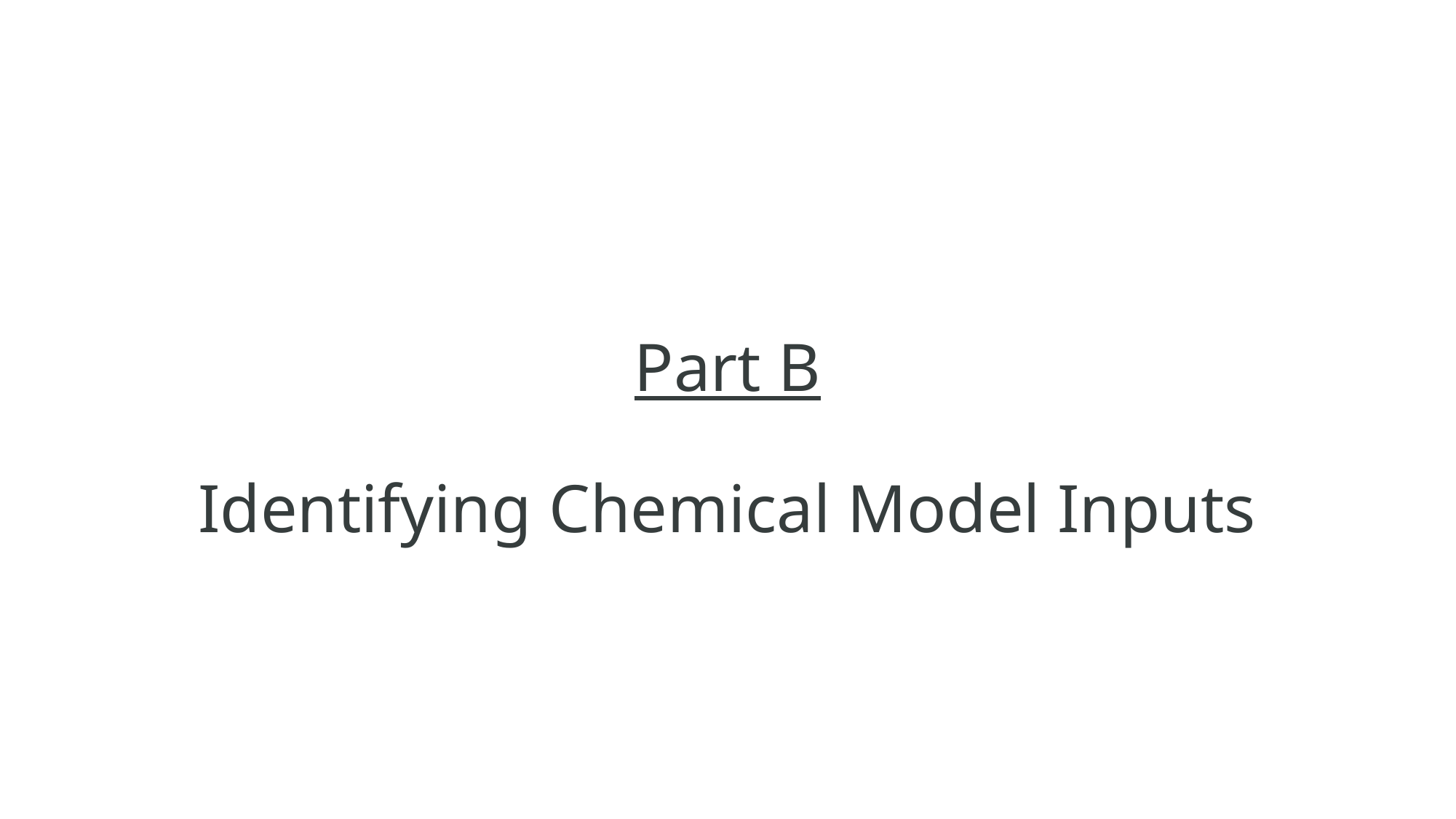

# Part B Identifying Chemical Model Inputs

## Slide 6
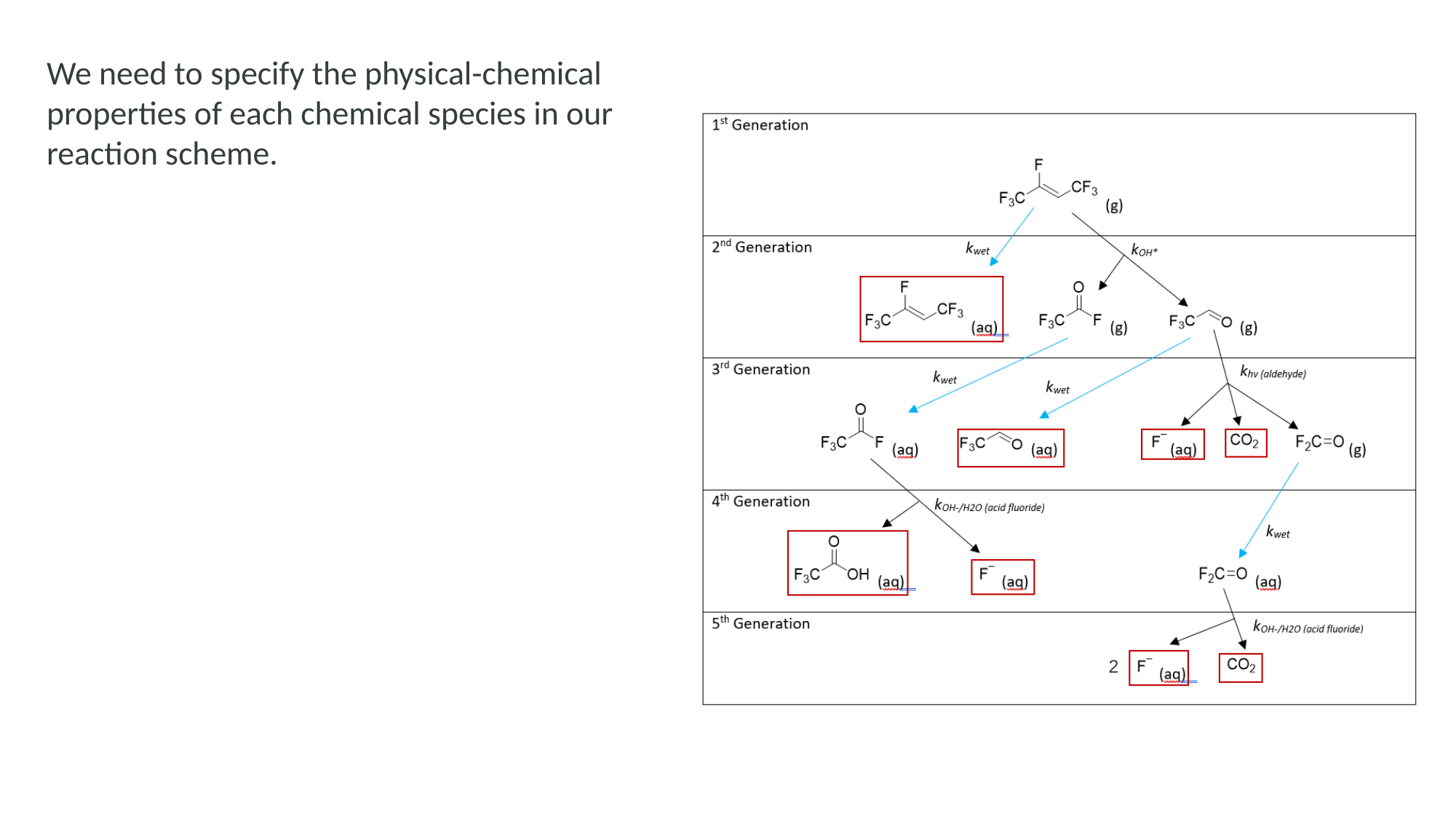

We need to specify the physical-chemical properties of each chemical species in our reaction scheme.
2

## Slide 7
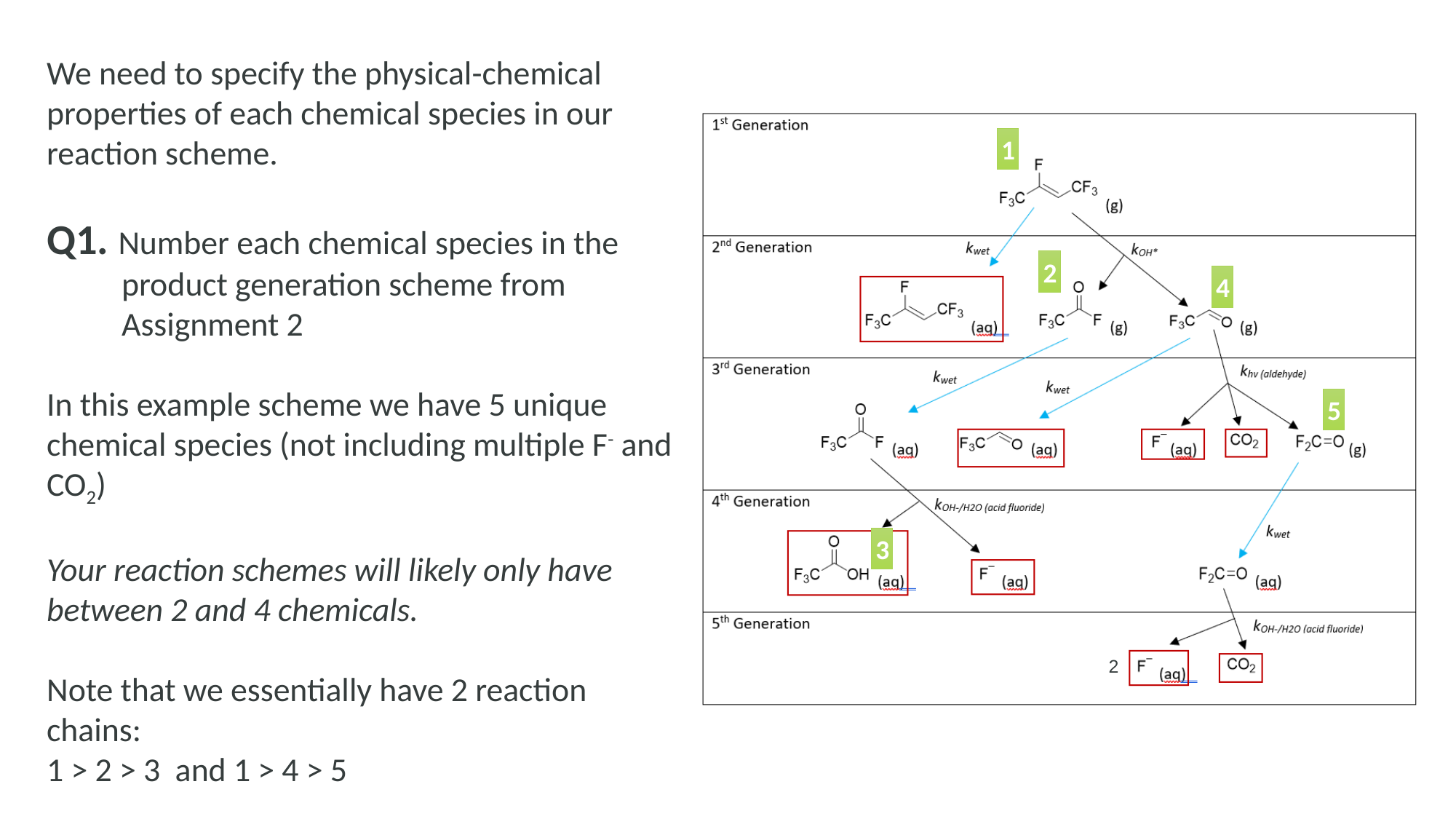

We need to specify the physical-chemical properties of each chemical species in our reaction scheme.
Q1. Number each chemical species in the product generation scheme from Assignment 2
In this example scheme we have 5 unique chemical species (not including multiple F- and CO2)
Your reaction schemes will likely only have between 2 and 4 chemicals.
Note that we essentially have 2 reaction chains:
1 > 2 > 3 and 1 > 4 > 5
1
2
4
5
3
2

## Slide 8
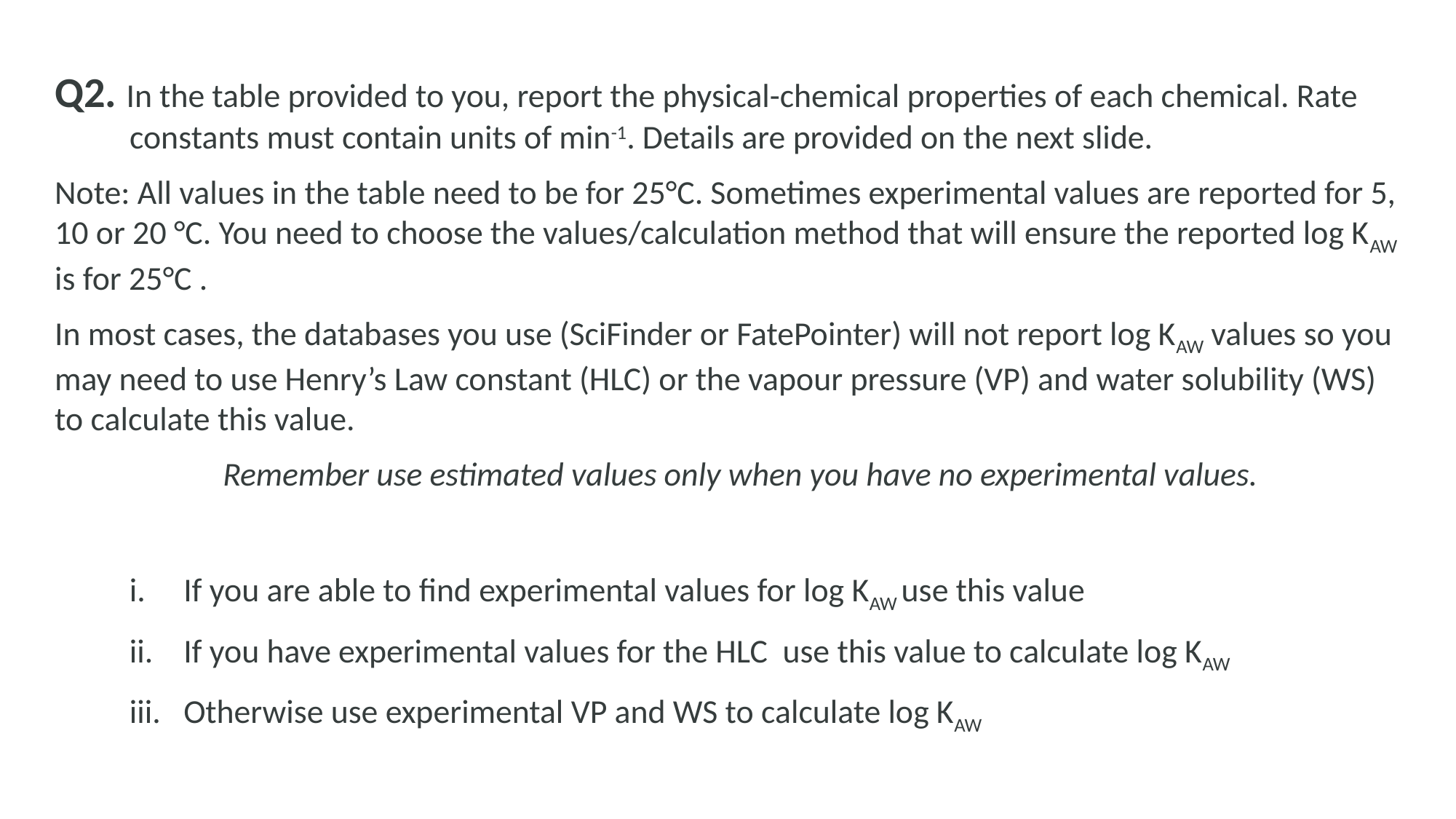

Q2. In the table provided to you, report the physical-chemical properties of each chemical. Rate constants must contain units of min-1. Details are provided on the next slide.
Note: All values in the table need to be for 25°C. Sometimes experimental values are reported for 5, 10 or 20 °C. You need to choose the values/calculation method that will ensure the reported log KAW is for 25°C .
In most cases, the databases you use (SciFinder or FatePointer) will not report log KAW values so you may need to use Henry’s Law constant (HLC) or the vapour pressure (VP) and water solubility (WS) to calculate this value.
Remember use estimated values only when you have no experimental values.
If you are able to find experimental values for log KAW use this value
If you have experimental values for the HLC use this value to calculate log KAW
Otherwise use experimental VP and WS to calculate log KAW

## Slide 9
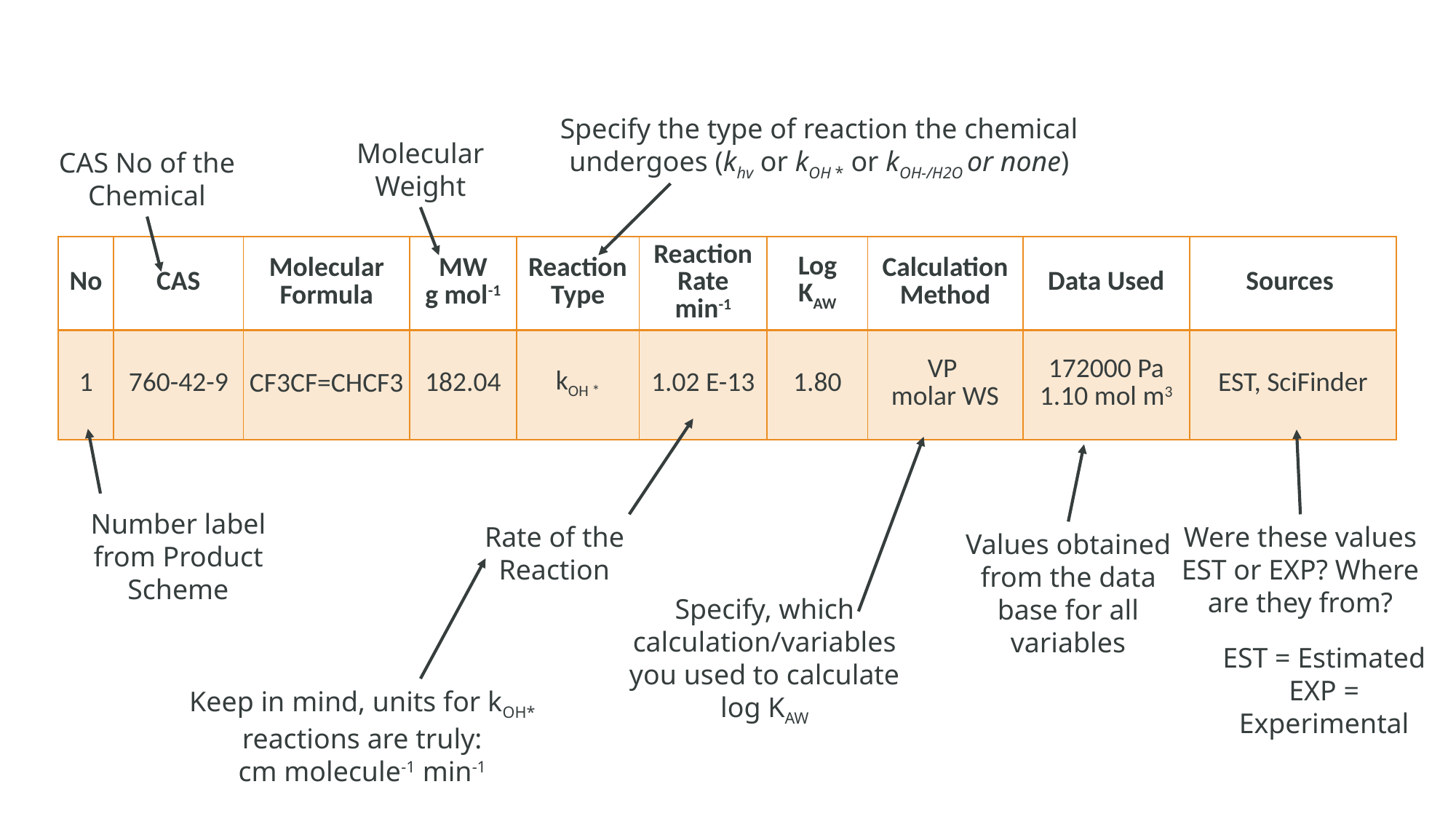

Specify the type of reaction the chemical undergoes (khv or kOH * or kOH-/H2O or none)
Molecular Weight
CAS No of the Chemical
| No | CAS | Molecular Formula | MW g mol-1 | Reaction Type | Reaction Rate min-1 | Log KAW | Calculation Method | Data Used | Sources |
| --- | --- | --- | --- | --- | --- | --- | --- | --- | --- |
| 1 | 760-42-9 | CF3CF=CHCF3 | 182.04 | kOH \* | 1.02 E-13 | 1.80 | VP molar WS | 172000 Pa 1.10 mol m3 | EST, SciFinder |
Number label from Product Scheme
Rate of the Reaction
Were these values EST or EXP? Where are they from?
Values obtained from the data base for all variables
Specify, which calculation/variables you used to calculate log KAW
EST = Estimated
EXP = Experimental
Keep in mind, units for kOH* reactions are truly:
cm molecule-1 min-1

## Slide 10
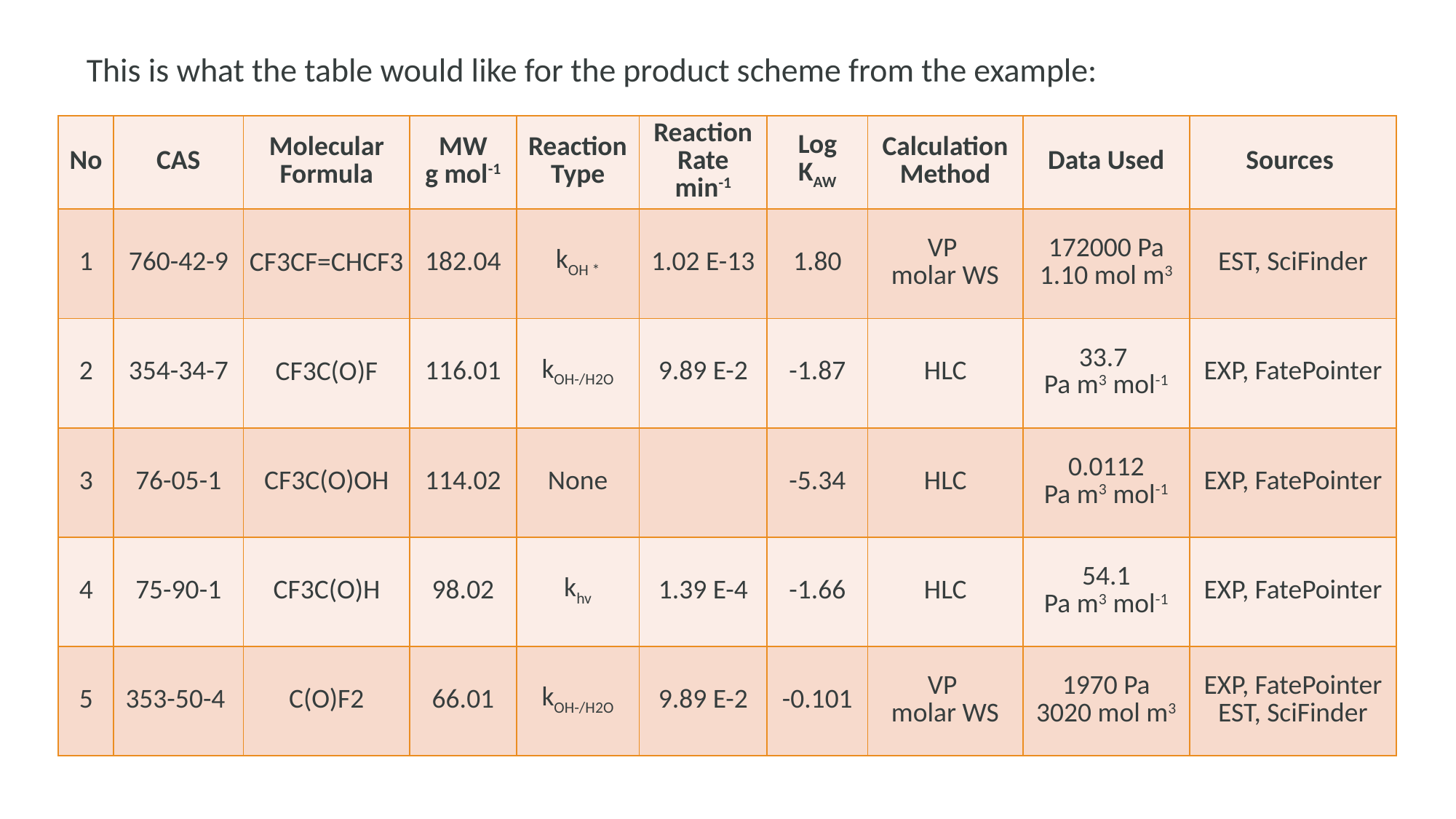

This is what the table would like for the product scheme from the example:
| No | CAS | Molecular Formula | MW g mol-1 | Reaction Type | Reaction Rate min-1 | Log KAW | Calculation Method | Data Used | Sources |
| --- | --- | --- | --- | --- | --- | --- | --- | --- | --- |
| 1 | 760-42-9 | CF3CF=CHCF3 | 182.04 | kOH \* | 1.02 E-13 | 1.80 | VP molar WS | 172000 Pa 1.10 mol m3 | EST, SciFinder |
| 2 | 354-34-7 | CF3C(O)F | 116.01 | kOH-/H2O | 9.89 E-2 | -1.87 | HLC | 33.7 Pa m3 mol-1 | EXP, FatePointer |
| 3 | 76-05-1 | CF3C(O)OH | 114.02 | None | | -5.34 | HLC | 0.0112 Pa m3 mol-1 | EXP, FatePointer |
| 4 | 75-90-1 | CF3C(O)H | 98.02 | khv | 1.39 E-4 | -1.66 | HLC | 54.1 Pa m3 mol-1 | EXP, FatePointer |
| 5 | 353-50-4 | C(O)F2 | 66.01 | kOH-/H2O | 9.89 E-2 | -0.101 | VP molar WS | 1970 Pa 3020 mol m3 | EXP, FatePointer EST, SciFinder |

## Slide 11
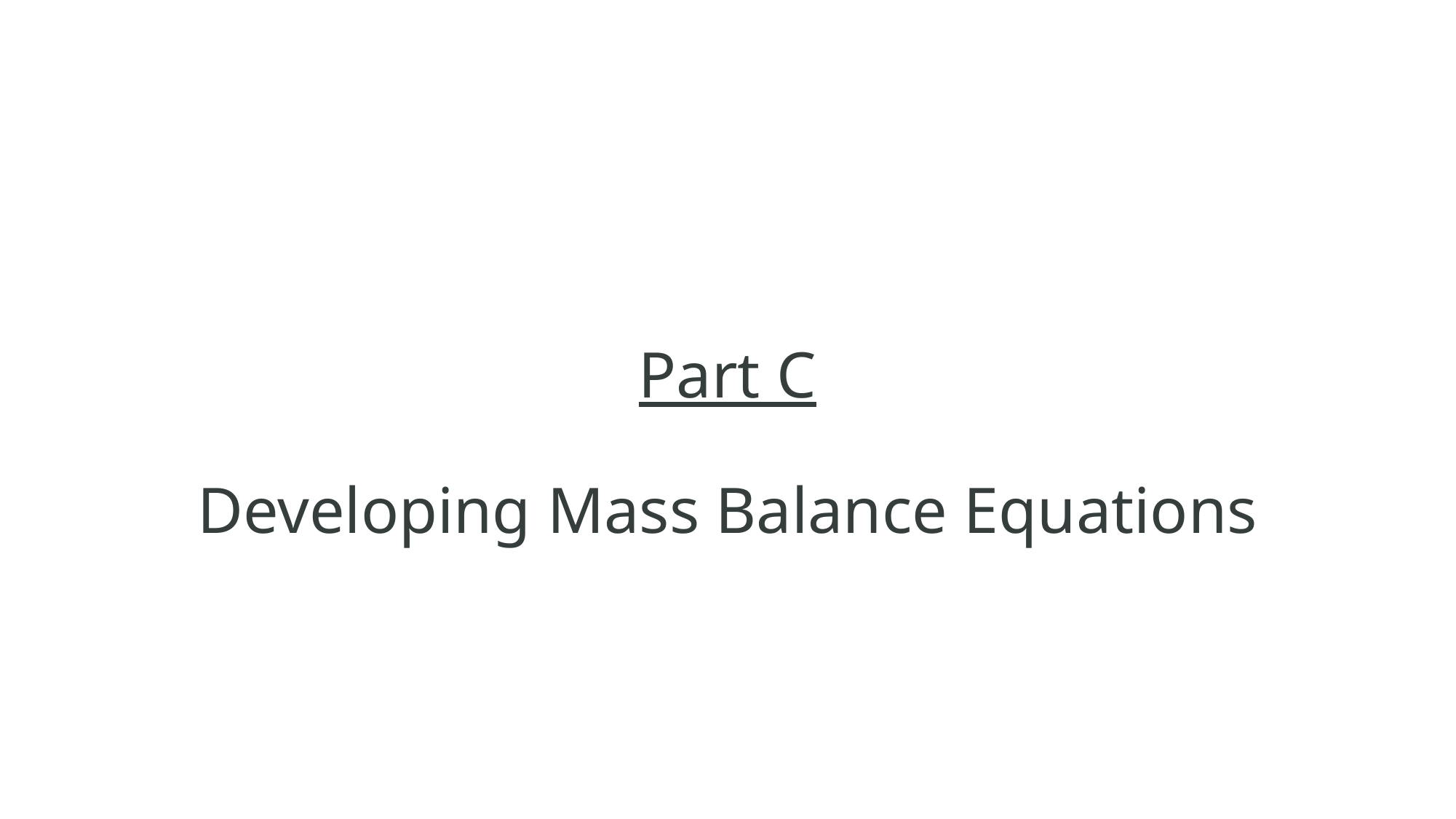

# Part C Developing Mass Balance Equations

## Slide 12
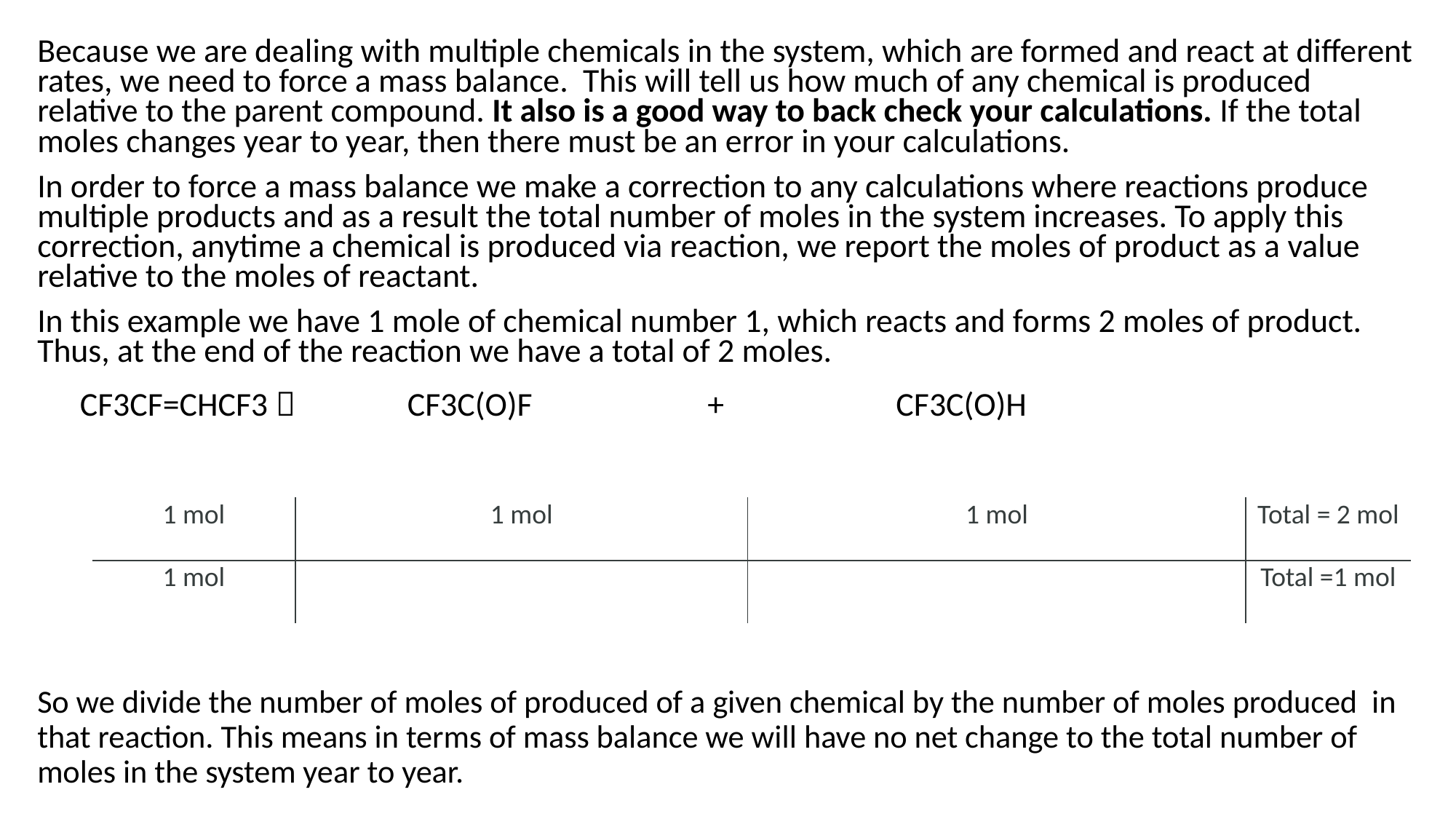

Because we are dealing with multiple chemicals in the system, which are formed and react at different rates, we need to force a mass balance. This will tell us how much of any chemical is produced relative to the parent compound. It also is a good way to back check your calculations. If the total moles changes year to year, then there must be an error in your calculations.
In order to force a mass balance we make a correction to any calculations where reactions produce multiple products and as a result the total number of moles in the system increases. To apply this correction, anytime a chemical is produced via reaction, we report the moles of product as a value relative to the moles of reactant.
In this example we have 1 mole of chemical number 1, which reacts and forms 2 moles of product. Thus, at the end of the reaction we have a total of 2 moles.
CF3CF=CHCF3 		CF3C(O)F	 +		 CF3C(O)H
So we divide the number of moles of produced of a given chemical by the number of moles produced in that reaction. This means in terms of mass balance we will have no net change to the total number of moles in the system year to year.

## Slide 13
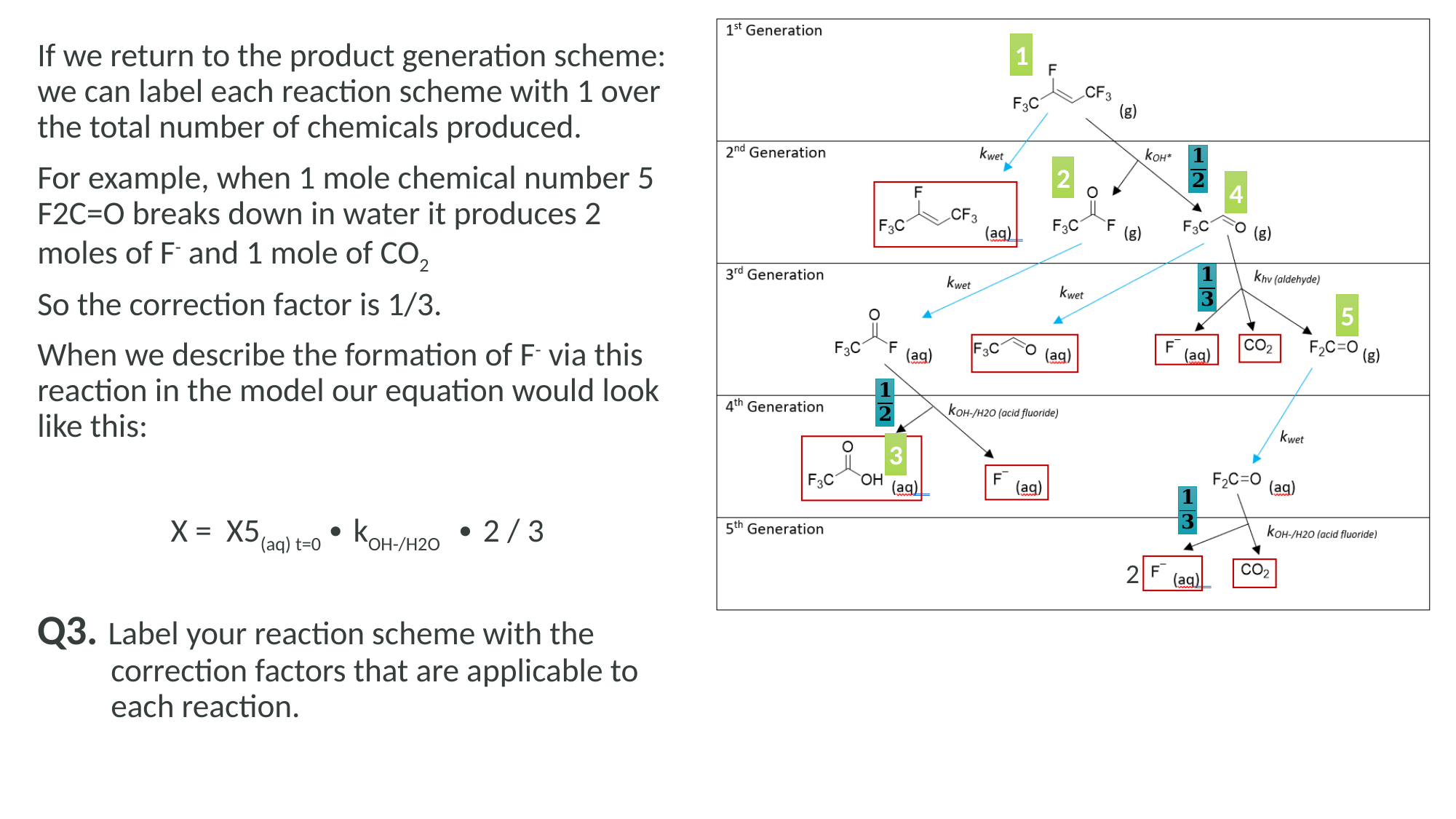

1
2
4
5
3
2
If we return to the product generation scheme: we can label each reaction scheme with 1 over the total number of chemicals produced.
For example, when 1 mole chemical number 5 F2C=O breaks down in water it produces 2 moles of F- and 1 mole of CO2
So the correction factor is 1/3.
When we describe the formation of F- via this reaction in the model our equation would look like this:
X = X5(aq) t=0 ∙ kOH-/H2O ∙ 2 / 3
Q3. Label your reaction scheme with the correction factors that are applicable to each reaction.

## Slide 14
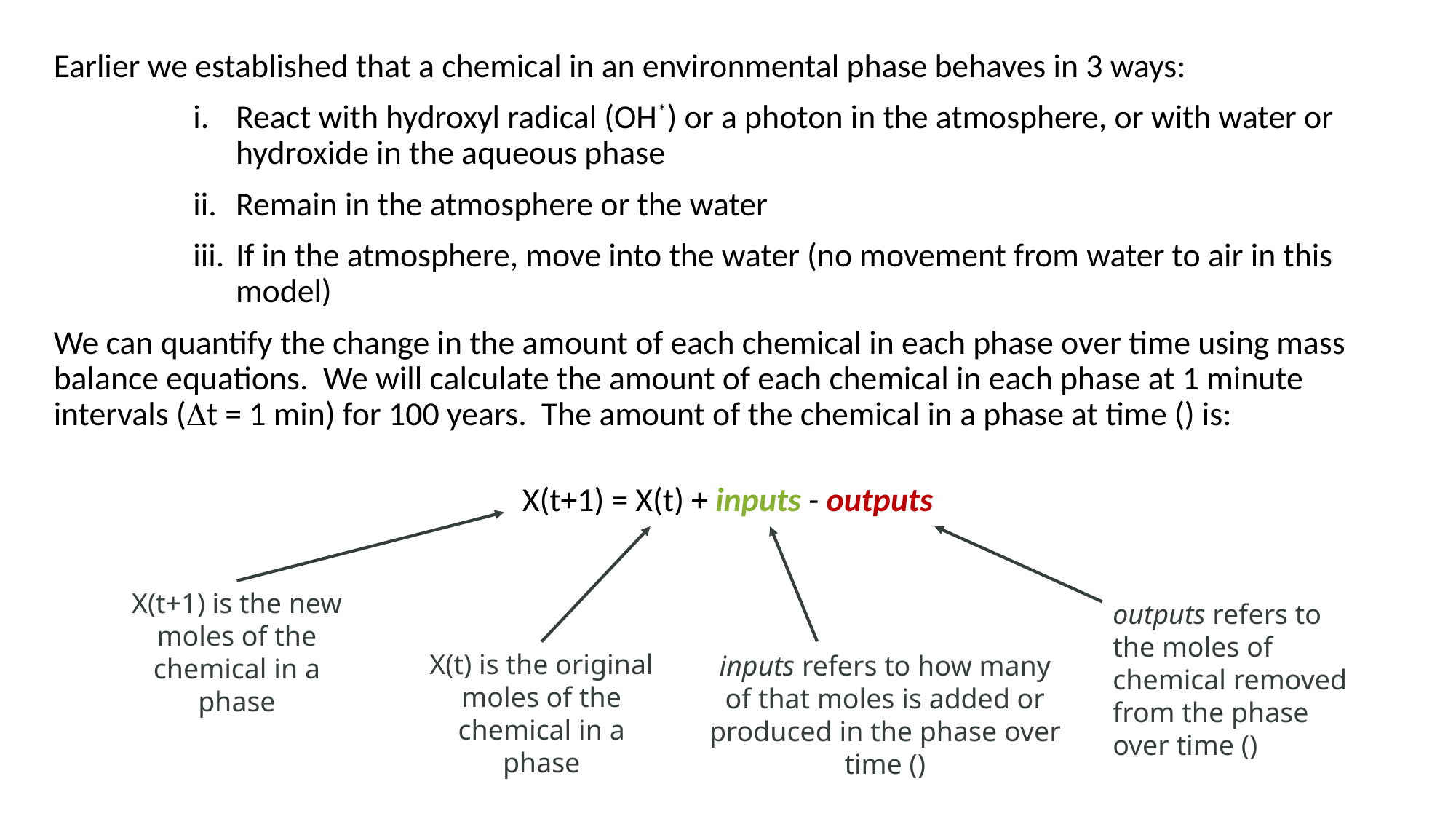

X(t+1) is the new moles of the chemical in a phase
X(t) is the original moles of the chemical in a phase

## Slide 15
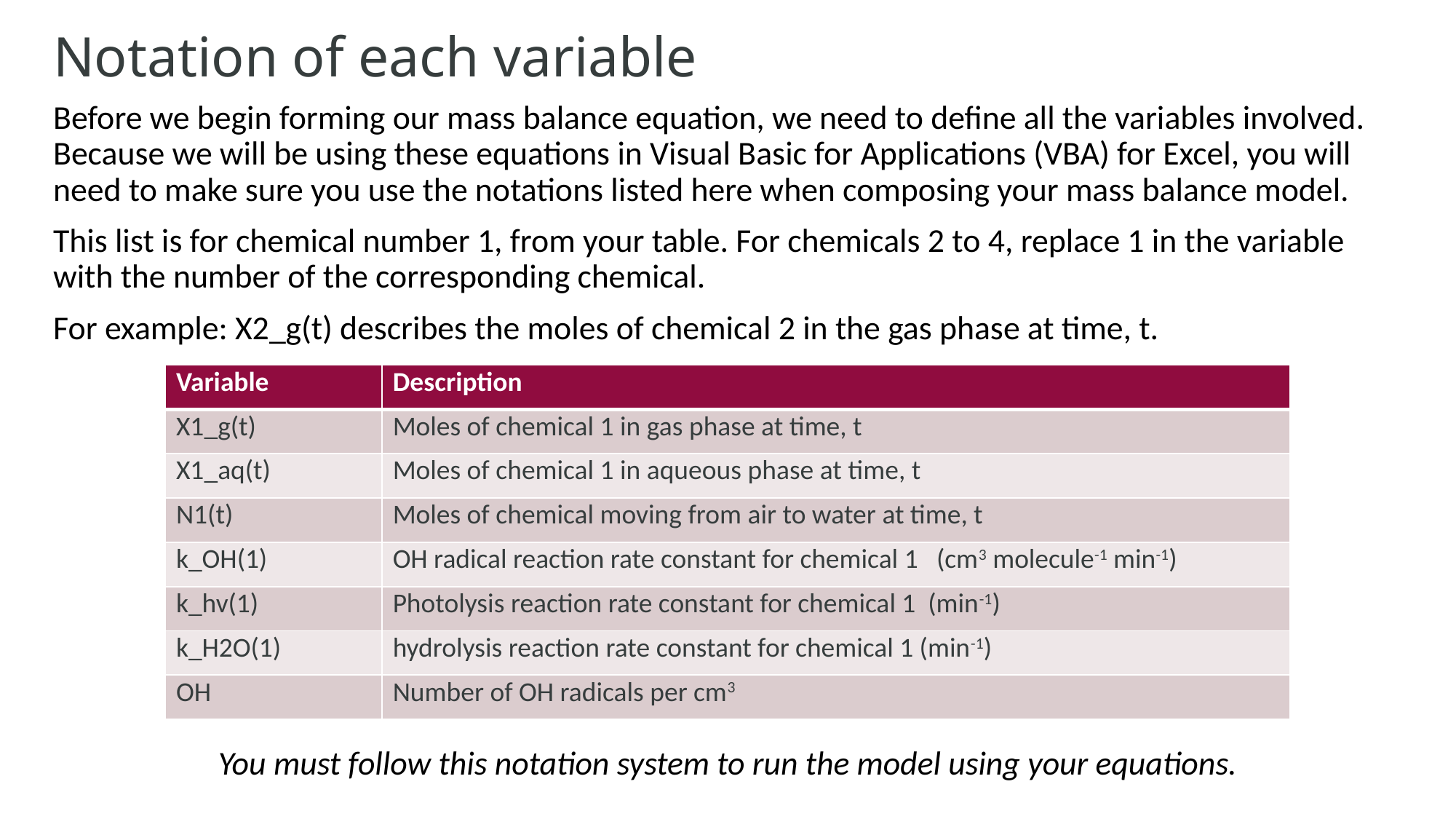

# Notation of each variable
Before we begin forming our mass balance equation, we need to define all the variables involved. Because we will be using these equations in Visual Basic for Applications (VBA) for Excel, you will need to make sure you use the notations listed here when composing your mass balance model.
This list is for chemical number 1, from your table. For chemicals 2 to 4, replace 1 in the variable with the number of the corresponding chemical.
For example: X2_g(t) describes the moles of chemical 2 in the gas phase at time, t.
You must follow this notation system to run the model using your equations.
| Variable | Description |
| --- | --- |
| X1\_g(t) | Moles of chemical 1 in gas phase at time, t |
| X1\_aq(t) | Moles of chemical 1 in aqueous phase at time, t |
| N1(t) | Moles of chemical moving from air to water at time, t |
| k\_OH(1) | OH radical reaction rate constant for chemical 1 (cm3 molecule-1 min-1) |
| k\_hv(1) | Photolysis reaction rate constant for chemical 1 (min-1) |
| k\_H2O(1) | hydrolysis reaction rate constant for chemical 1 (min-1) |
| OH | Number of OH radicals per cm3 |

## Slide 16
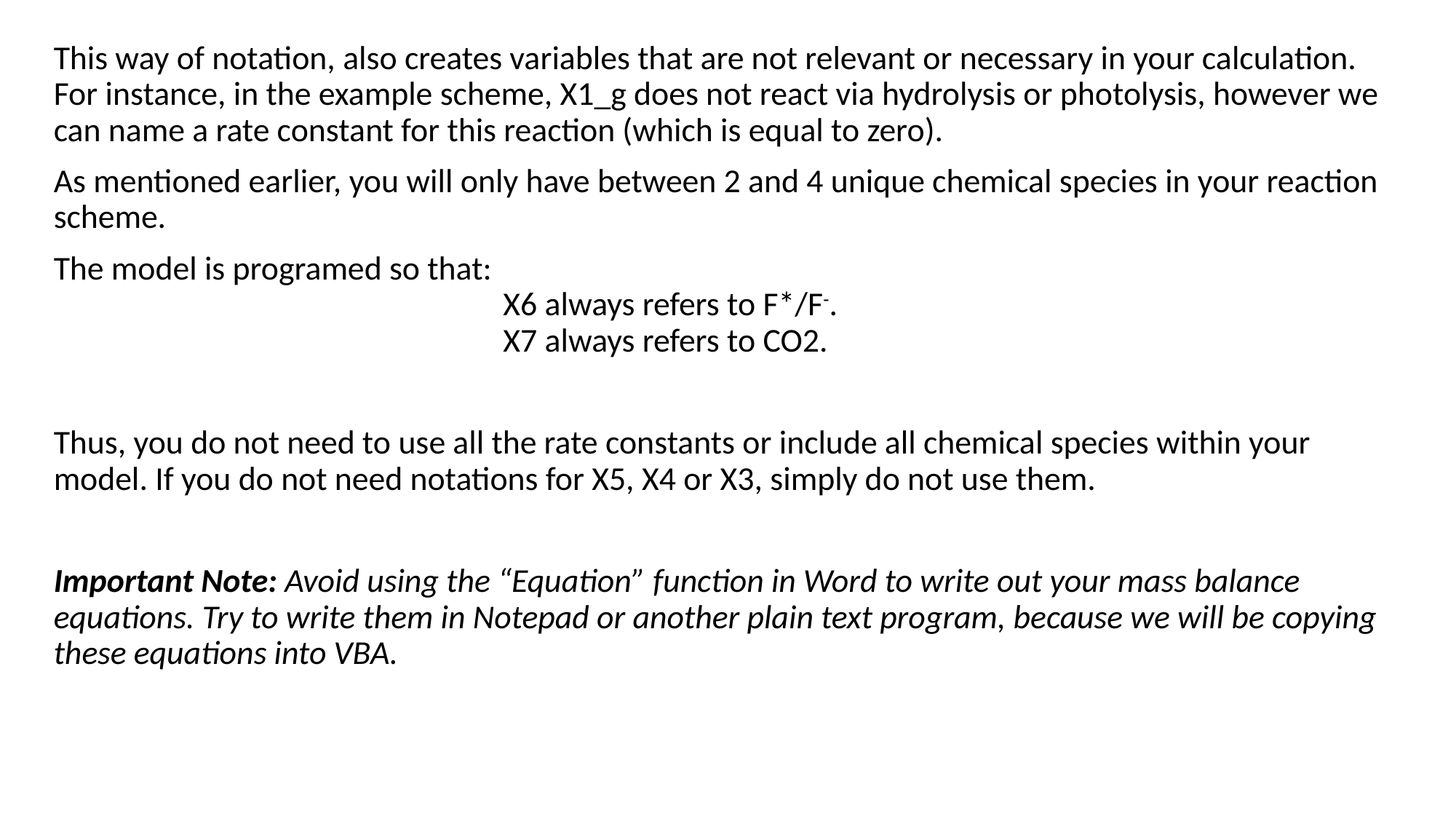

This way of notation, also creates variables that are not relevant or necessary in your calculation. For instance, in the example scheme, X1_g does not react via hydrolysis or photolysis, however we can name a rate constant for this reaction (which is equal to zero).
As mentioned earlier, you will only have between 2 and 4 unique chemical species in your reaction scheme.
The model is programed so that:
X6 always refers to F*/F-.
X7 always refers to CO2.
Thus, you do not need to use all the rate constants or include all chemical species within your model. If you do not need notations for X5, X4 or X3, simply do not use them.
Important Note: Avoid using the “Equation” function in Word to write out your mass balance equations. Try to write them in Notepad or another plain text program, because we will be copying these equations into VBA.

## Slide 17
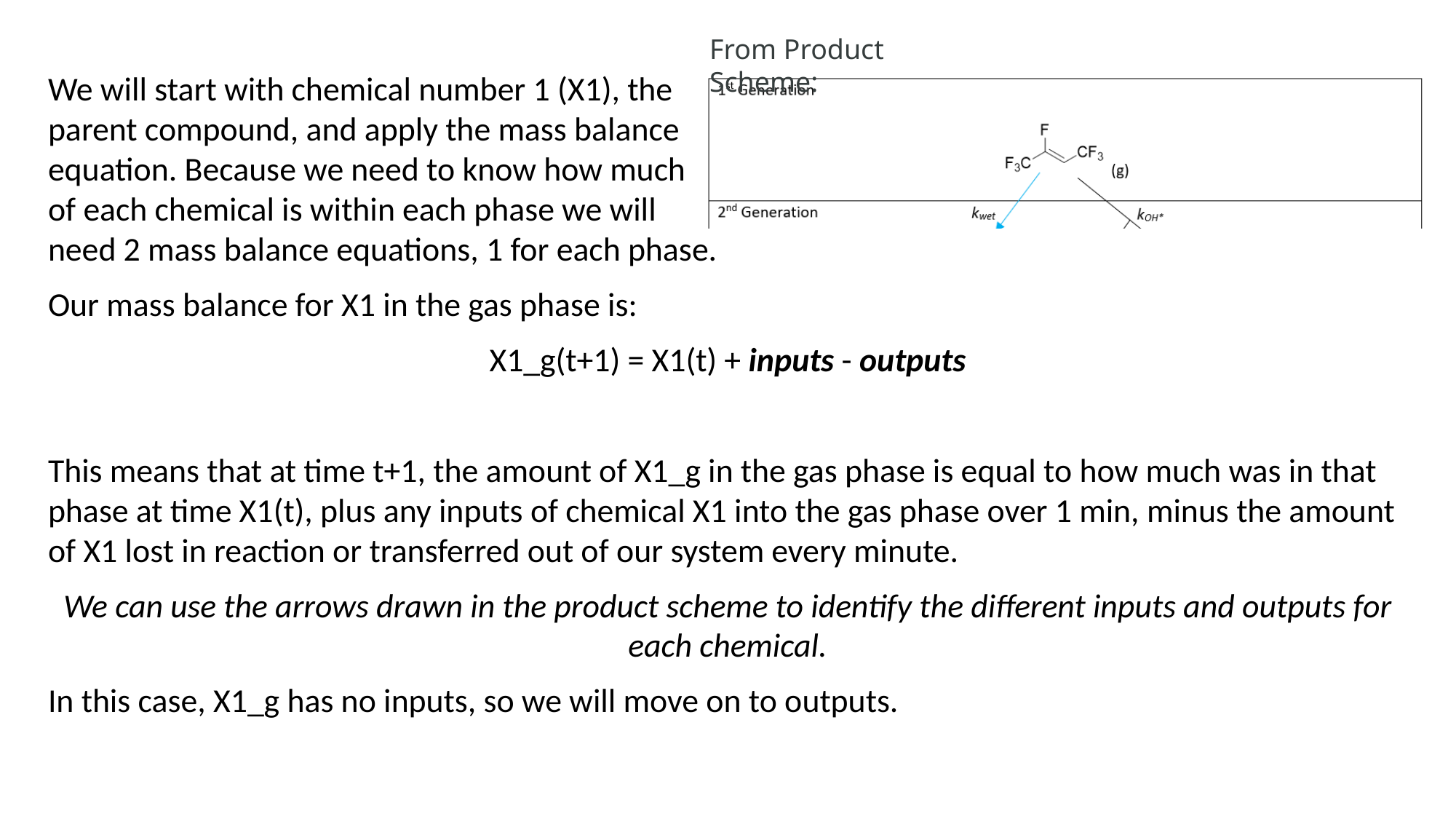

From Product Scheme:
We will start with chemical number 1 (X1), the
parent compound, and apply the mass balance
equation. Because we need to know how much
of each chemical is within each phase we will
need 2 mass balance equations, 1 for each phase.
Our mass balance for X1 in the gas phase is:
X1_g(t+1) = X1(t) + inputs - outputs
This means that at time t+1, the amount of X1_g in the gas phase is equal to how much was in that phase at time X1(t), plus any inputs of chemical X1 into the gas phase over 1 min, minus the amount of X1 lost in reaction or transferred out of our system every minute.
We can use the arrows drawn in the product scheme to identify the different inputs and outputs for each chemical.
In this case, X1_g has no inputs, so we will move on to outputs.

## Slide 18
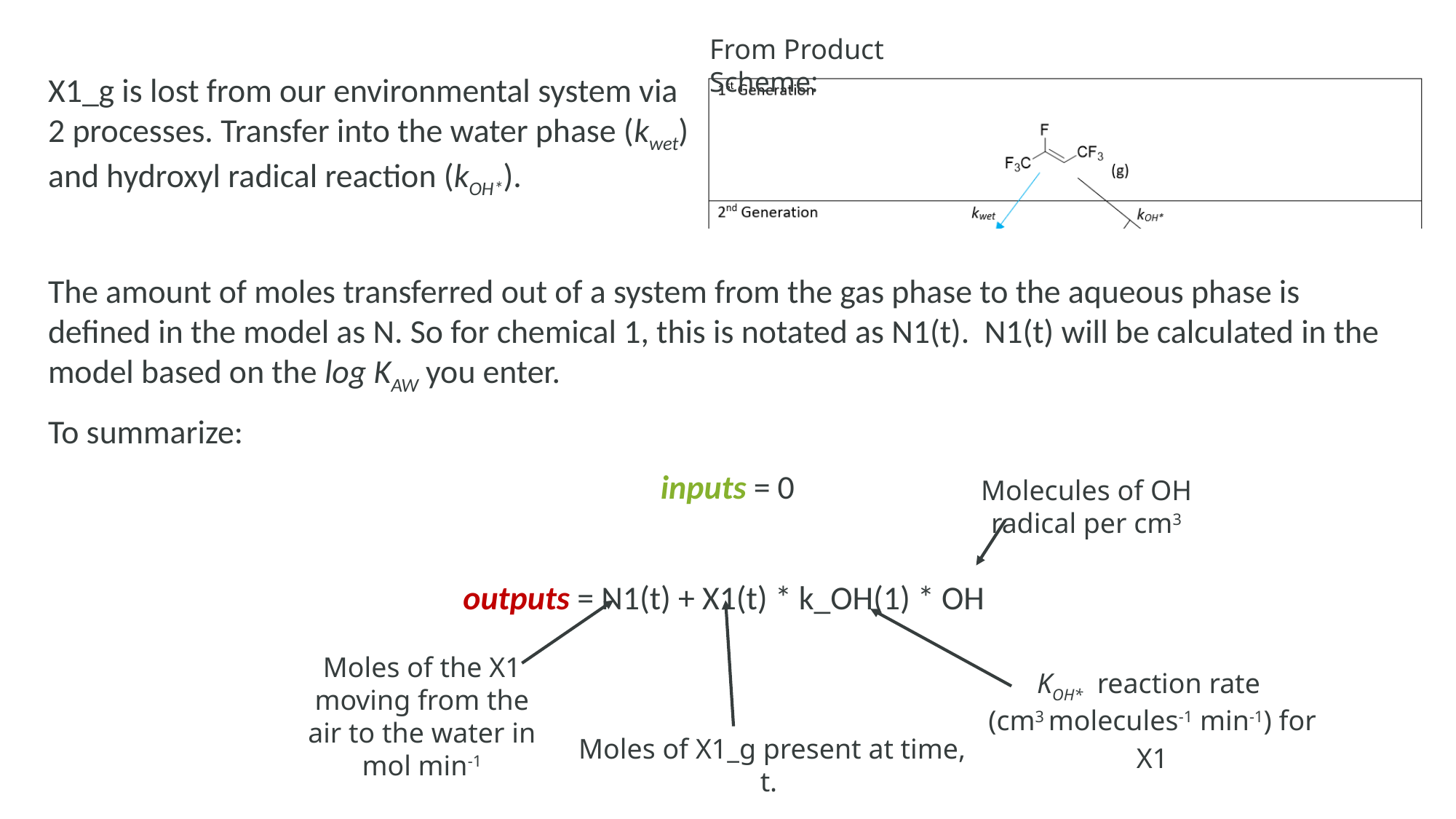

From Product Scheme:
X1_g is lost from our environmental system via
2 processes. Transfer into the water phase (kwet)
and hydroxyl radical reaction (kOH*).
The amount of moles transferred out of a system from the gas phase to the aqueous phase is defined in the model as N. So for chemical 1, this is notated as N1(t). N1(t) will be calculated in the model based on the log KAW you enter.
To summarize:
inputs = 0
outputs = N1(t) + X1(t) * k_OH(1) * OH
Molecules of OH radical per cm3
Moles of the X1 moving from the air to the water in mol min-1
KOH* reaction rate
(cm3 molecules-1 min-1) for X1
Moles of X1_g present at time, t.

## Slide 19
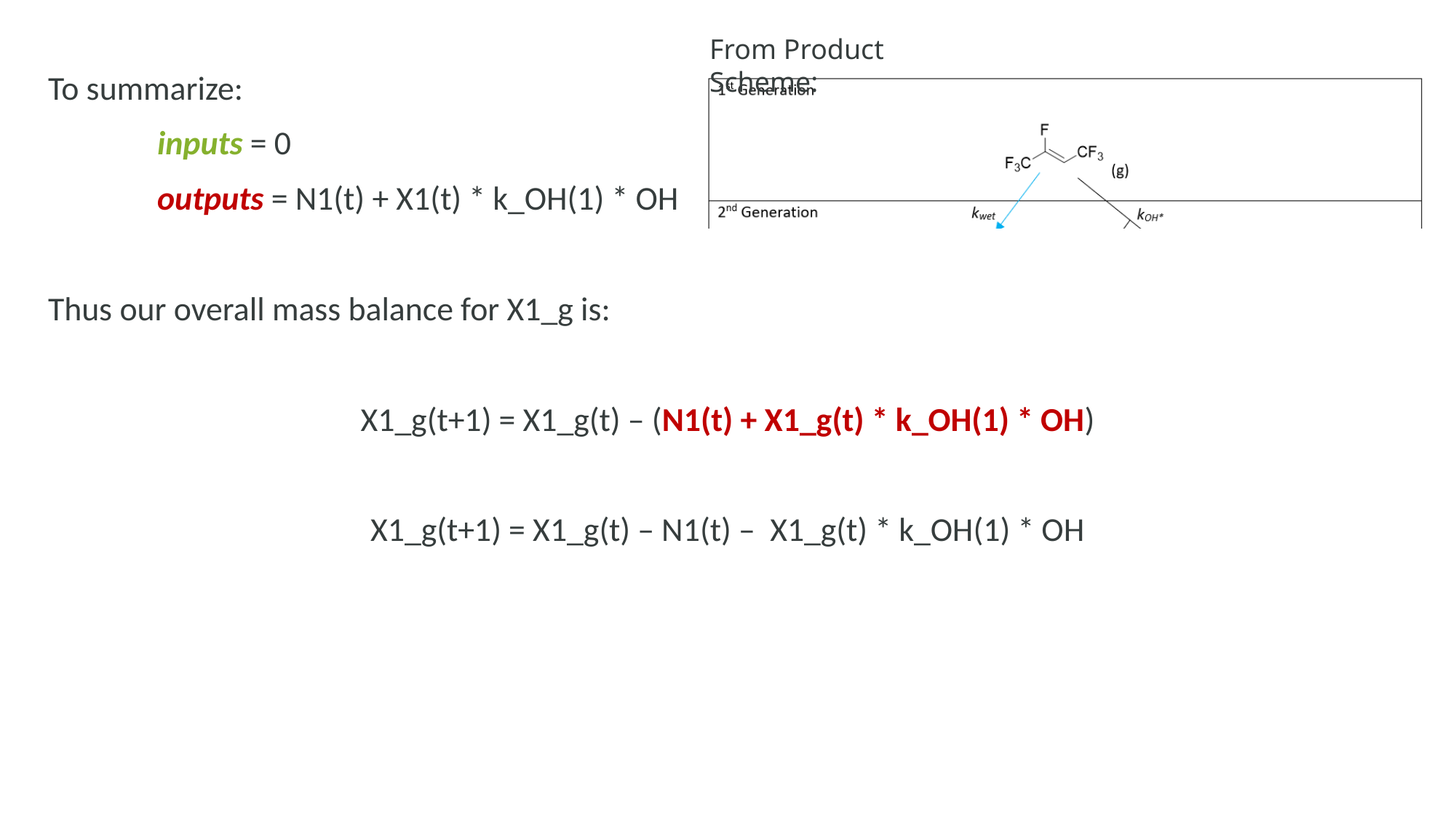

From Product Scheme:
To summarize:
	inputs = 0
	outputs = N1(t) + X1(t) * k_OH(1) * OH
Thus our overall mass balance for X1_g is:
X1_g(t+1) = X1_g(t) – (N1(t) + X1_g(t) * k_OH(1) * OH)
X1_g(t+1) = X1_g(t) – N1(t) – X1_g(t) * k_OH(1) * OH

## Slide 20
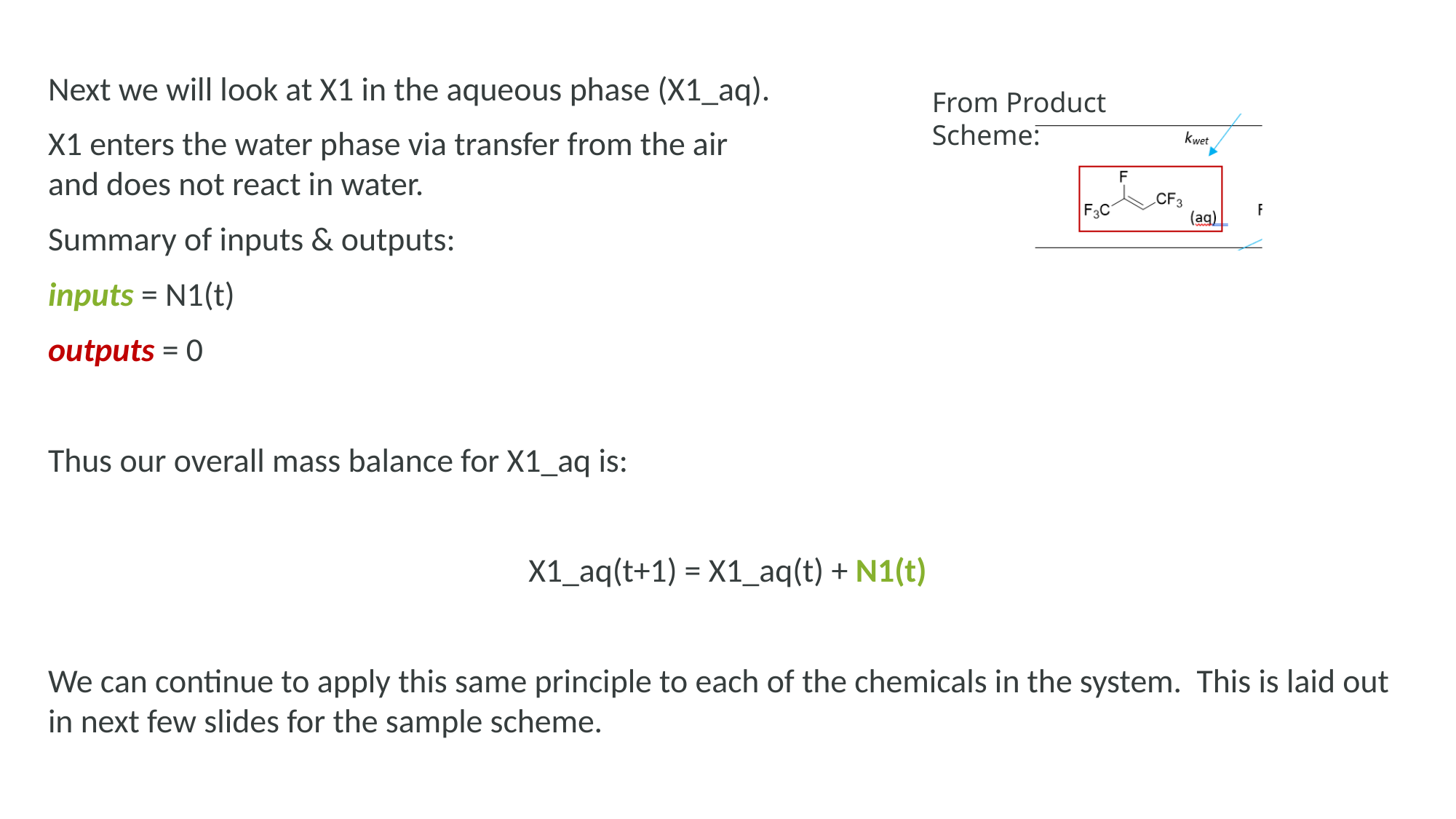

Next we will look at X1 in the aqueous phase (X1_aq).
X1 enters the water phase via transfer from the air
and does not react in water.
Summary of inputs & outputs:
inputs = N1(t)
outputs = 0
Thus our overall mass balance for X1_aq is:
X1_aq(t+1) = X1_aq(t) + N1(t)
We can continue to apply this same principle to each of the chemicals in the system. This is laid out in next few slides for the sample scheme.
From Product Scheme:

## Slide 21
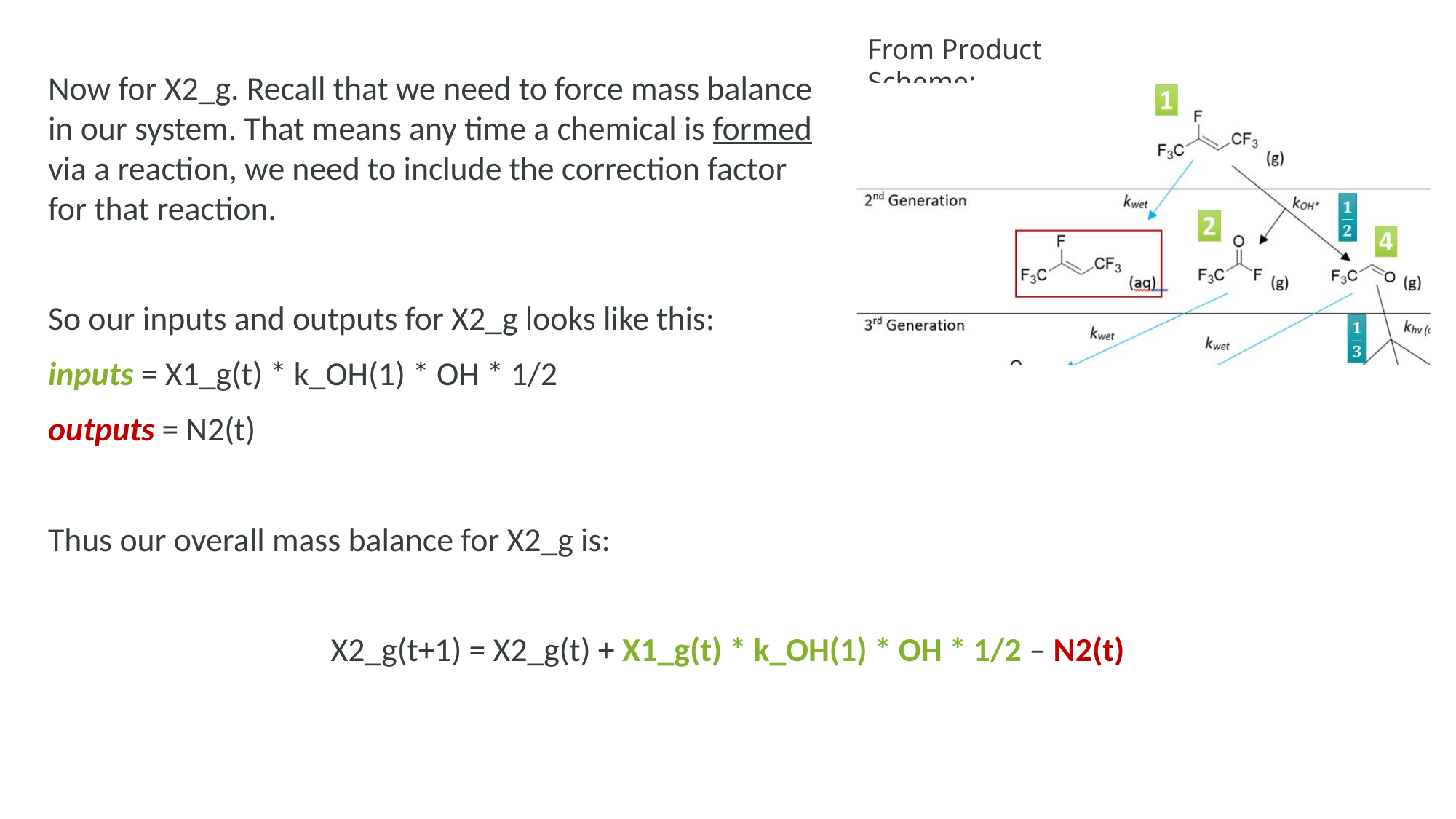

From Product Scheme:
Now for X2_g. Recall that we need to force mass balance
in our system. That means any time a chemical is formed
via a reaction, we need to include the correction factor
for that reaction.
So our inputs and outputs for X2_g looks like this:
inputs = X1_g(t) * k_OH(1) * OH * 1/2
outputs = N2(t)
Thus our overall mass balance for X2_g is:
X2_g(t+1) = X2_g(t) + X1_g(t) * k_OH(1) * OH * 1/2 – N2(t)

## Slide 22
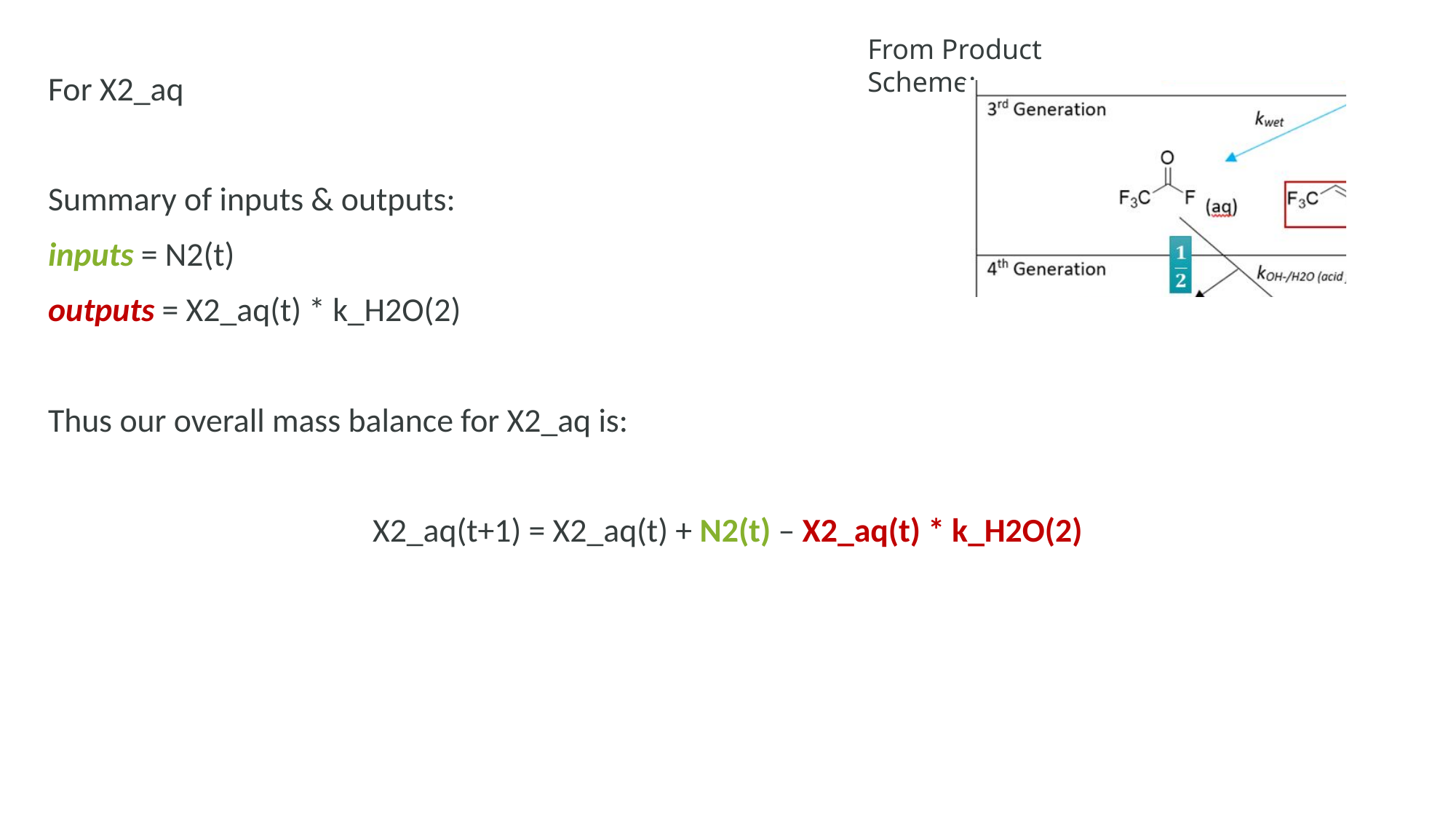

From Product Scheme:
For X2_aq
Summary of inputs & outputs:
inputs = N2(t)
outputs = X2_aq(t) * k_H2O(2)
Thus our overall mass balance for X2_aq is:
X2_aq(t+1) = X2_aq(t) + N2(t) – X2_aq(t) * k_H2O(2)

## Slide 23
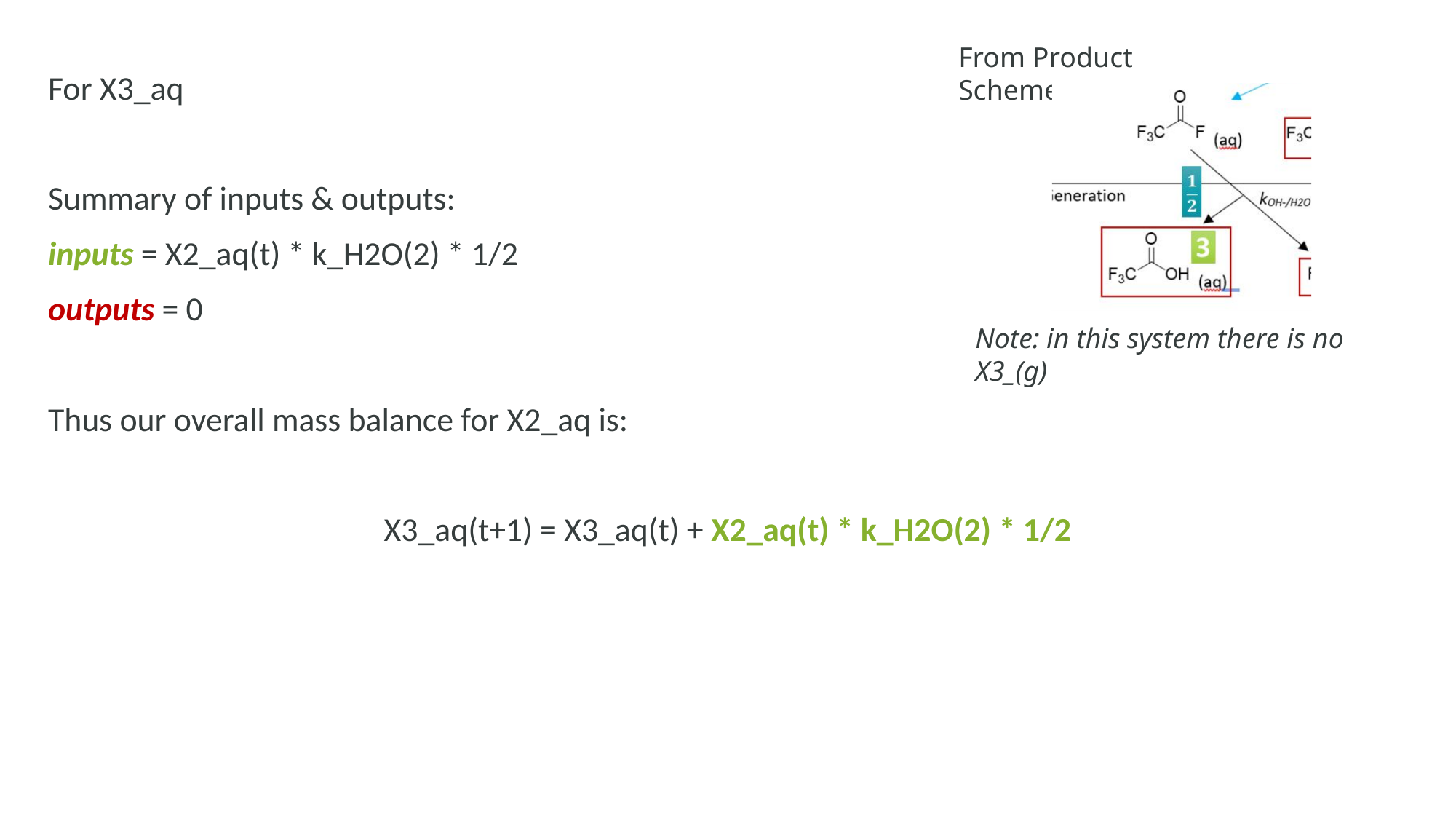

From Product Scheme:
For X3_aq
Summary of inputs & outputs:
inputs = X2_aq(t) * k_H2O(2) * 1/2
outputs = 0
Thus our overall mass balance for X2_aq is:
X3_aq(t+1) = X3_aq(t) + X2_aq(t) * k_H2O(2) * 1/2
Note: in this system there is no X3_(g)

## Slide 24
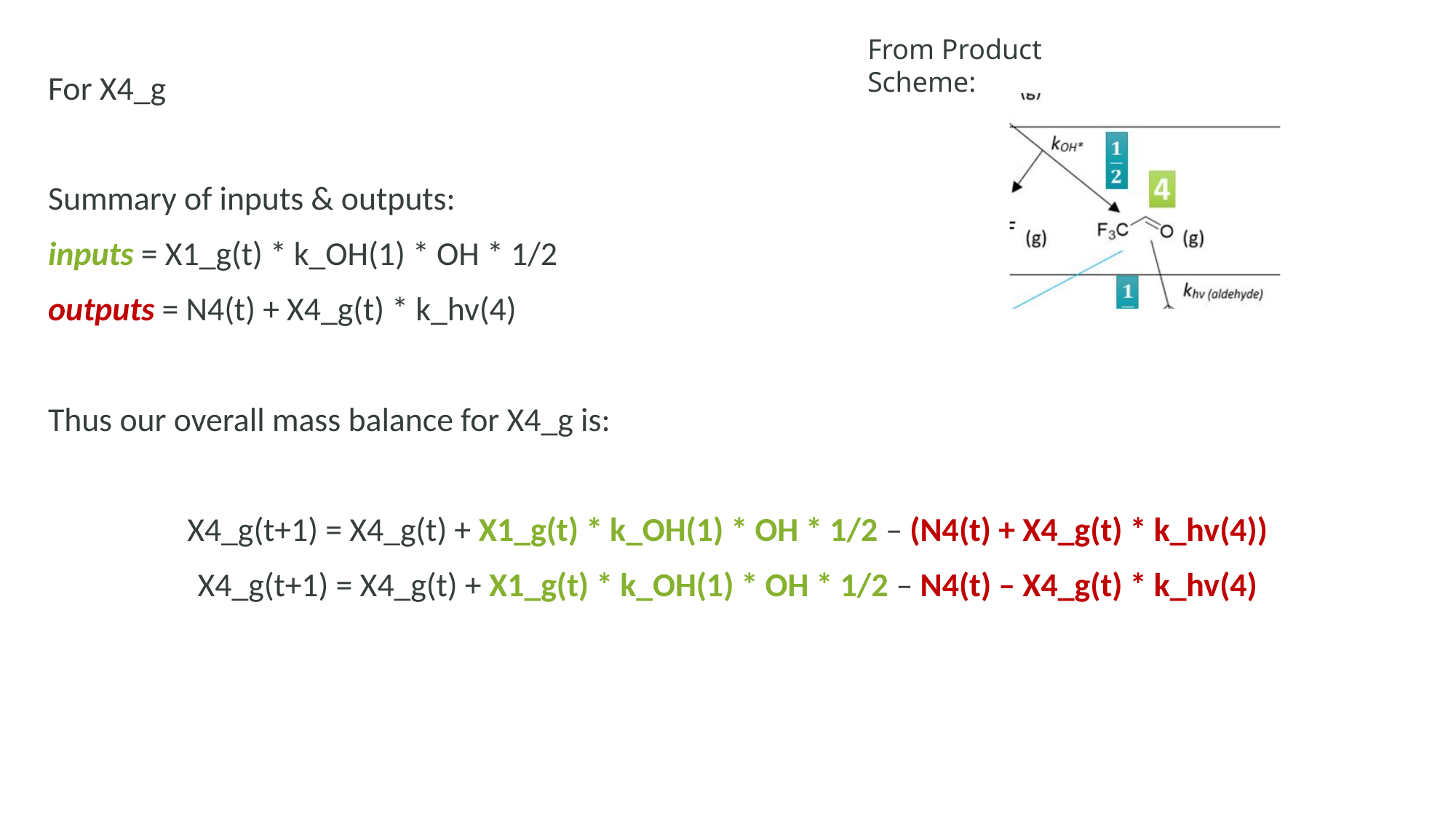

From Product Scheme:
For X4_g
Summary of inputs & outputs:
inputs = X1_g(t) * k_OH(1) * OH * 1/2
outputs = N4(t) + X4_g(t) * k_hv(4)
Thus our overall mass balance for X4_g is:
X4_g(t+1) = X4_g(t) + X1_g(t) * k_OH(1) * OH * 1/2 – (N4(t) + X4_g(t) * k_hv(4))
X4_g(t+1) = X4_g(t) + X1_g(t) * k_OH(1) * OH * 1/2 – N4(t) – X4_g(t) * k_hv(4)

## Slide 25
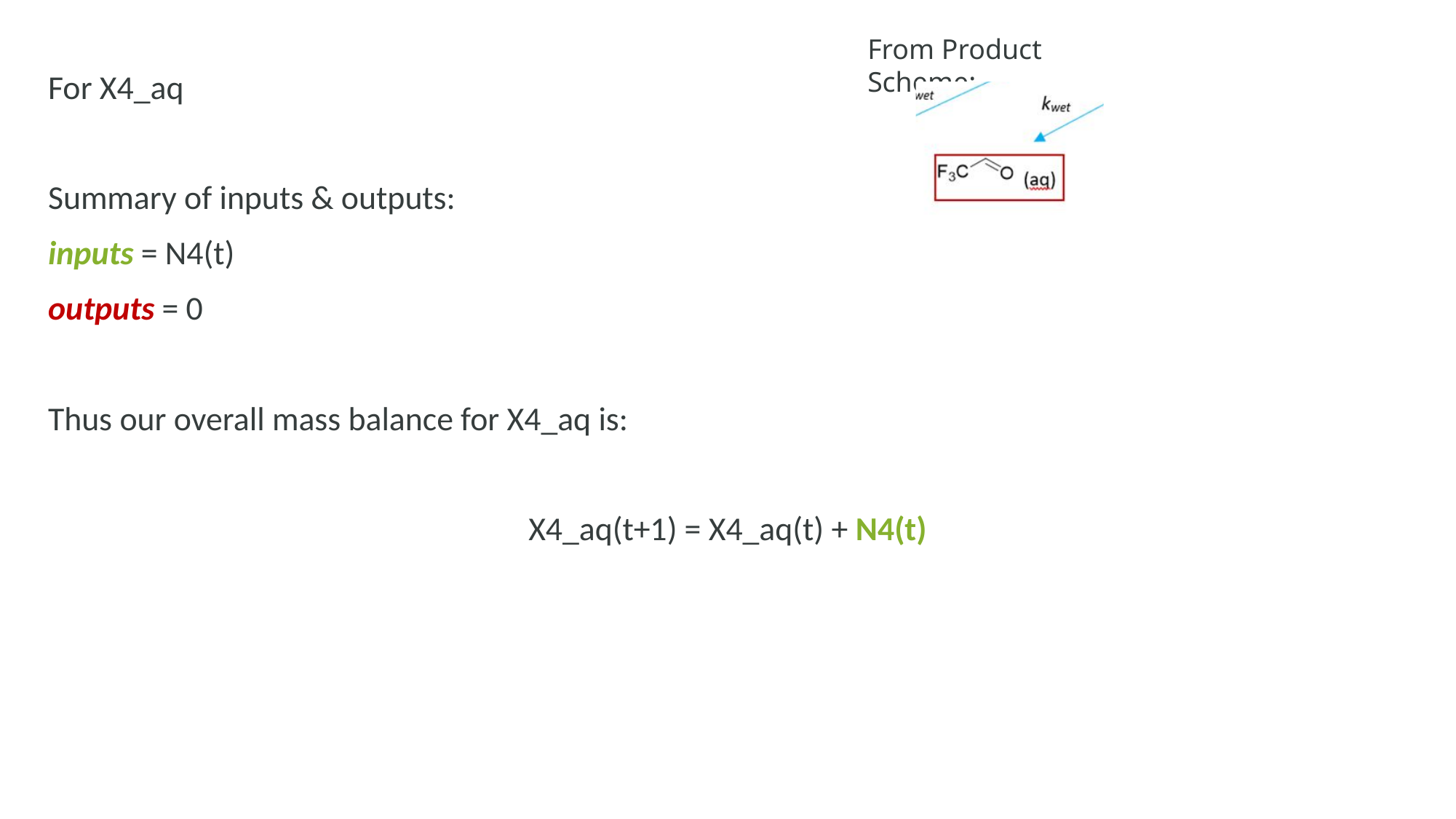

From Product Scheme:
For X4_aq
Summary of inputs & outputs:
inputs = N4(t)
outputs = 0
Thus our overall mass balance for X4_aq is:
X4_aq(t+1) = X4_aq(t) + N4(t)

## Slide 26
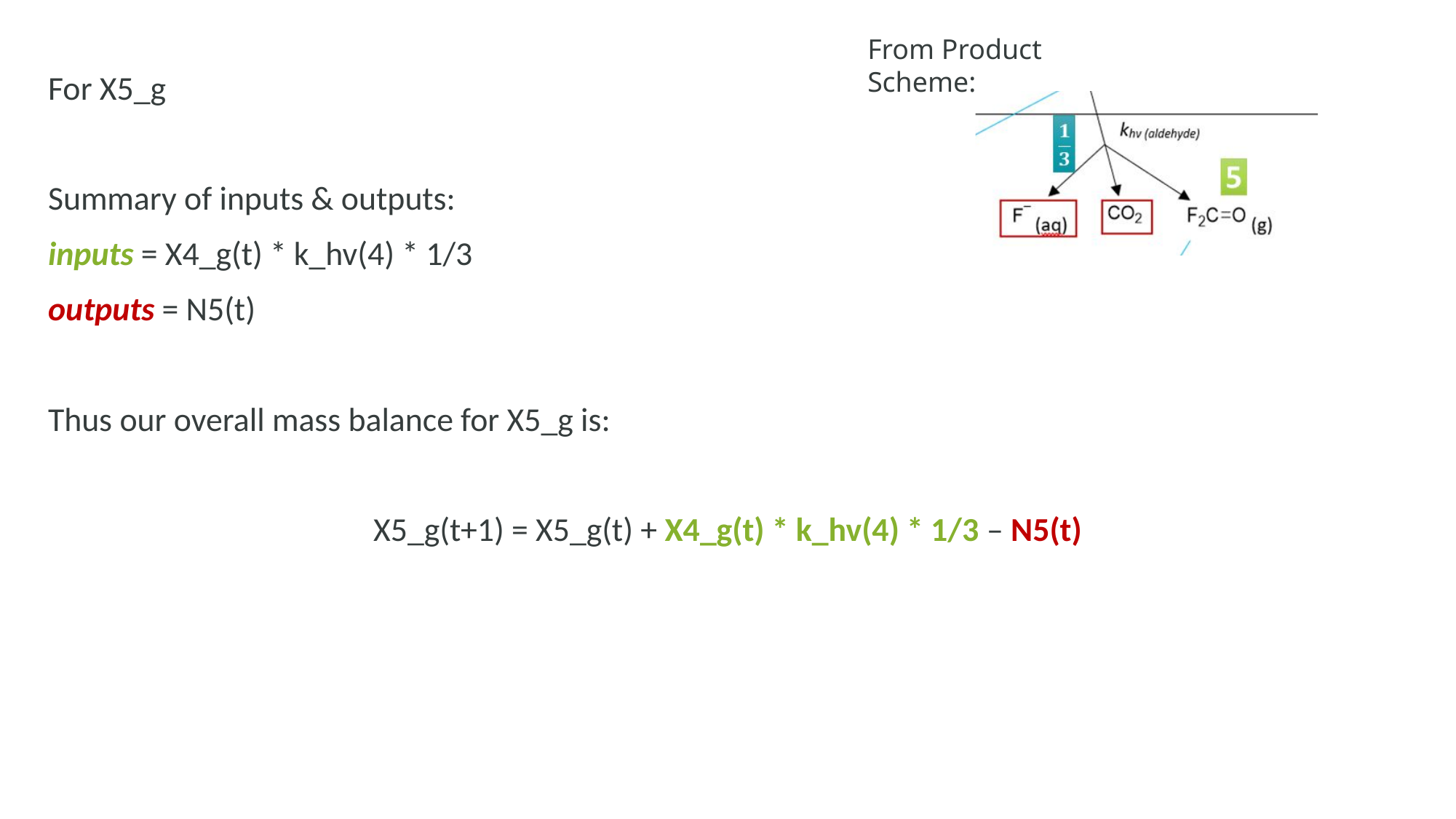

From Product Scheme:
For X5_g
Summary of inputs & outputs:
inputs = X4_g(t) * k_hv(4) * 1/3
outputs = N5(t)
Thus our overall mass balance for X5_g is:
X5_g(t+1) = X5_g(t) + X4_g(t) * k_hv(4) * 1/3 – N5(t)

## Slide 27
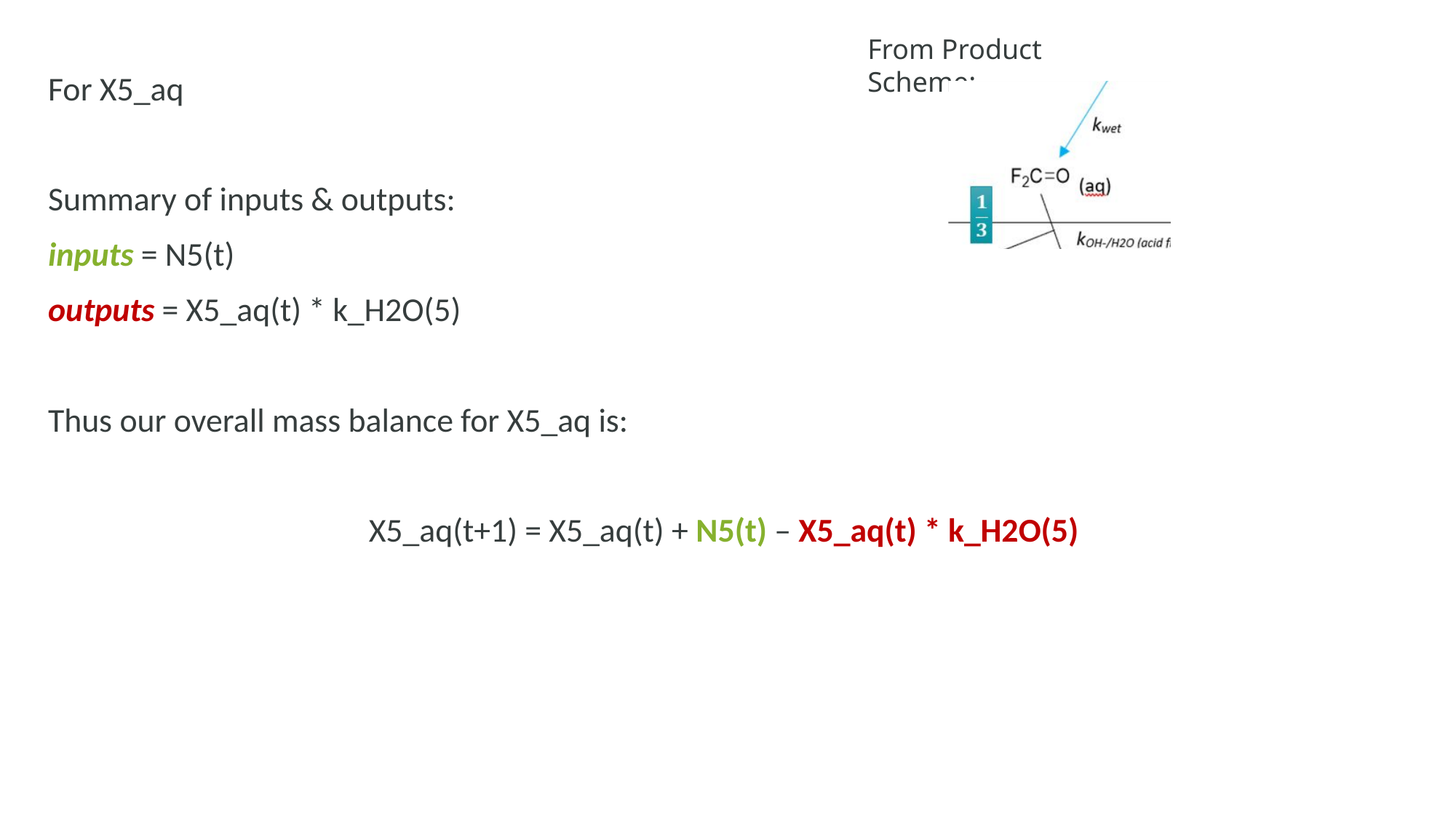

From Product Scheme:
For X5_aq
Summary of inputs & outputs:
inputs = N5(t)
outputs = X5_aq(t) * k_H2O(5)
Thus our overall mass balance for X5_aq is:
X5_aq(t+1) = X5_aq(t) + N5(t) – X5_aq(t) * k_H2O(5)

## Slide 28
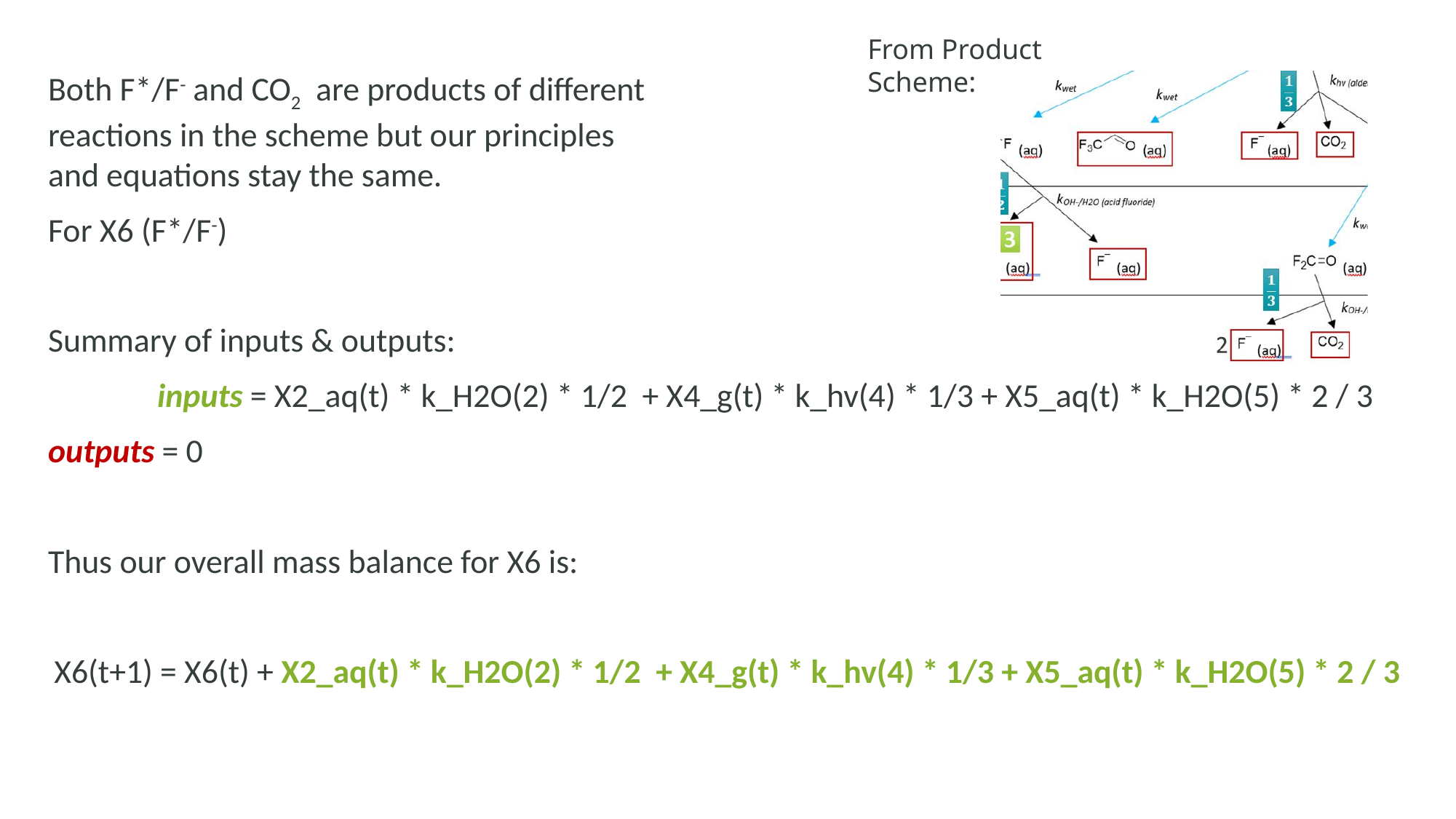

From Product Scheme:
Both F*/F- and CO2 are products of different
reactions in the scheme but our principles
and equations stay the same.
For X6 (F*/F-)
Summary of inputs & outputs:
	inputs = X2_aq(t) * k_H2O(2) * 1/2 + X4_g(t) * k_hv(4) * 1/3 + X5_aq(t) * k_H2O(5) * 2 / 3
outputs = 0
Thus our overall mass balance for X6 is:
X6(t+1) = X6(t) + X2_aq(t) * k_H2O(2) * 1/2 + X4_g(t) * k_hv(4) * 1/3 + X5_aq(t) * k_H2O(5) * 2 / 3

## Slide 29
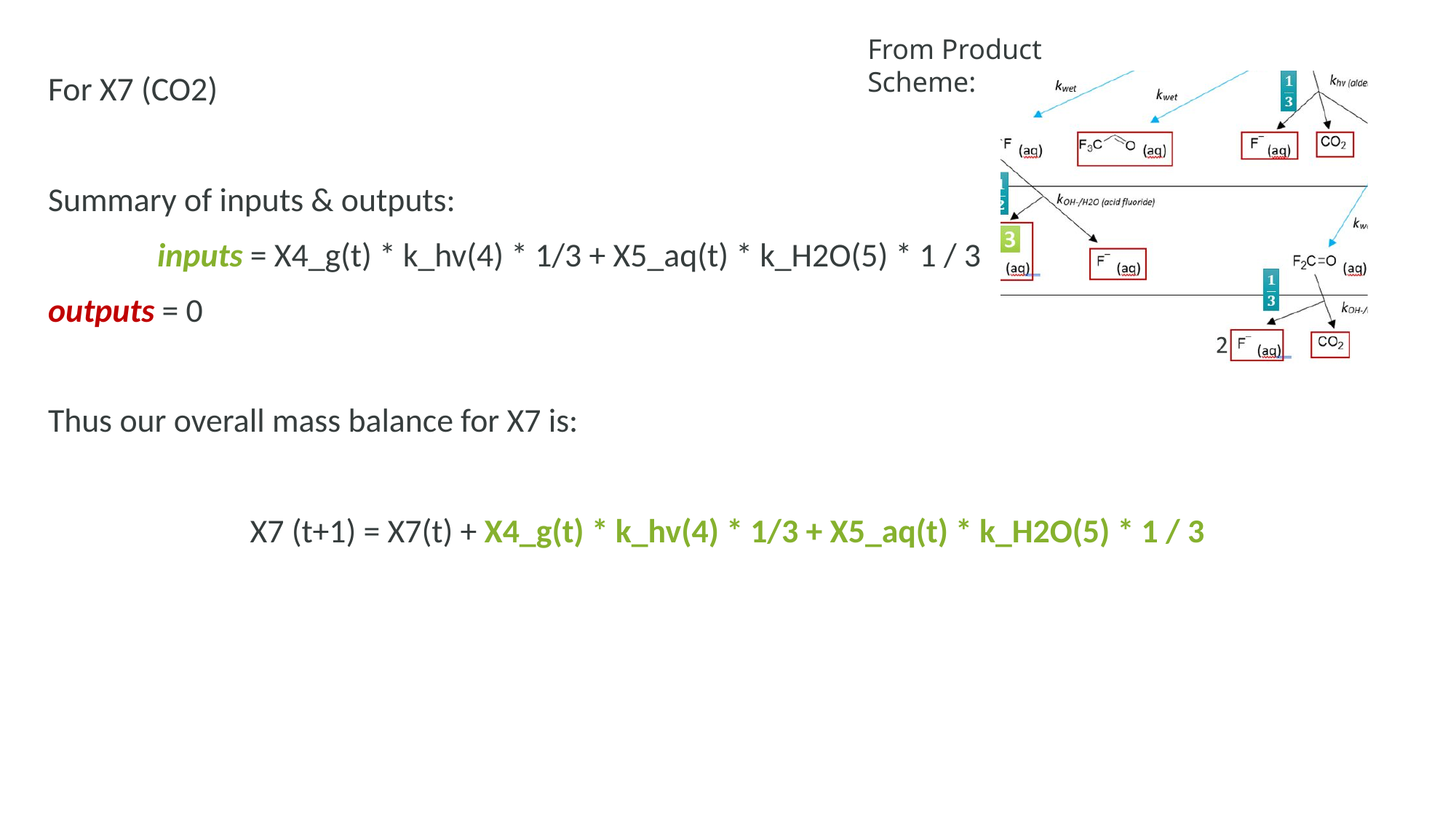

From Product Scheme:
For X7 (CO2)
Summary of inputs & outputs:
	inputs = X4_g(t) * k_hv(4) * 1/3 + X5_aq(t) * k_H2O(5) * 1 / 3
outputs = 0
Thus our overall mass balance for X7 is:
X7 (t+1) = X7(t) + X4_g(t) * k_hv(4) * 1/3 + X5_aq(t) * k_H2O(5) * 1 / 3

## Slide 30
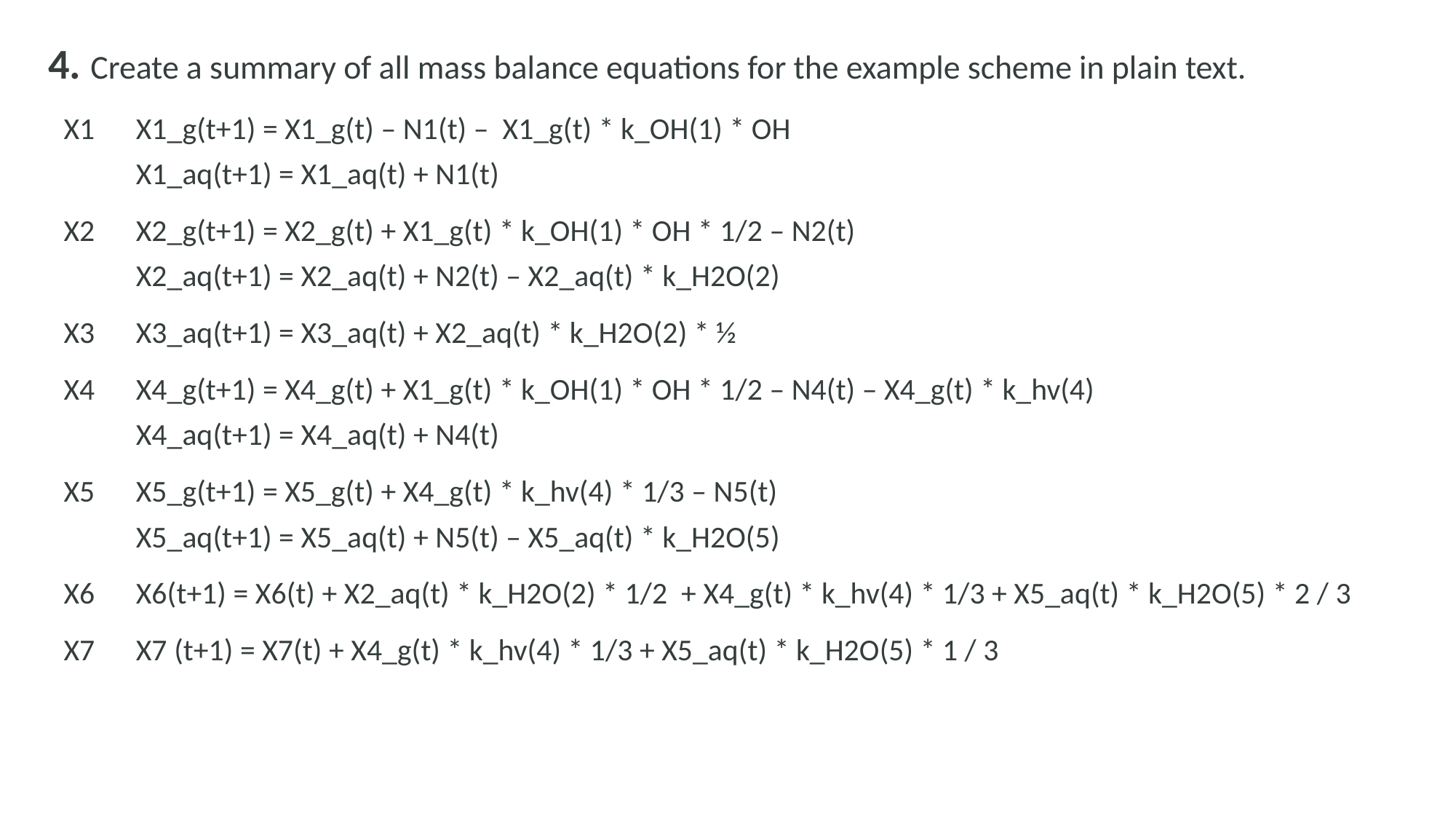

4. Create a summary of all mass balance equations for the example scheme in plain text.
| X1 | X1\_g(t+1) = X1\_g(t) – N1(t) – X1\_g(t) \* k\_OH(1) \* OH X1\_aq(t+1) = X1\_aq(t) + N1(t) |
| --- | --- |
| X2 | X2\_g(t+1) = X2\_g(t) + X1\_g(t) \* k\_OH(1) \* OH \* 1/2 – N2(t) X2\_aq(t+1) = X2\_aq(t) + N2(t) – X2\_aq(t) \* k\_H2O(2) |
| X3 | X3\_aq(t+1) = X3\_aq(t) + X2\_aq(t) \* k\_H2O(2) \* ½ |
| X4 | X4\_g(t+1) = X4\_g(t) + X1\_g(t) \* k\_OH(1) \* OH \* 1/2 – N4(t) – X4\_g(t) \* k\_hv(4) X4\_aq(t+1) = X4\_aq(t) + N4(t) |
| X5 | X5\_g(t+1) = X5\_g(t) + X4\_g(t) \* k\_hv(4) \* 1/3 – N5(t) X5\_aq(t+1) = X5\_aq(t) + N5(t) – X5\_aq(t) \* k\_H2O(5) |
| X6 | X6(t+1) = X6(t) + X2\_aq(t) \* k\_H2O(2) \* 1/2 + X4\_g(t) \* k\_hv(4) \* 1/3 + X5\_aq(t) \* k\_H2O(5) \* 2 / 3 |
| X7 | X7 (t+1) = X7(t) + X4\_g(t) \* k\_hv(4) \* 1/3 + X5\_aq(t) \* k\_H2O(5) \* 1 / 3 |

## Slide 31
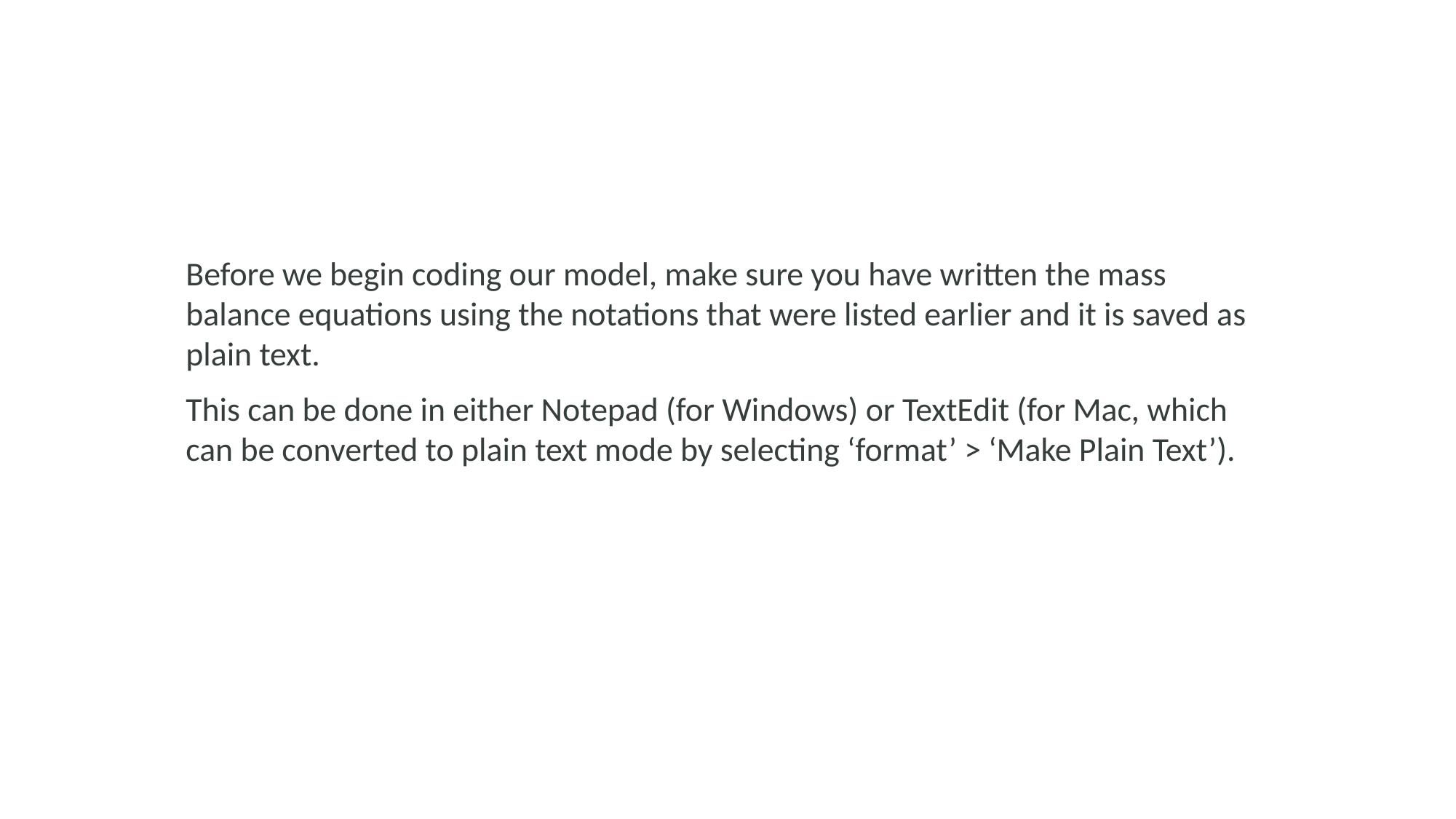

Before we begin coding our model, make sure you have written the mass balance equations using the notations that were listed earlier and it is saved as plain text.
This can be done in either Notepad (for Windows) or TextEdit (for Mac, which can be converted to plain text mode by selecting ‘format’ > ‘Make Plain Text’).

## Slide 32
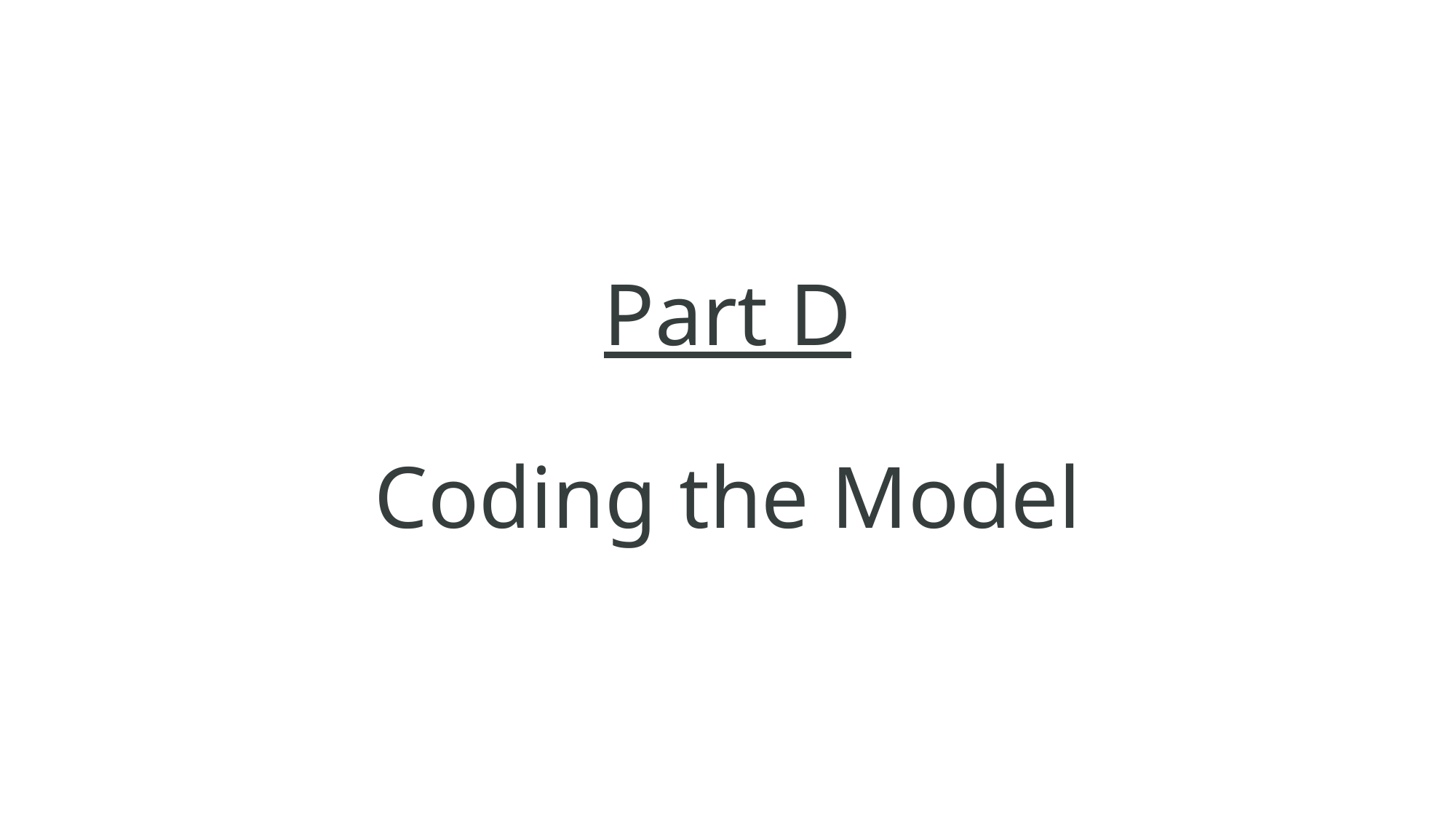

# Part D Coding the Model

## Slide 33
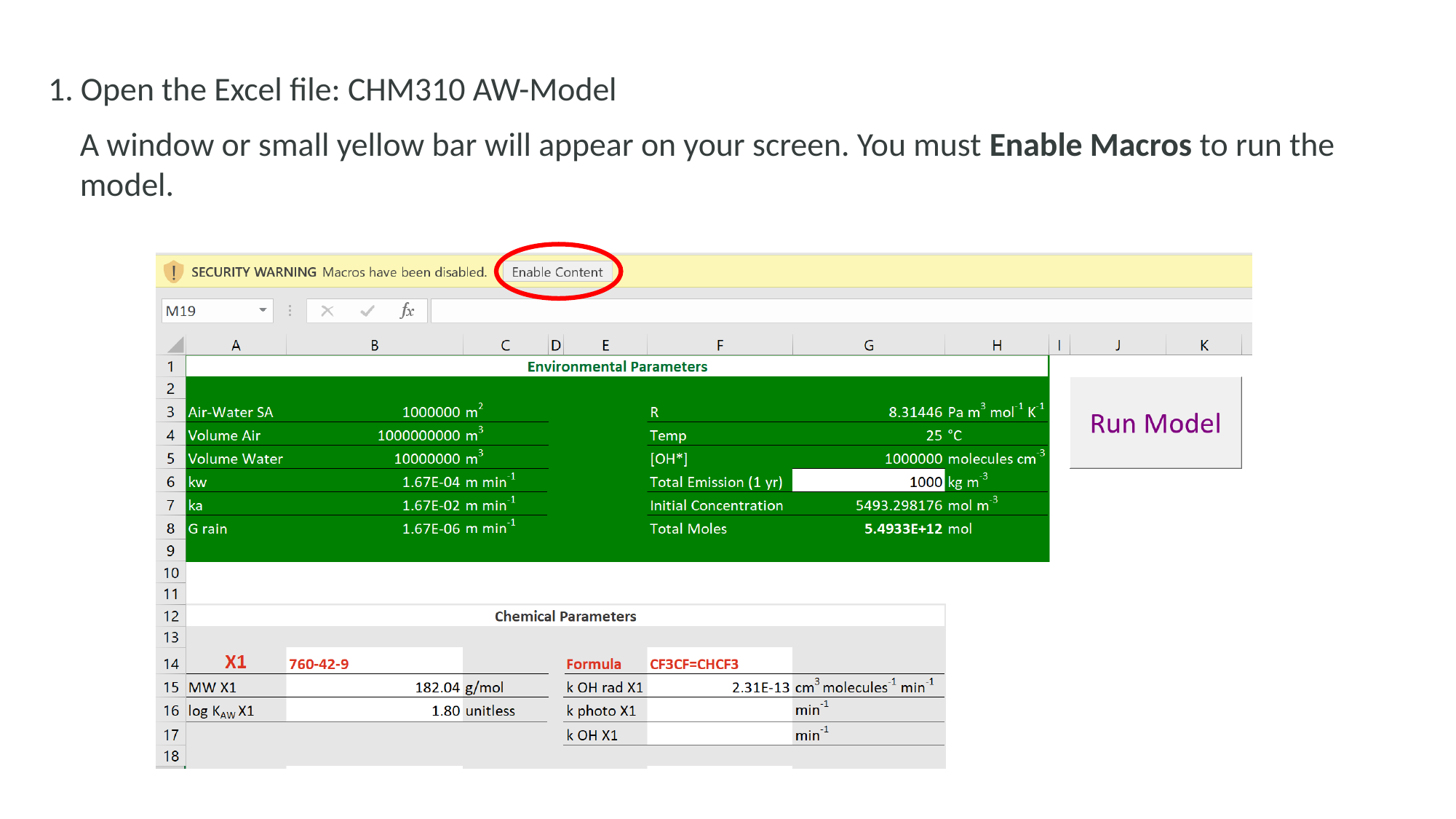

1. Open the Excel file: CHM310 AW-Model
A window or small yellow bar will appear on your screen. You must Enable Macros to run the model.

## Slide 34
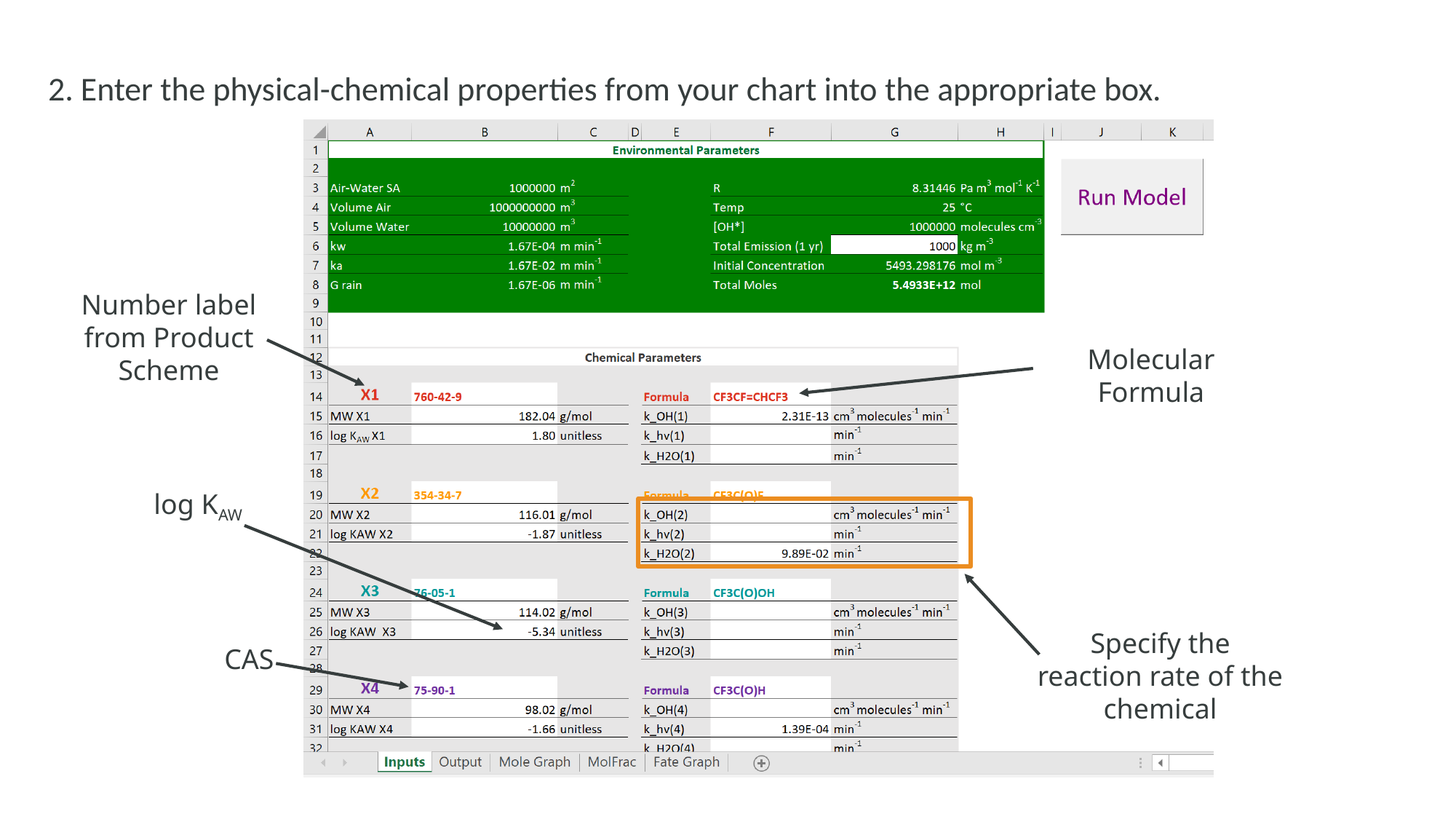

2. Enter the physical-chemical properties from your chart into the appropriate box.
Number label from Product Scheme
Molecular Formula
log KAW
Specify the reaction rate of the chemical
CAS

## Slide 35
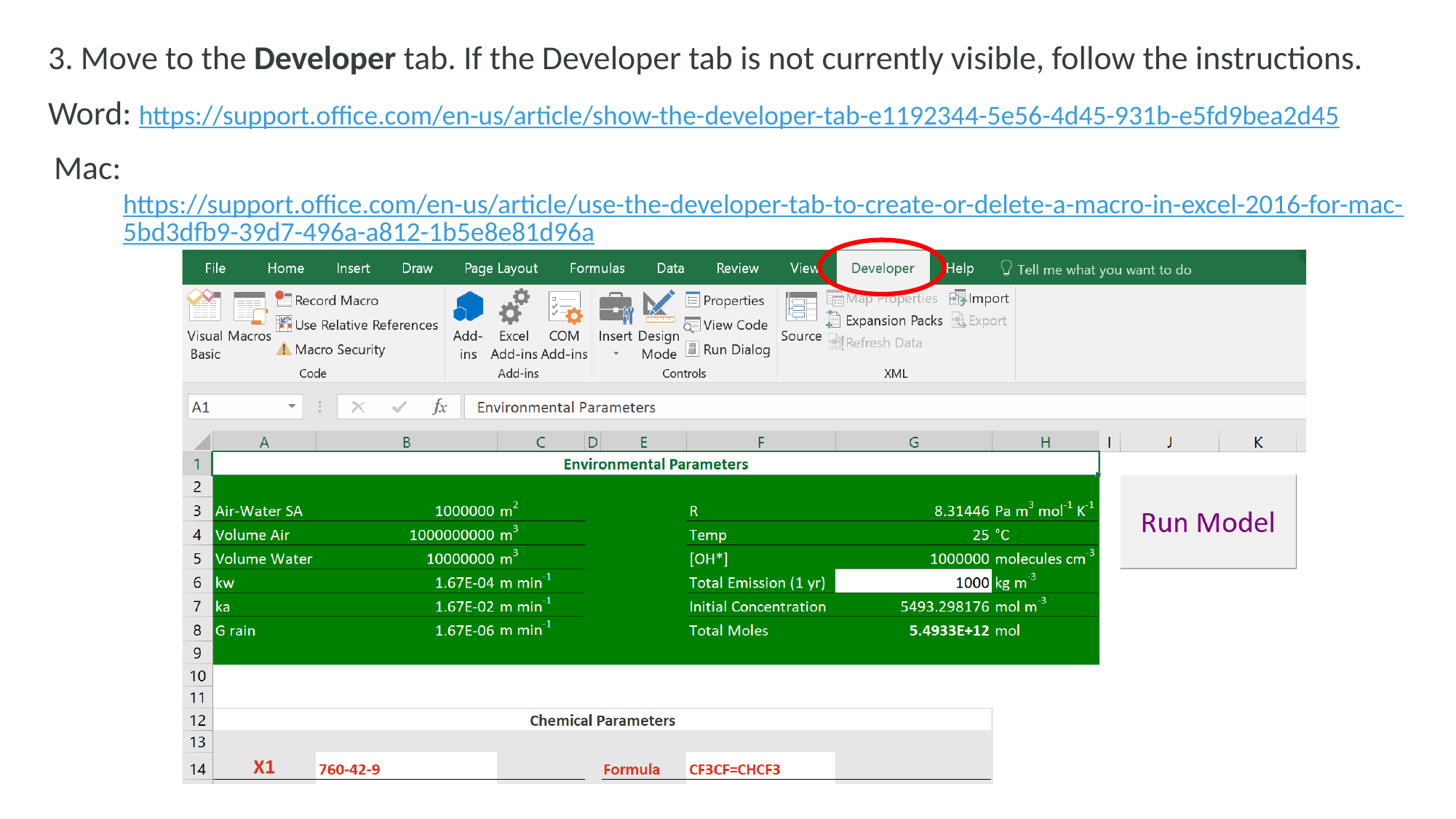

3. Move to the Developer tab. If the Developer tab is not currently visible, follow the instructions.
Word: https://support.office.com/en-us/article/show-the-developer-tab-e1192344-5e56-4d45-931b-e5fd9bea2d45
Mac: https://support.office.com/en-us/article/use-the-developer-tab-to-create-or-delete-a-macro-in-excel-2016-for-mac-5bd3dfb9-39d7-496a-a812-1b5e8e81d96a

## Slide 36
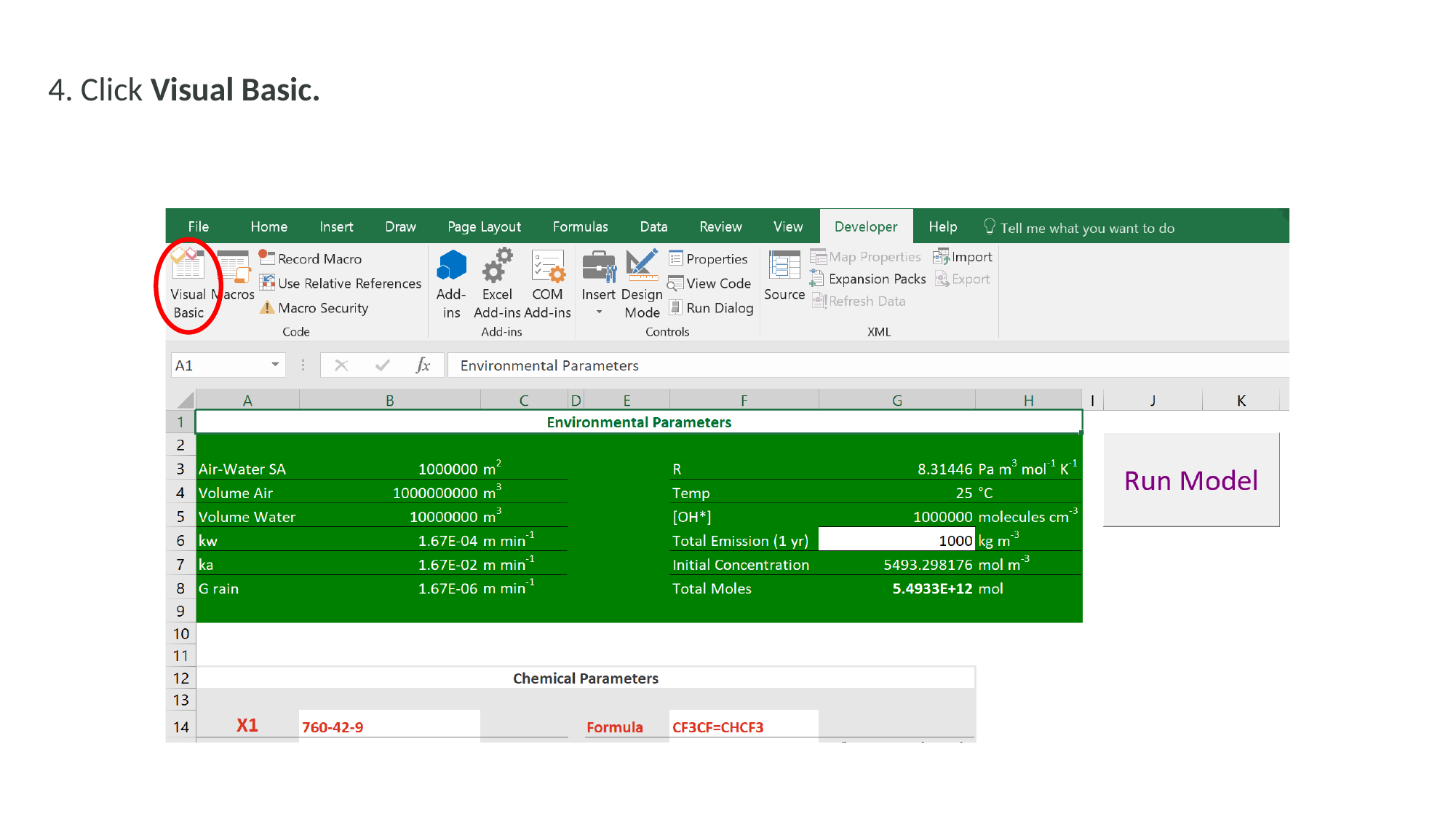

4. Click Visual Basic.

## Slide 37
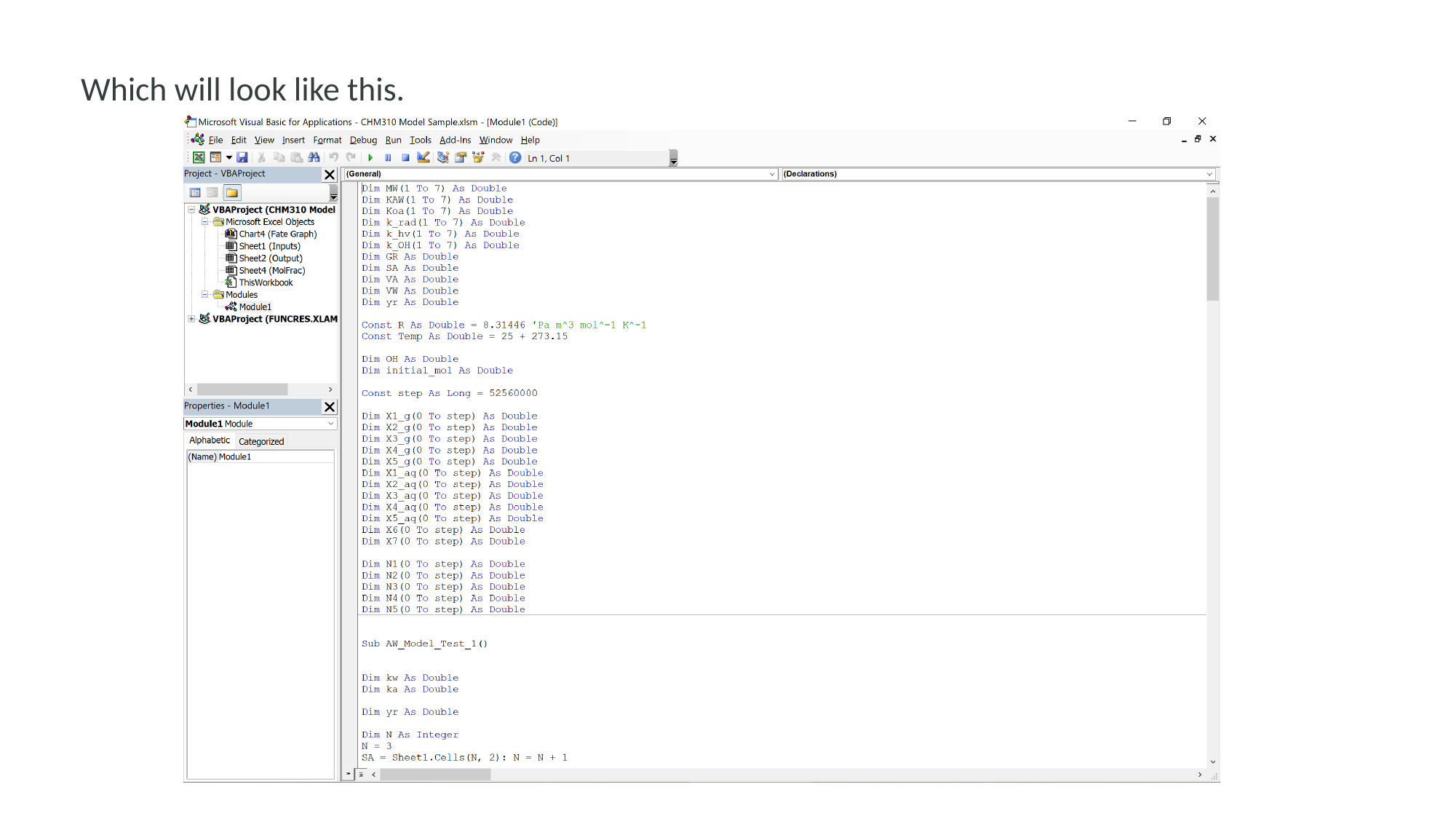

4. Which will look like this.

## Slide 38
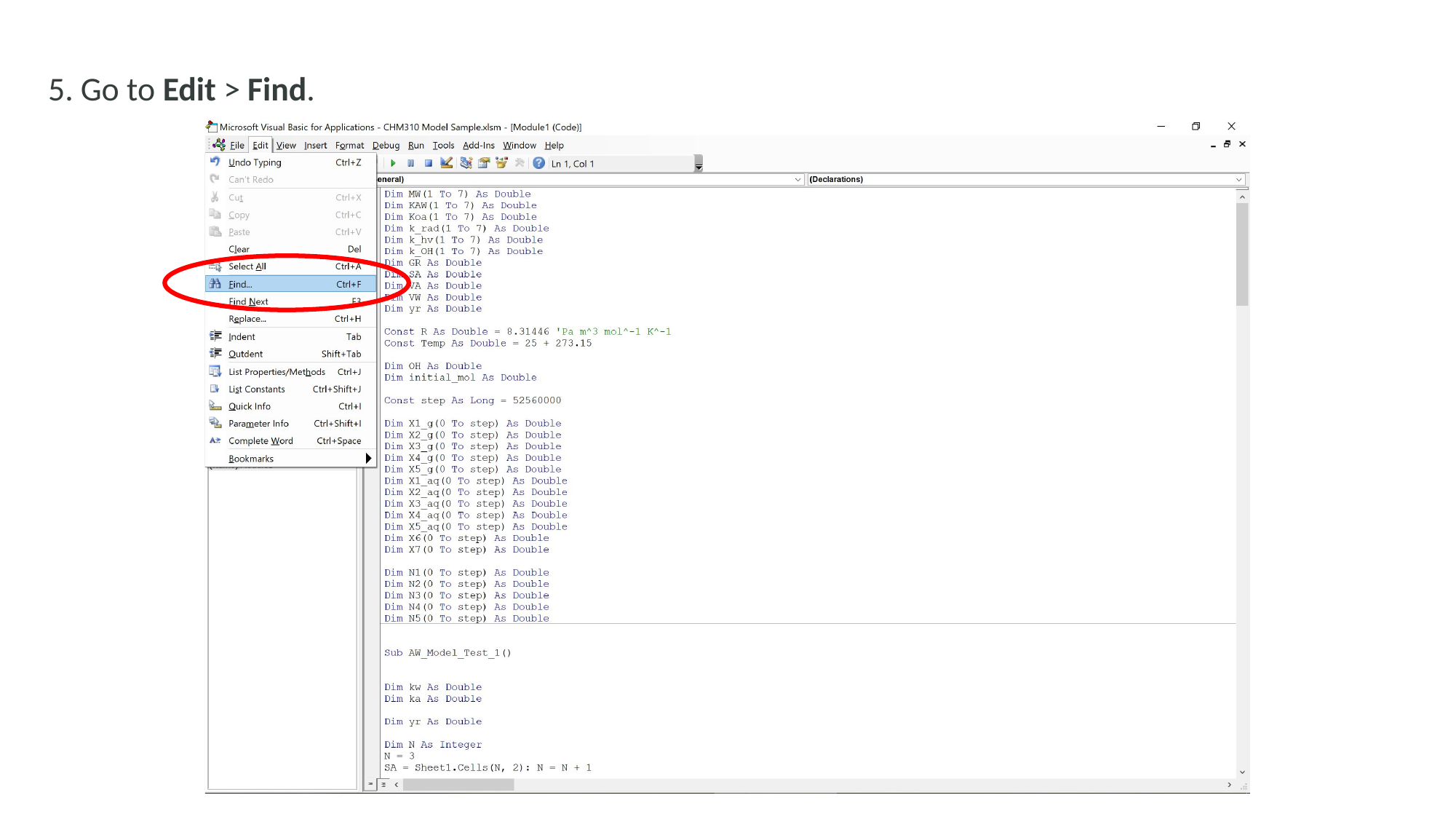

5. Go to Edit > Find.

## Slide 39
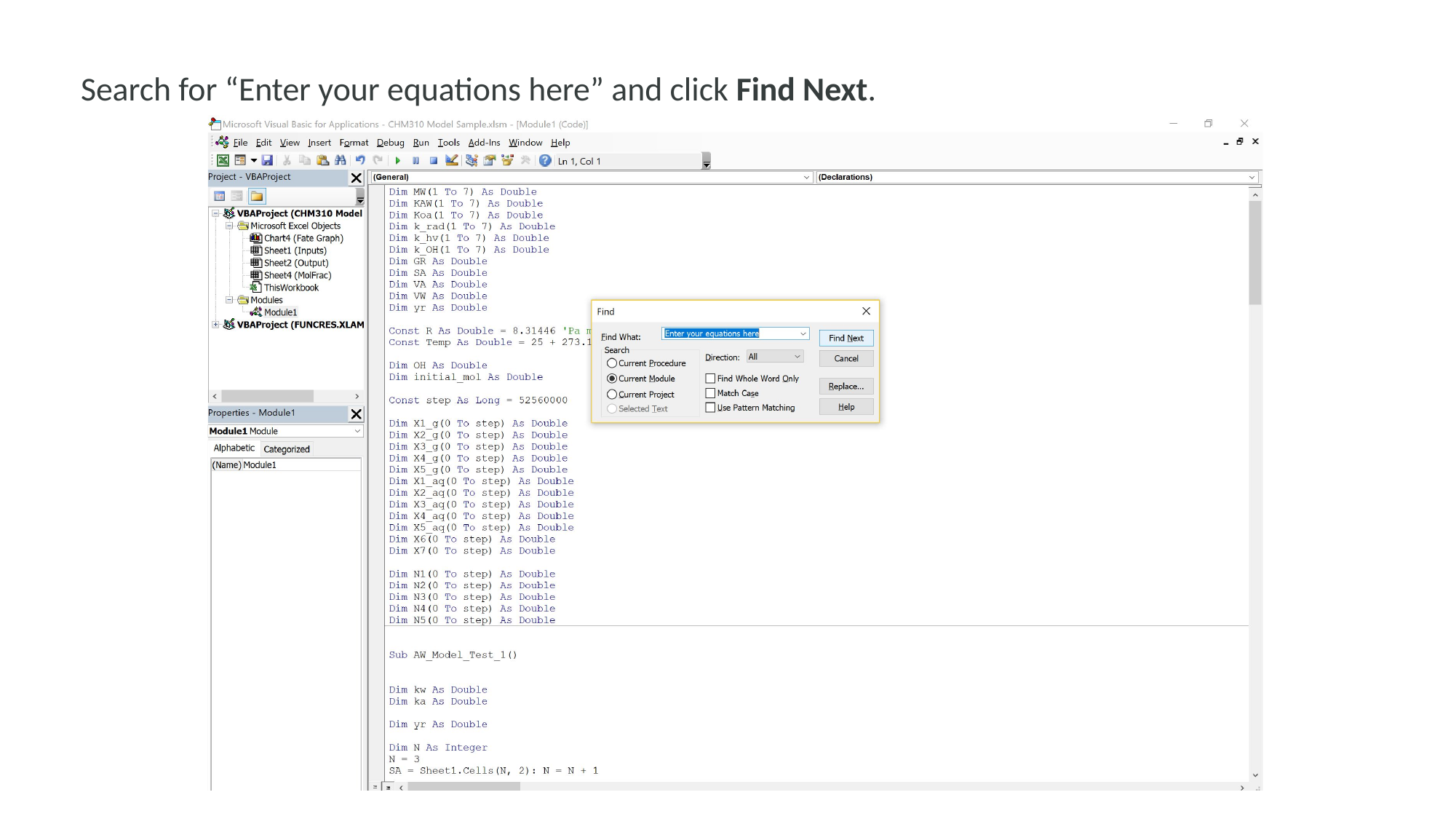

5. Search for “Enter your equations here” and click Find Next.

## Slide 40
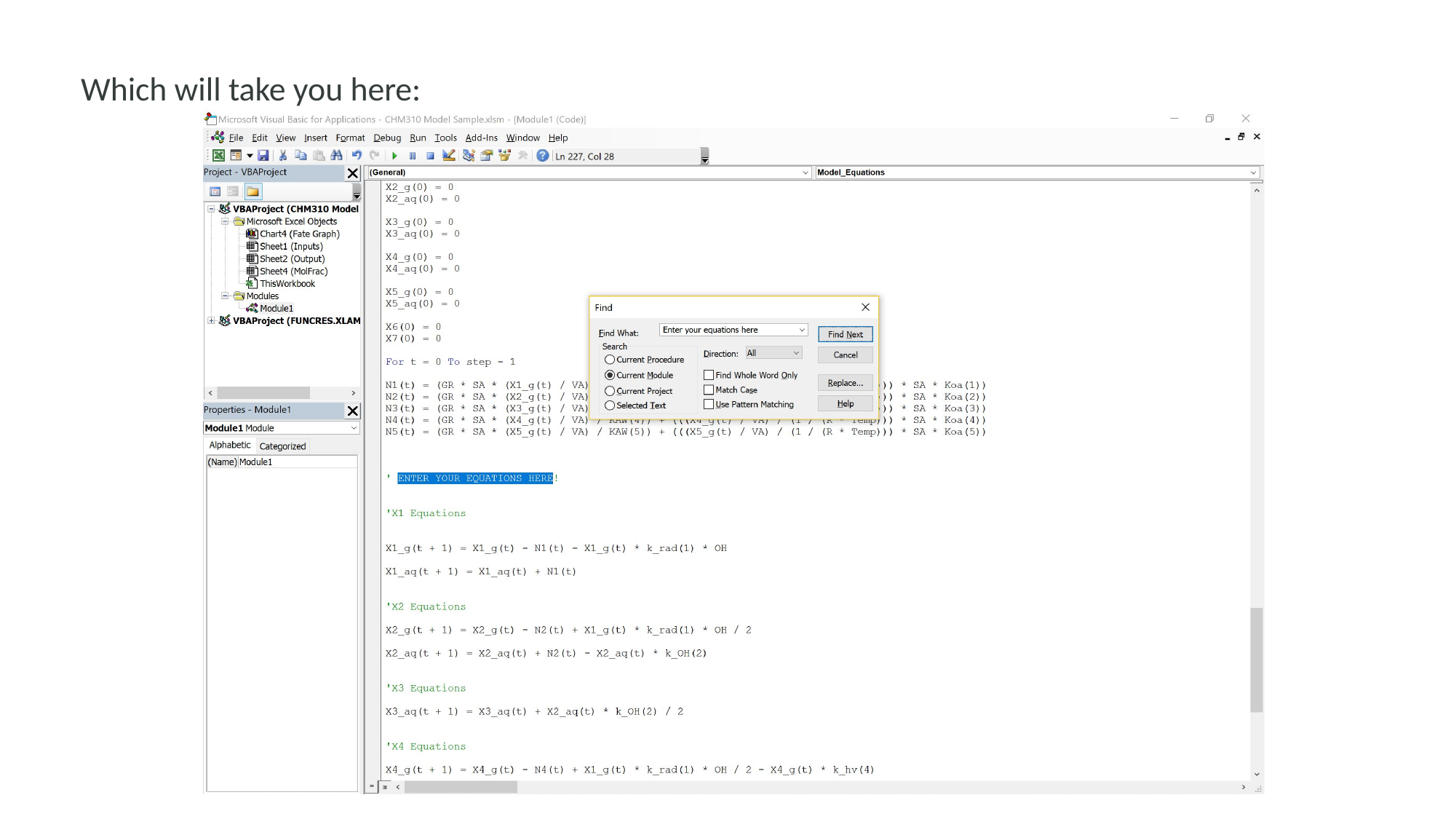

5. Which will take you here:

## Slide 41
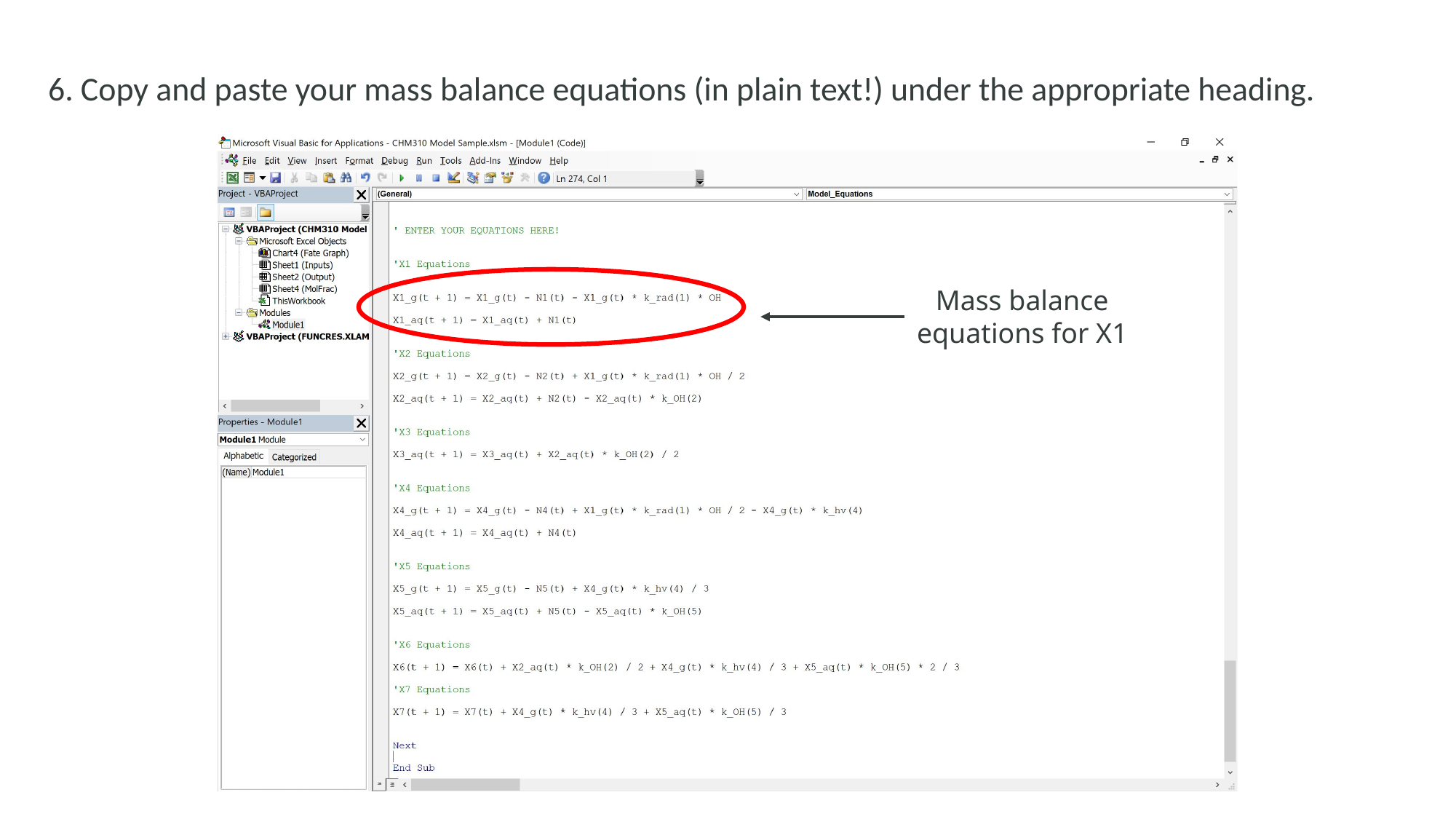

6. Copy and paste your mass balance equations (in plain text!) under the appropriate heading.
Mass balance equations for X1

## Slide 42
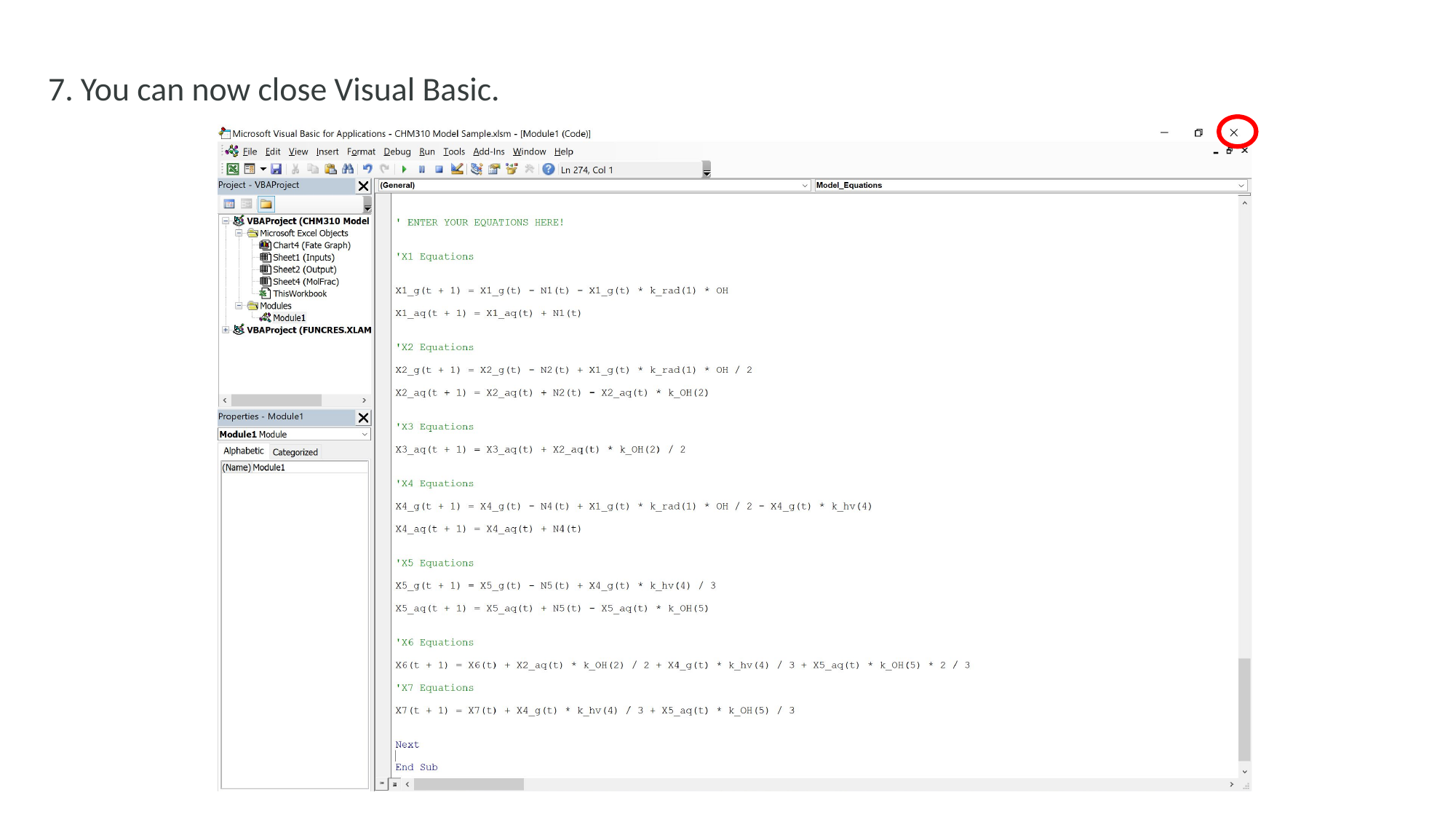

7. You can now close Visual Basic.

## Slide 43
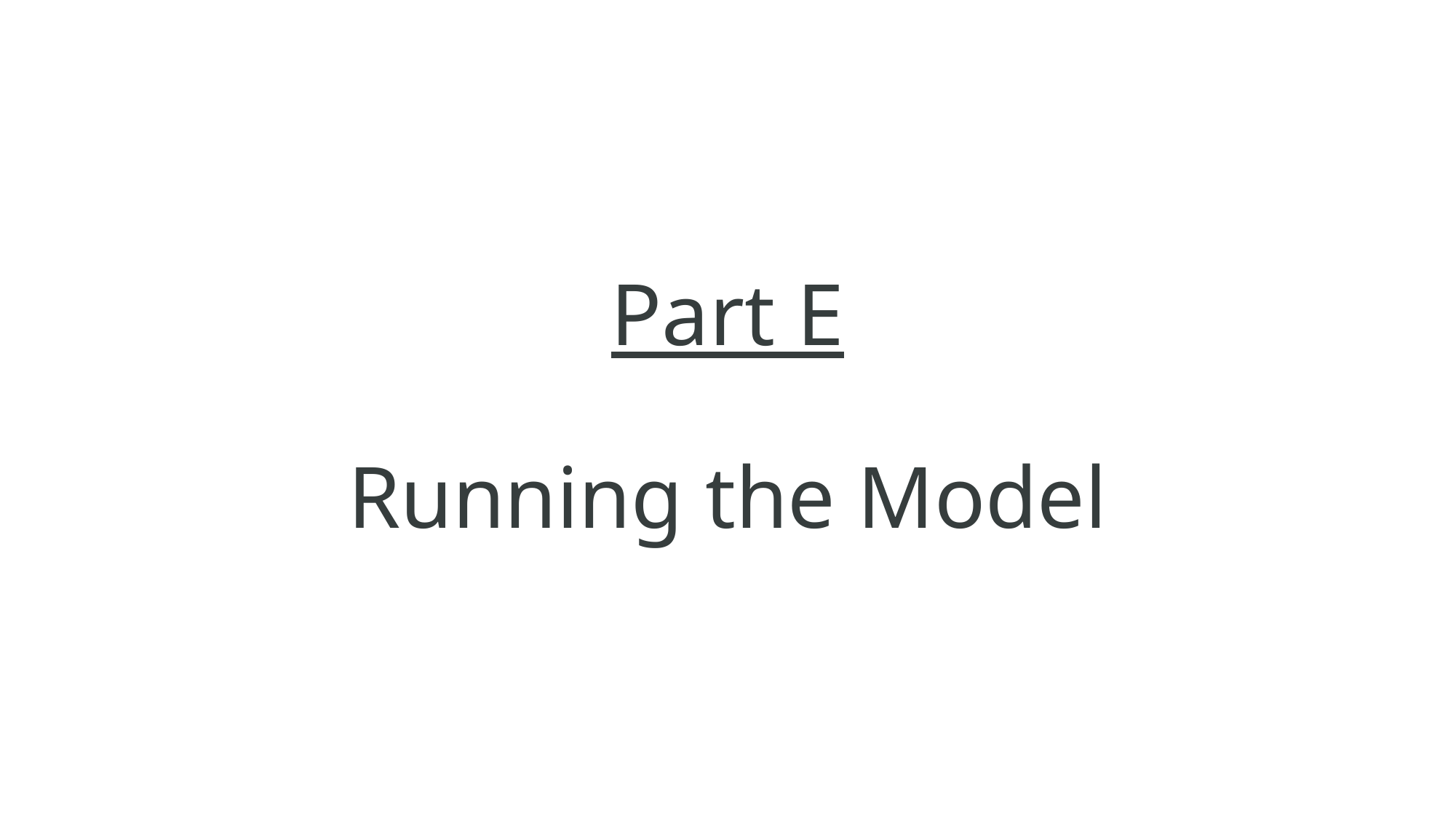

# Part E Running the Model

## Slide 44
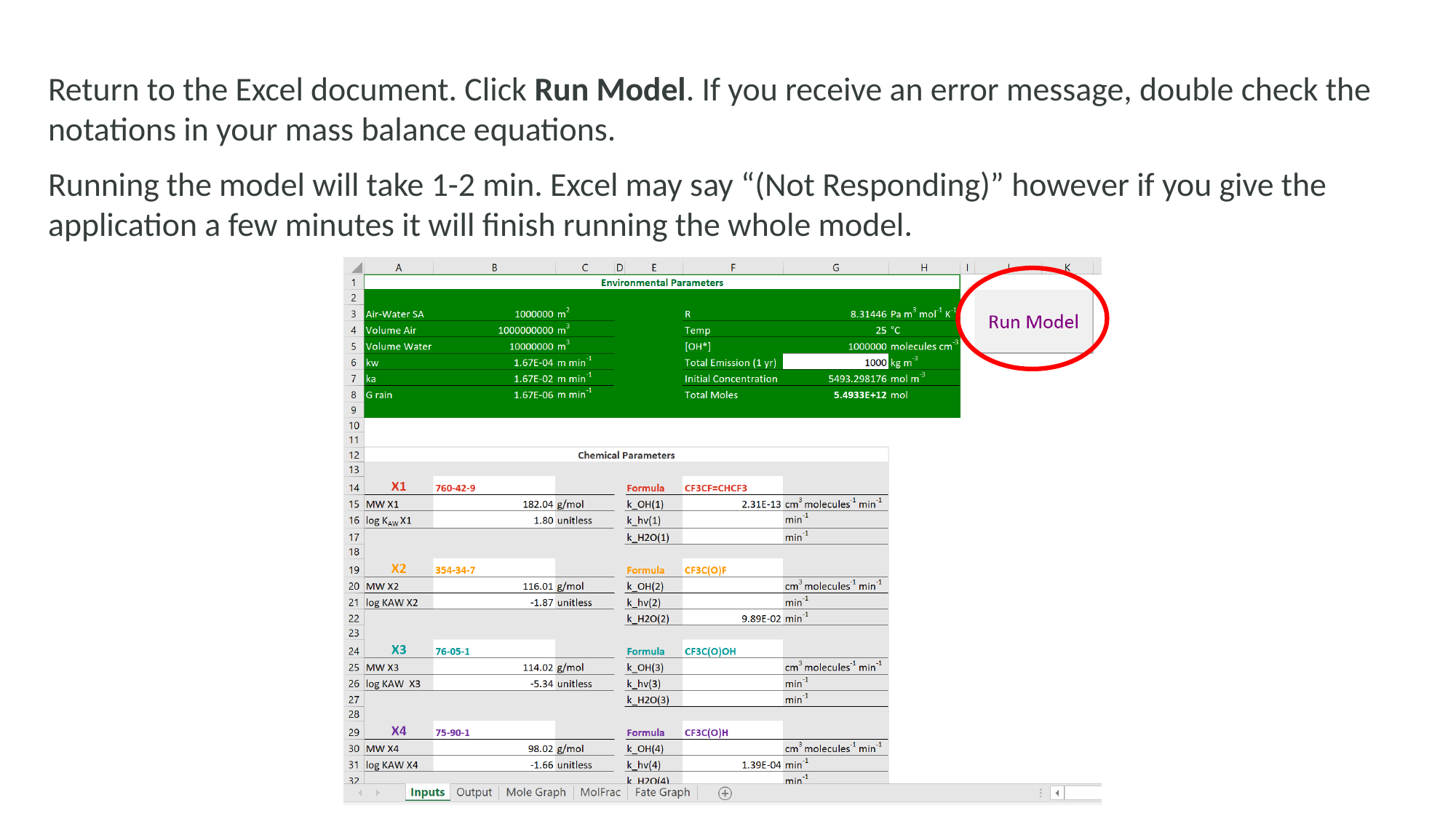

Return to the Excel document. Click Run Model. If you receive an error message, double check the notations in your mass balance equations.
Running the model will take 1-2 min. Excel may say “(Not Responding)” however if you give the application a few minutes it will finish running the whole model.

## Slide 45
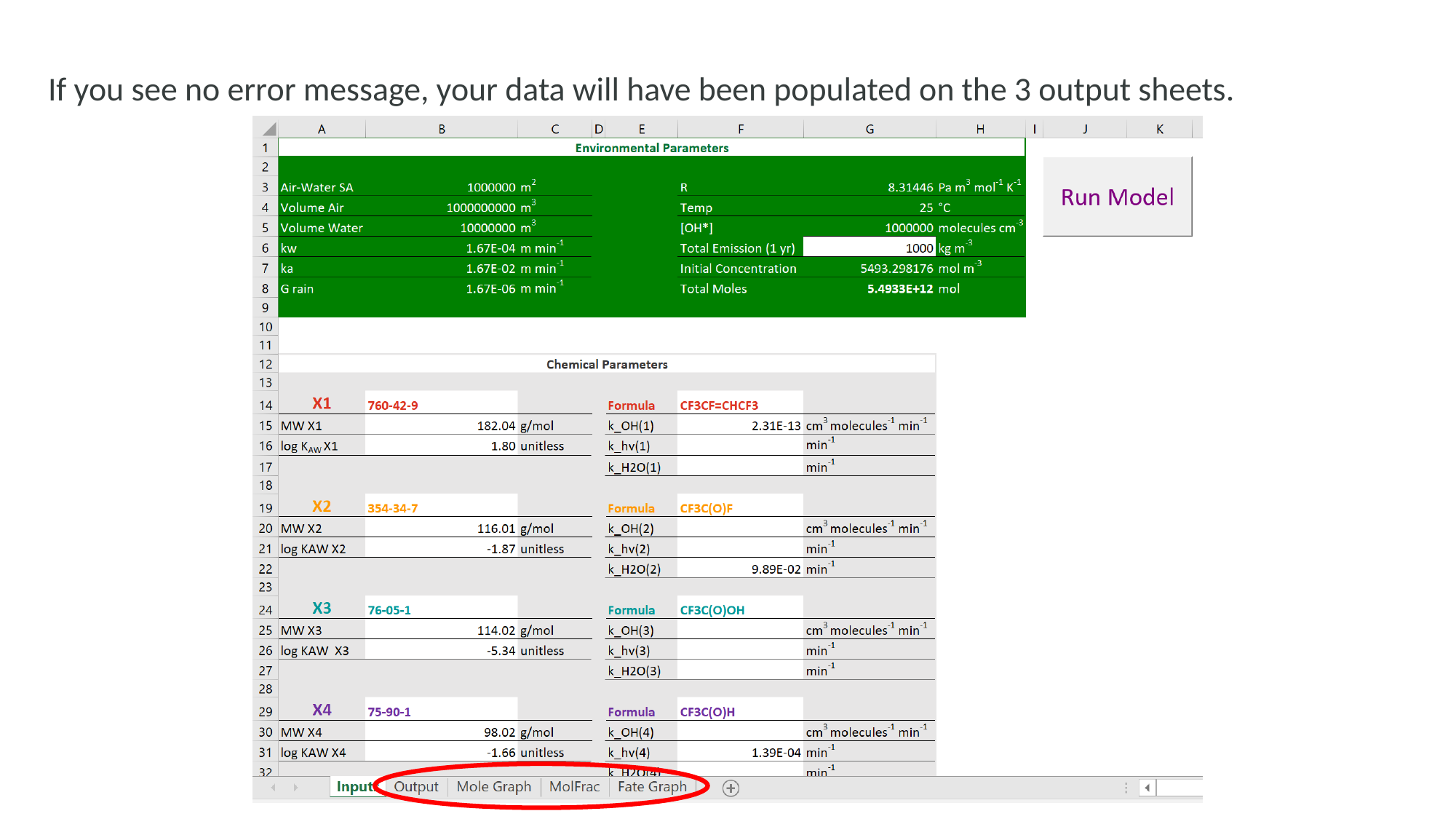

If you see no error message, your data will have been populated on the 3 output sheets.

## Slide 46
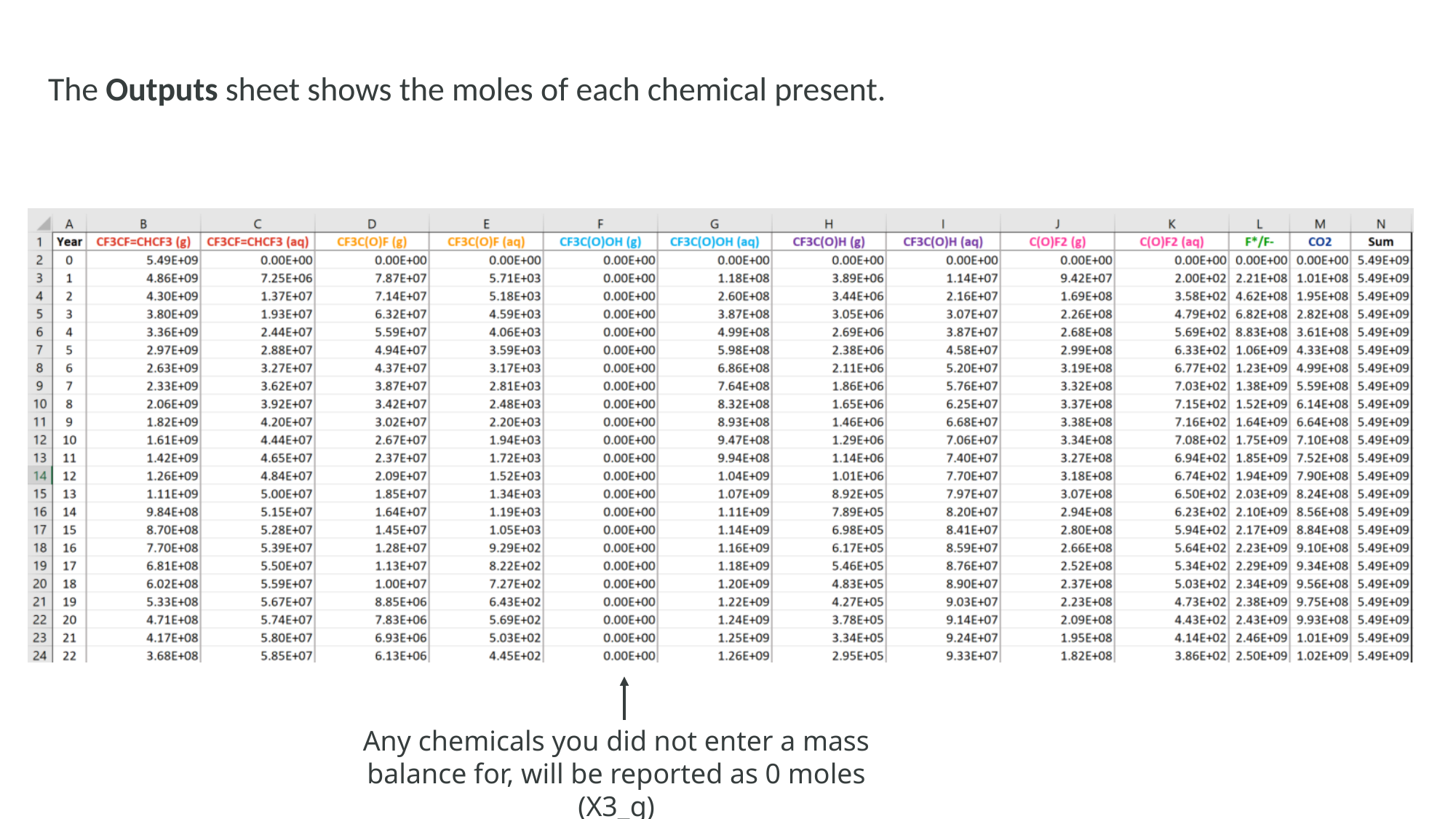

The Outputs sheet shows the moles of each chemical present.
Any chemicals you did not enter a mass balance for, will be reported as 0 moles (X3_g)

## Slide 47
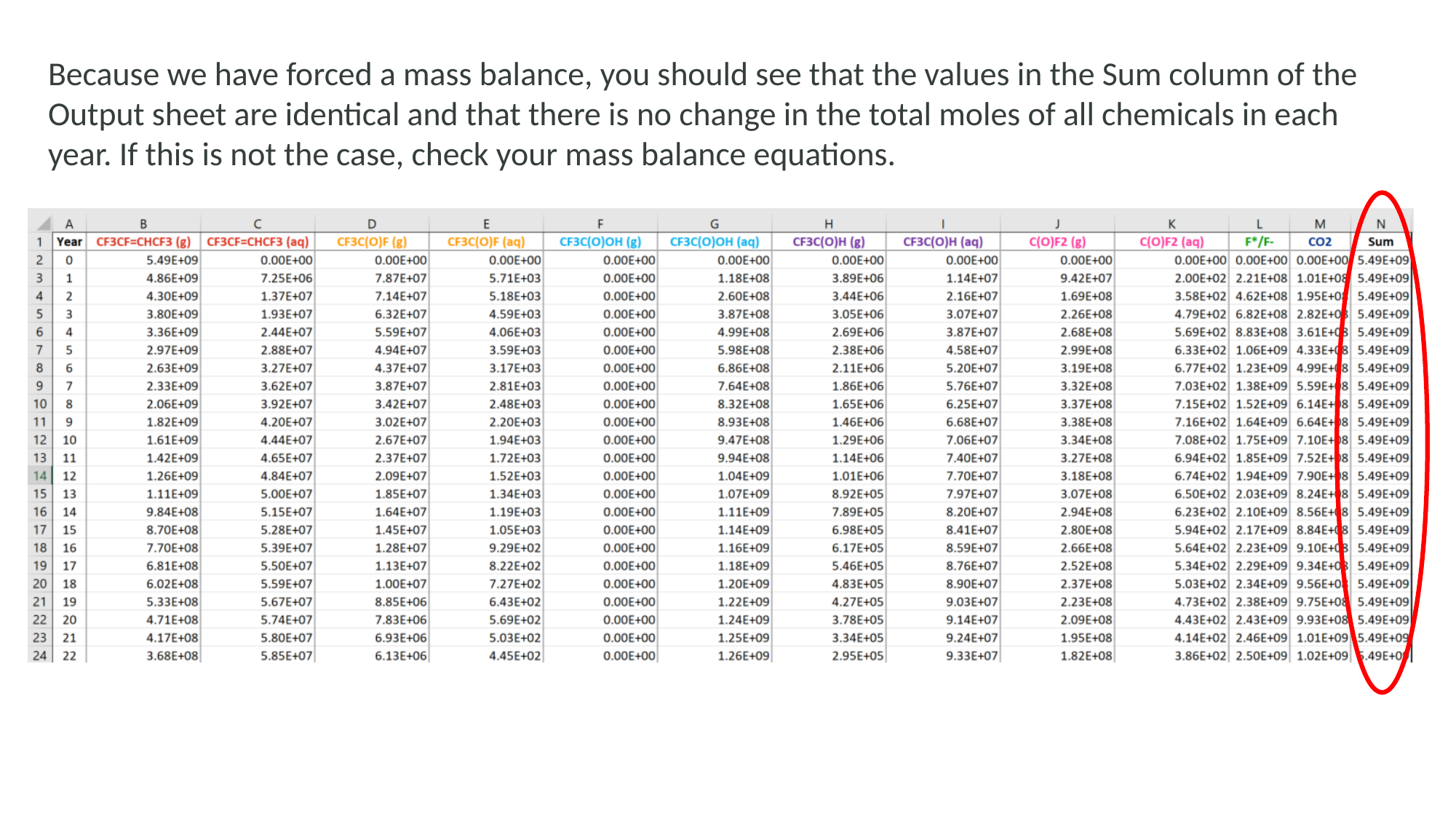

Because we have forced a mass balance, you should see that the values in the Sum column of the Output sheet are identical and that there is no change in the total moles of all chemicals in each year. If this is not the case, check your mass balance equations.

## Slide 48
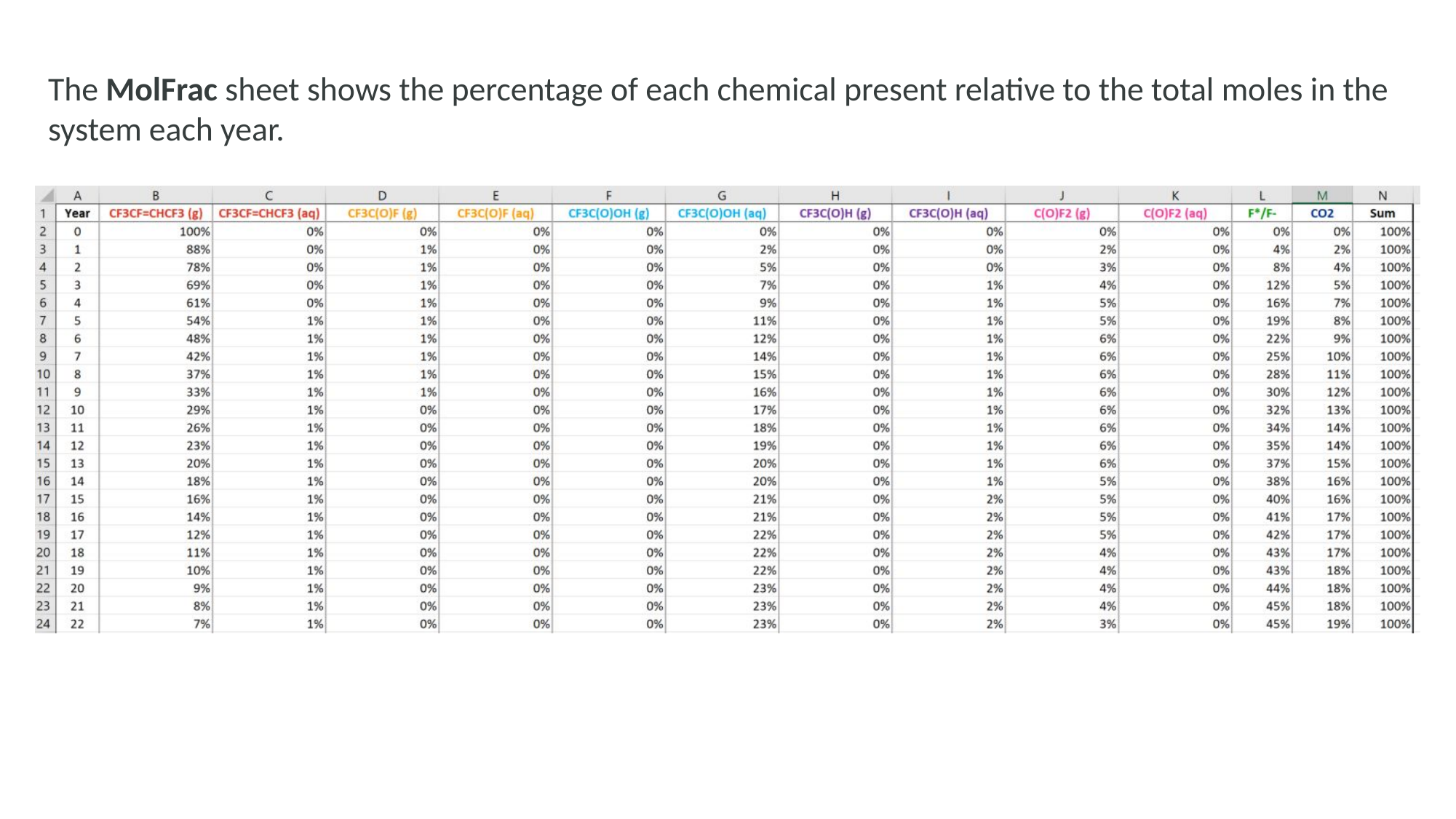

The MolFrac sheet shows the percentage of each chemical present relative to the total moles in the system each year.

## Slide 49
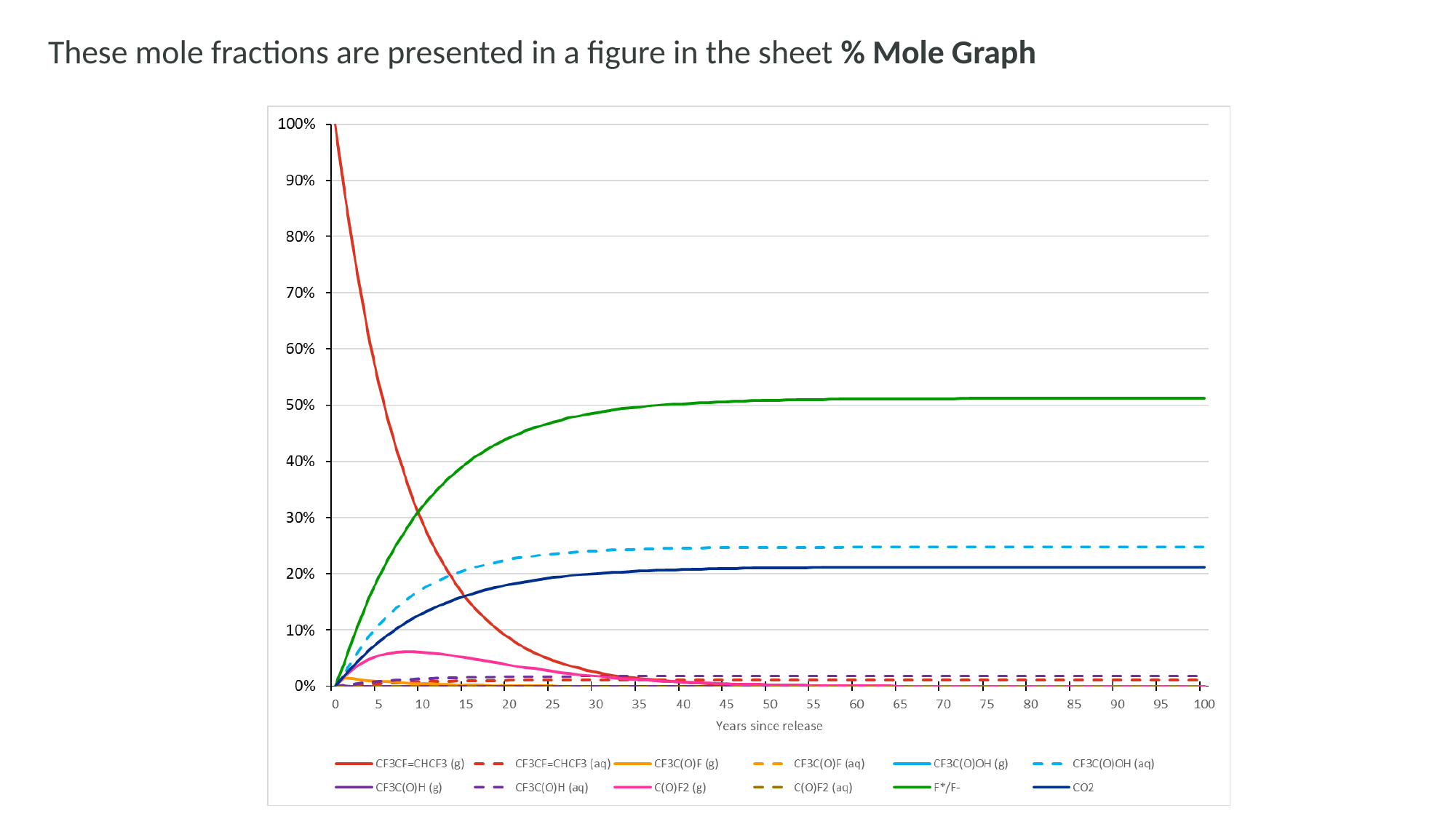

These mole fractions are presented in a figure in the sheet % Mole Graph

## Slide 50
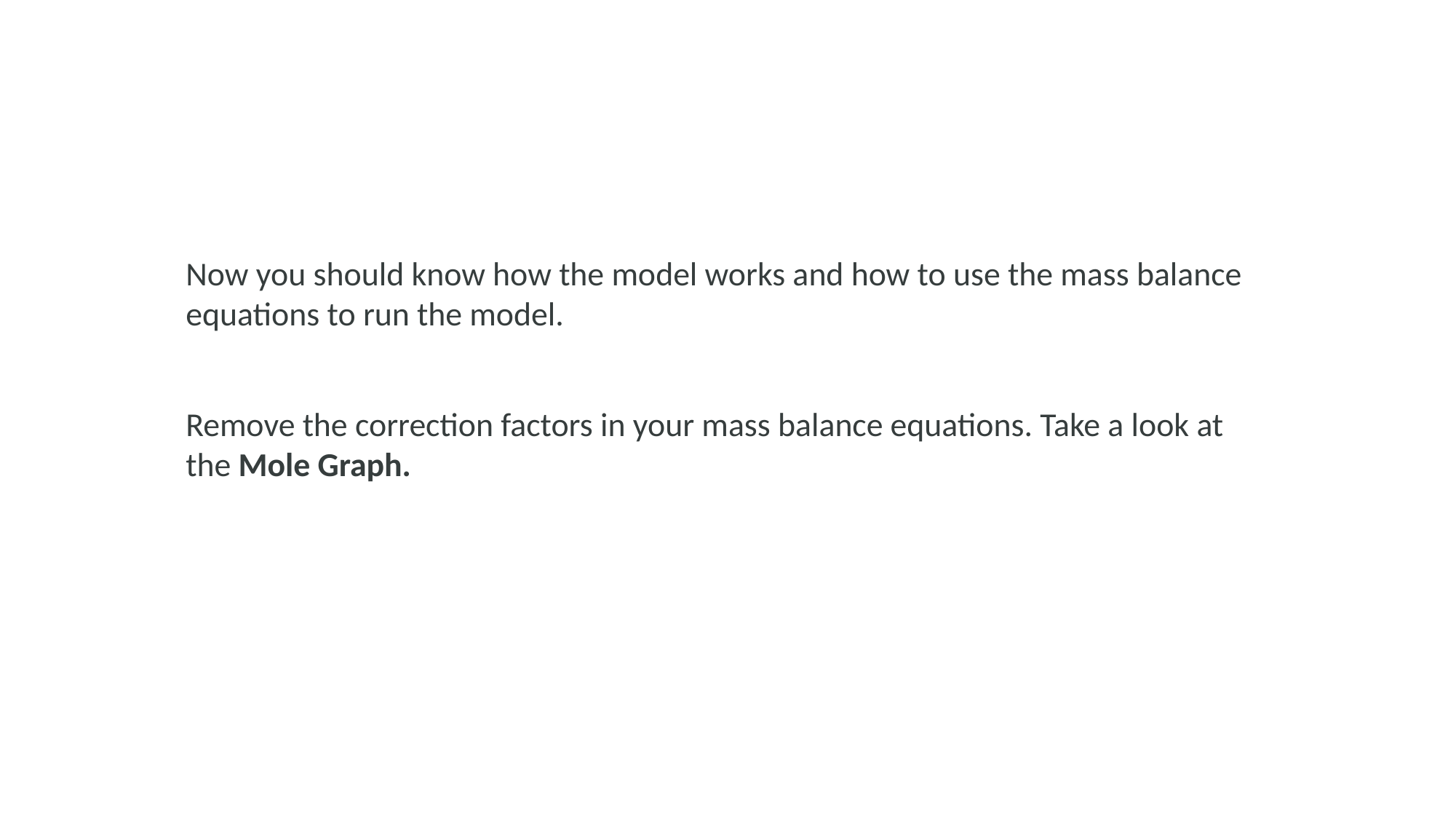

Now you should know how the model works and how to use the mass balance equations to run the model.
Remove the correction factors in your mass balance equations. Take a look at the Mole Graph.

## Slide 51
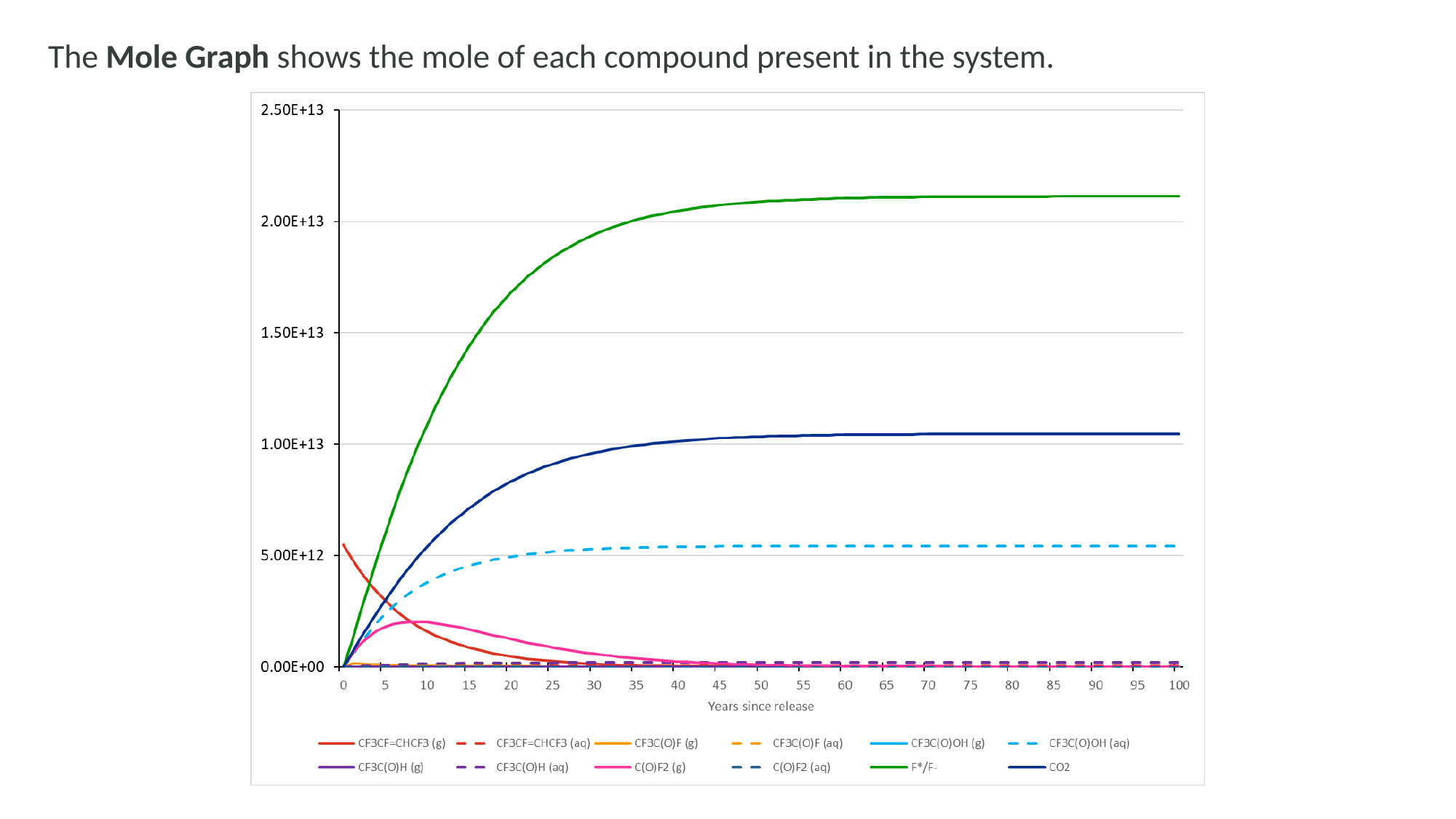

The Mole Graph shows the mole of each compound present in the system.
