# Supplementary material for: Welcome to 310 Environmental Working Group! A Group Project That Places Students in the Role of Consultants Helping Businesses Choose the Most Climate Friendly Fluorinated Gas
Source: J Chem Educ. 2024 Sep 6;101(10):4203–13. doi: 10.1021/acs.jchemed.4c00479 (PMC11465463; doi:10.1021/acs.jchemed.4c00479)
Supplement: Supplementary file 1 — ed4c00479_si_001.zip [file ed4c00479_si_001.zip › Supporting Information/Assignment 4/CHM310_Model_Instructions_R.pptx]

## Slide 1
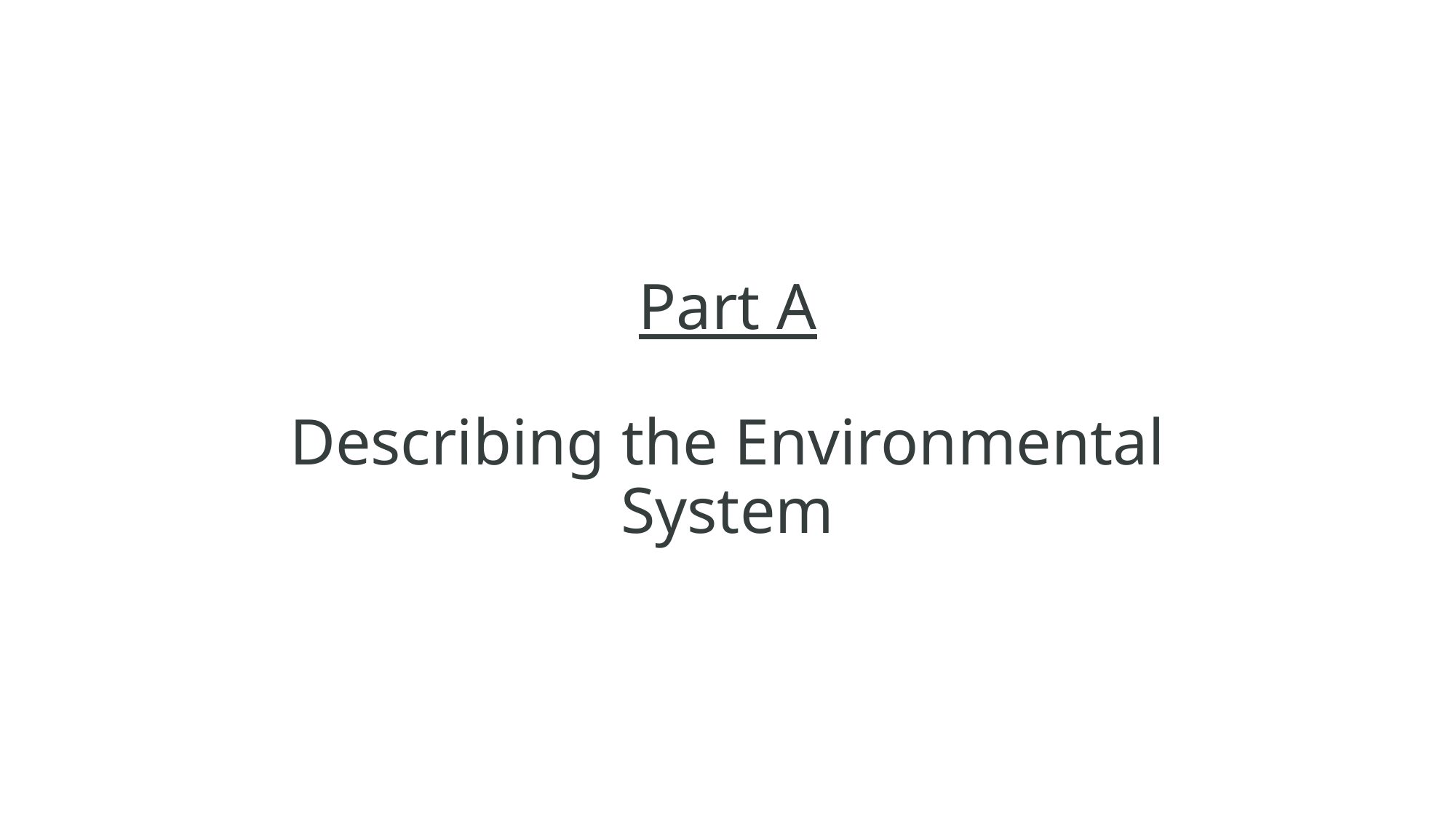

# Part A Describing the Environmental System

## Slide 2
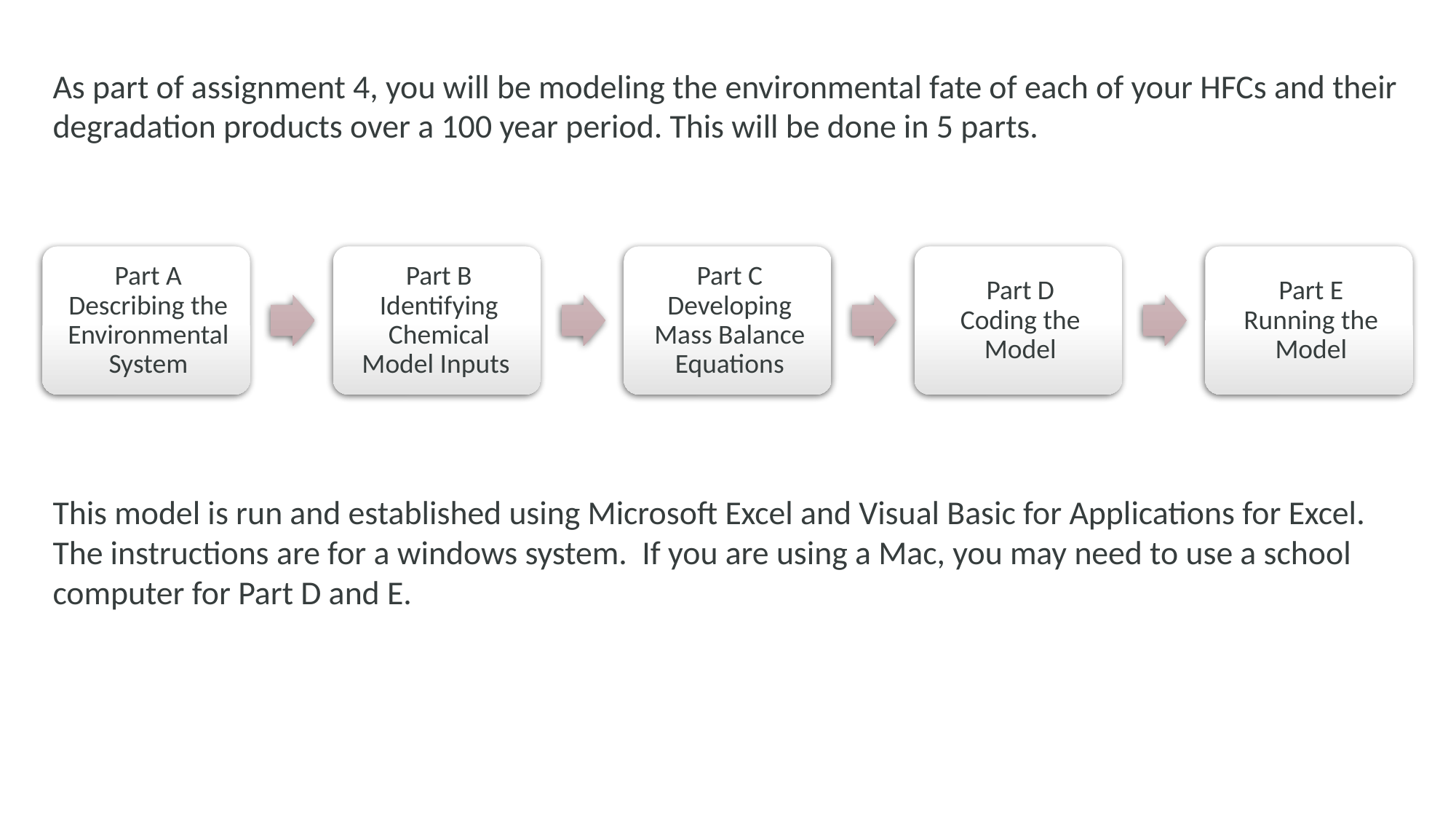

As part of assignment 4, you will be modeling the environmental fate of each of your HFCs and their degradation products over a 100 year period. This will be done in 5 parts.
This model is run and established using Microsoft Excel and Visual Basic for Applications for Excel. The instructions are for a windows system. If you are using a Mac, you may need to use a school computer for Part D and E.

## Slide 3
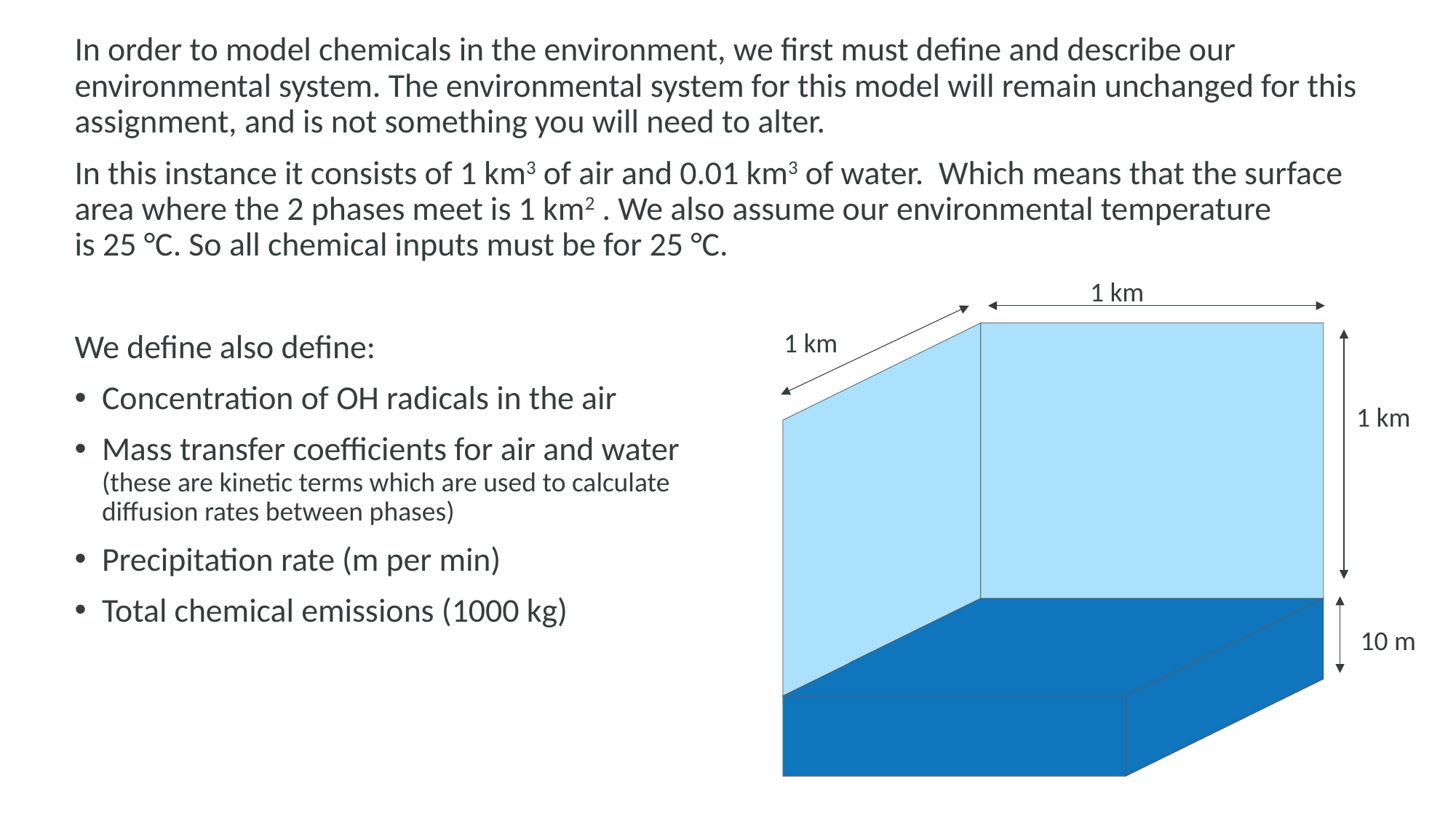

In order to model chemicals in the environment, we first must define and describe our environmental system. The environmental system for this model will remain unchanged for this assignment, and is not something you will need to alter.
In this instance it consists of 1 km3 of air and 0.01 km3 of water. Which means that the surface area where the 2 phases meet is 1 km2 . We also assume our environmental temperature
is 25 °C. So all chemical inputs must be for 25 °C.
We define also define:
Concentration of OH radicals in the air
Mass transfer coefficients for air and water
(these are kinetic terms which are used to calculate
diffusion rates between phases)
Precipitation rate (m per min)
Total chemical emissions (1000 kg)
1 km
1 km
1 km
10 m

## Slide 4
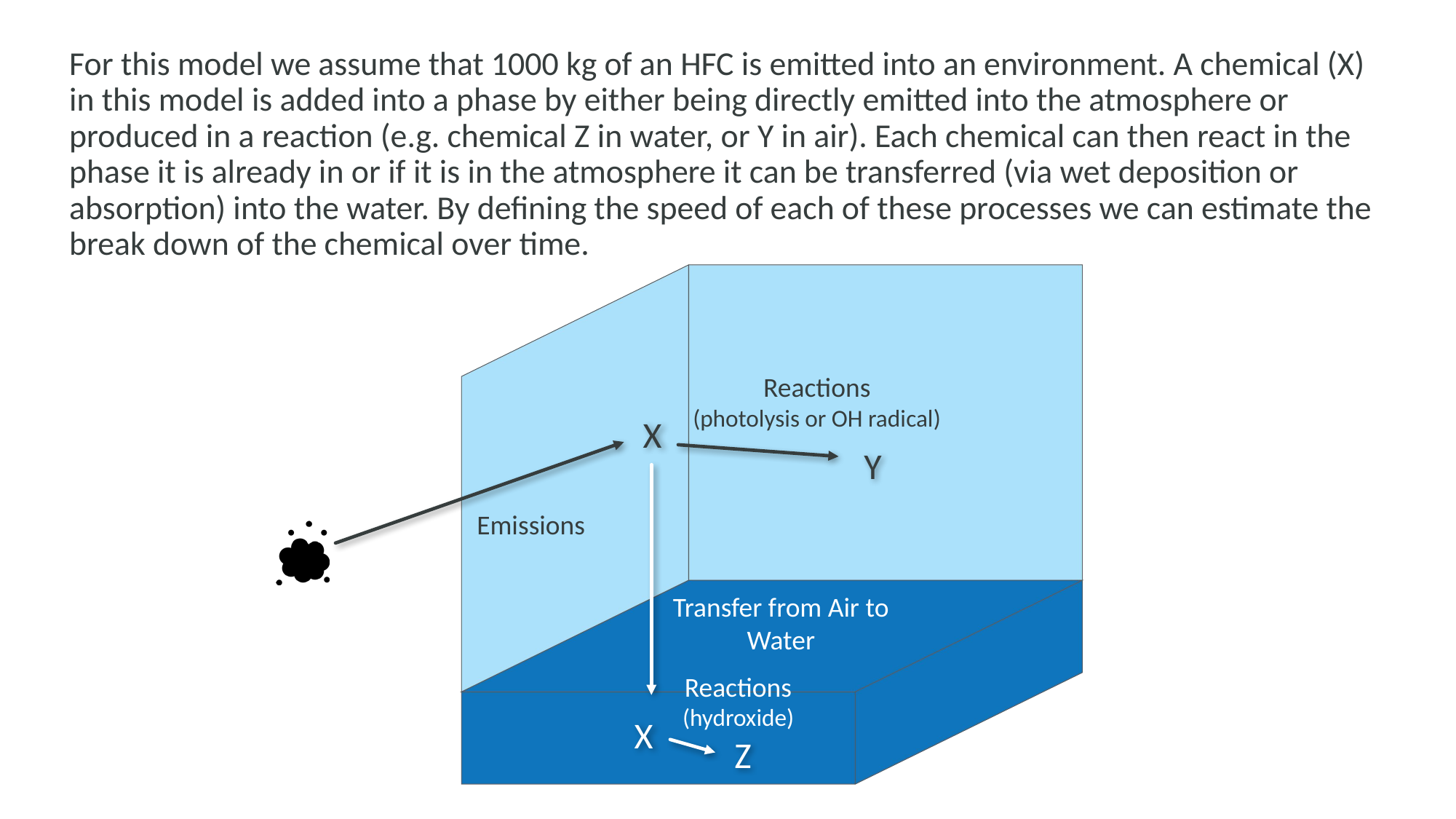

For this model we assume that 1000 kg of an HFC is emitted into an environment. A chemical (X) in this model is added into a phase by either being directly emitted into the atmosphere or produced in a reaction (e.g. chemical Z in water, or Y in air). Each chemical can then react in the phase it is already in or if it is in the atmosphere it can be transferred (via wet deposition or absorption) into the water. By defining the speed of each of these processes we can estimate the break down of the chemical over time.
Reactions
(photolysis or OH radical)
X
Y
Emissions
Transfer from Air to Water
Reactions (hydroxide)
X
Z

## Slide 5
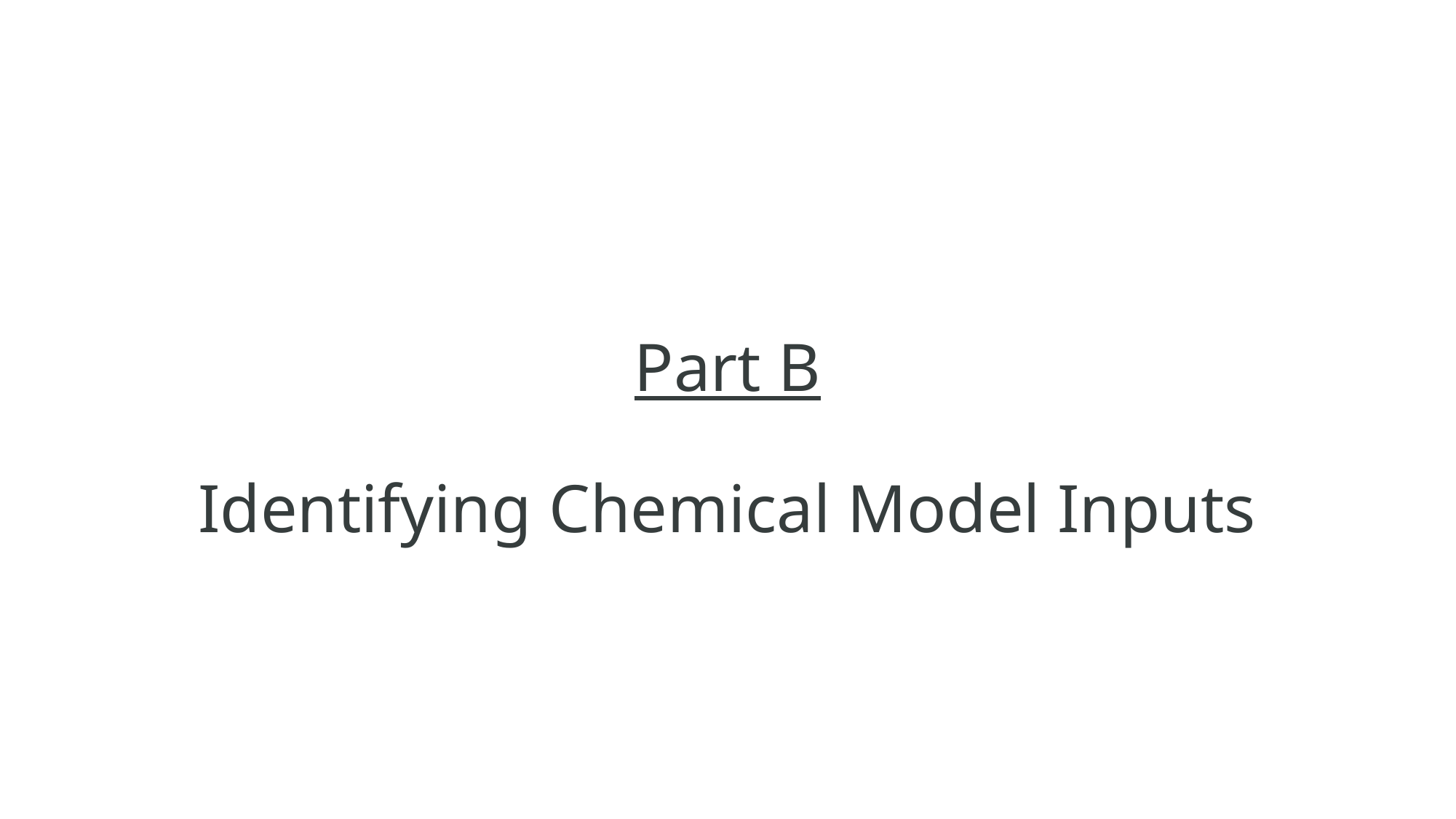

# Part B Identifying Chemical Model Inputs

## Slide 6
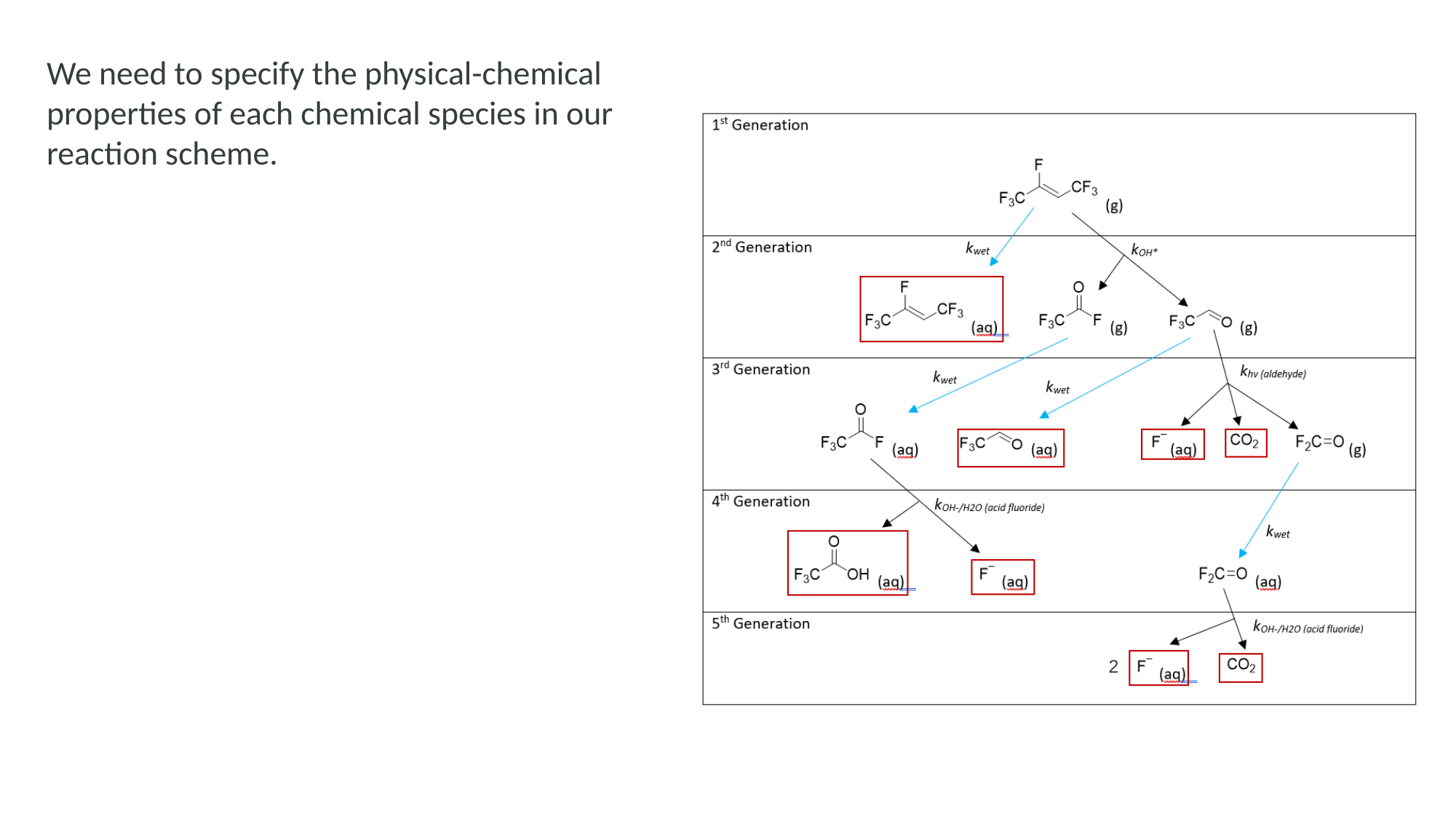

We need to specify the physical-chemical properties of each chemical species in our reaction scheme.
2

## Slide 7
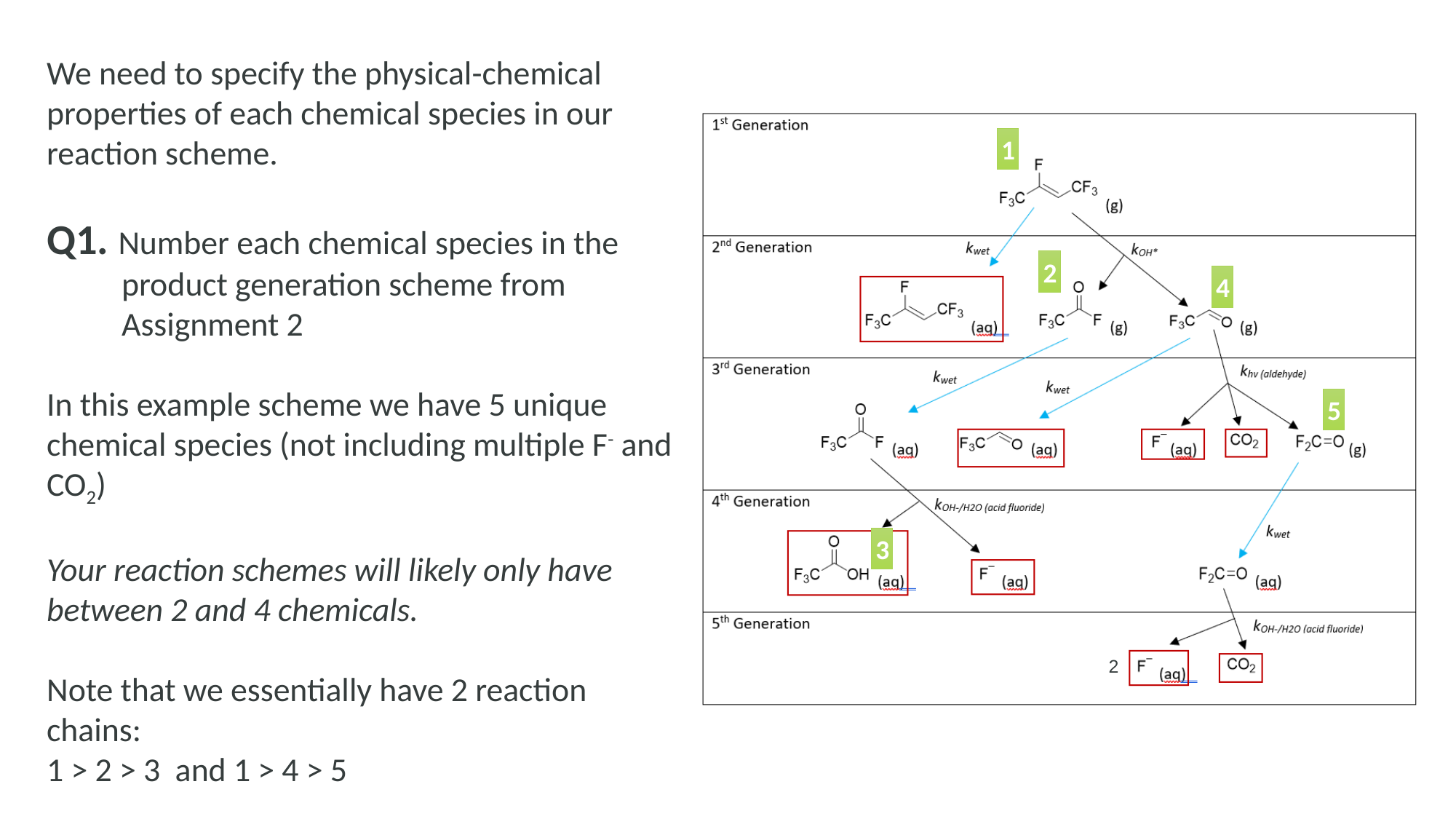

We need to specify the physical-chemical properties of each chemical species in our reaction scheme.
Q1. Number each chemical species in the product generation scheme from Assignment 2
In this example scheme we have 5 unique chemical species (not including multiple F- and CO2)
Your reaction schemes will likely only have between 2 and 4 chemicals.
Note that we essentially have 2 reaction chains:
1 > 2 > 3 and 1 > 4 > 5
1
2
4
5
3
2

## Slide 8
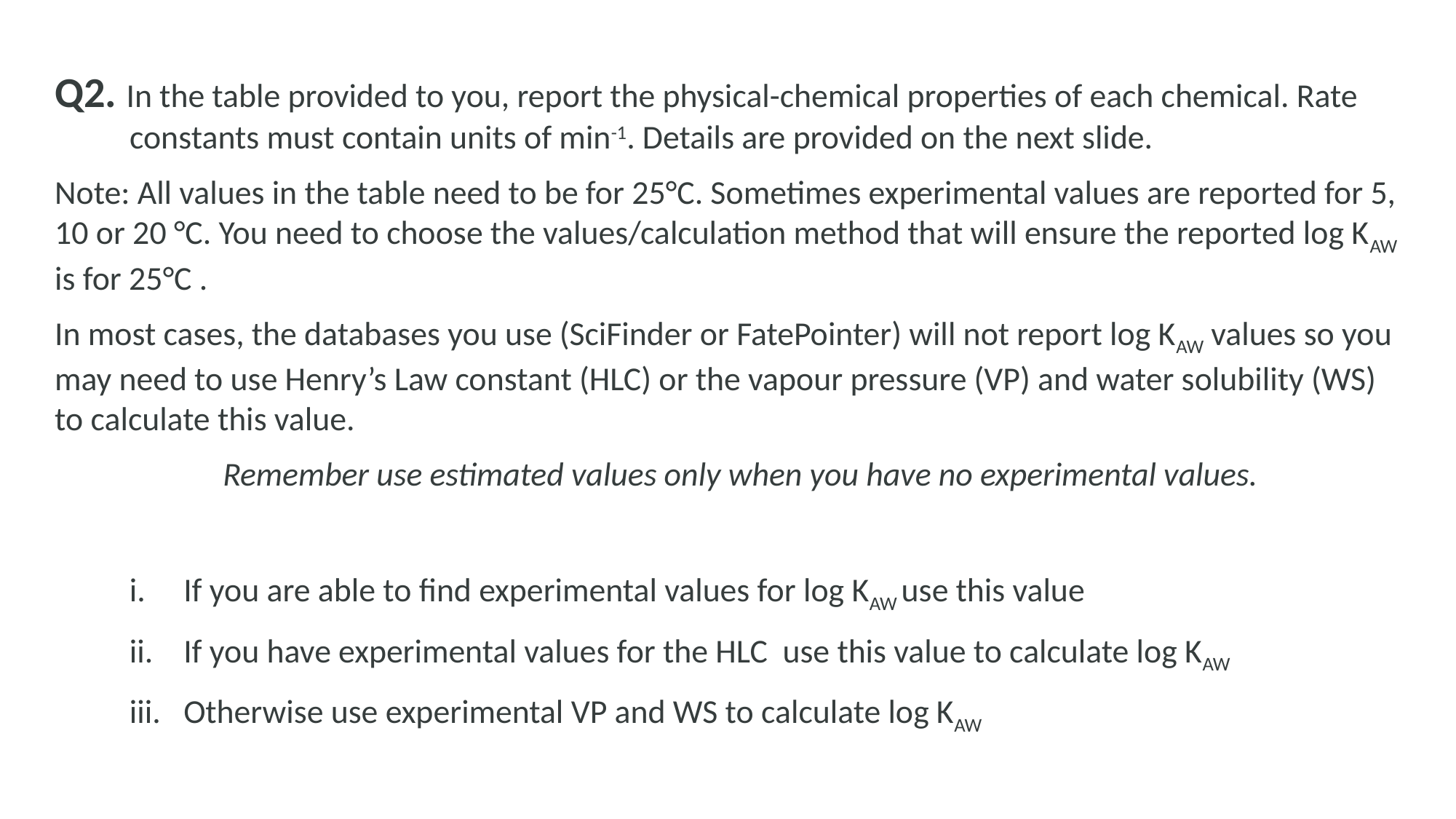

Q2. In the table provided to you, report the physical-chemical properties of each chemical. Rate constants must contain units of min-1. Details are provided on the next slide.
Note: All values in the table need to be for 25°C. Sometimes experimental values are reported for 5, 10 or 20 °C. You need to choose the values/calculation method that will ensure the reported log KAW is for 25°C .
In most cases, the databases you use (SciFinder or FatePointer) will not report log KAW values so you may need to use Henry’s Law constant (HLC) or the vapour pressure (VP) and water solubility (WS) to calculate this value.
Remember use estimated values only when you have no experimental values.
If you are able to find experimental values for log KAW use this value
If you have experimental values for the HLC use this value to calculate log KAW
Otherwise use experimental VP and WS to calculate log KAW

## Slide 9
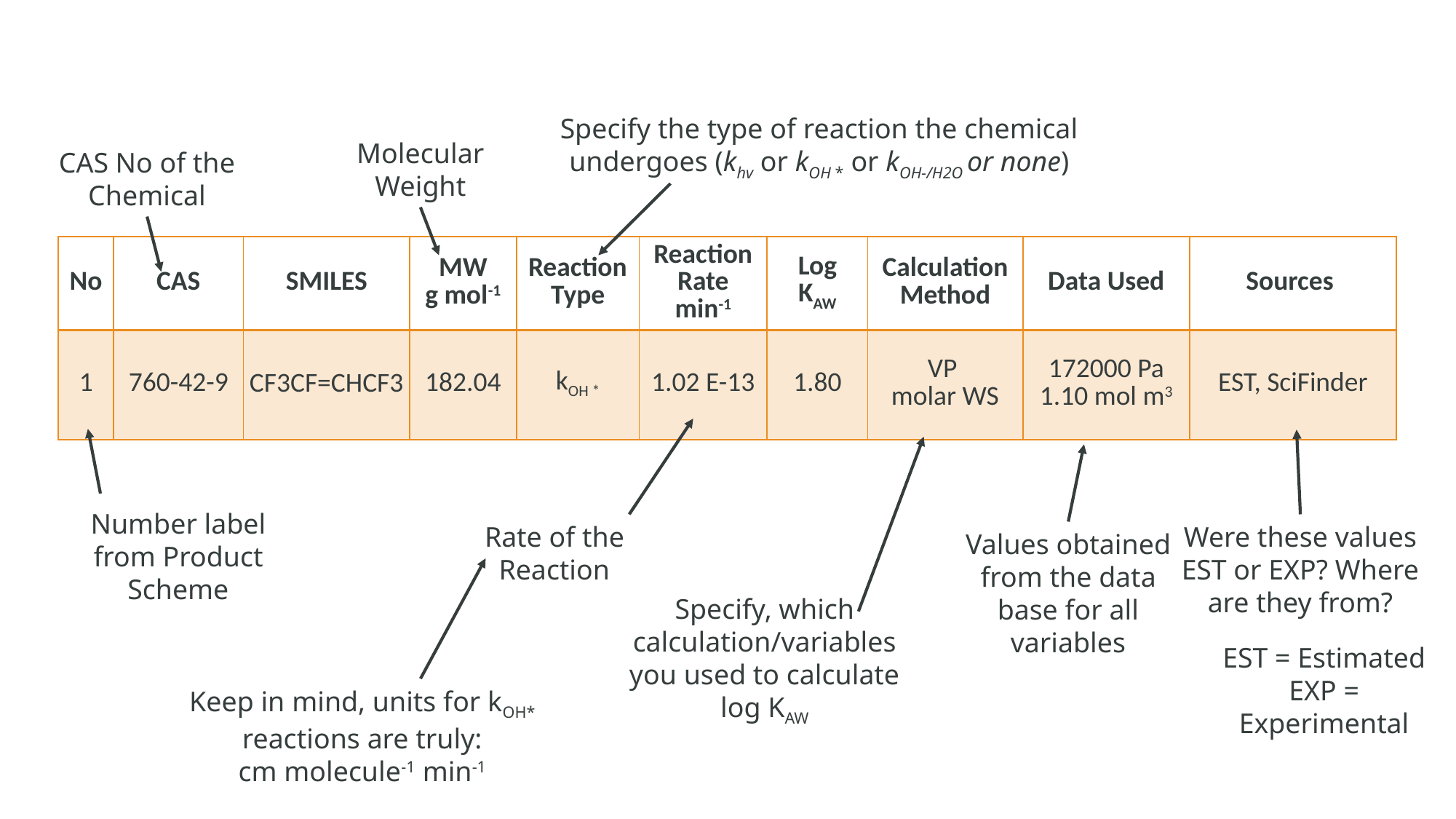

Specify the type of reaction the chemical undergoes (khv or kOH * or kOH-/H2O or none)
Molecular Weight
CAS No of the Chemical
| No | CAS | SMILES | MW g mol-1 | Reaction Type | Reaction Rate min-1 | Log KAW | Calculation Method | Data Used | Sources |
| --- | --- | --- | --- | --- | --- | --- | --- | --- | --- |
| 1 | 760-42-9 | CF3CF=CHCF3 | 182.04 | kOH \* | 1.02 E-13 | 1.80 | VP molar WS | 172000 Pa 1.10 mol m3 | EST, SciFinder |
Number label from Product Scheme
Rate of the Reaction
Were these values EST or EXP? Where are they from?
Values obtained from the data base for all variables
Specify, which calculation/variables you used to calculate log KAW
EST = Estimated
EXP = Experimental
Keep in mind, units for kOH* reactions are truly:
cm molecule-1 min-1

## Slide 10
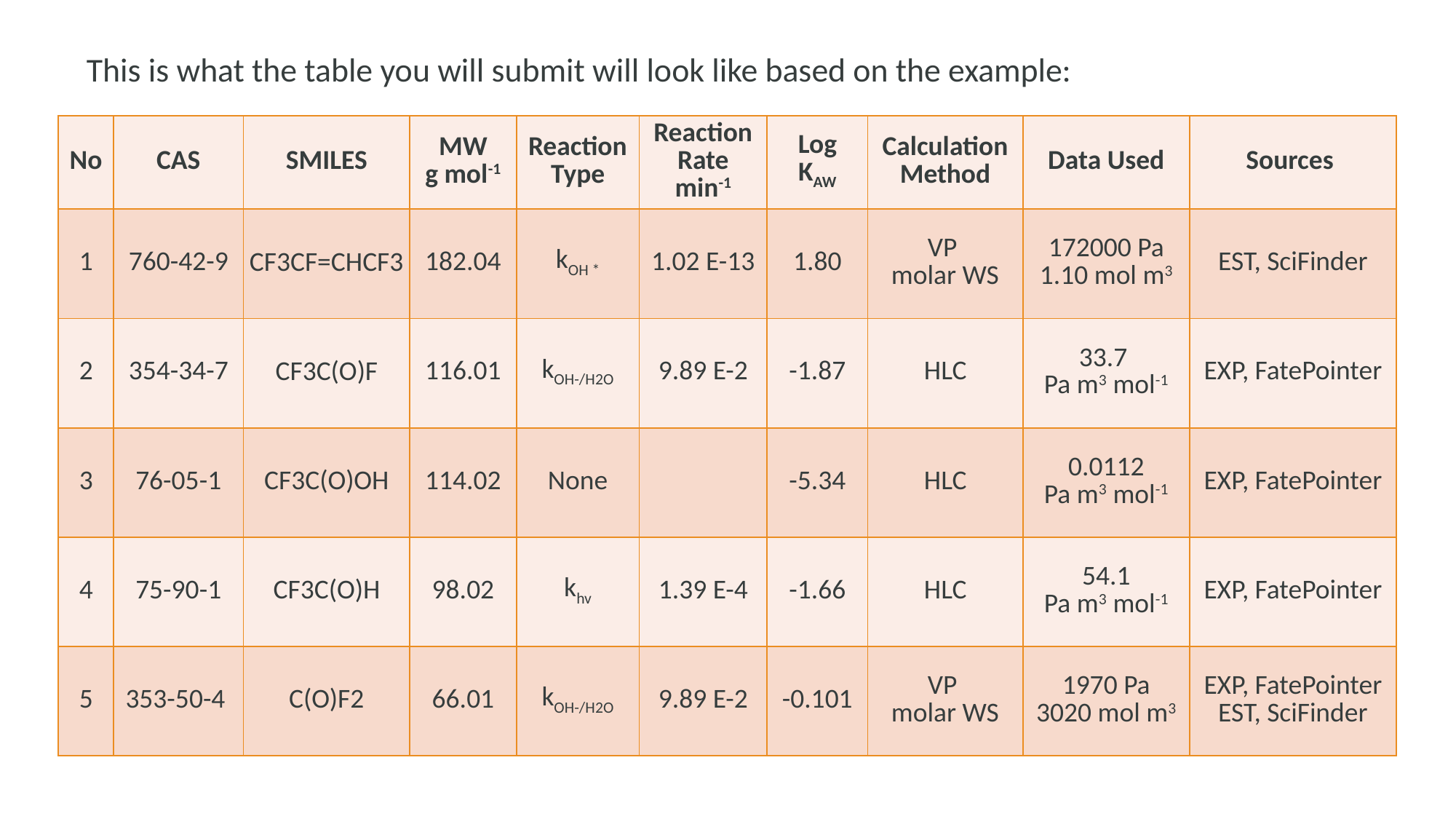

This is what the table you will submit will look like based on the example:
| No | CAS | SMILES | MW g mol-1 | Reaction Type | Reaction Rate min-1 | Log KAW | Calculation Method | Data Used | Sources |
| --- | --- | --- | --- | --- | --- | --- | --- | --- | --- |
| 1 | 760-42-9 | CF3CF=CHCF3 | 182.04 | kOH \* | 1.02 E-13 | 1.80 | VP molar WS | 172000 Pa 1.10 mol m3 | EST, SciFinder |
| 2 | 354-34-7 | CF3C(O)F | 116.01 | kOH-/H2O | 9.89 E-2 | -1.87 | HLC | 33.7 Pa m3 mol-1 | EXP, FatePointer |
| 3 | 76-05-1 | CF3C(O)OH | 114.02 | None | | -5.34 | HLC | 0.0112 Pa m3 mol-1 | EXP, FatePointer |
| 4 | 75-90-1 | CF3C(O)H | 98.02 | khv | 1.39 E-4 | -1.66 | HLC | 54.1 Pa m3 mol-1 | EXP, FatePointer |
| 5 | 353-50-4 | C(O)F2 | 66.01 | kOH-/H2O | 9.89 E-2 | -0.101 | VP molar WS | 1970 Pa 3020 mol m3 | EXP, FatePointer EST, SciFinder |

## Slide 11
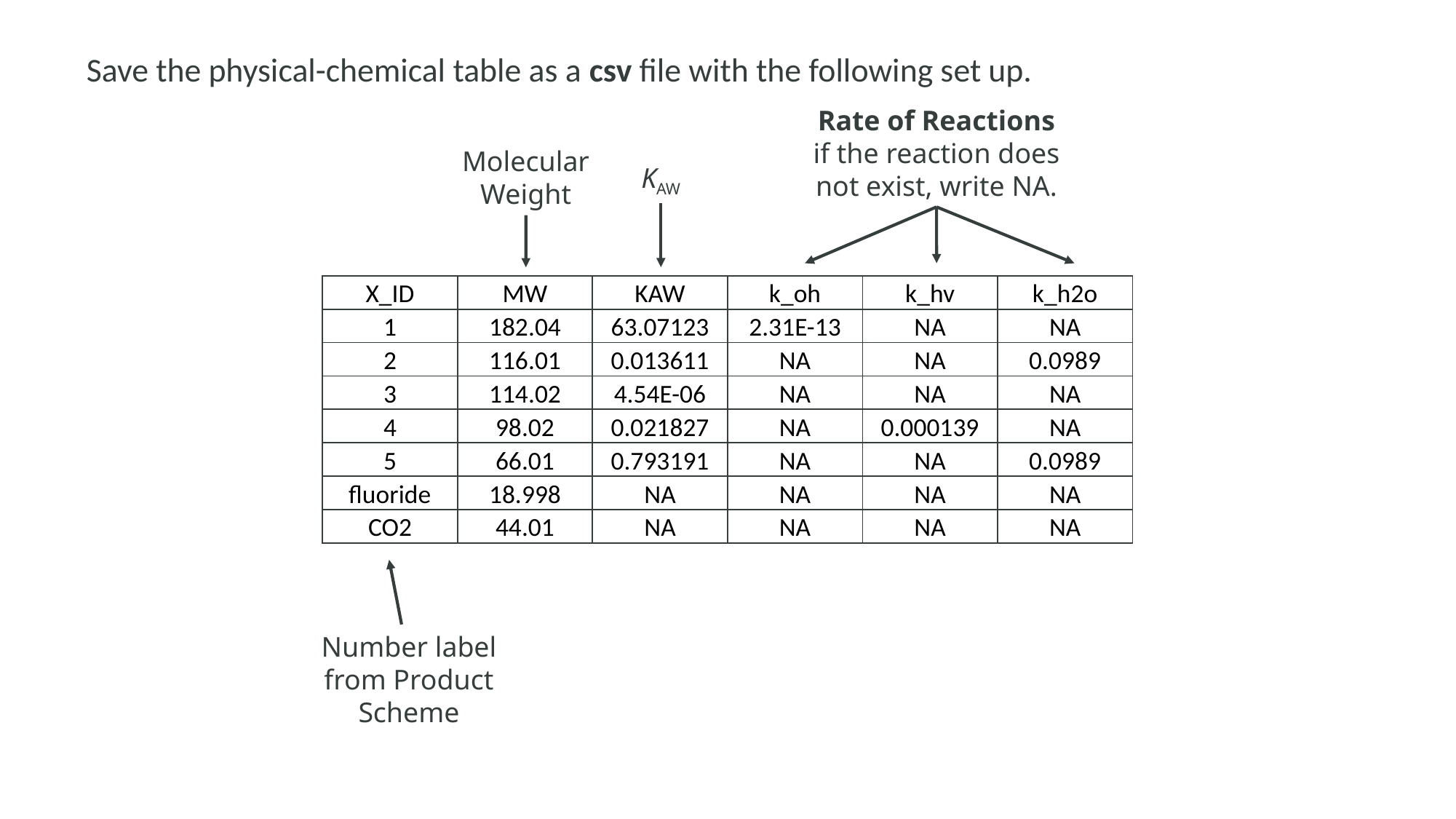

Save the physical-chemical table as a csv file with the following set up.
Rate of Reactions
if the reaction does not exist, write NA.
Molecular Weight
KAW
| X\_ID | MW | KAW | k\_oh | k\_hv | k\_h2o |
| --- | --- | --- | --- | --- | --- |
| 1 | 182.04 | 63.07123 | 2.31E-13 | NA | NA |
| 2 | 116.01 | 0.013611 | NA | NA | 0.0989 |
| 3 | 114.02 | 4.54E-06 | NA | NA | NA |
| 4 | 98.02 | 0.021827 | NA | 0.000139 | NA |
| 5 | 66.01 | 0.793191 | NA | NA | 0.0989 |
| fluoride | 18.998 | NA | NA | NA | NA |
| CO2 | 44.01 | NA | NA | NA | NA |
Number label from Product Scheme

## Slide 12
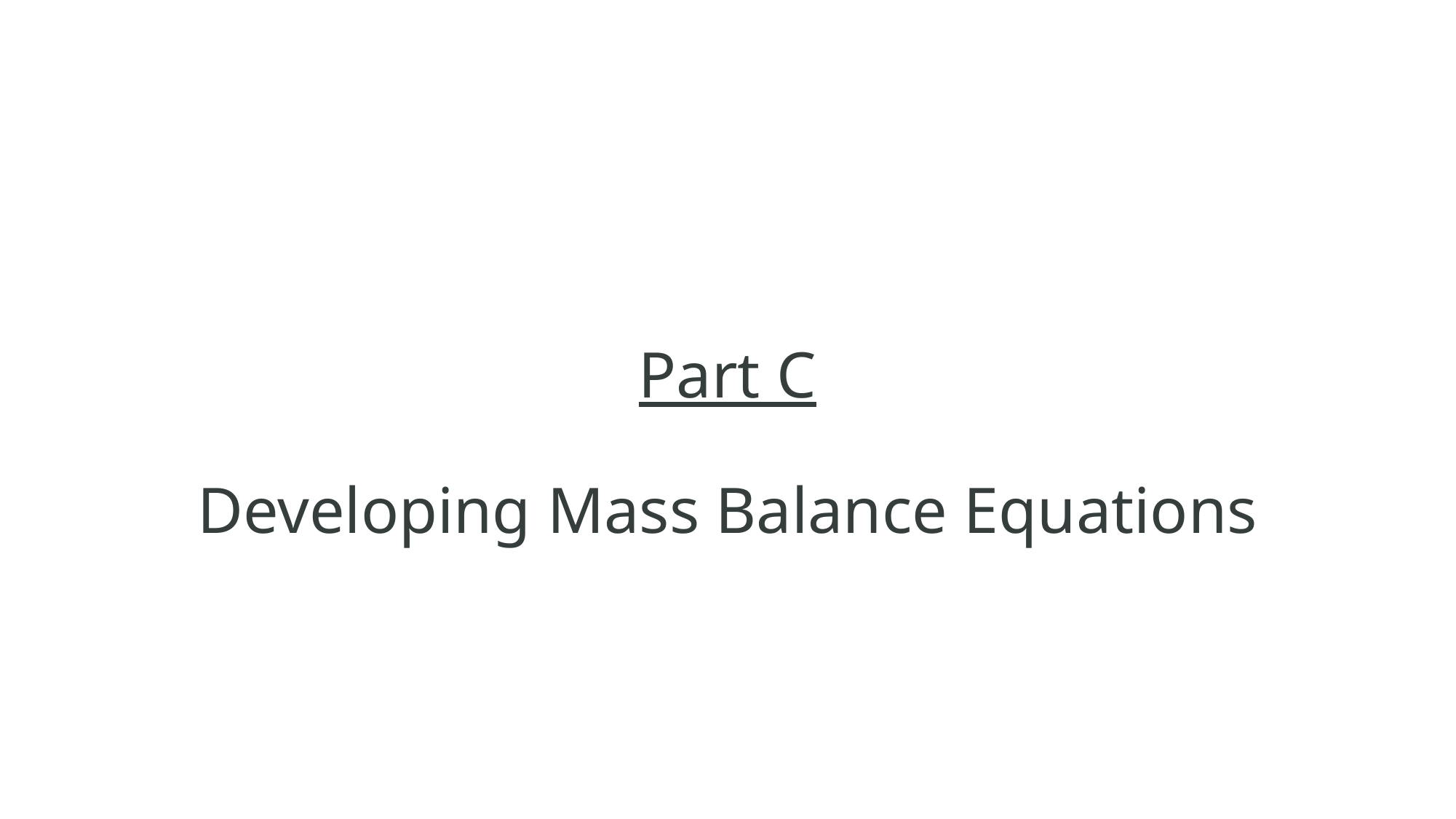

# Part C Developing Mass Balance Equations

## Slide 13
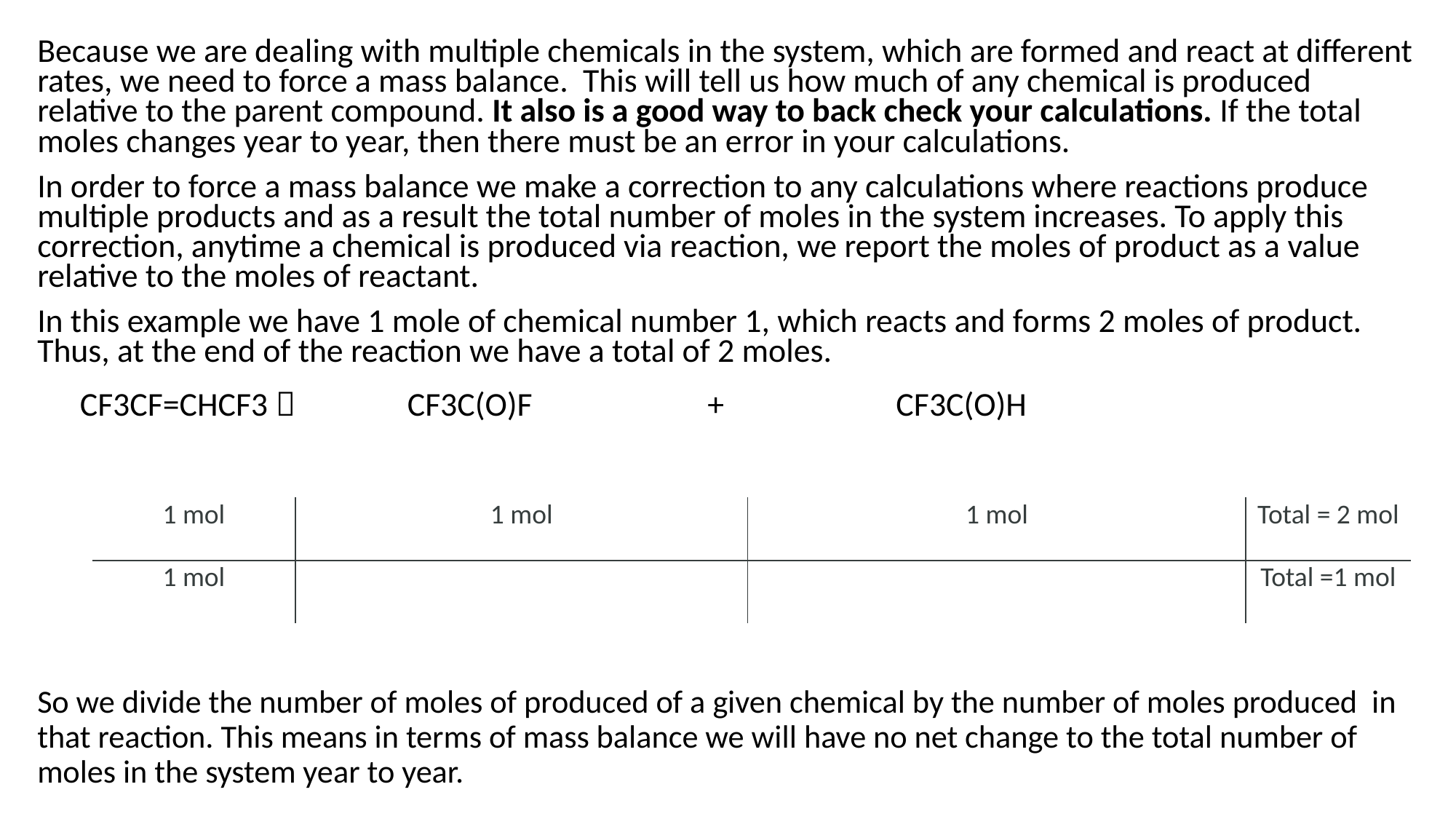

Because we are dealing with multiple chemicals in the system, which are formed and react at different rates, we need to force a mass balance. This will tell us how much of any chemical is produced relative to the parent compound. It also is a good way to back check your calculations. If the total moles changes year to year, then there must be an error in your calculations.
In order to force a mass balance we make a correction to any calculations where reactions produce multiple products and as a result the total number of moles in the system increases. To apply this correction, anytime a chemical is produced via reaction, we report the moles of product as a value relative to the moles of reactant.
In this example we have 1 mole of chemical number 1, which reacts and forms 2 moles of product. Thus, at the end of the reaction we have a total of 2 moles.
CF3CF=CHCF3 		CF3C(O)F	 +		 CF3C(O)H
So we divide the number of moles of produced of a given chemical by the number of moles produced in that reaction. This means in terms of mass balance we will have no net change to the total number of moles in the system year to year.

## Slide 14
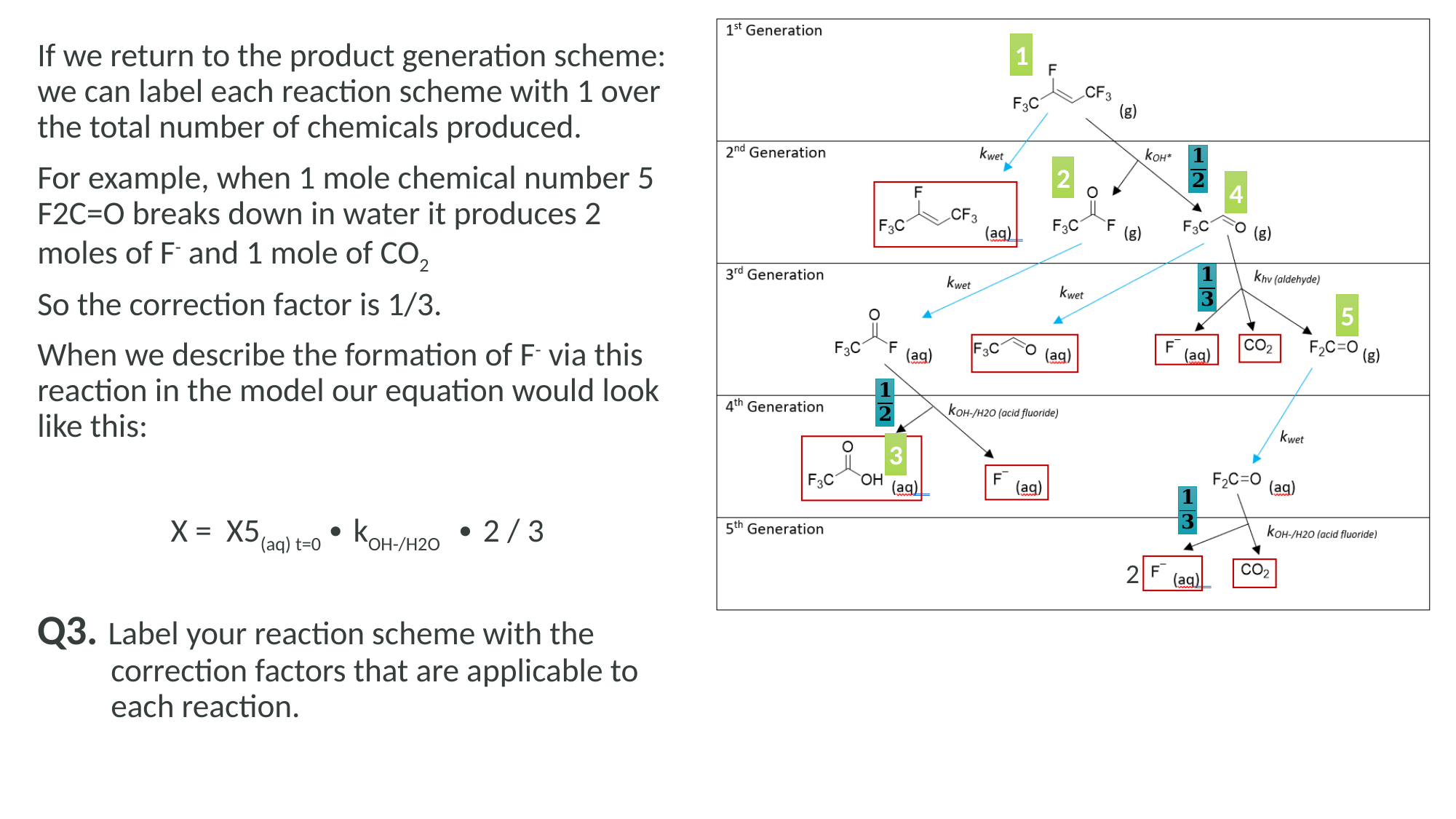

If we return to the product generation scheme: we can label each reaction scheme with 1 over the total number of chemicals produced.
For example, when 1 mole chemical number 5 F2C=O breaks down in water it produces 2 moles of F- and 1 mole of CO2
So the correction factor is 1/3.
When we describe the formation of F- via this reaction in the model our equation would look like this:
X = X5(aq) t=0 ∙ kOH-/H2O ∙ 2 / 3
Q3. Label your reaction scheme with the correction factors that are applicable to each reaction.
1
2
4
5
3
2

## Slide 15
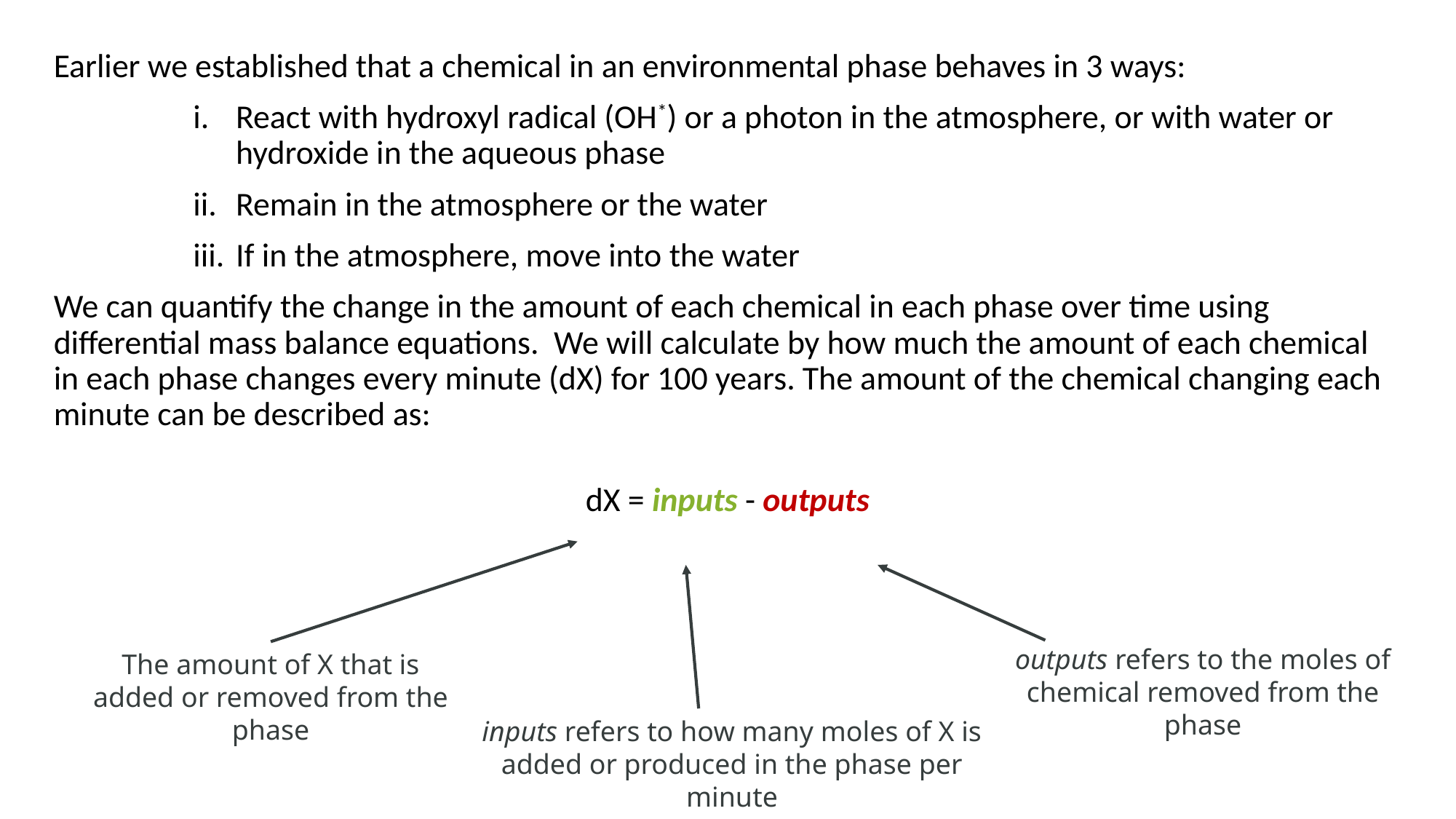

Earlier we established that a chemical in an environmental phase behaves in 3 ways:
React with hydroxyl radical (OH*) or a photon in the atmosphere, or with water or hydroxide in the aqueous phase
Remain in the atmosphere or the water
If in the atmosphere, move into the water
We can quantify the change in the amount of each chemical in each phase over time using differential mass balance equations. We will calculate by how much the amount of each chemical in each phase changes every minute (dX) for 100 years. The amount of the chemical changing each minute can be described as:
dX = inputs - outputs
outputs refers to the moles of chemical removed from the phase
The amount of X that is added or removed from the phase
inputs refers to how many moles of X is added or produced in the phase per minute

## Slide 16
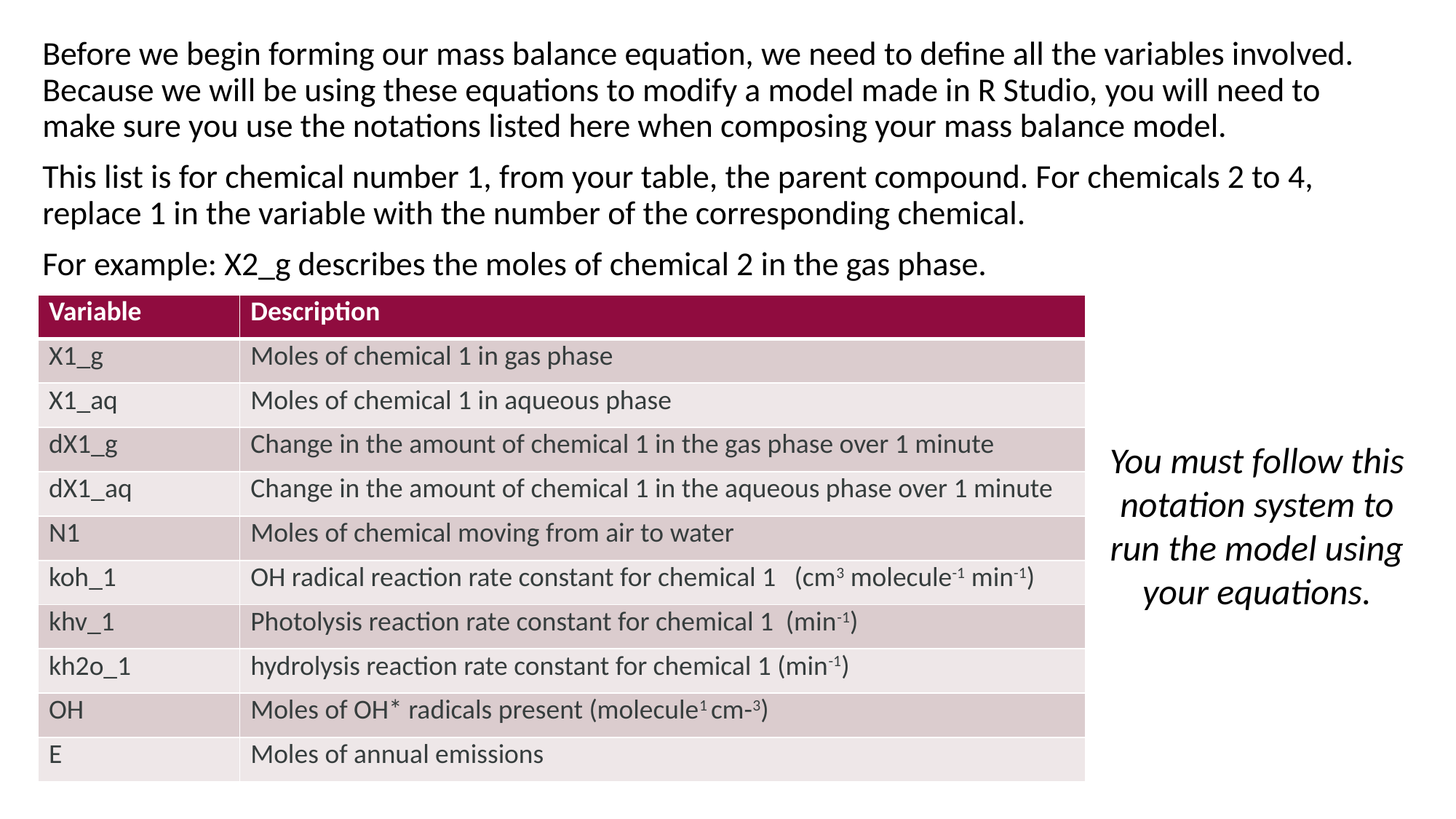

Before we begin forming our mass balance equation, we need to define all the variables involved. Because we will be using these equations to modify a model made in R Studio, you will need to make sure you use the notations listed here when composing your mass balance model.
This list is for chemical number 1, from your table, the parent compound. For chemicals 2 to 4, replace 1 in the variable with the number of the corresponding chemical.
For example: X2_g describes the moles of chemical 2 in the gas phase.
| Variable | Description |
| --- | --- |
| X1\_g | Moles of chemical 1 in gas phase |
| X1\_aq | Moles of chemical 1 in aqueous phase |
| dX1\_g | Change in the amount of chemical 1 in the gas phase over 1 minute |
| dX1\_aq | Change in the amount of chemical 1 in the aqueous phase over 1 minute |
| N1 | Moles of chemical moving from air to water |
| koh\_1 | OH radical reaction rate constant for chemical 1 (cm3 molecule-1 min-1) |
| khv\_1 | Photolysis reaction rate constant for chemical 1 (min-1) |
| kh2o\_1 | hydrolysis reaction rate constant for chemical 1 (min-1) |
| OH | Moles of OH\* radicals present (molecule1 cm-3) |
| E | Moles of annual emissions |
You must follow this notation system to run the model using your equations.

## Slide 17
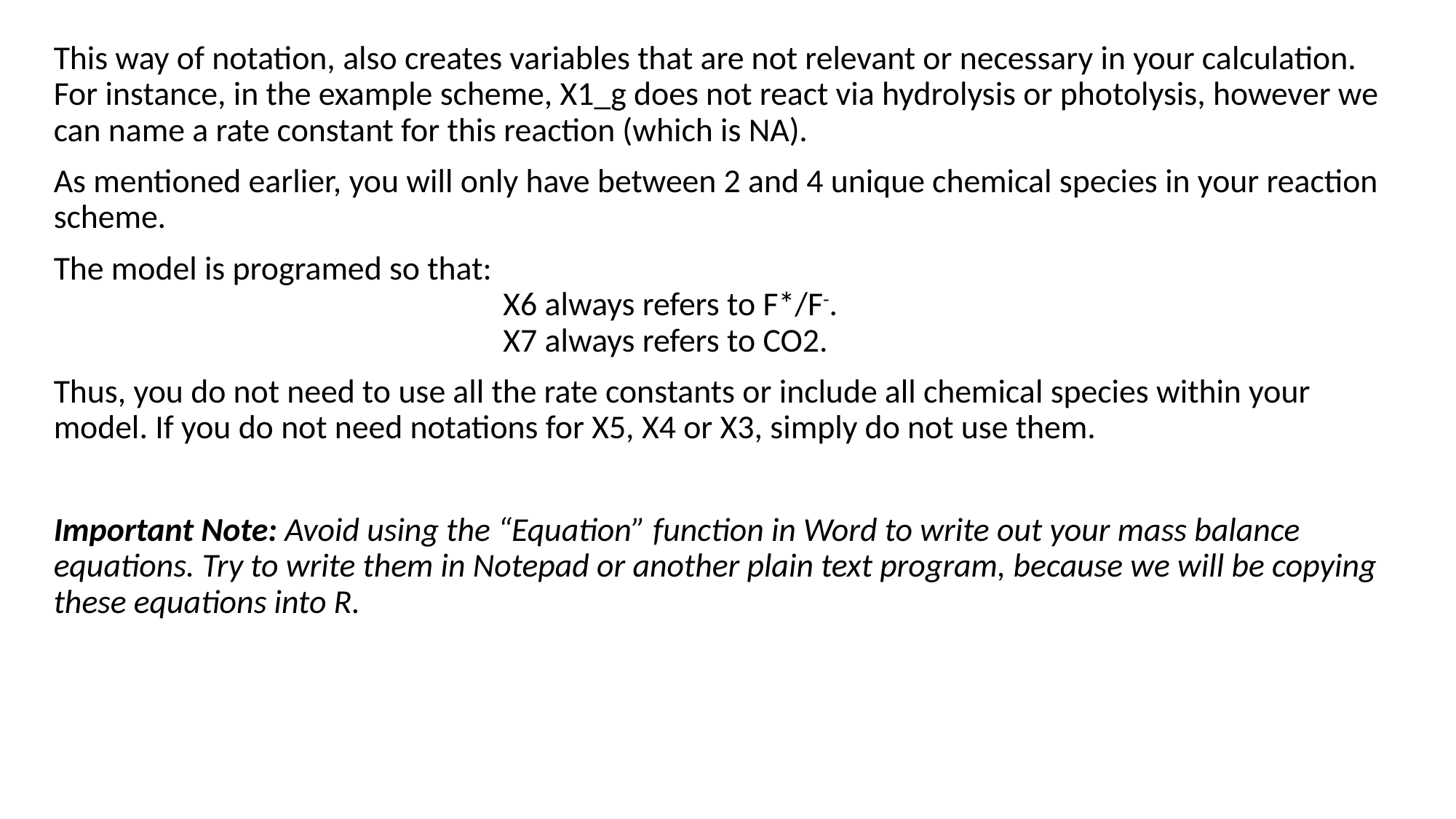

This way of notation, also creates variables that are not relevant or necessary in your calculation. For instance, in the example scheme, X1_g does not react via hydrolysis or photolysis, however we can name a rate constant for this reaction (which is NA).
As mentioned earlier, you will only have between 2 and 4 unique chemical species in your reaction scheme.
The model is programed so that:
X6 always refers to F*/F-.
X7 always refers to CO2.
Thus, you do not need to use all the rate constants or include all chemical species within your model. If you do not need notations for X5, X4 or X3, simply do not use them.
Important Note: Avoid using the “Equation” function in Word to write out your mass balance equations. Try to write them in Notepad or another plain text program, because we will be copying these equations into R.

## Slide 18
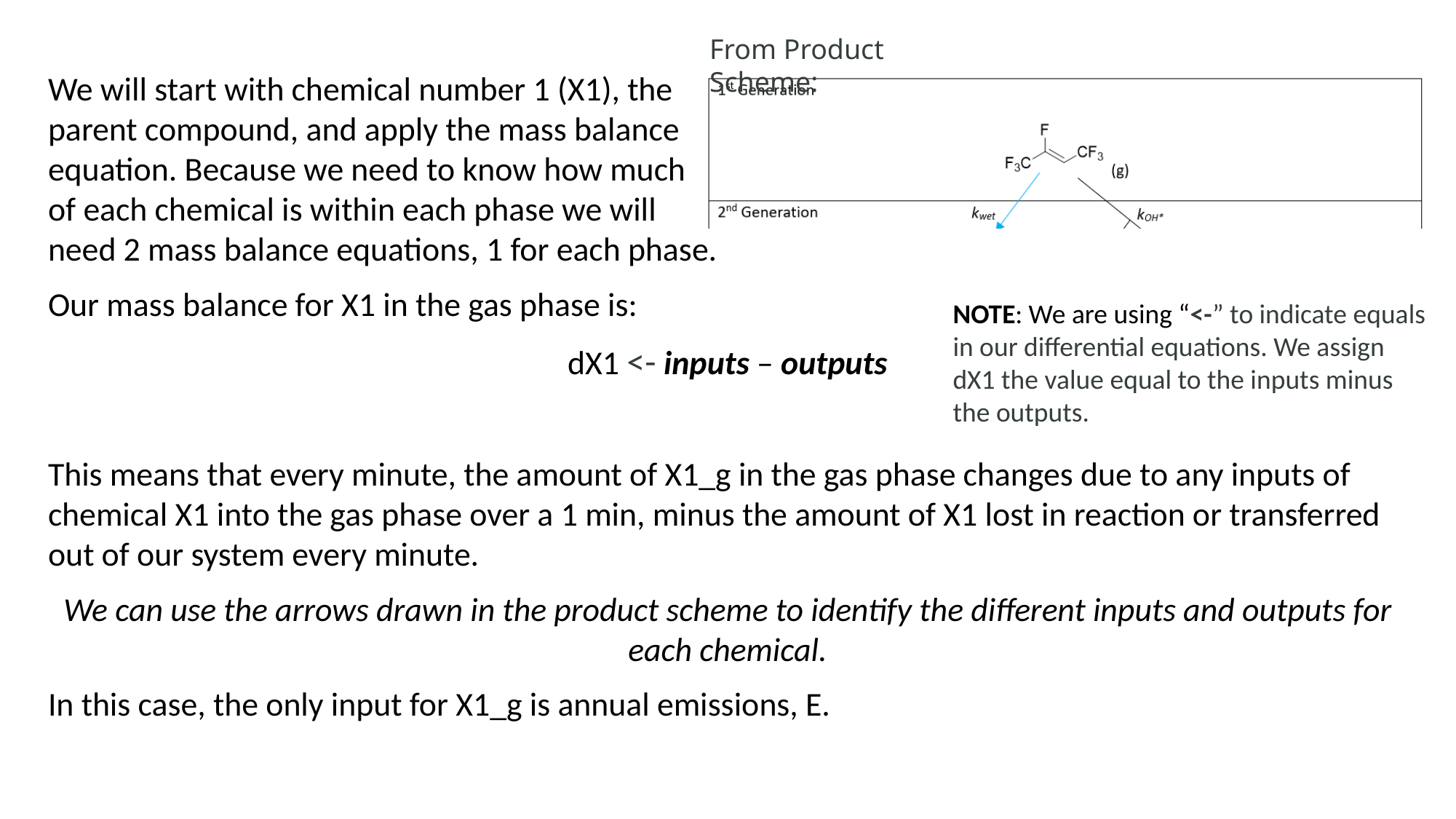

From Product Scheme:
We will start with chemical number 1 (X1), the
parent compound, and apply the mass balance
equation. Because we need to know how much
of each chemical is within each phase we will
need 2 mass balance equations, 1 for each phase.
Our mass balance for X1 in the gas phase is:
dX1 <- inputs – outputs
This means that every minute, the amount of X1_g in the gas phase changes due to any inputs of chemical X1 into the gas phase over a 1 min, minus the amount of X1 lost in reaction or transferred out of our system every minute.
We can use the arrows drawn in the product scheme to identify the different inputs and outputs for each chemical.
In this case, the only input for X1_g is annual emissions, E.
NOTE: We are using “<-” to indicate equals in our differential equations. We assign dX1 the value equal to the inputs minus the outputs.

## Slide 19
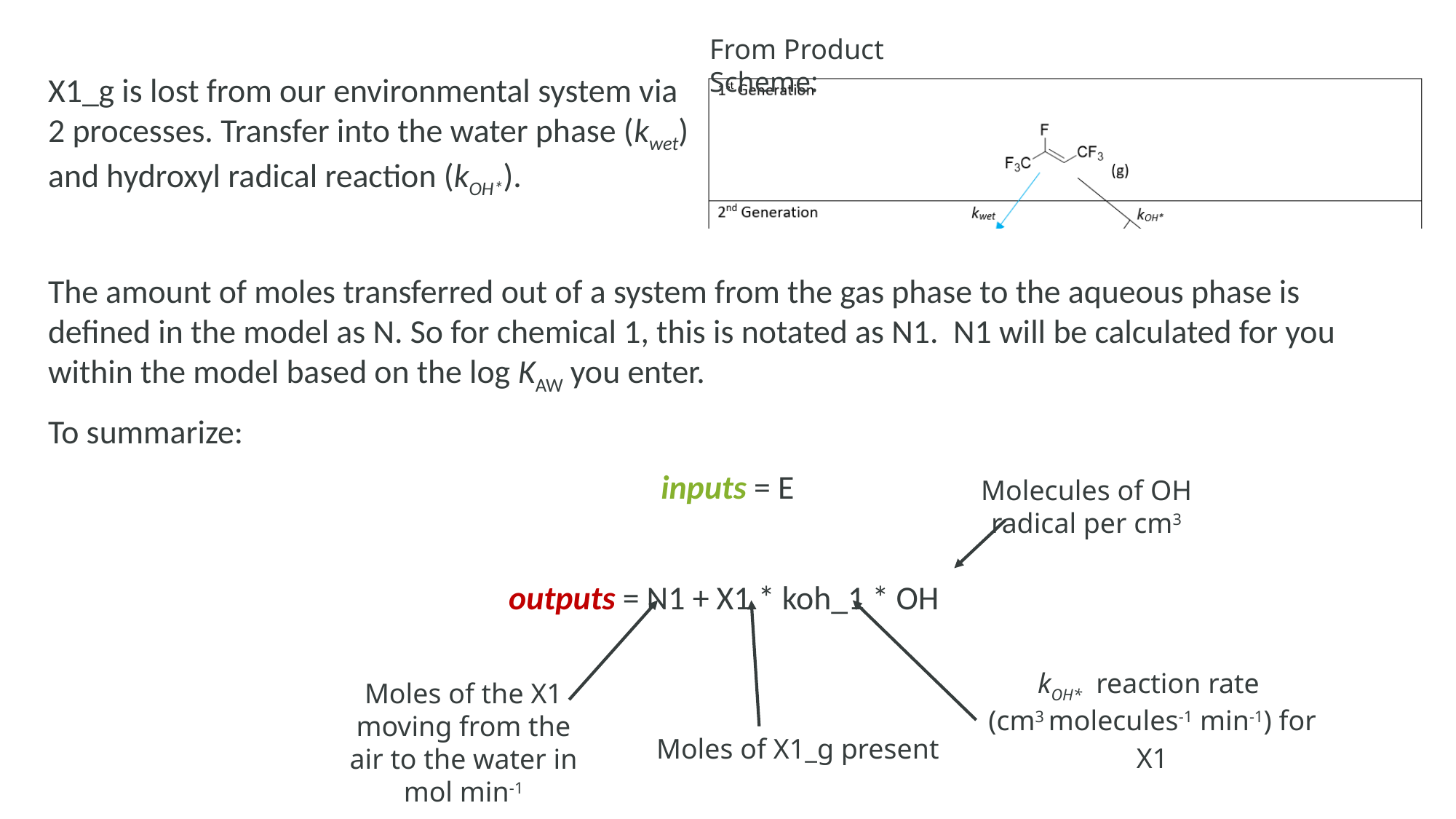

From Product Scheme:
X1_g is lost from our environmental system via
2 processes. Transfer into the water phase (kwet)
and hydroxyl radical reaction (kOH*).
The amount of moles transferred out of a system from the gas phase to the aqueous phase is defined in the model as N. So for chemical 1, this is notated as N1. N1 will be calculated for you within the model based on the log KAW you enter.
To summarize:
inputs = E
outputs = N1 + X1 * koh_1 * OH
Molecules of OH radical per cm3
kOH* reaction rate
(cm3 molecules-1 min-1) for X1
Moles of the X1 moving from the air to the water in mol min-1
Moles of X1_g present

## Slide 20
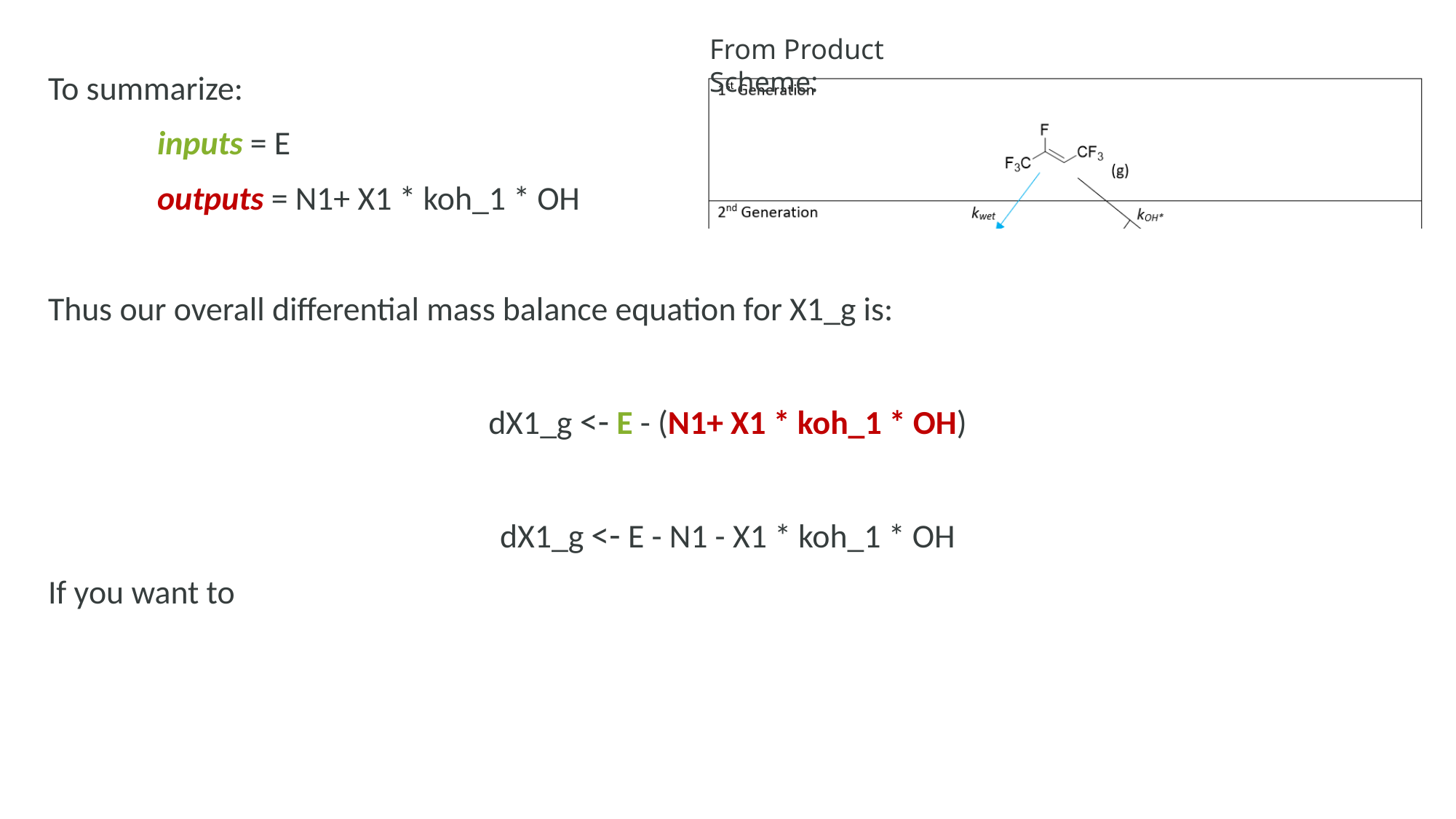

From Product Scheme:
To summarize:
	inputs = E
	outputs = N1+ X1 * koh_1 * OH
Thus our overall differential mass balance equation for X1_g is:
dX1_g <- E - (N1+ X1 * koh_1 * OH)
dX1_g <- E - N1 - X1 * koh_1 * OH
If you want to

## Slide 21
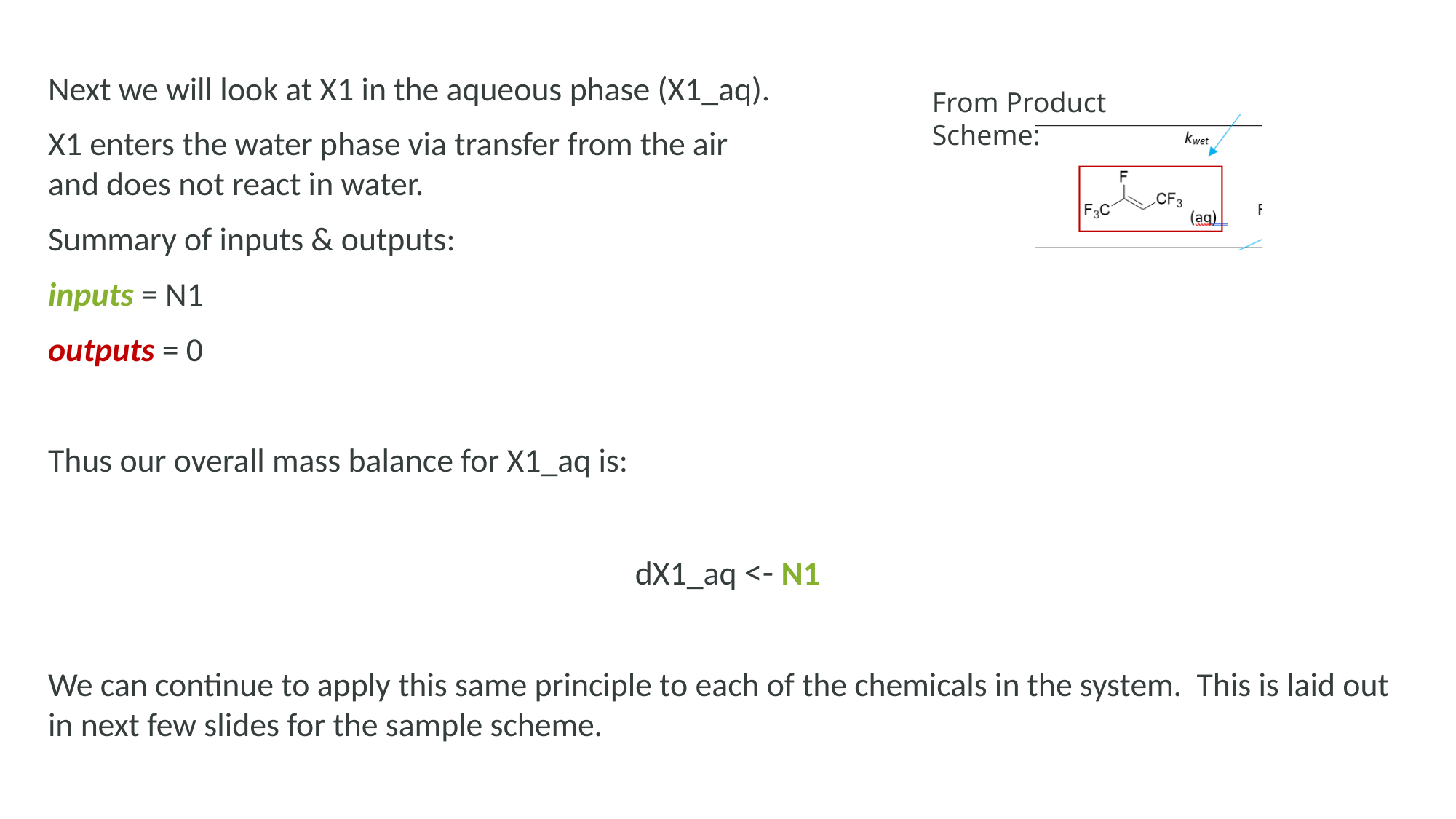

Next we will look at X1 in the aqueous phase (X1_aq).
X1 enters the water phase via transfer from the air
and does not react in water.
Summary of inputs & outputs:
inputs = N1
outputs = 0
Thus our overall mass balance for X1_aq is:
dX1_aq <- N1
We can continue to apply this same principle to each of the chemicals in the system. This is laid out in next few slides for the sample scheme.
From Product Scheme:

## Slide 22
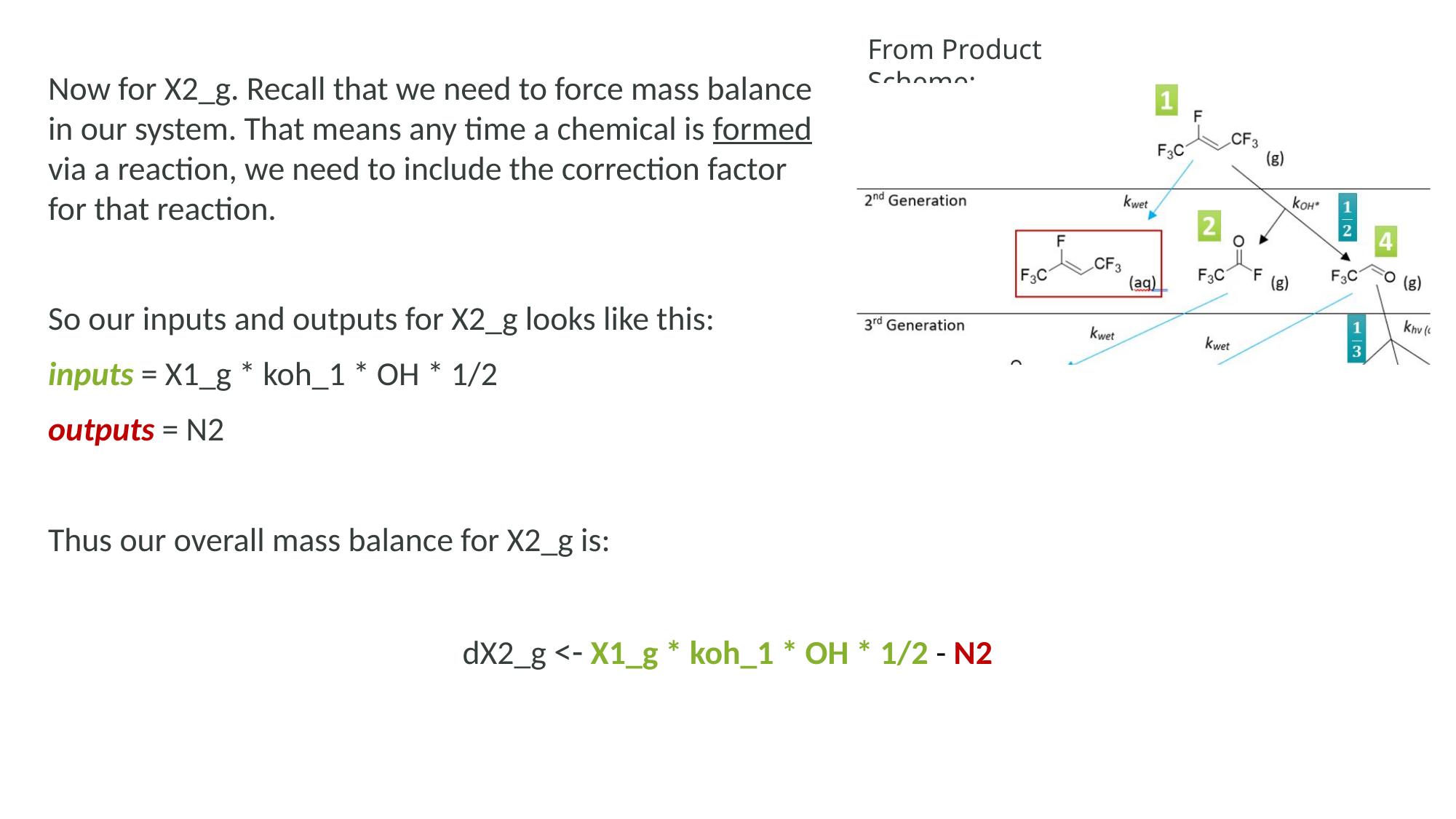

From Product Scheme:
Now for X2_g. Recall that we need to force mass balance
in our system. That means any time a chemical is formed
via a reaction, we need to include the correction factor
for that reaction.
So our inputs and outputs for X2_g looks like this:
inputs = X1_g * koh_1 * OH * 1/2
outputs = N2
Thus our overall mass balance for X2_g is:
dX2_g <- X1_g * koh_1 * OH * 1/2 - N2

## Slide 23
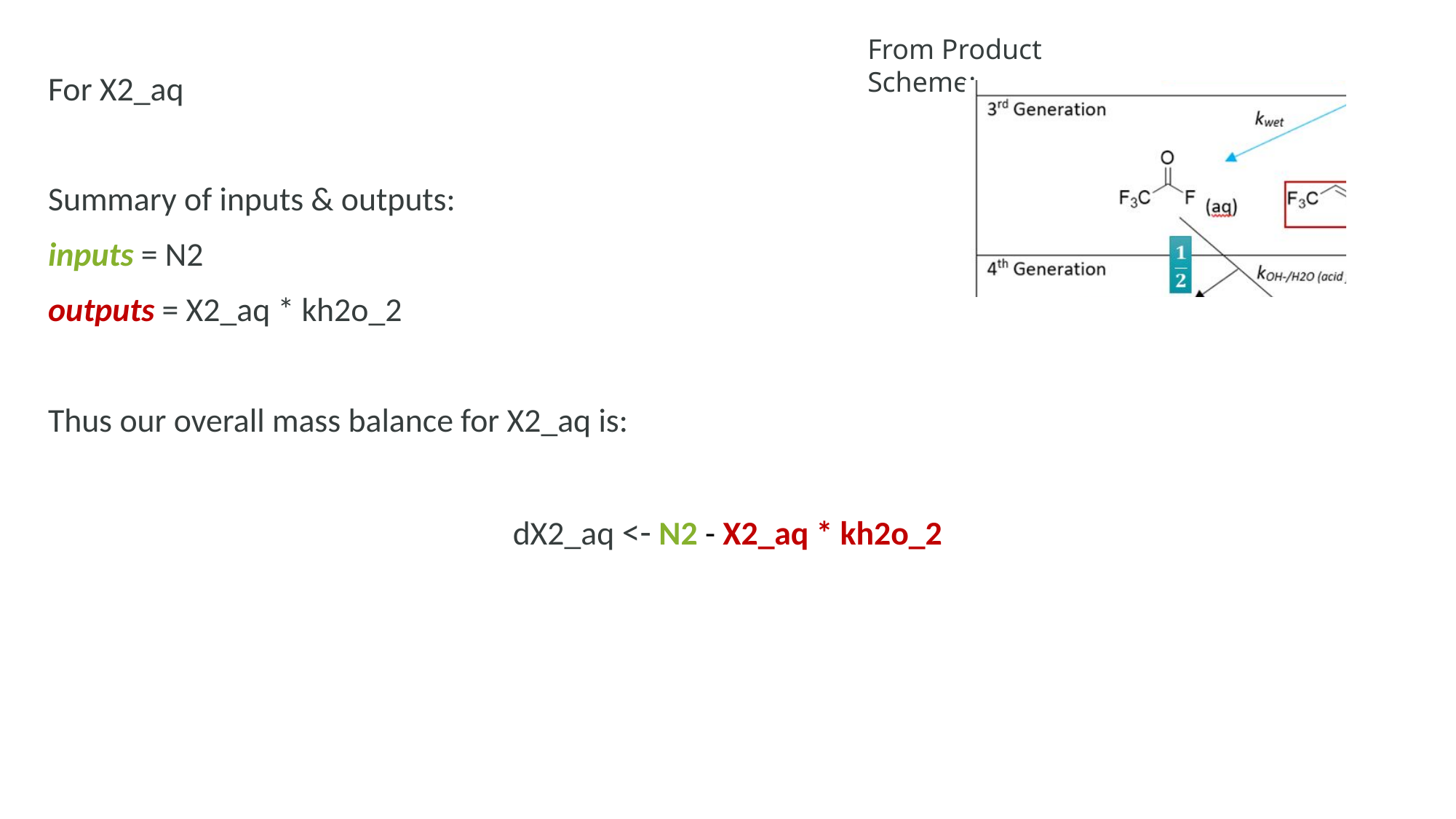

From Product Scheme:
For X2_aq
Summary of inputs & outputs:
inputs = N2
outputs = X2_aq * kh2o_2
Thus our overall mass balance for X2_aq is:
dX2_aq <- N2 - X2_aq * kh2o_2

## Slide 24
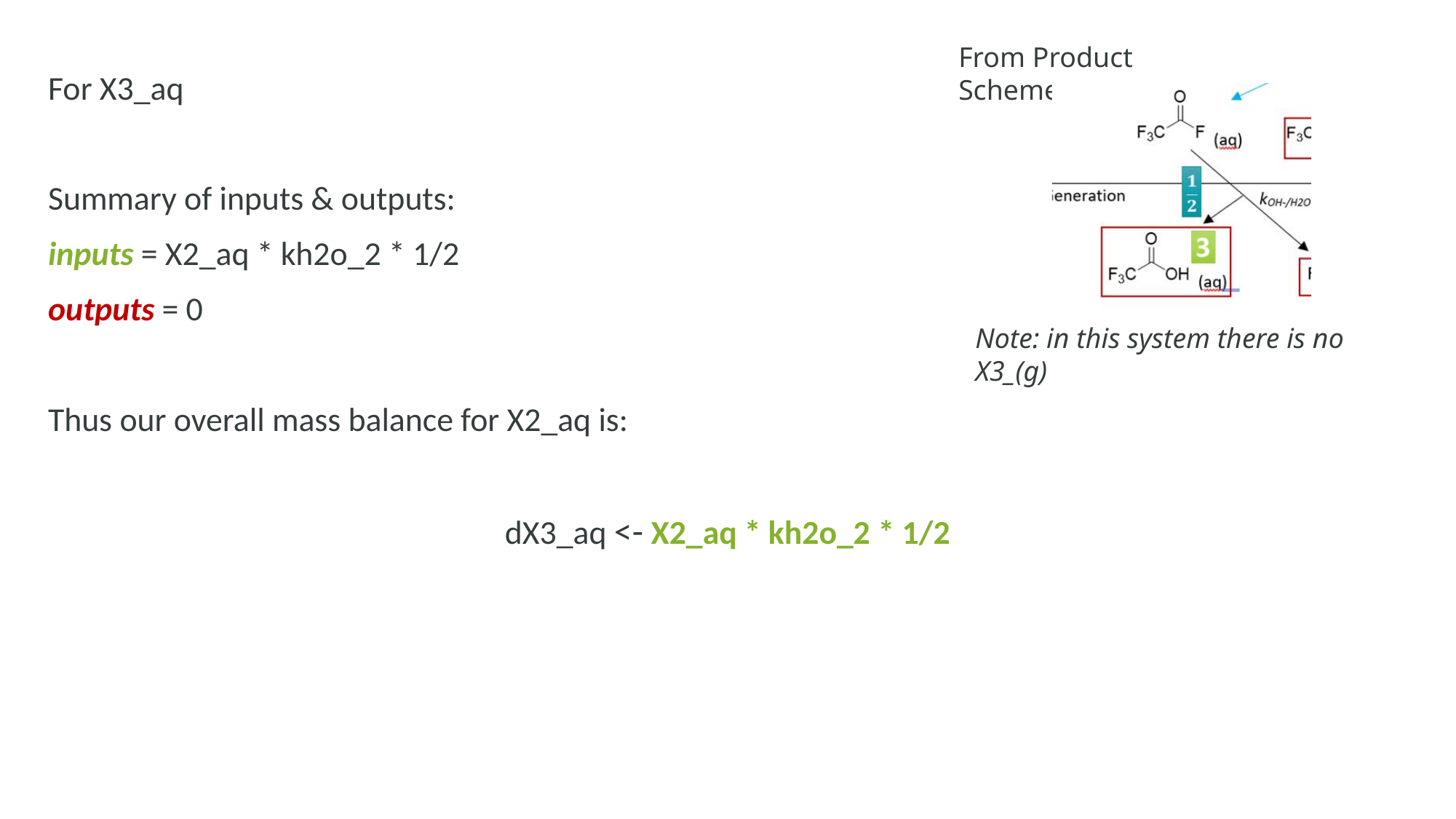

From Product Scheme:
For X3_aq
Summary of inputs & outputs:
inputs = X2_aq * kh2o_2 * 1/2
outputs = 0
Thus our overall mass balance for X2_aq is:
dX3_aq <- X2_aq * kh2o_2 * 1/2
Note: in this system there is no X3_(g)

## Slide 25
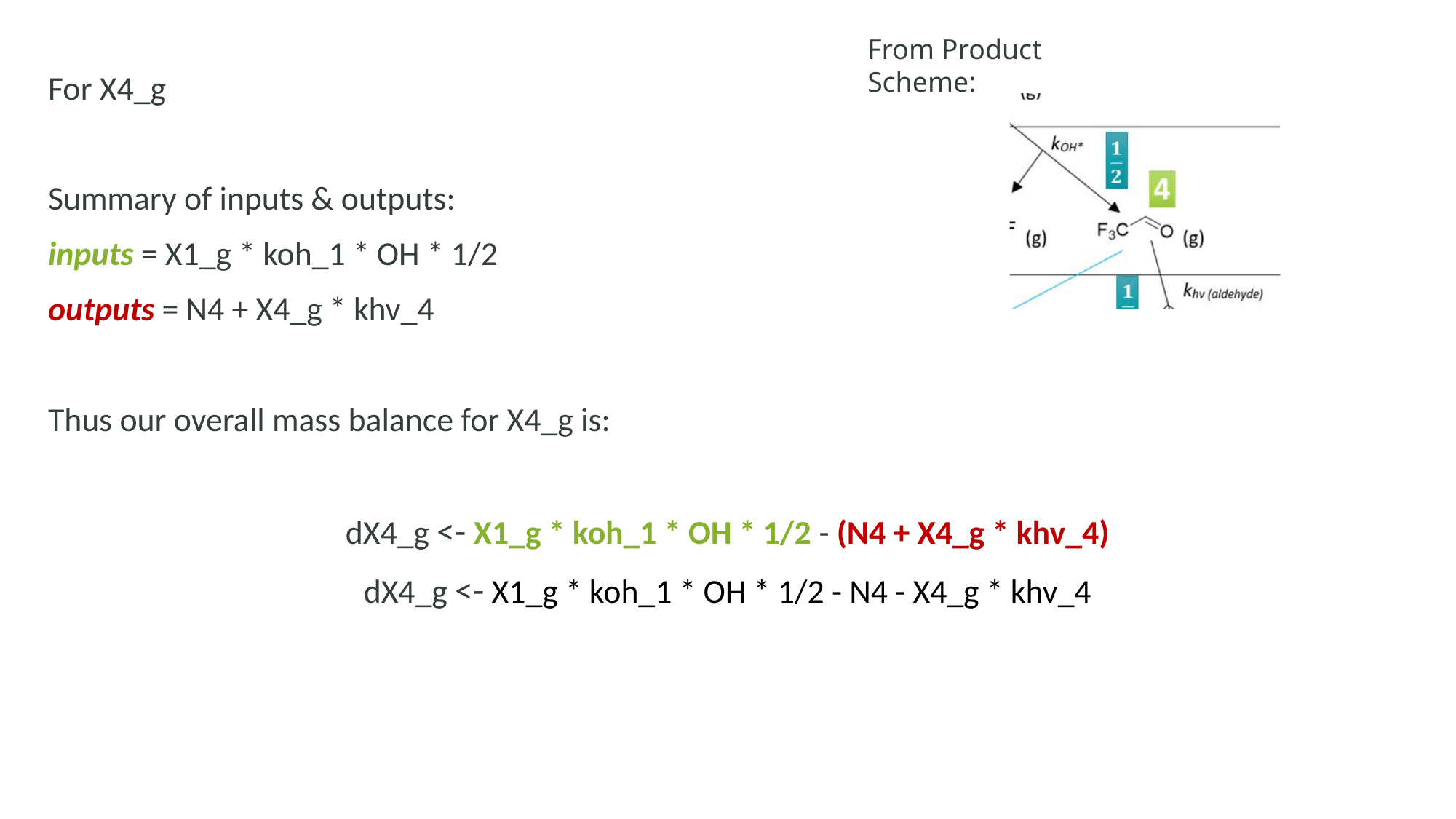

From Product Scheme:
For X4_g
Summary of inputs & outputs:
inputs = X1_g * koh_1 * OH * 1/2
outputs = N4 + X4_g * khv_4
Thus our overall mass balance for X4_g is:
dX4_g <- X1_g * koh_1 * OH * 1/2 - (N4 + X4_g * khv_4)
dX4_g <- X1_g * koh_1 * OH * 1/2 - N4 - X4_g * khv_4

## Slide 26
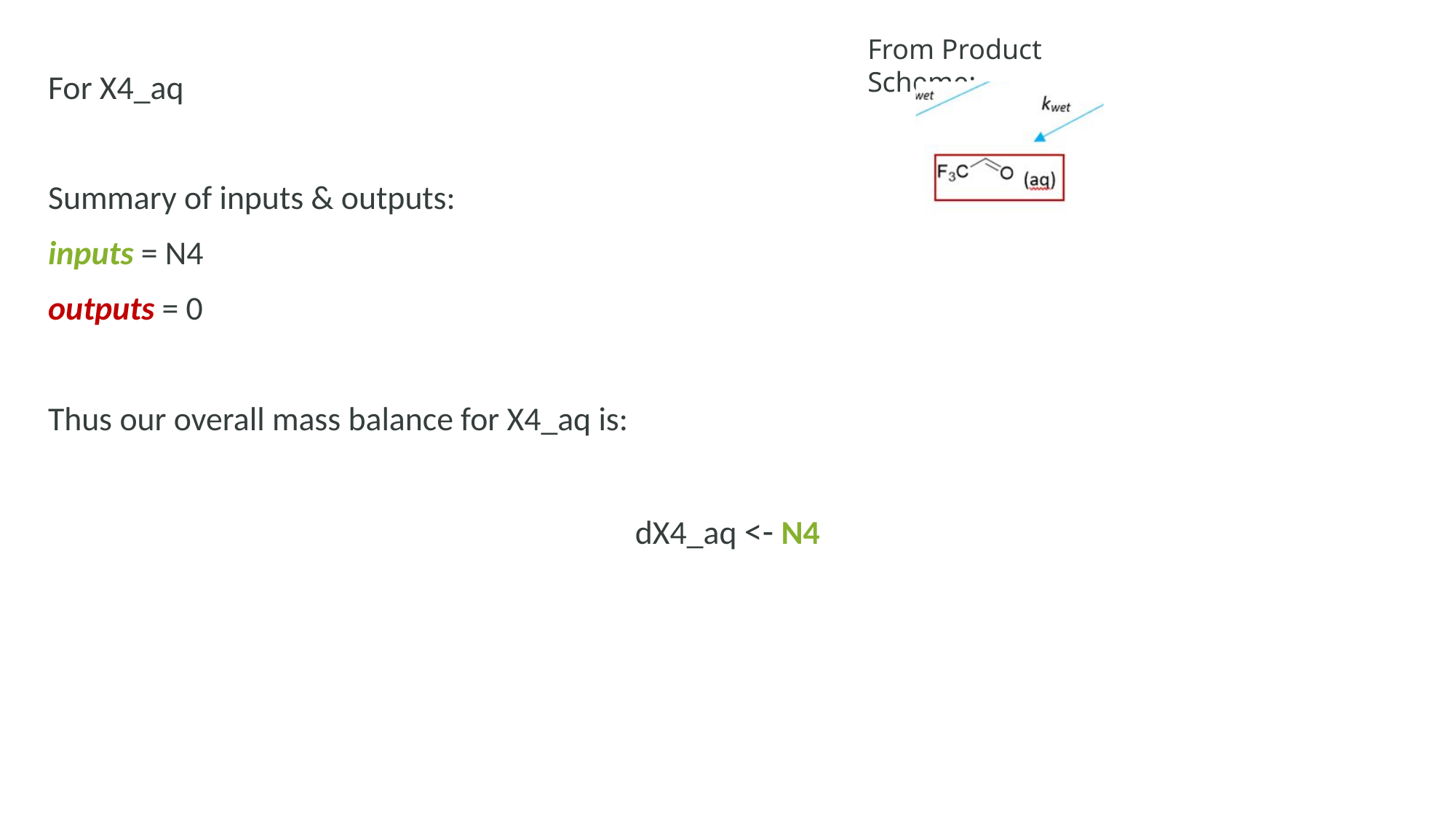

From Product Scheme:
For X4_aq
Summary of inputs & outputs:
inputs = N4
outputs = 0
Thus our overall mass balance for X4_aq is:
dX4_aq <- N4

## Slide 27
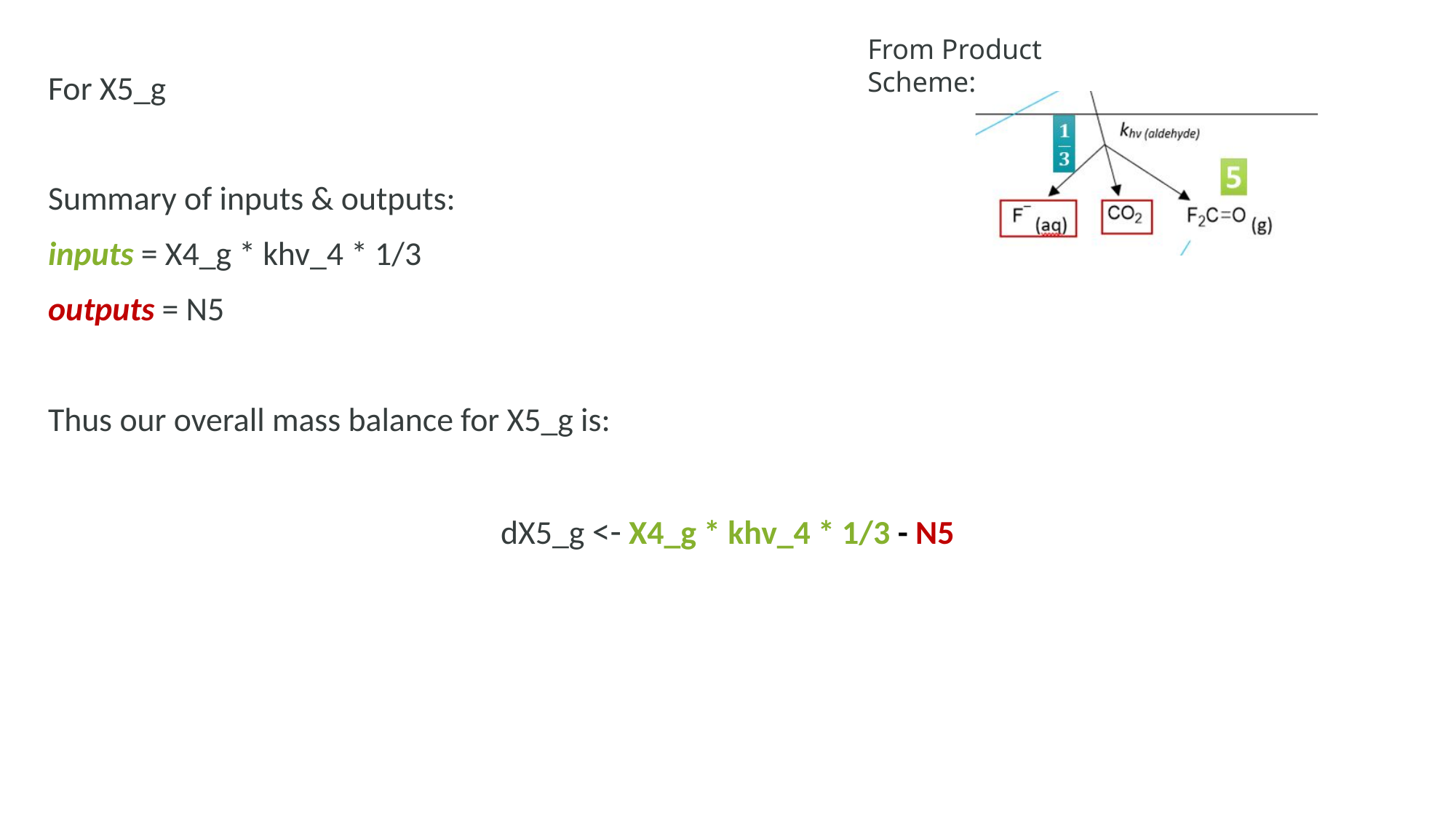

From Product Scheme:
For X5_g
Summary of inputs & outputs:
inputs = X4_g * khv_4 * 1/3
outputs = N5
Thus our overall mass balance for X5_g is:
dX5_g <- X4_g * khv_4 * 1/3 - N5

## Slide 28
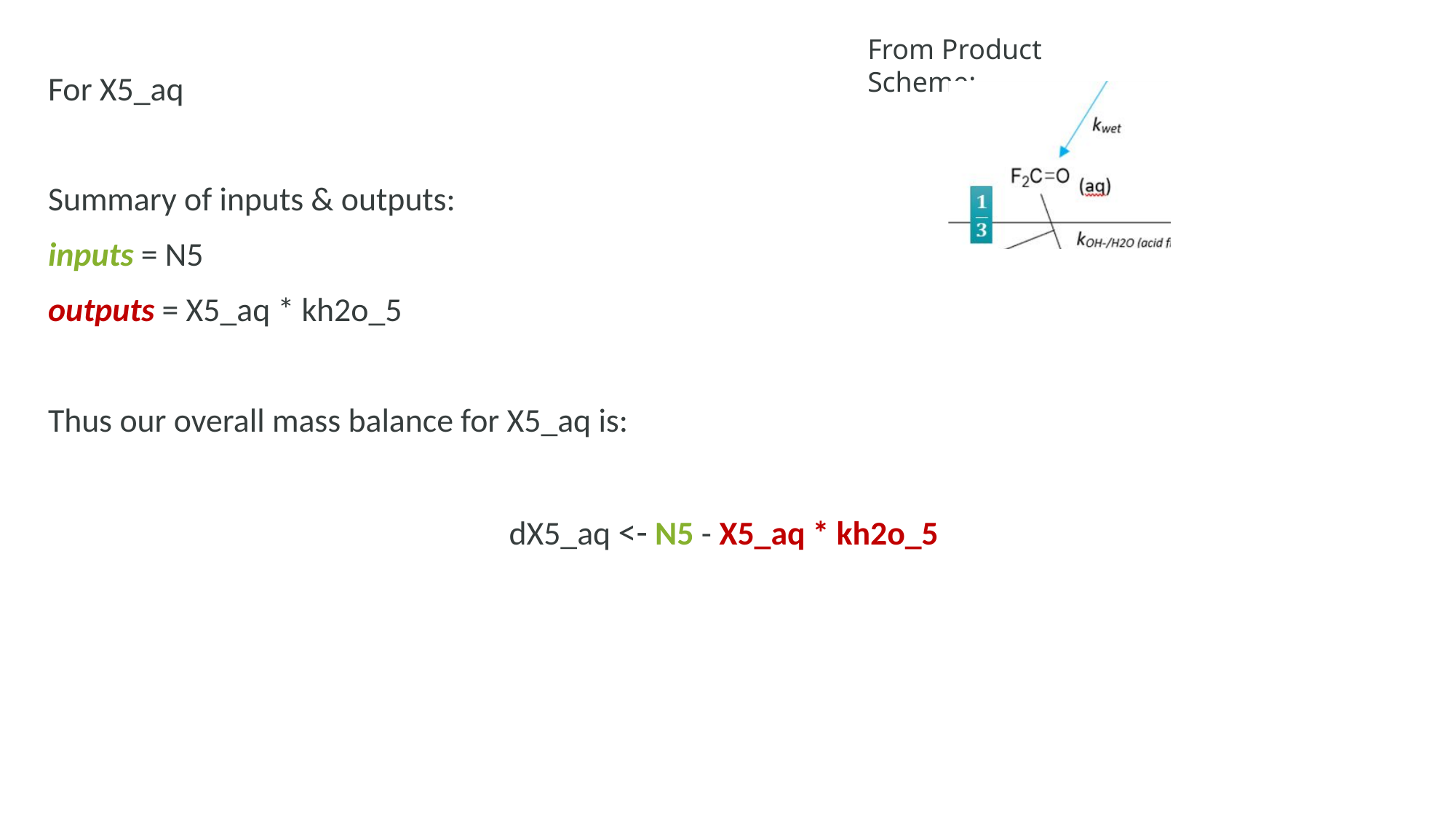

From Product Scheme:
For X5_aq
Summary of inputs & outputs:
inputs = N5
outputs = X5_aq * kh2o_5
Thus our overall mass balance for X5_aq is:
dX5_aq <- N5 - X5_aq * kh2o_5

## Slide 29
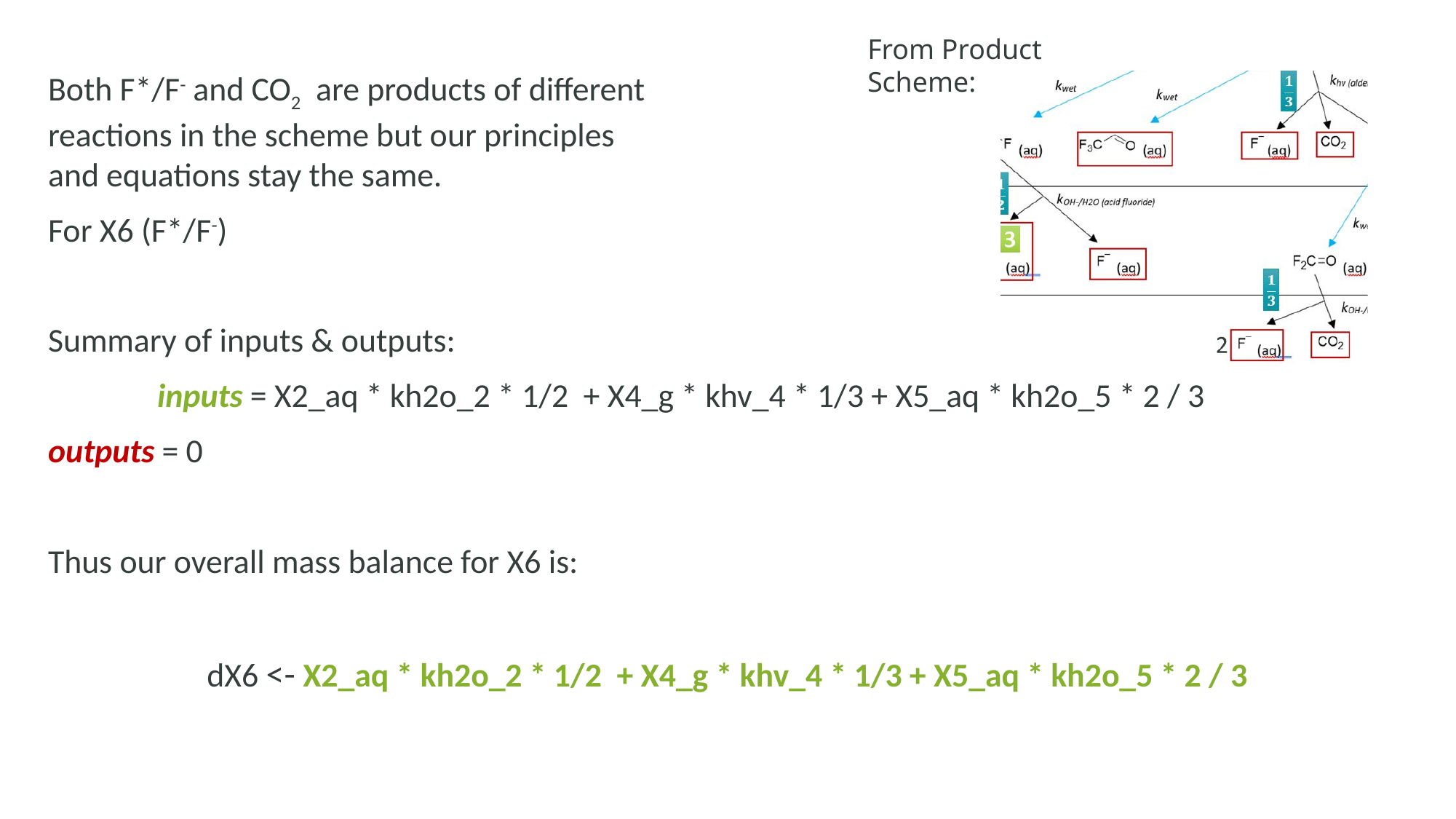

From Product Scheme:
Both F*/F- and CO2 are products of different
reactions in the scheme but our principles
and equations stay the same.
For X6 (F*/F-)
Summary of inputs & outputs:
	inputs = X2_aq * kh2o_2 * 1/2 + X4_g * khv_4 * 1/3 + X5_aq * kh2o_5 * 2 / 3
outputs = 0
Thus our overall mass balance for X6 is:
dX6 <- X2_aq * kh2o_2 * 1/2 + X4_g * khv_4 * 1/3 + X5_aq * kh2o_5 * 2 / 3

## Slide 30
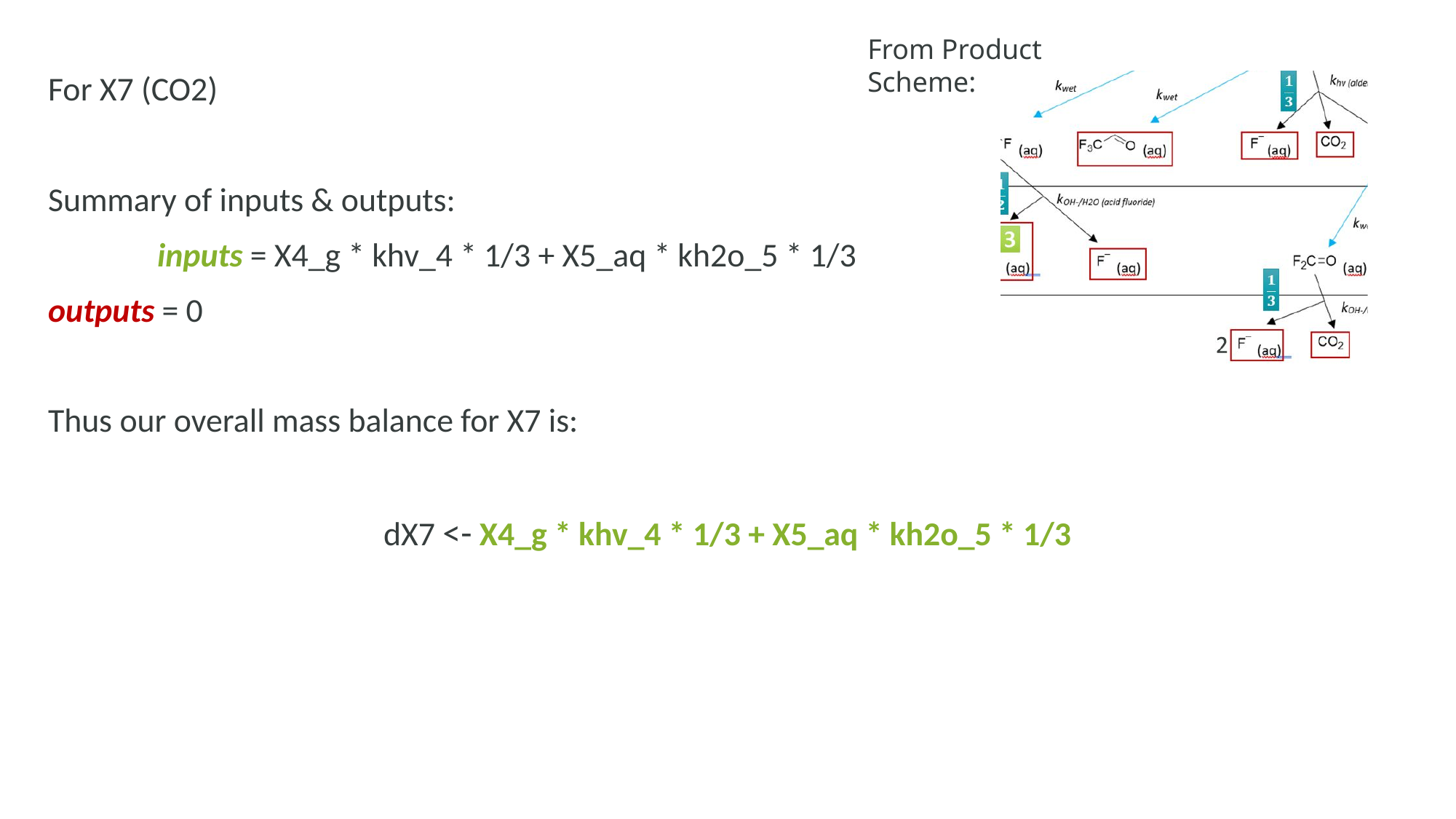

From Product Scheme:
For X7 (CO2)
Summary of inputs & outputs:
	inputs = X4_g * khv_4 * 1/3 + X5_aq * kh2o_5 * 1/3
outputs = 0
Thus our overall mass balance for X7 is:
dX7 <- X4_g * khv_4 * 1/3 + X5_aq * kh2o_5 * 1/3

## Slide 31
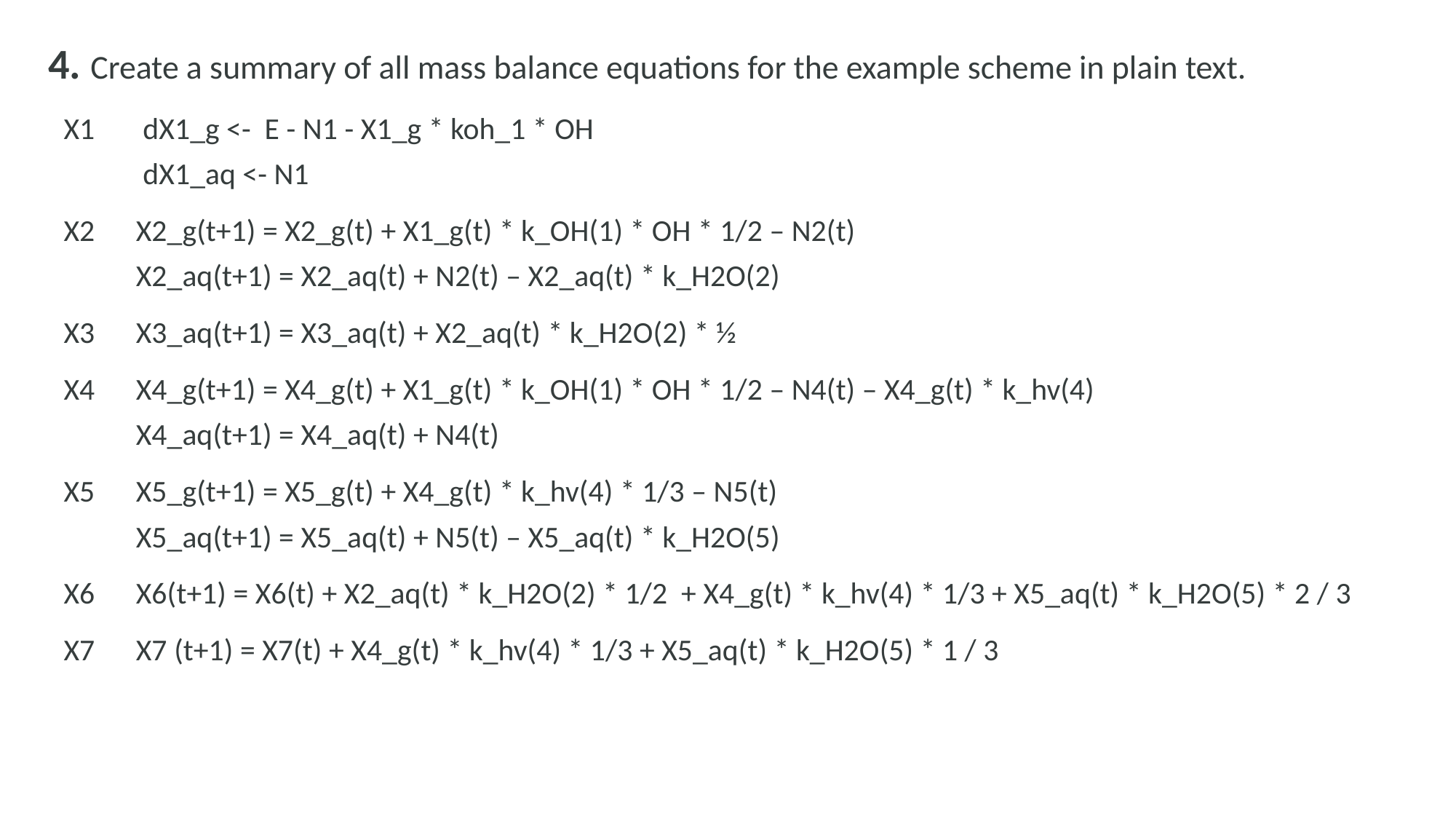

4. Create a summary of all mass balance equations for the example scheme in plain text.
| X1 | dX1\_g <- E - N1 - X1\_g \* koh\_1 \* OH dX1\_aq <- N1 |
| --- | --- |
| X2 | X2\_g(t+1) = X2\_g(t) + X1\_g(t) \* k\_OH(1) \* OH \* 1/2 – N2(t) X2\_aq(t+1) = X2\_aq(t) + N2(t) – X2\_aq(t) \* k\_H2O(2) |
| X3 | X3\_aq(t+1) = X3\_aq(t) + X2\_aq(t) \* k\_H2O(2) \* ½ |
| X4 | X4\_g(t+1) = X4\_g(t) + X1\_g(t) \* k\_OH(1) \* OH \* 1/2 – N4(t) – X4\_g(t) \* k\_hv(4) X4\_aq(t+1) = X4\_aq(t) + N4(t) |
| X5 | X5\_g(t+1) = X5\_g(t) + X4\_g(t) \* k\_hv(4) \* 1/3 – N5(t) X5\_aq(t+1) = X5\_aq(t) + N5(t) – X5\_aq(t) \* k\_H2O(5) |
| X6 | X6(t+1) = X6(t) + X2\_aq(t) \* k\_H2O(2) \* 1/2 + X4\_g(t) \* k\_hv(4) \* 1/3 + X5\_aq(t) \* k\_H2O(5) \* 2 / 3 |
| X7 | X7 (t+1) = X7(t) + X4\_g(t) \* k\_hv(4) \* 1/3 + X5\_aq(t) \* k\_H2O(5) \* 1 / 3 |

## Slide 32
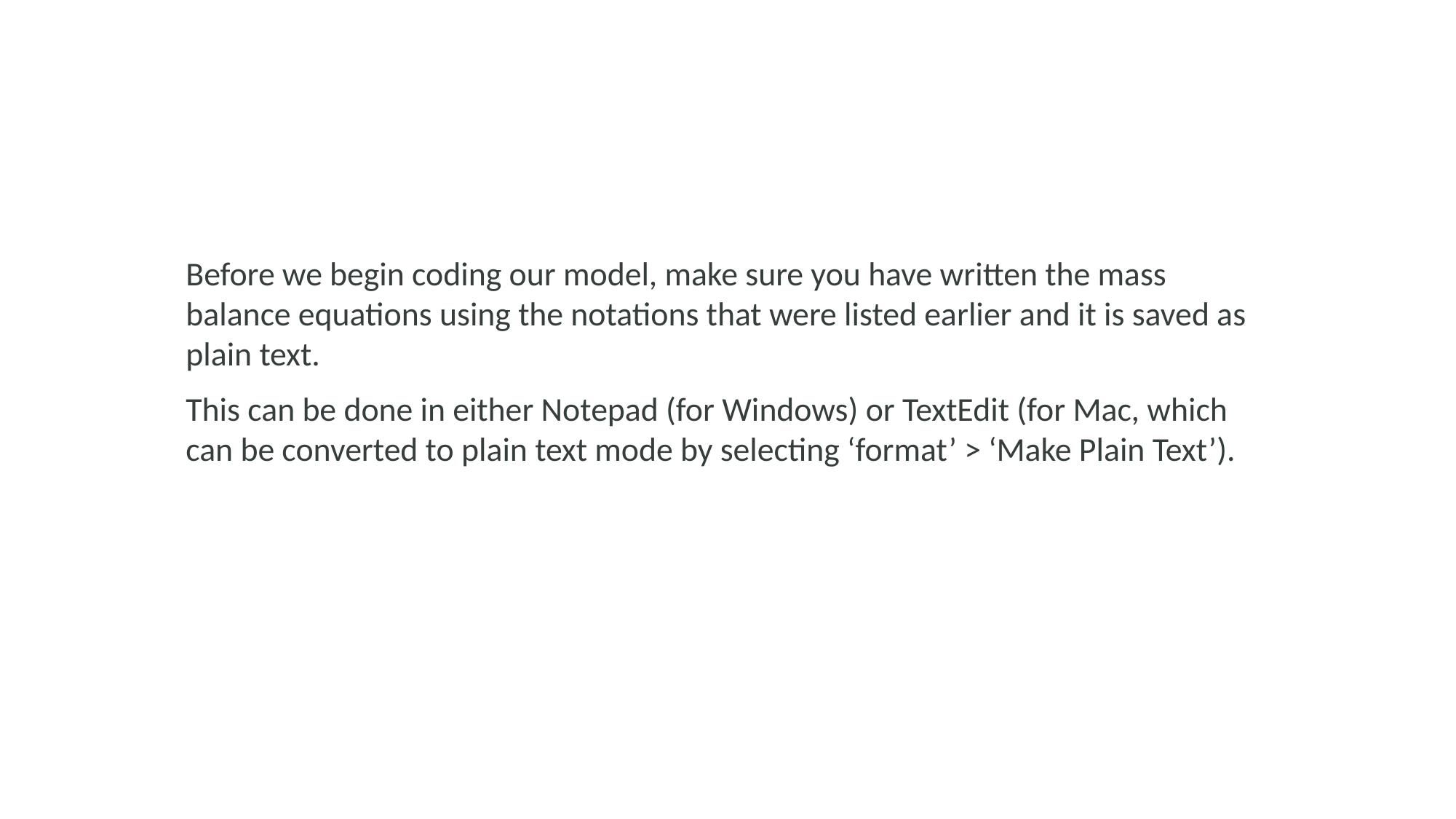

Before we begin coding our model, make sure you have written the mass balance equations using the notations that were listed earlier and it is saved as plain text.
This can be done in either Notepad (for Windows) or TextEdit (for Mac, which can be converted to plain text mode by selecting ‘format’ > ‘Make Plain Text’).

## Slide 33
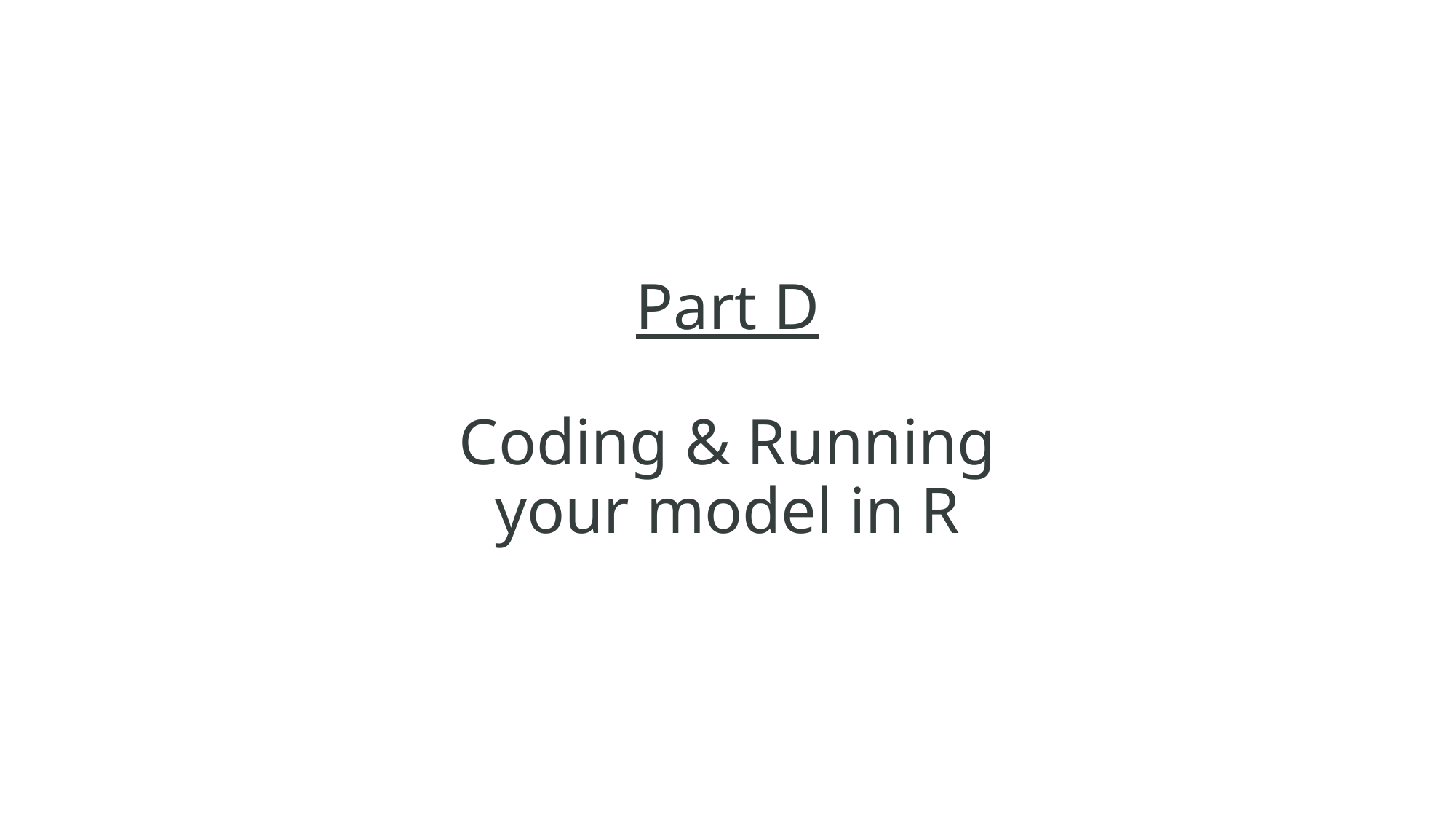

# Part D Coding & Running your model in R

## Slide 34
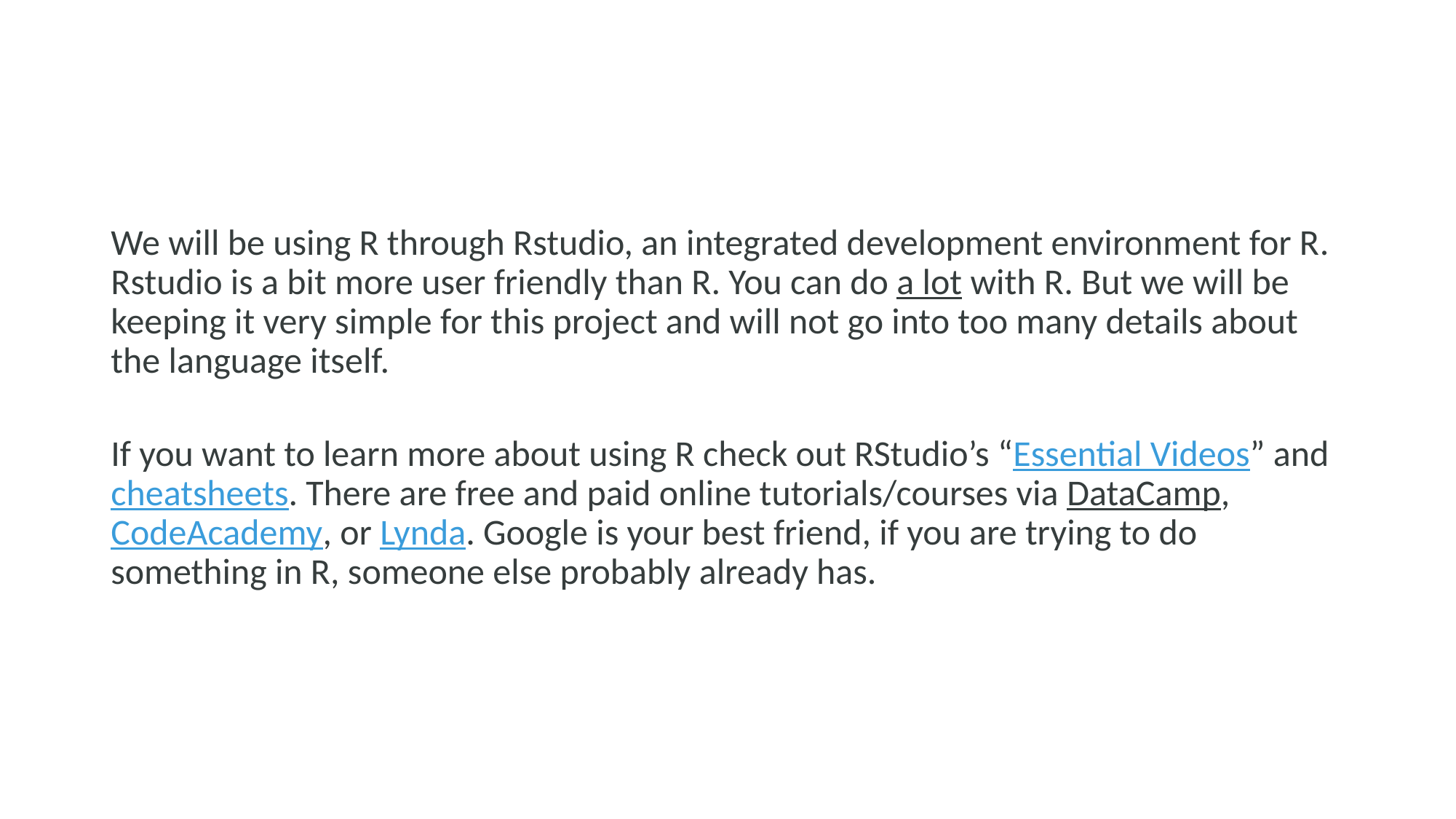

#
We will be using R through Rstudio, an integrated development environment for R. Rstudio is a bit more user friendly than R. You can do a lot with R. But we will be keeping it very simple for this project and will not go into too many details about the language itself.
If you want to learn more about using R check out RStudio’s “Essential Videos” and cheatsheets. There are free and paid online tutorials/courses via DataCamp, CodeAcademy, or Lynda. Google is your best friend, if you are trying to do something in R, someone else probably already has.

## Slide 35
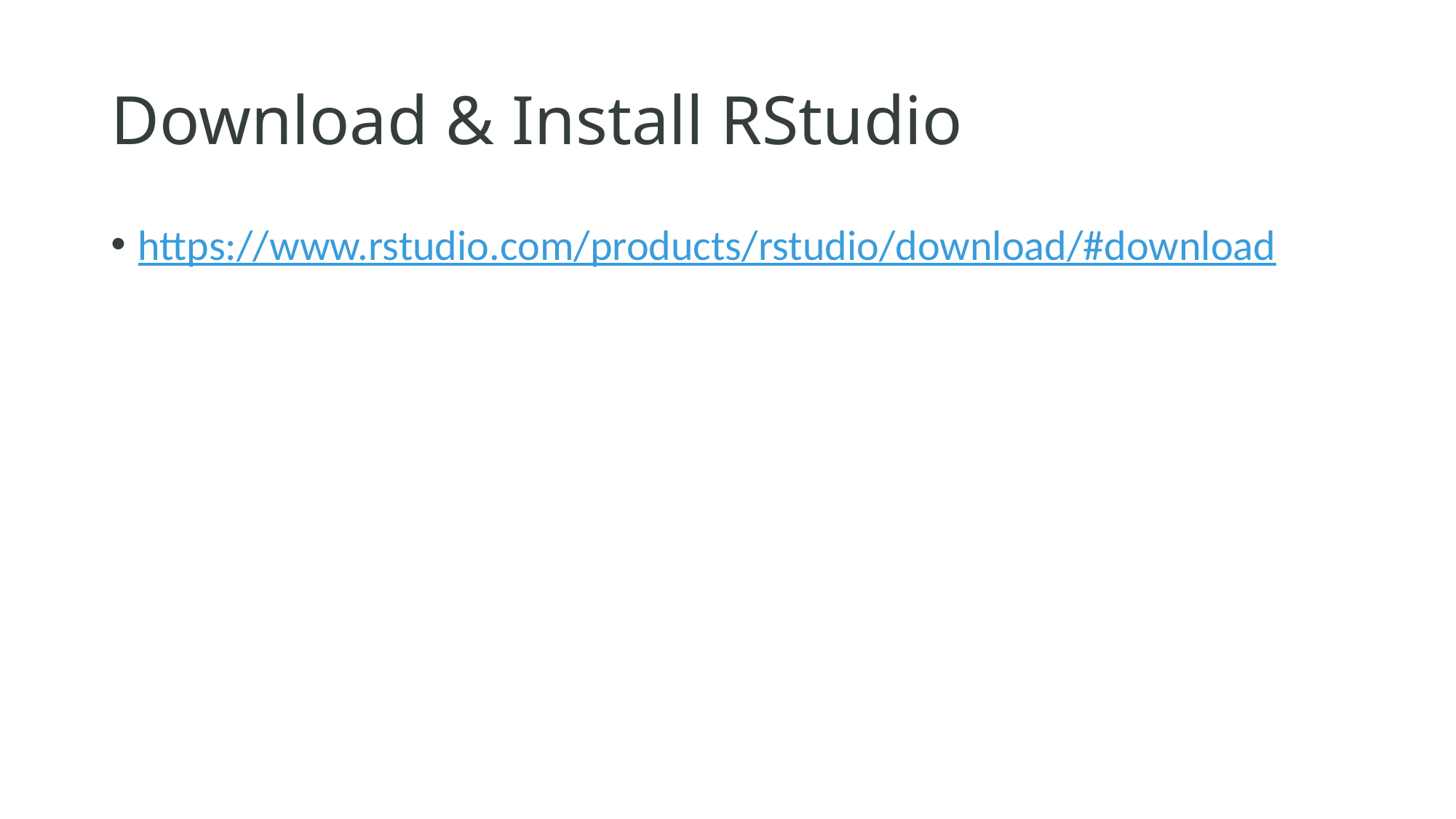

# Download & Install RStudio
https://www.rstudio.com/products/rstudio/download/#download

## Slide 36
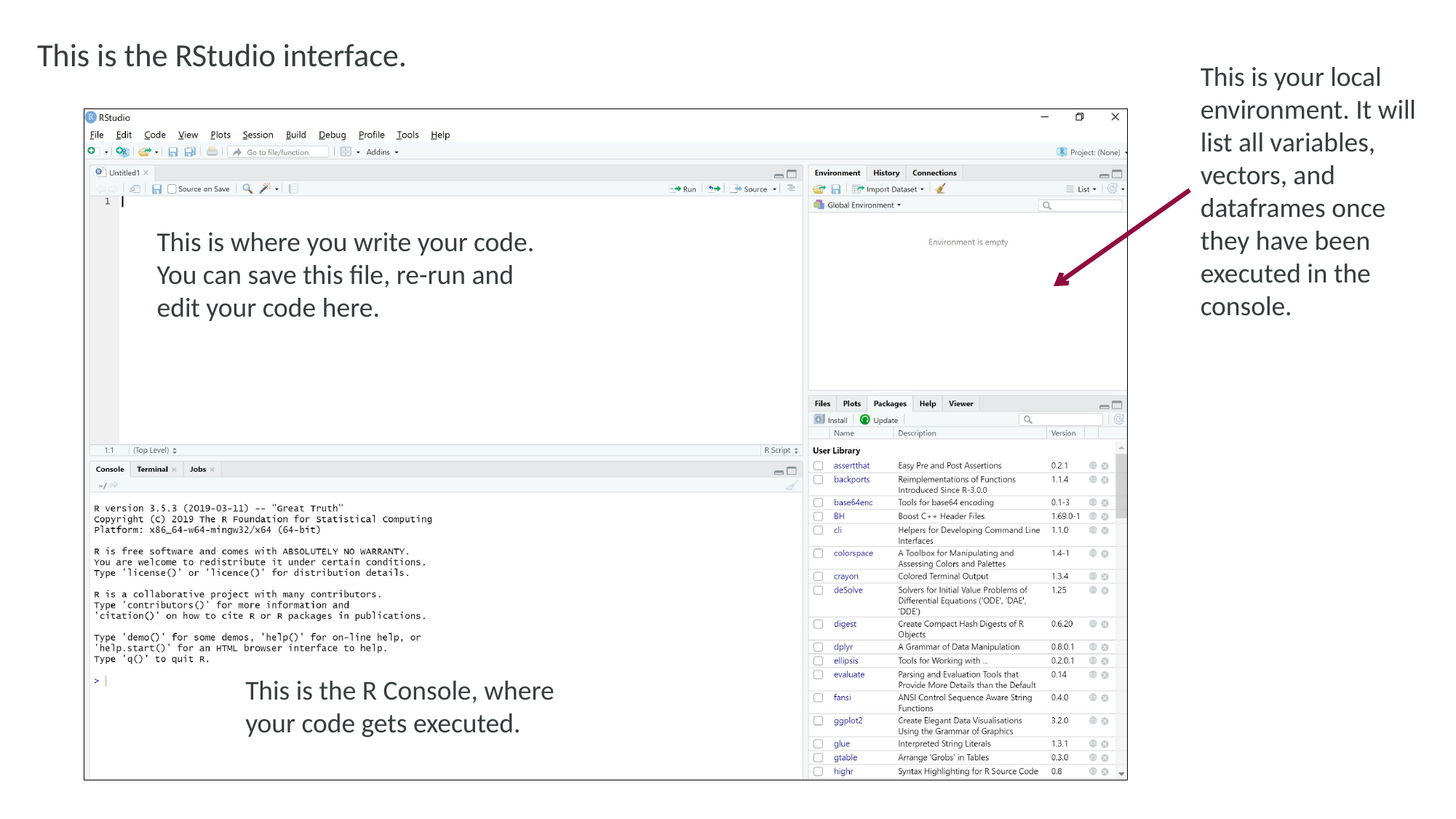

This is the RStudio interface.
This is your local environment. It will list all variables, vectors, and dataframes once they have been executed in the console.
This is where you write your code. You can save this file, re-run and edit your code here.
This is the R Console, where your code gets executed.

## Slide 37
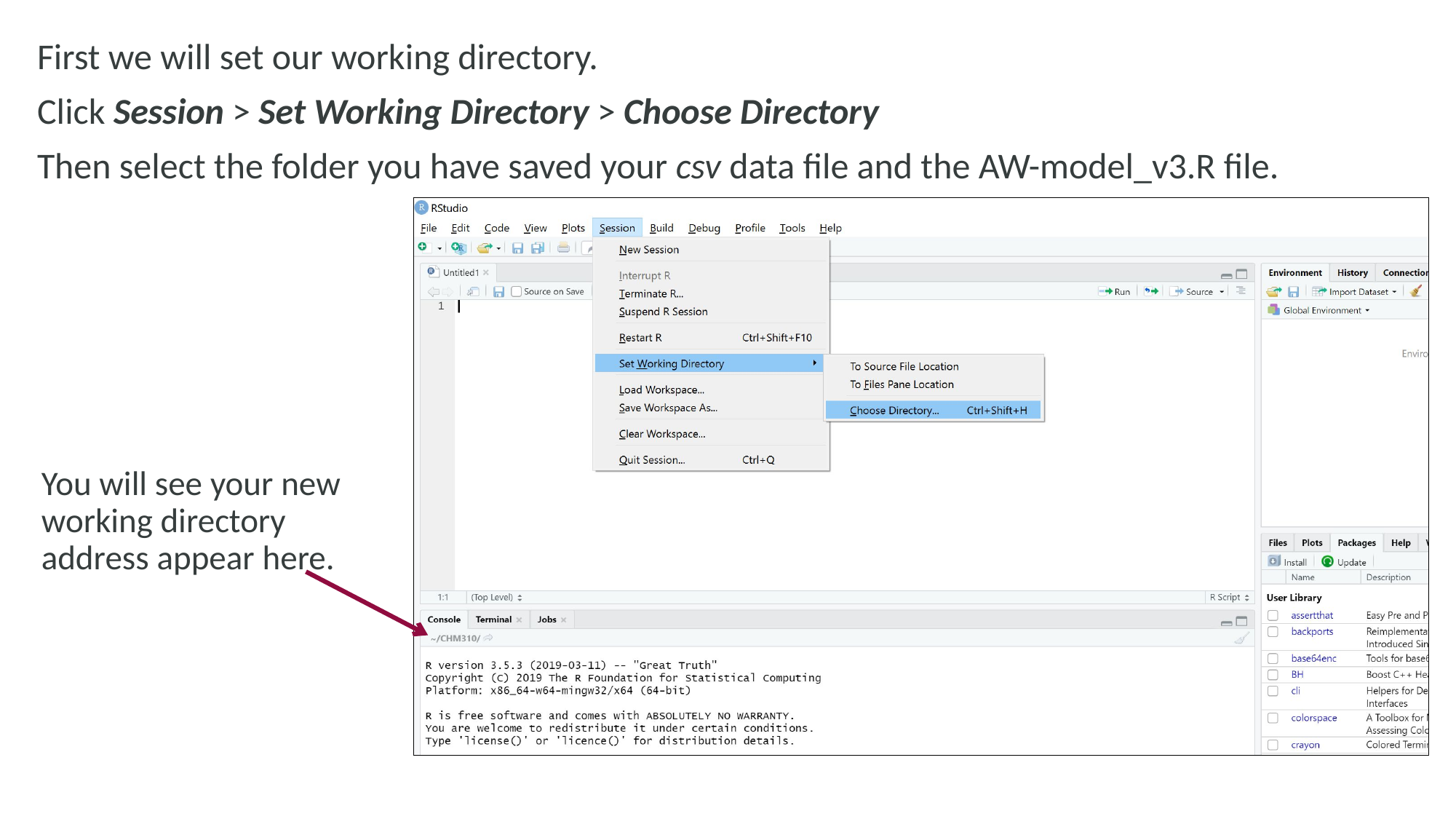

First we will set our working directory.
Click Session > Set Working Directory > Choose Directory
Then select the folder you have saved your csv data file and the AW-model_v3.R file.
You will see your new working directory address appear here.

## Slide 38
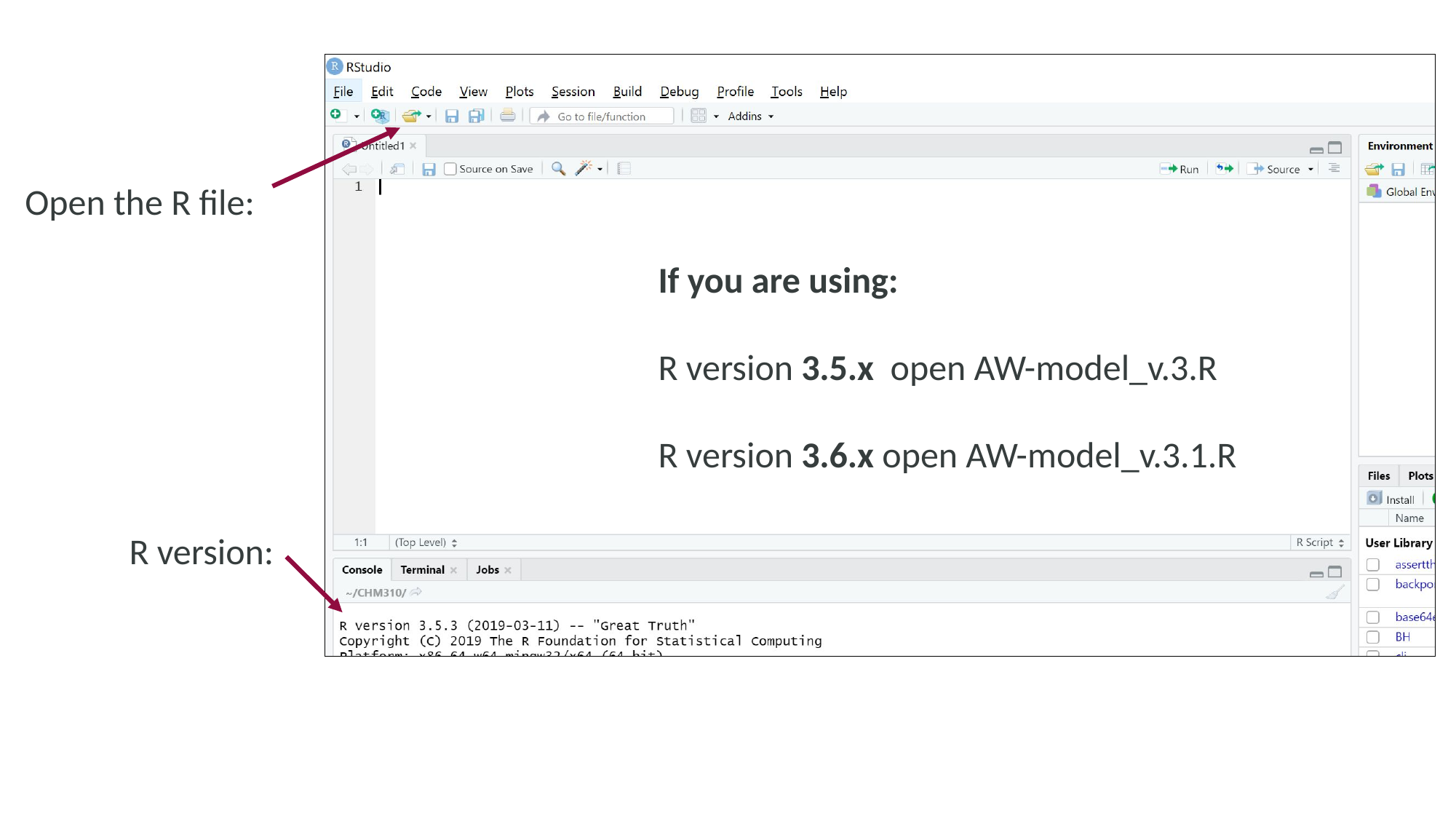

Open the R file:
If you are using:
R version 3.5.x open AW-model_v.3.R
R version 3.6.x open AW-model_v.3.1.R
R version:

## Slide 39
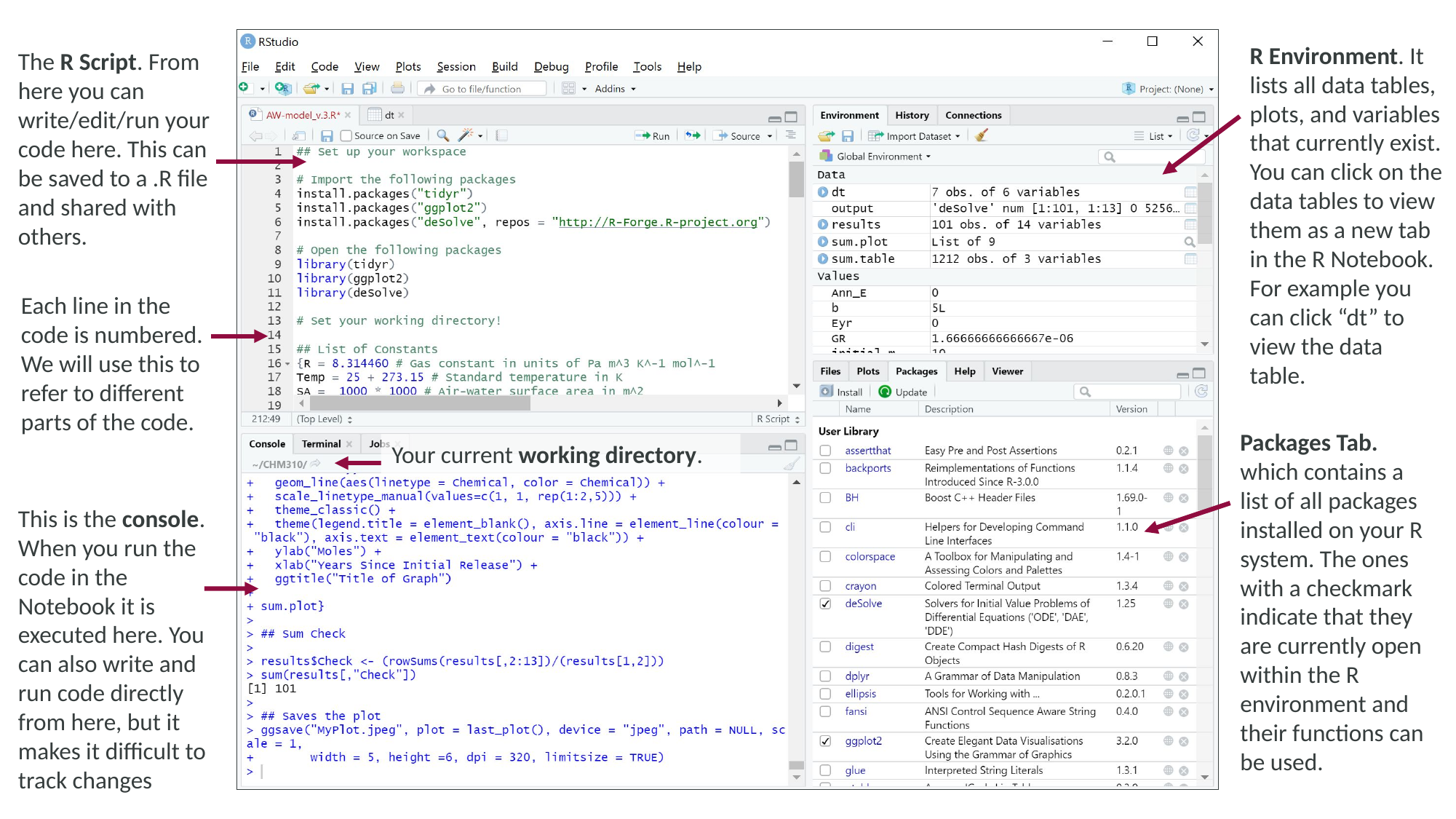

R Environment. It lists all data tables, plots, and variables that currently exist.
You can click on the data tables to view them as a new tab in the R Notebook.
For example you can click “dt” to view the data table.
The R Script. From here you can write/edit/run your code here. This can be saved to a .R file and shared with others.
Each line in the code is numbered. We will use this to refer to different parts of the code.
Packages Tab. which contains a list of all packages installed on your R system. The ones with a checkmark indicate that they are currently open within the R environment and their functions can be used.
Your current working directory.
This is the console. When you run the code in the Notebook it is executed here. You can also write and run code directly from here, but it makes it difficult to track changes

## Slide 40
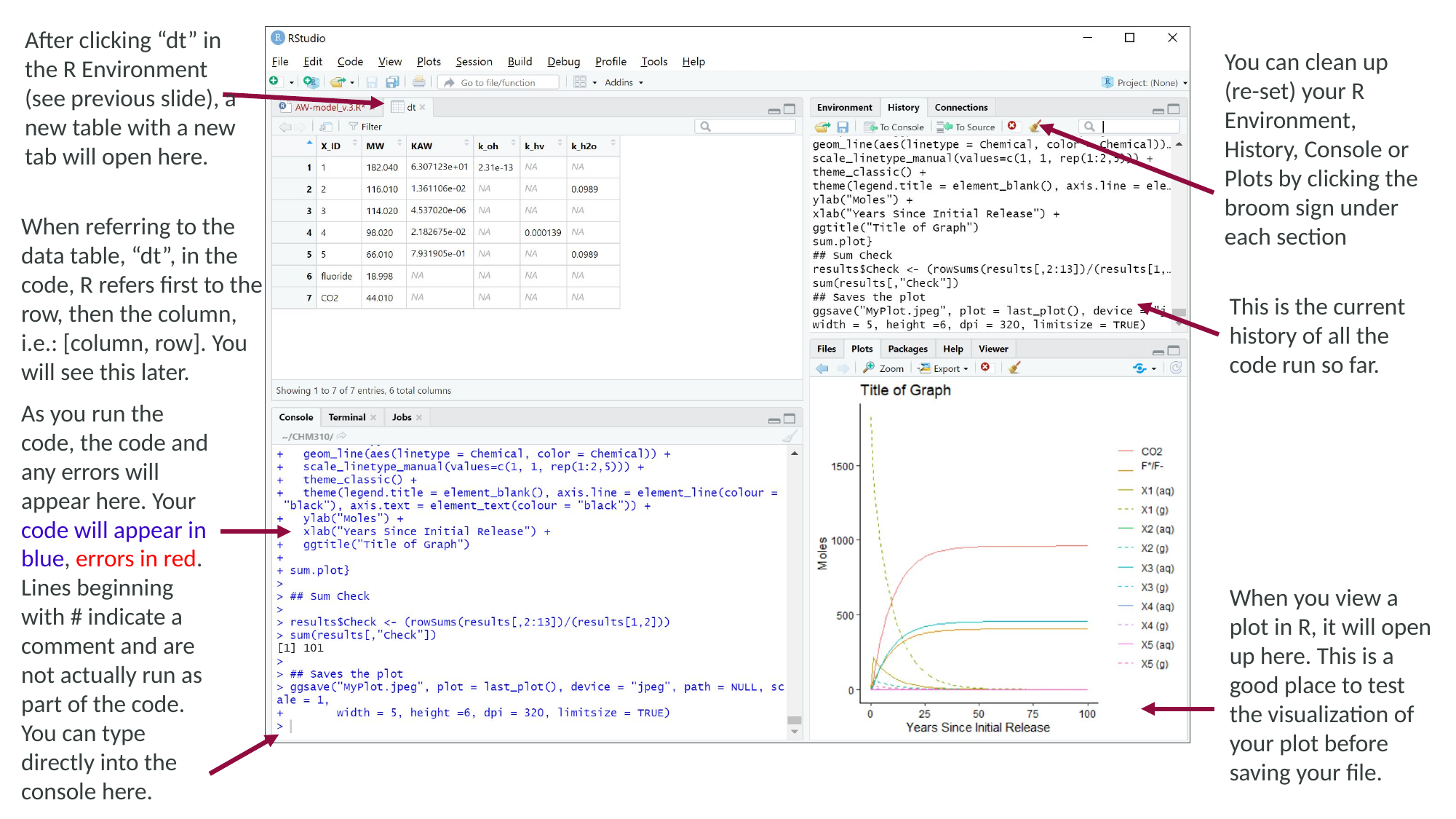

After clicking “dt” in the R Environment (see previous slide), a new table with a new tab will open here.
You can clean up (re-set) your R Environment, History, Console or Plots by clicking the broom sign under each section
When referring to the data table, “dt”, in the code, R refers first to the row, then the column, i.e.: [column, row]. You will see this later.
This is the current history of all the code run so far.
As you run the code, the code and any errors will appear here. Your code will appear in blue, errors in red.
Lines beginning with # indicate a comment and are not actually run as part of the code.
You can type directly into the console here.
When you view a plot in R, it will open up here. This is a good place to test the visualization of your plot before saving your file.

## Slide 41
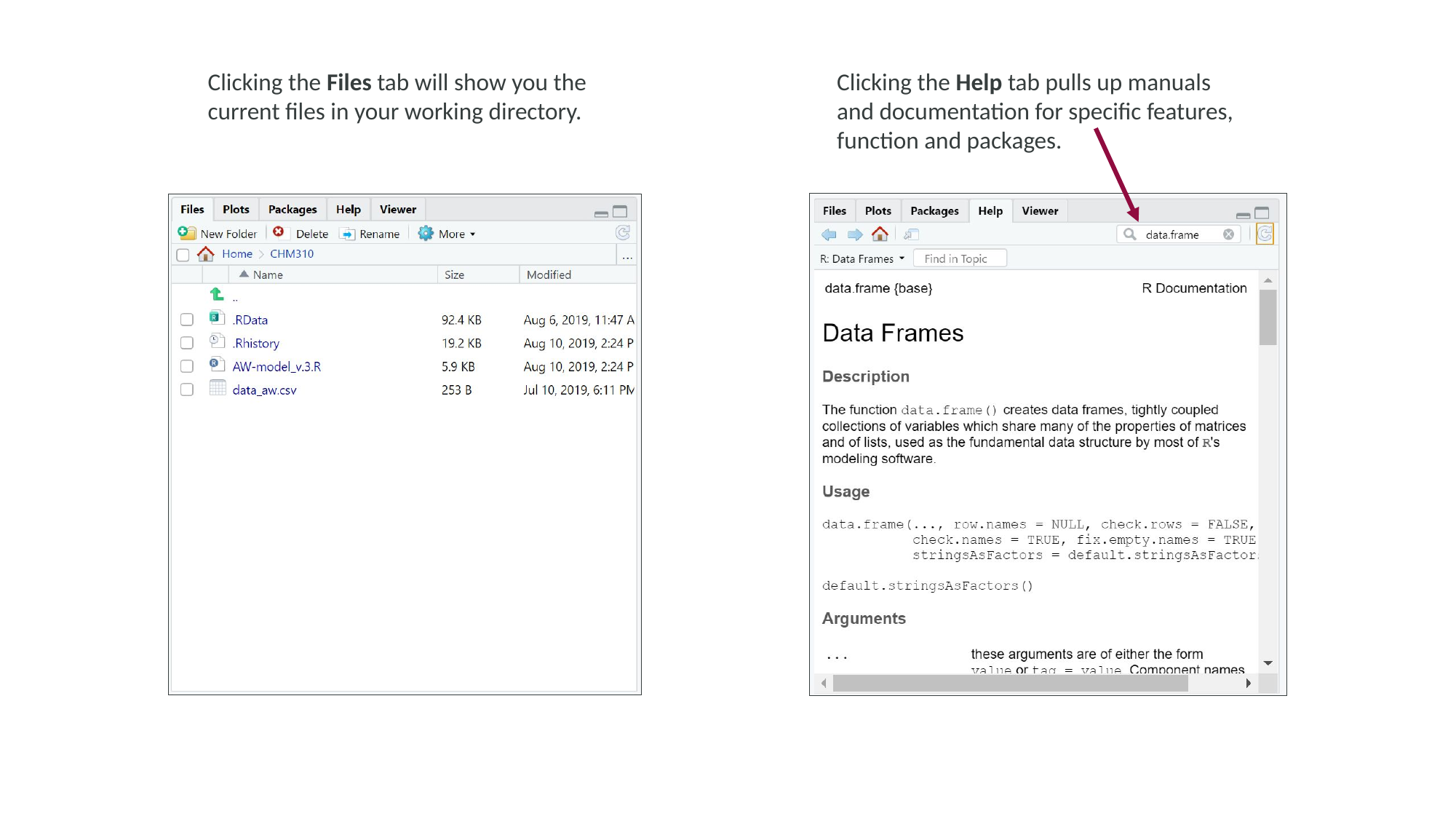

Clicking the Files tab will show you the current files in your working directory.
Clicking the Help tab pulls up manuals and documentation for specific features, function and packages.

## Slide 42
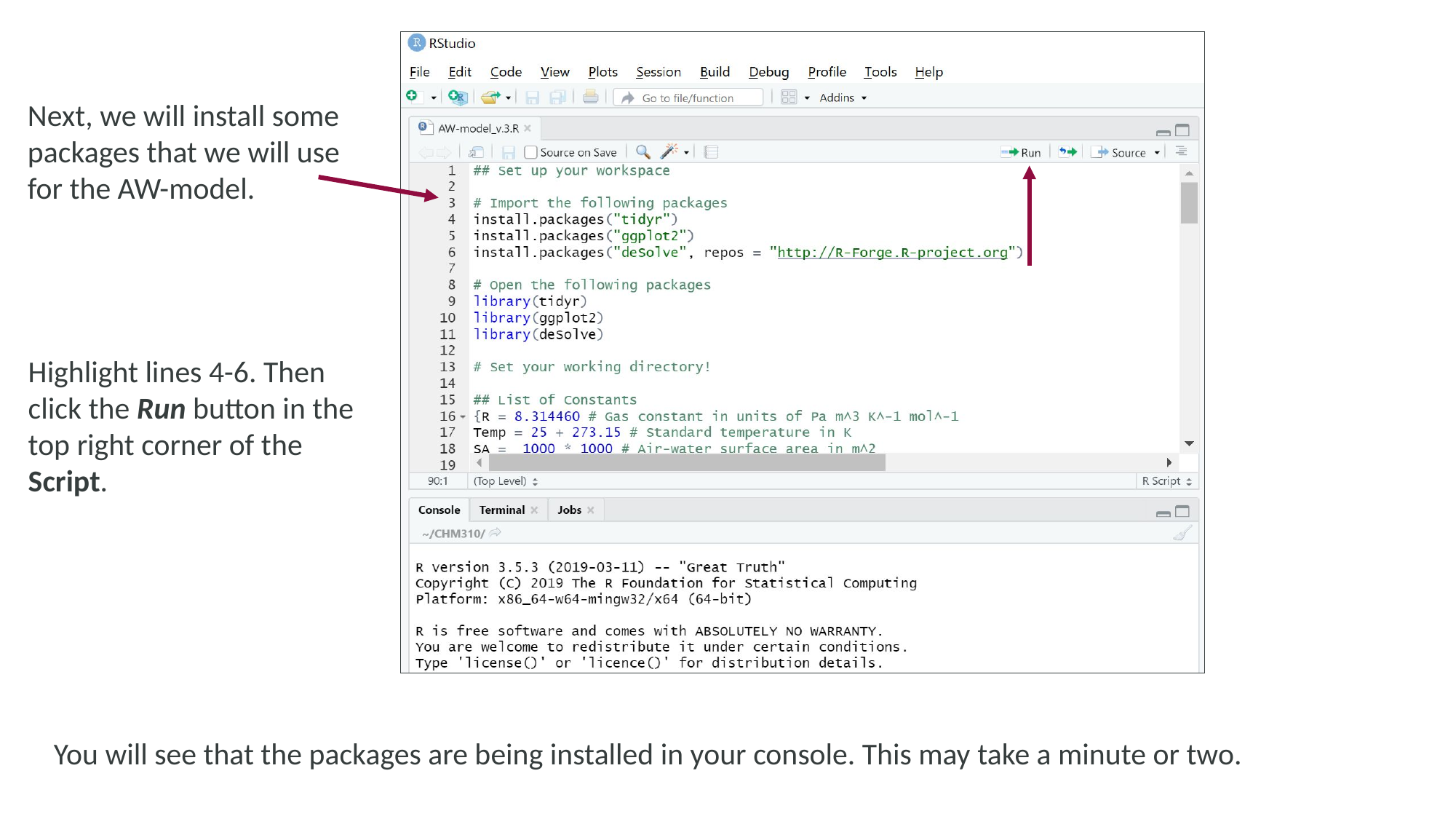

Next, we will install some packages that we will use for the AW-model.
Highlight lines 4-6. Then click the Run button in the top right corner of the Script.
You will see that the packages are being installed in your console. This may take a minute or two.

## Slide 43
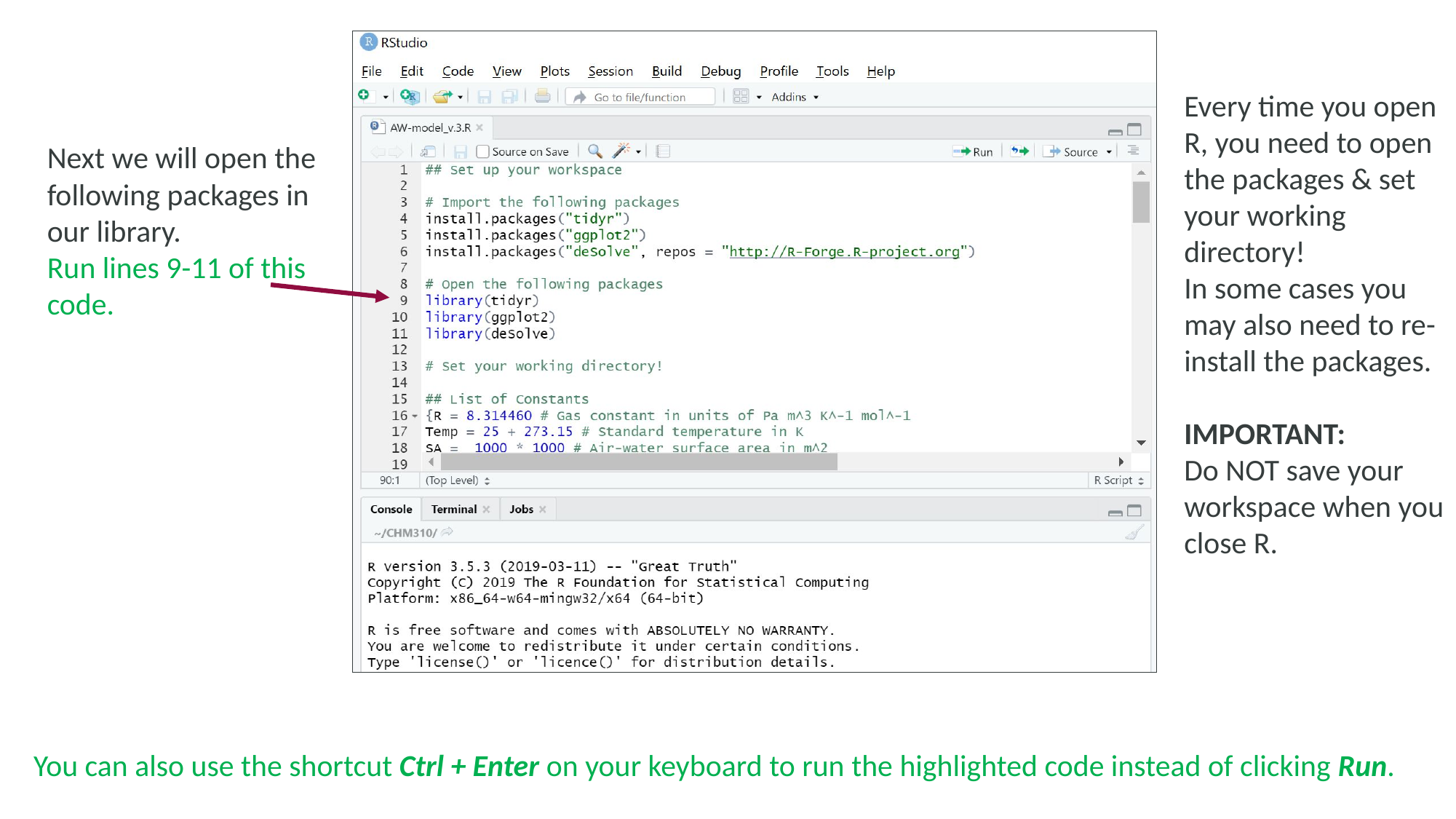

Every time you open R, you need to open the packages & set your working directory!
In some cases you may also need to re-install the packages.
IMPORTANT:
Do NOT save your workspace when you close R.
Next we will open the following packages in our library.
Run lines 9-11 of this code.
You can also use the shortcut Ctrl + Enter on your keyboard to run the highlighted code instead of clicking Run.

## Slide 44
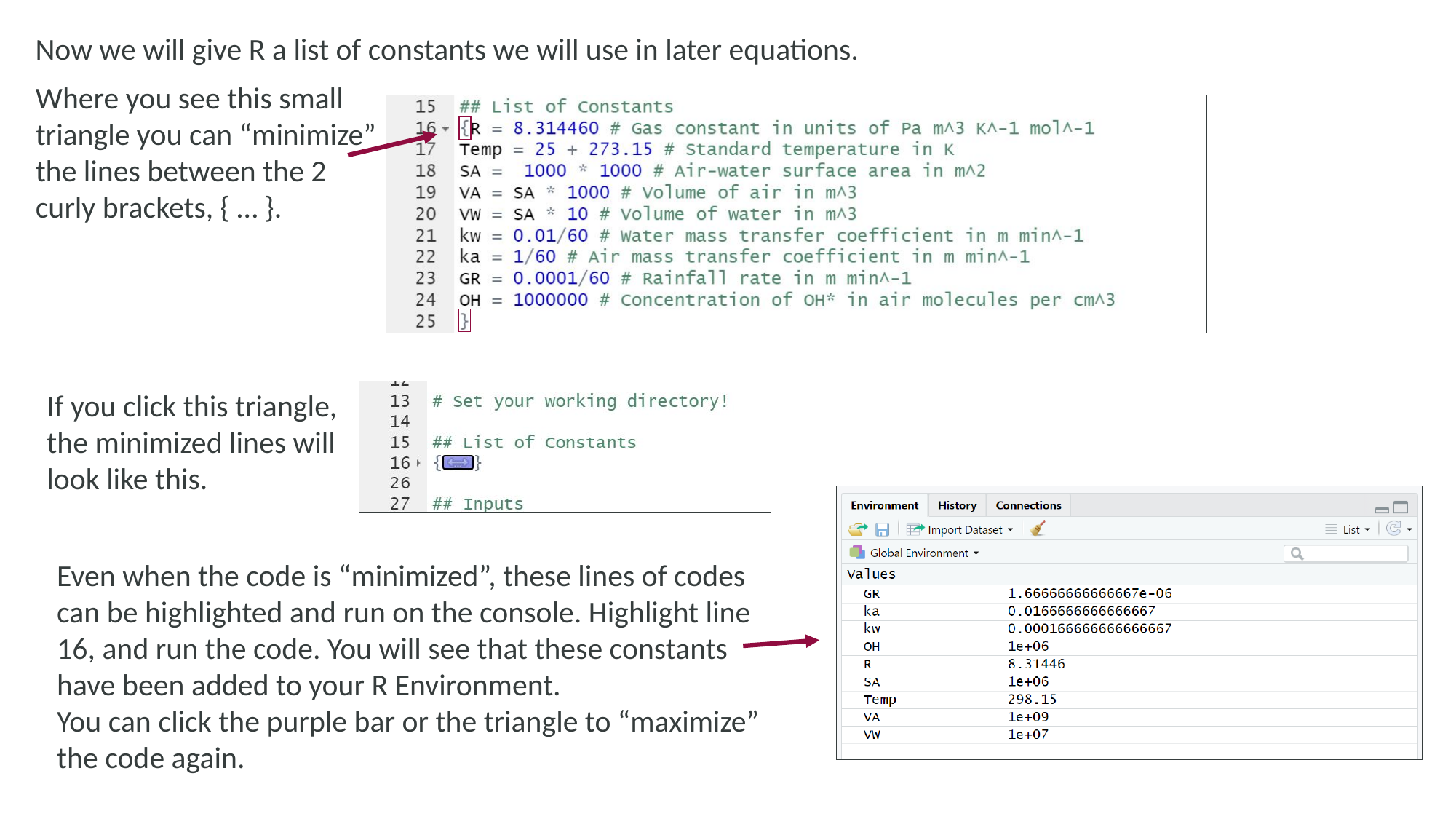

Now we will give R a list of constants we will use in later equations.
Where you see this small triangle you can “minimize” the lines between the 2 curly brackets, { … }.
If you click this triangle, the minimized lines will look like this.
Even when the code is “minimized”, these lines of codes can be highlighted and run on the console. Highlight line 16, and run the code. You will see that these constants have been added to your R Environment.
You can click the purple bar or the triangle to “maximize” the code again.

## Slide 45
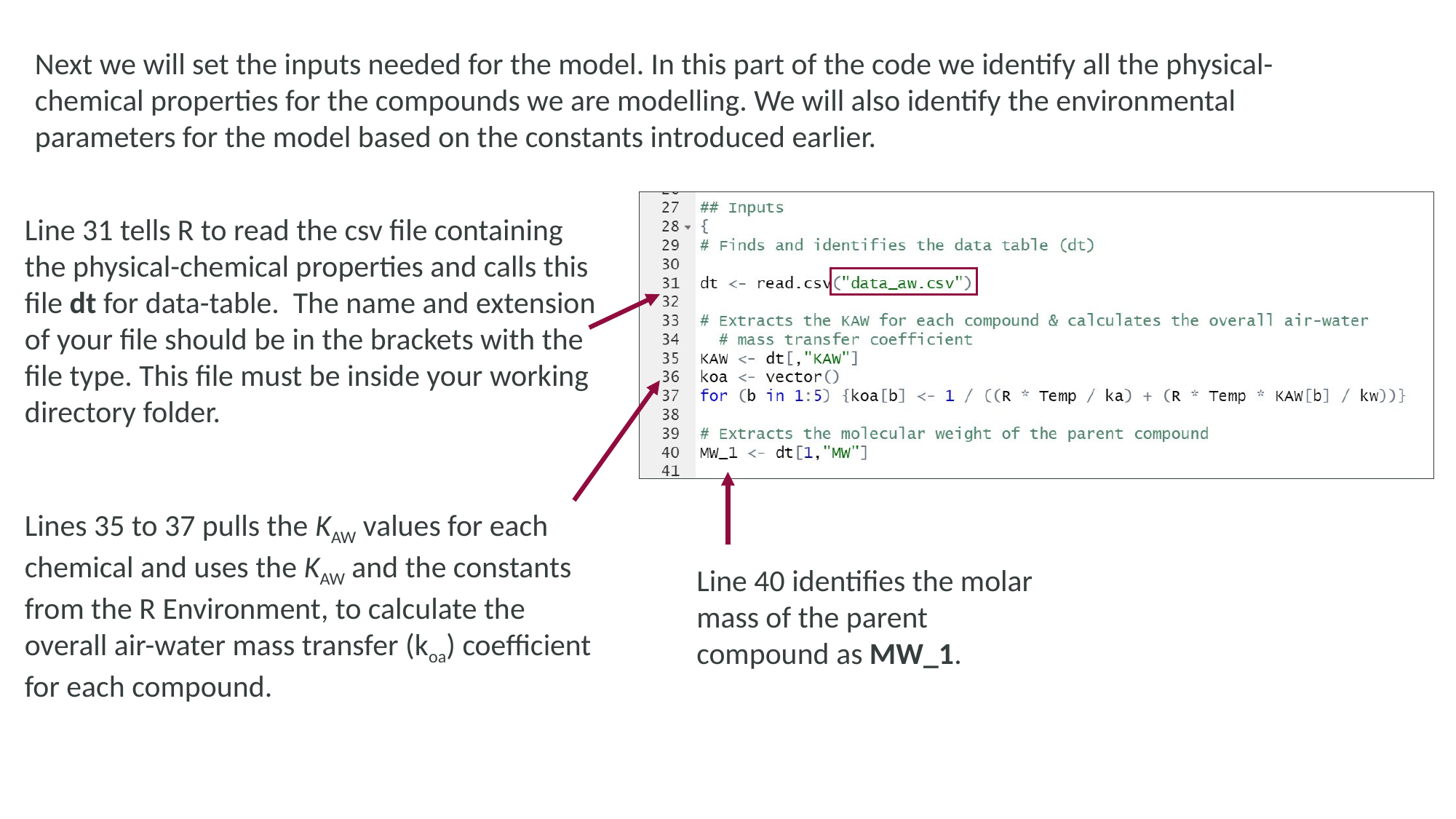

Next we will set the inputs needed for the model. In this part of the code we identify all the physical-chemical properties for the compounds we are modelling. We will also identify the environmental parameters for the model based on the constants introduced earlier.
Line 31 tells R to read the csv file containing the physical-chemical properties and calls this file dt for data-table. The name and extension of your file should be in the brackets with the file type. This file must be inside your working directory folder.
Lines 35 to 37 pulls the KAW values for each chemical and uses the KAW and the constants from the R Environment, to calculate the overall air-water mass transfer (koa) coefficient for each compound.
Line 40 identifies the molar mass of the parent compound as MW_1.

## Slide 46
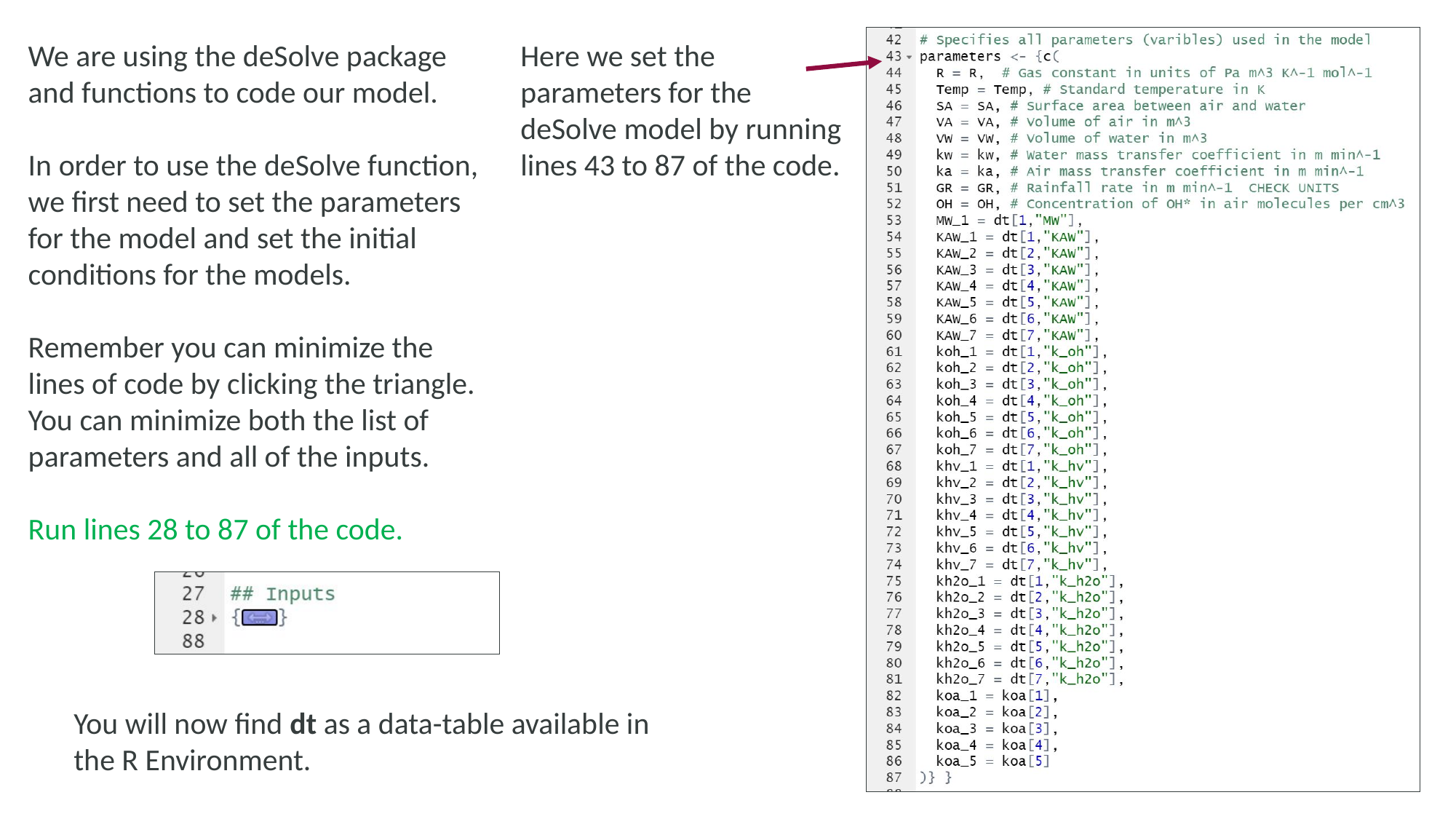

We are using the deSolve package and functions to code our model.
In order to use the deSolve function, we first need to set the parameters for the model and set the initial conditions for the models.
Remember you can minimize the lines of code by clicking the triangle. You can minimize both the list of parameters and all of the inputs.
Run lines 28 to 87 of the code.
Here we set the parameters for the deSolve model by running lines 43 to 87 of the code.
You will now find dt as a data-table available in the R Environment.

## Slide 47
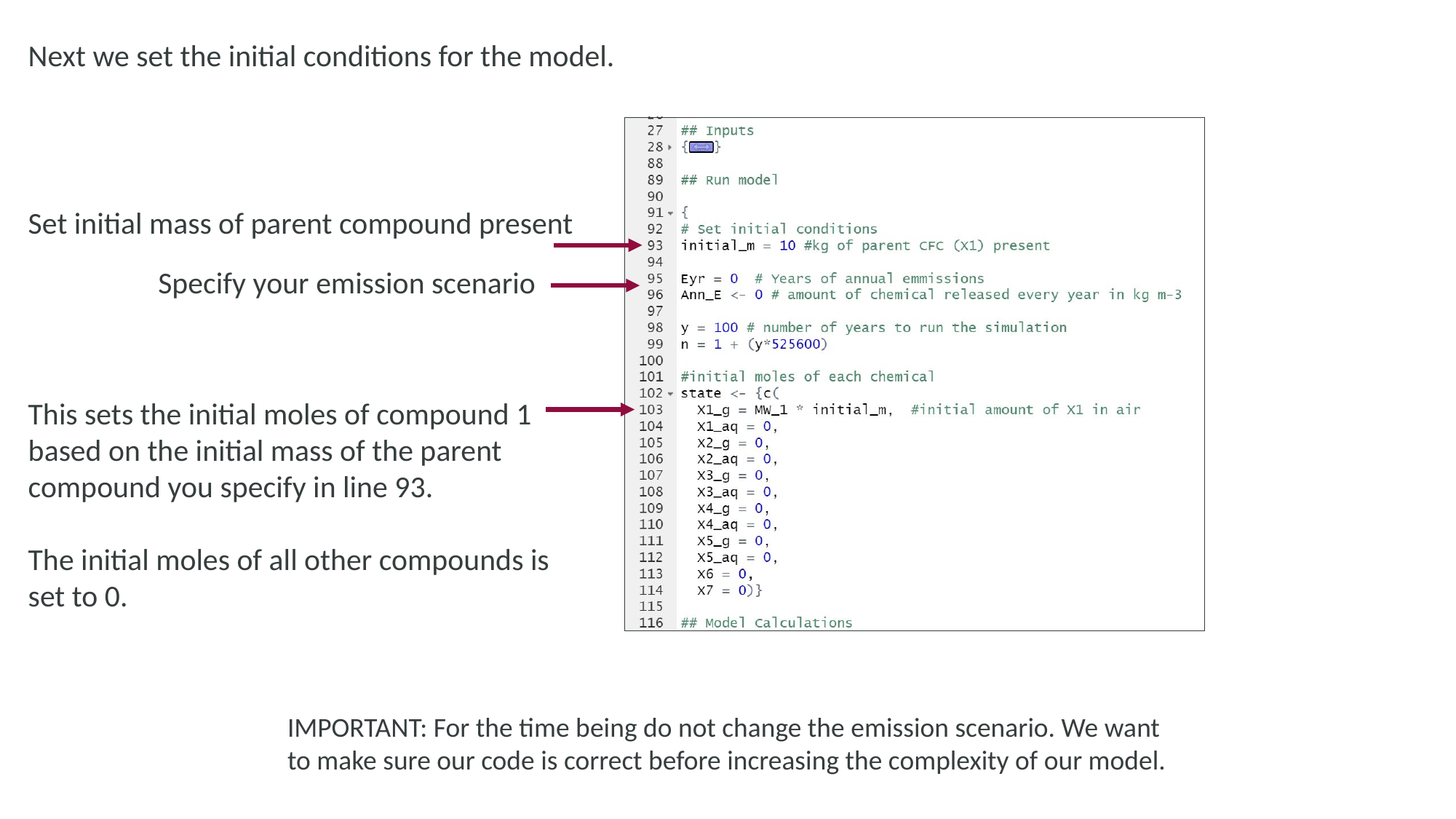

Next we set the initial conditions for the model.
Set initial mass of parent compound present
Specify your emission scenario
This sets the initial moles of compound 1 based on the initial mass of the parent compound you specify in line 93.
The initial moles of all other compounds is set to 0.
IMPORTANT: For the time being do not change the emission scenario. We want to make sure our code is correct before increasing the complexity of our model.

## Slide 48
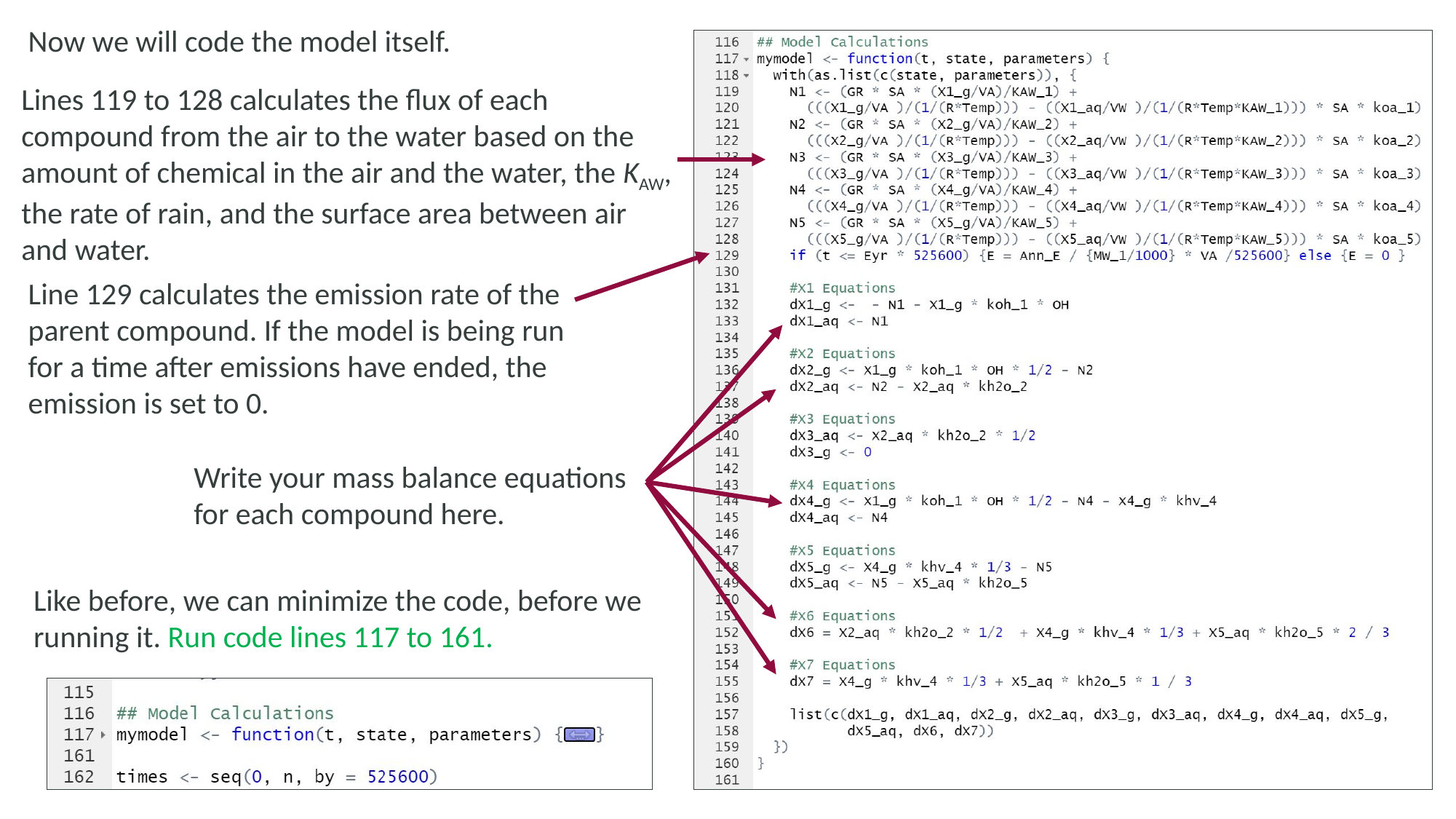

Now we will code the model itself.
Lines 119 to 128 calculates the flux of each compound from the air to the water based on the amount of chemical in the air and the water, the KAW, the rate of rain, and the surface area between air and water.
Line 129 calculates the emission rate of the parent compound. If the model is being run for a time after emissions have ended, the emission is set to 0.
Write your mass balance equations for each compound here.
Like before, we can minimize the code, before we running it. Run code lines 117 to 161.

## Slide 49
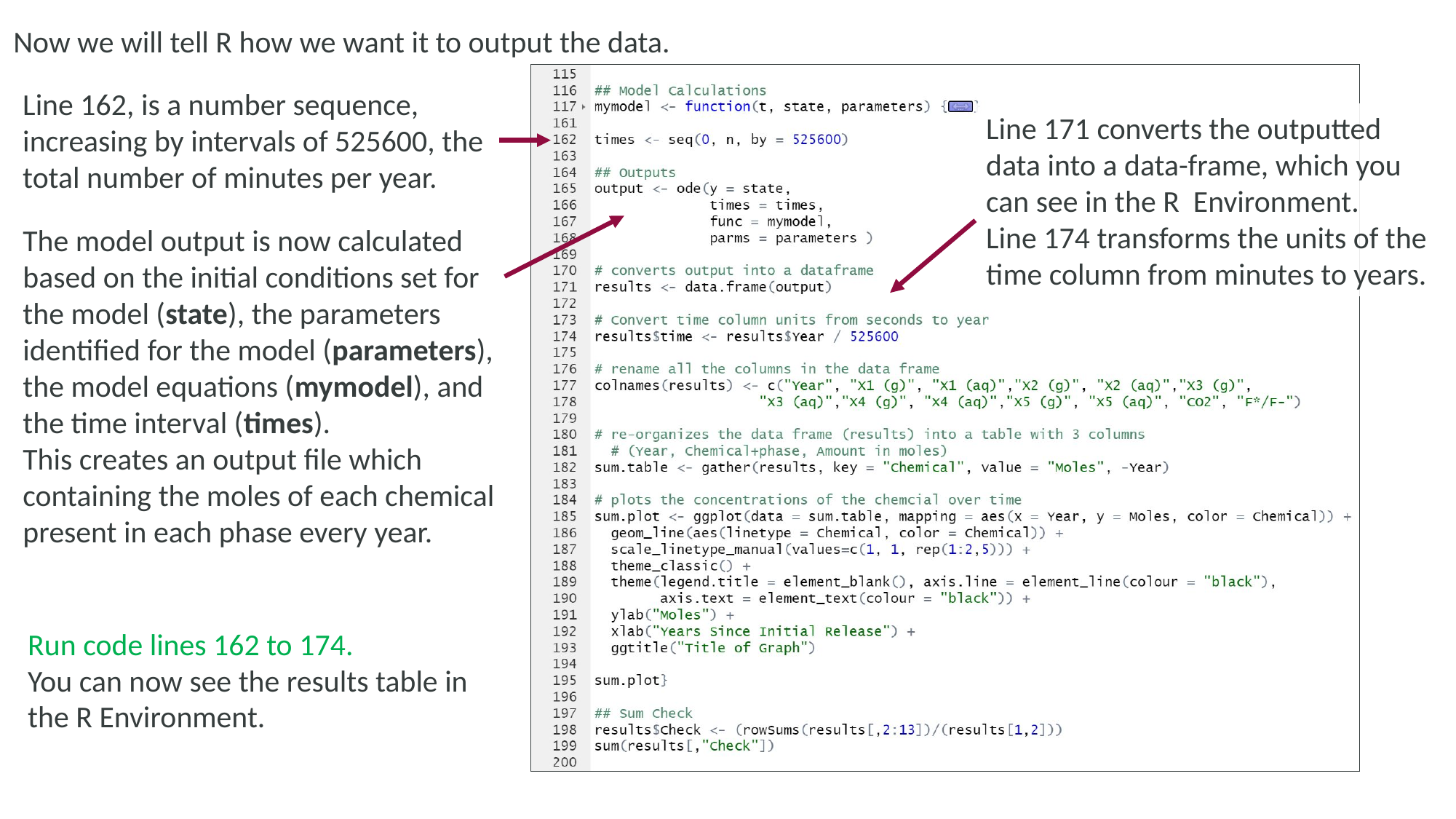

Now we will tell R how we want it to output the data.
Line 162, is a number sequence, increasing by intervals of 525600, the total number of minutes per year.
Line 171 converts the outputted data into a data-frame, which you can see in the R Environment.
Line 174 transforms the units of the time column from minutes to years.
The model output is now calculated based on the initial conditions set for the model (state), the parameters identified for the model (parameters), the model equations (mymodel), and the time interval (times).
This creates an output file which containing the moles of each chemical present in each phase every year.
Run code lines 162 to 174.
You can now see the results table in the R Environment.

## Slide 50
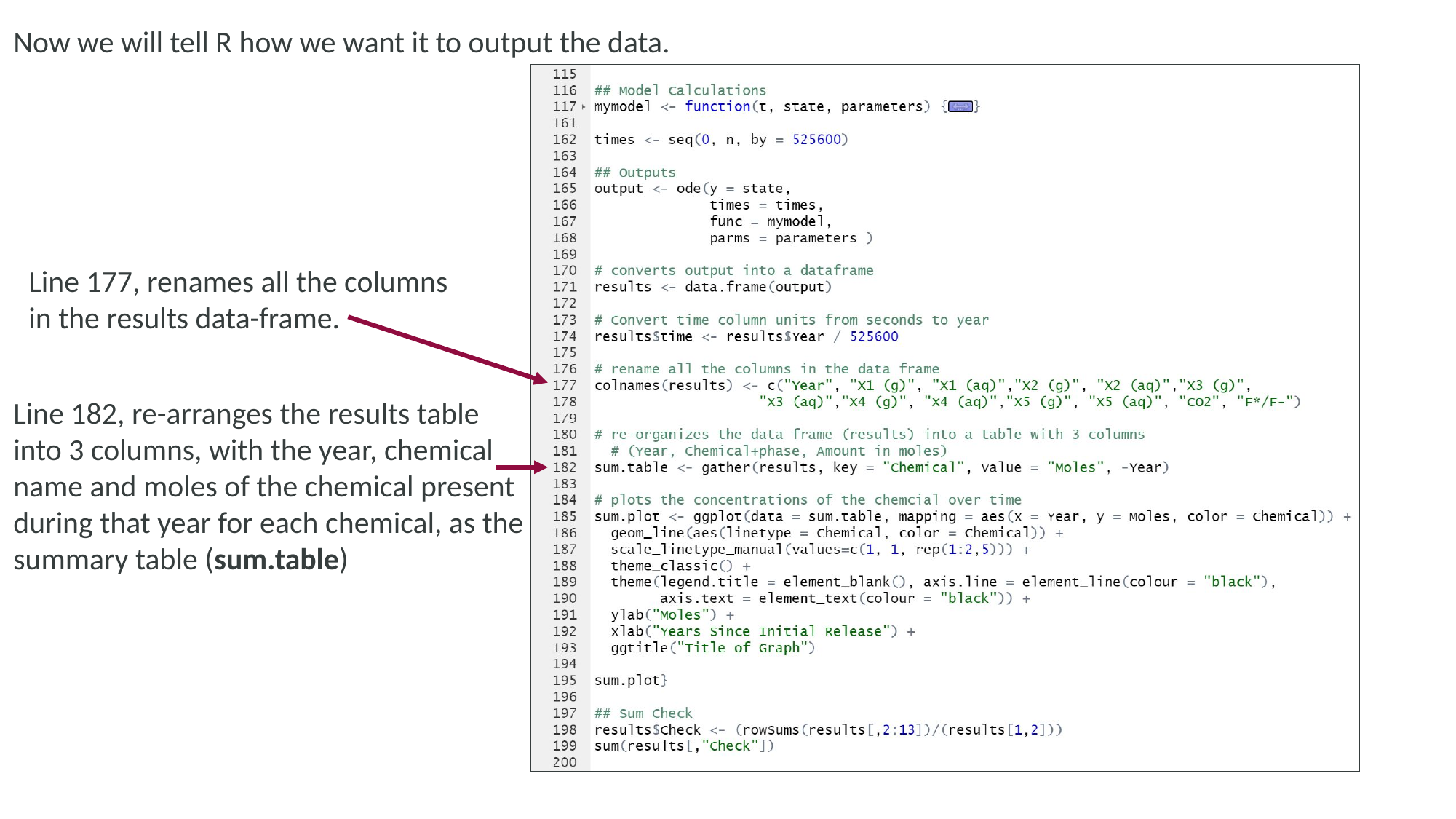

Now we will tell R how we want it to output the data.
Line 177, renames all the columns in the results data-frame.
Line 182, re-arranges the results table into 3 columns, with the year, chemical name and moles of the chemical present during that year for each chemical, as the summary table (sum.table)

## Slide 51
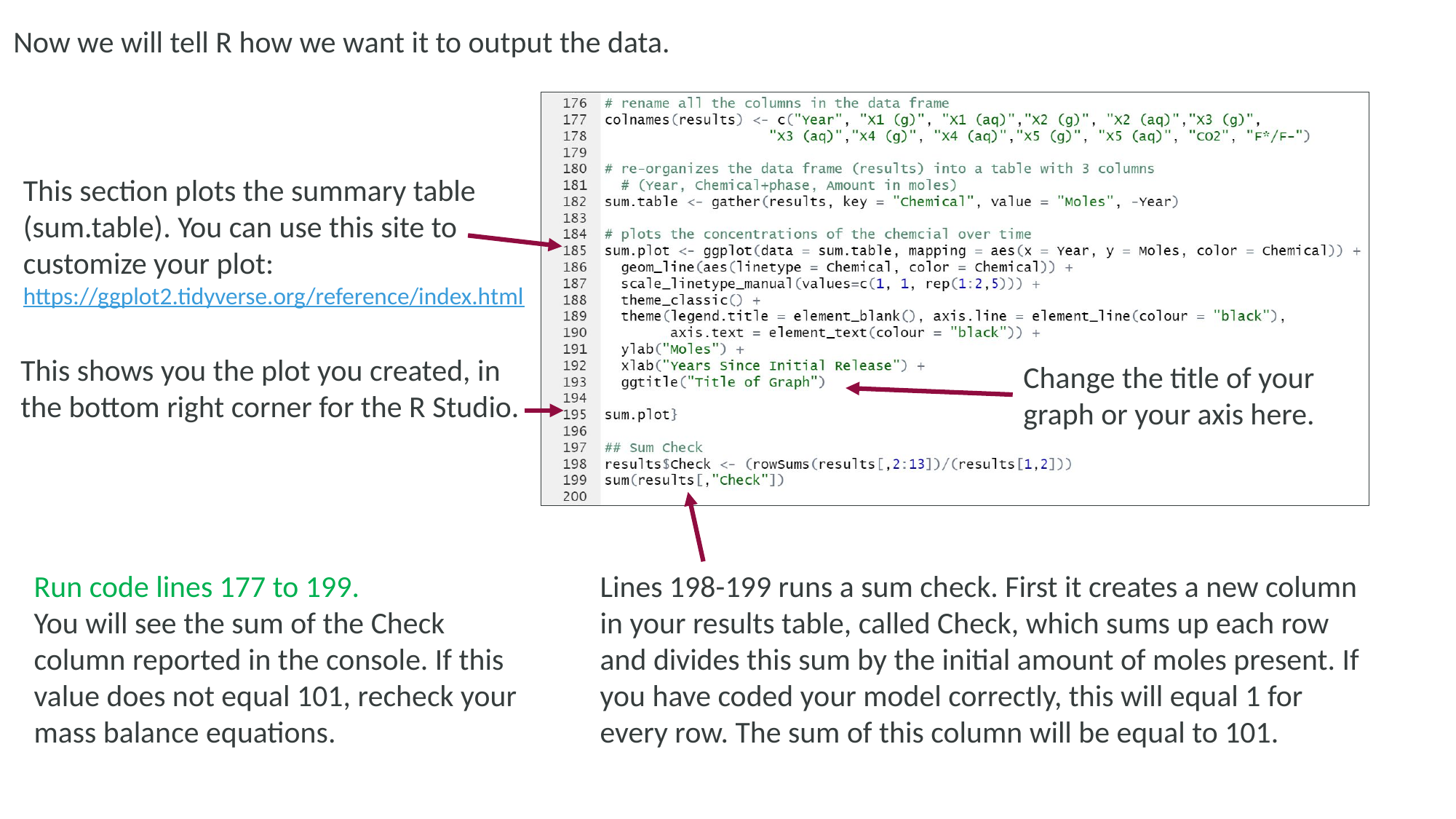

Now we will tell R how we want it to output the data.
This section plots the summary table (sum.table). You can use this site to customize your plot:
https://ggplot2.tidyverse.org/reference/index.html
This shows you the plot you created, in the bottom right corner for the R Studio.
Change the title of your graph or your axis here.
Run code lines 177 to 199.
You will see the sum of the Check column reported in the console. If this value does not equal 101, recheck your mass balance equations.
Lines 198-199 runs a sum check. First it creates a new column in your results table, called Check, which sums up each row and divides this sum by the initial amount of moles present. If you have coded your model correctly, this will equal 1 for every row. The sum of this column will be equal to 101.

## Slide 52
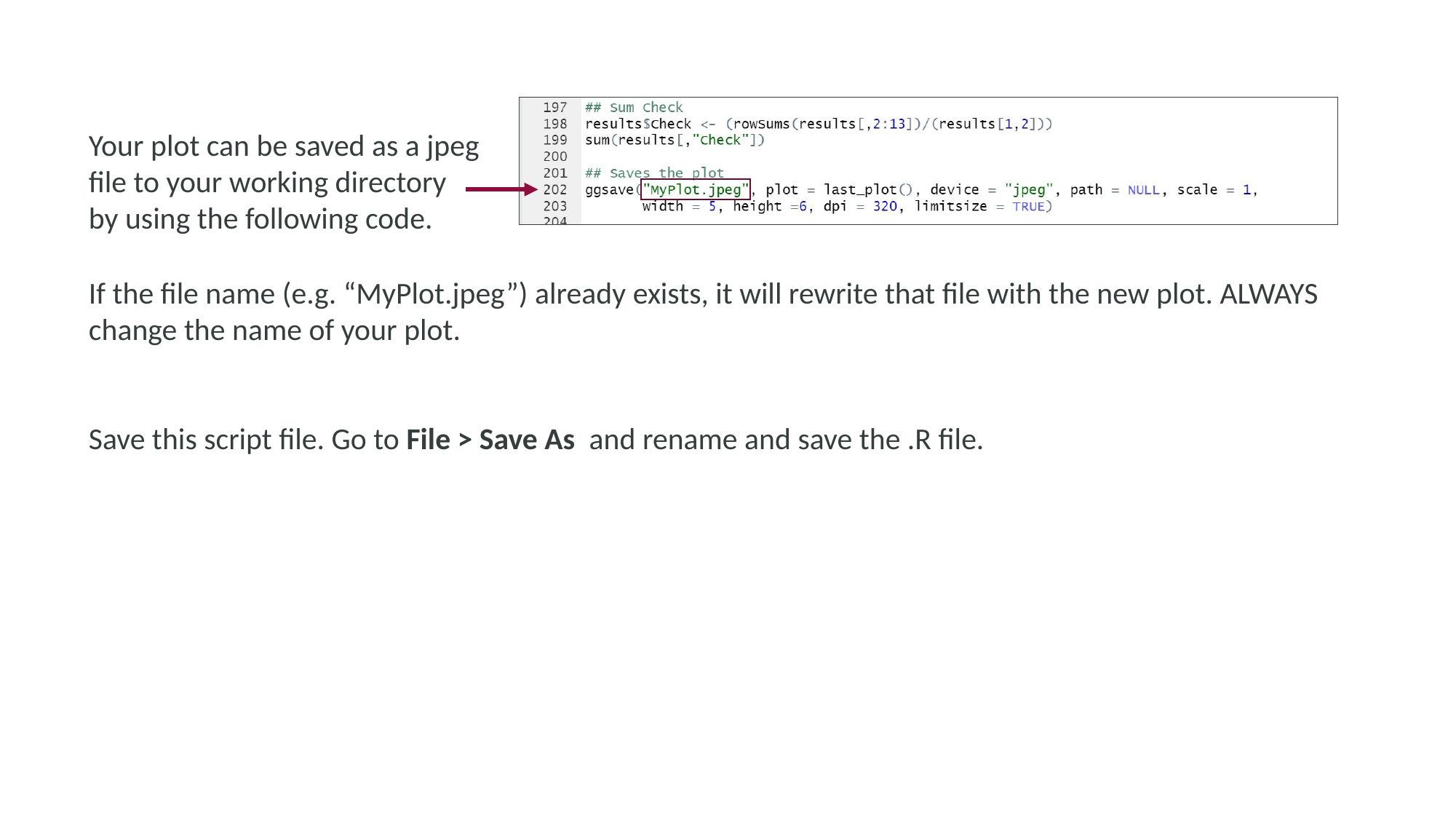

Your plot can be saved as a jpeg file to your working directory by using the following code.
If the file name (e.g. “MyPlot.jpeg”) already exists, it will rewrite that file with the new plot. ALWAYS change the name of your plot.
Save this script file. Go to File > Save As and rename and save the .R file.

## Slide 53
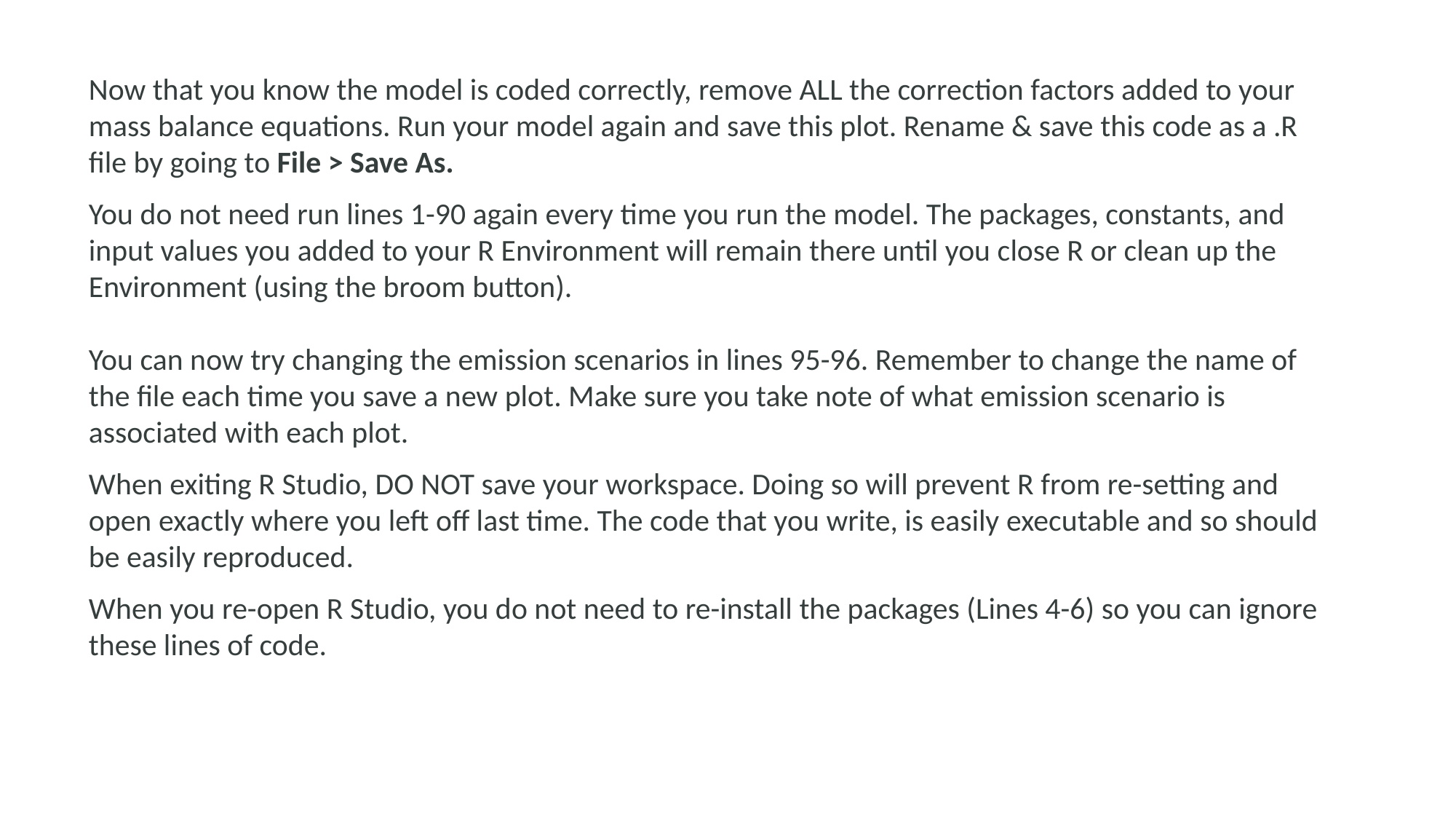

Now that you know the model is coded correctly, remove ALL the correction factors added to your mass balance equations. Run your model again and save this plot. Rename & save this code as a .R file by going to File > Save As.
You do not need run lines 1-90 again every time you run the model. The packages, constants, and input values you added to your R Environment will remain there until you close R or clean up the Environment (using the broom button).
You can now try changing the emission scenarios in lines 95-96. Remember to change the name of the file each time you save a new plot. Make sure you take note of what emission scenario is associated with each plot.
When exiting R Studio, DO NOT save your workspace. Doing so will prevent R from re-setting and open exactly where you left off last time. The code that you write, is easily executable and so should be easily reproduced.
When you re-open R Studio, you do not need to re-install the packages (Lines 4-6) so you can ignore these lines of code.
