# Supplementary material for: Welcome to 310 Environmental Working Group! A Group Project That Places Students in the Role of Consultants Helping Businesses Choose the Most Climate Friendly Fluorinated Gas
Source: J Chem Educ. 2024 Sep 6;101(10):4203–13. doi: 10.1021/acs.jchemed.4c00479 (PMC11465463; doi:10.1021/acs.jchemed.4c00479)
Supplement: Supplementary file 1 — ed4c00479_si_001.zip [file ed4c00479_si_001.zip › Supporting Information/Assignment 4/310 EWG Assignment 4 Fall 2018 Report Sheet.docx]

**Name:** **Student Number:** **Consulting Group:**

| **Questions** | | **Chemical 1** | **Chemical 2** |
| --- | --- | --- | --- |
| **Q1 (Chemical Structure)** | |  |  |
| **Q2** | **log (D or P)** |  |  |
|  | **C_w_** |  |  |
|  | **VP** |  |  |
| **Q3 (program)** | |  |  |
| **Q4** | **K_AW_** |  |  |
|  | **K_oW_** |  |  |
|  | **K_OA_** |  |  |
| **Q5 (a)**  **Dry Summer Day** | ***m*_air_ (%)** |  |  |
|  | ***m*_water_ (%)** |  |  |
|  | ***m*_organic_ (%)** |  |  |
| **Q5 (b)**  **Thunder-storm** | ***m*_air_ (%)** |  |  |
|  | ***m*_water_ (%)** |  |  |
|  | ***m*_organic_ (%)** |  |  |
| **Q5 (c)**  **Lung** | ***m*_air_ (%)** |  |  |
|  | ***m*_water_ (%)** |  |  |
|  | ***m*_organic_ (%)** |  |  |
| **Q6** | |  |  |
| **Q7** | |  | |
| **Q8** | |  |  |
| **9 (a)** | | Table below | Table below |
| **9 (b)** | | Attach equations | Attach equations |
| **9 (c)** | | Attach graphs | Attach graphs |
| **9 (d) (i)** | |  |  |
| **9 (d) (ii)** | |  |  |

**Table for Question 9(a)**

**Chemical 1**

| **No.** | **CAS** | **Molecular Formula** | **MW**  **(g mol-1)** | **Reaction Type** | **Reaction Rate**  **(min-1)** | **Log KAW** |
| --- | --- | --- | --- | --- | --- | --- |
|  |  |  |  |  |  |  |
|  |  |  |  |  |  |  |
|  |  |  |  |  |  |  |
|  |  |  |  |  |  |  |
|  |  |  |  |  |  |  |

**Chemical 2**

| **No.** | **CAS** | **Molecular Formula** | **MW**  **(g mol-1)** | **Reaction Type** | **Reaction Rate**  **(min-1)** | **Log KAW** |
| --- | --- | --- | --- | --- | --- | --- |
|  |  |  |  |  |  |  |
|  |  |  |  |  |  |  |
|  |  |  |  |  |  |  |
|  |  |  |  |  |  |  |
|  |  |  |  |  |  |  |
