# Supplementary material for: Welcome to 310 Environmental Working Group! A Group Project That Places Students in the Role of Consultants Helping Businesses Choose the Most Climate Friendly Fluorinated Gas
Source: J Chem Educ. 2024 Sep 6;101(10):4203–13. doi: 10.1021/acs.jchemed.4c00479 (PMC11465463; doi:10.1021/acs.jchemed.4c00479)
Supplement: Supplementary file 1 — ed4c00479_si_001.zip [file ed4c00479_si_001.zip › Supporting Information/Assignment 3/310 EWG Assignment 3 Fall 2018.docx]

| 310 Environmental Working Group |  |
| --- | --- |

Assignment 3

Deliverables

**A comprehensive Evaluation of Environmental Fate**

As part of your evaluation of the environmental impact of your assigned chemicals you are not satisfied with using Global Warming Potential as the sole indicator and would like to supplement it with a full evaluation of the environmental fate of both chemicals. In assignment 1 you began this task by calculating the lifetime of the chemicals with respect to atmospheric oxidation. In this assignment you will expand on what you began there and comment on the overall fate of the compounds in the environment. The most effective way to present environmental fate is using a figure or a scheme. The scheme below is an example that describes the fate of CF_3_CF=CHCF_3_. It was made using ChemDraw, which is freely available from the Chemistry Website under the library tab, instructions on how to download and install it are available here:

<http://www.chem.utoronto.ca/library/instructions.php>

**Questions**

For this assignment all solutions can be submitted online, however a hard copy of product generation scheme (Question 4) can be submitted in class. The online submission is done through Quercus by completing the word document entitled “310 EWG Assignment 3 Fall 2018 Report Sheet.docx”. Your submitted report sheet should be renamed as “Surname_FirstName_310EWG_Assign3.docx”.

1. Prepare a figure similar to the one on the first page of this assignment for both of your chemicals. **This figure must be prepared using ChemDraw or similar software** so that it is of sufficient quality to include in your presentation and report. Indicate all closed-shell products (not radicals) using boxes. Continue your reaction scheme until the chemical is mineralized or the resulting product has an environmental lifetime greater than 10 years. Use the table below to predict the lifetime of any potential products. (**4 marks**)

| **Functional Group** | **Reaction** | **First Order Rate Constant or Pseudo First Order Rate Constant** |
| --- | --- | --- |
| Aldehyde | Photolysis | *k_hv(aldehyde)_* = 73 year^-1^ |
| Ketone | Photolysis | *k_hv(ketone)_* = 9.6 year^-1^ |
| Acyl Fluoride | Hydrolysis at pH 7 | *k_OH-/H2O(acyl fluoride)_* = 52,000 year^-1^ |
| Fluorinated Ester | Hydrolysis at pH 7 | *k_OH-/H2O(ester)_* = 0.50 year^-1^ |

1. If your chemical included two sites of reaction, did you get the same set of products from reaction at both sites? (**1 mark**)
2. If you answered yes to question 2 for either of your chemical’s, what role did the ether functional group play in the production of similar products from both reaction sites? (**1 mark**)
3. In Assignment 4 you will build and run a chemical fate model in Excel to understand and visualize the fate of your chemicals and their reaction products. In order to run the model you will need a clear understanding of each generation of reaction products. To do this you will transform your figure from Question 1 into a product generation scheme that identifies the different generations of closed shell chemicals that form as your chemical’s breakdown in the environment. The template for this generation scheme is provided as a word document and PDF with this assignment. A product generation scheme for CF3CF=CHCF3, whose degradation mechanism is provided as an example on the first page, is shown on page 3. **The generation scheme can be completed using the computer and submitted online or submitted as a hard copy in class.**

If your molecule includes two sites of H-abstraction, then prepare the product generation scheme for the site that was identified in Question 3 of Assignment 1 as being the dominate reaction pathway. Making this approximation will simplify the model calculations in Assignment 4.

When making your product generation scheme, first identify each of the closed shell species in your reaction scheme from Question 1 and number them as shown in the scheme on page 3. Note that any similar products should have the same number as the model will consider them the same, and so if a product such as carbonyl fluoride (C(O)F_2_) is produced at different spots in the mechanism it should still have the same number.

The model you will build in Assignment 4 involves a simplified environment that is composed of only air and water. In this model we will assume that all molecules can move between the air and aqueous phases and that the rate of this partitioning is determined by a *k_wet_* rate constant that will be calculated using the air-water partition coefficient K_AW_ for each compound. This movement between air and water should be incorporated into the generation scheme for every chemical, except fluoride (F^-^) and carbon dioxide (CO_2_). If a fluorine radical is formed in the gas phase (F*_(g)_), assume it immediately diffuses into water and forms an aqueous fluoride ion (F^-^_(aq)_). This transformation is so fast we assume it is essentially instantaneous and happens in the same generation as the fluorine radical was formed. We will also group both CO_2_ in the aqueous and gaseous phase together as inorganic carbon, and so the phase in which CO_2_ is produced need not be specified.

For each product in your product generation scheme (1 scheme for each chemical), be sure to:

1. Number of the chemical species (each chemical has to have a unique number in the model) as well as the phase in which the chemical exists (e.g. 1(g) and 1(aq)). (**1 mark**)
2. Use arrows to indicate reactants and products of each transformation or partitioning process. (**1 mark**)
3. Indicate relevant rate constants on top of the arrows for each reaction or process (**1 mark**)
4. Include a final (unbalanced) chemical equation that describes the fate of all the carbons and fluorines in the original compound. (**1 mark**)
5. For both of your chemicals comment on whether their atmospheric oxidation generates persistent degradation products. (**2 marks**)

**Example Product Generation Scheme:**
