# Supplementary material for: Welcome to 310 Environmental Working Group! A Group Project That Places Students in the Role of Consultants Helping Businesses Choose the Most Climate Friendly Fluorinated Gas
Source: J Chem Educ. 2024 Sep 6;101(10):4203–13. doi: 10.1021/acs.jchemed.4c00479 (PMC11465463; doi:10.1021/acs.jchemed.4c00479)
Supplement: Supplementary file 1 — ed4c00479_si_001.zip [file ed4c00479_si_001.zip › Supporting Information/Assignment 3/Generation Scheme Answer key.pdf]

Ref 1

Product Generation Scheme:

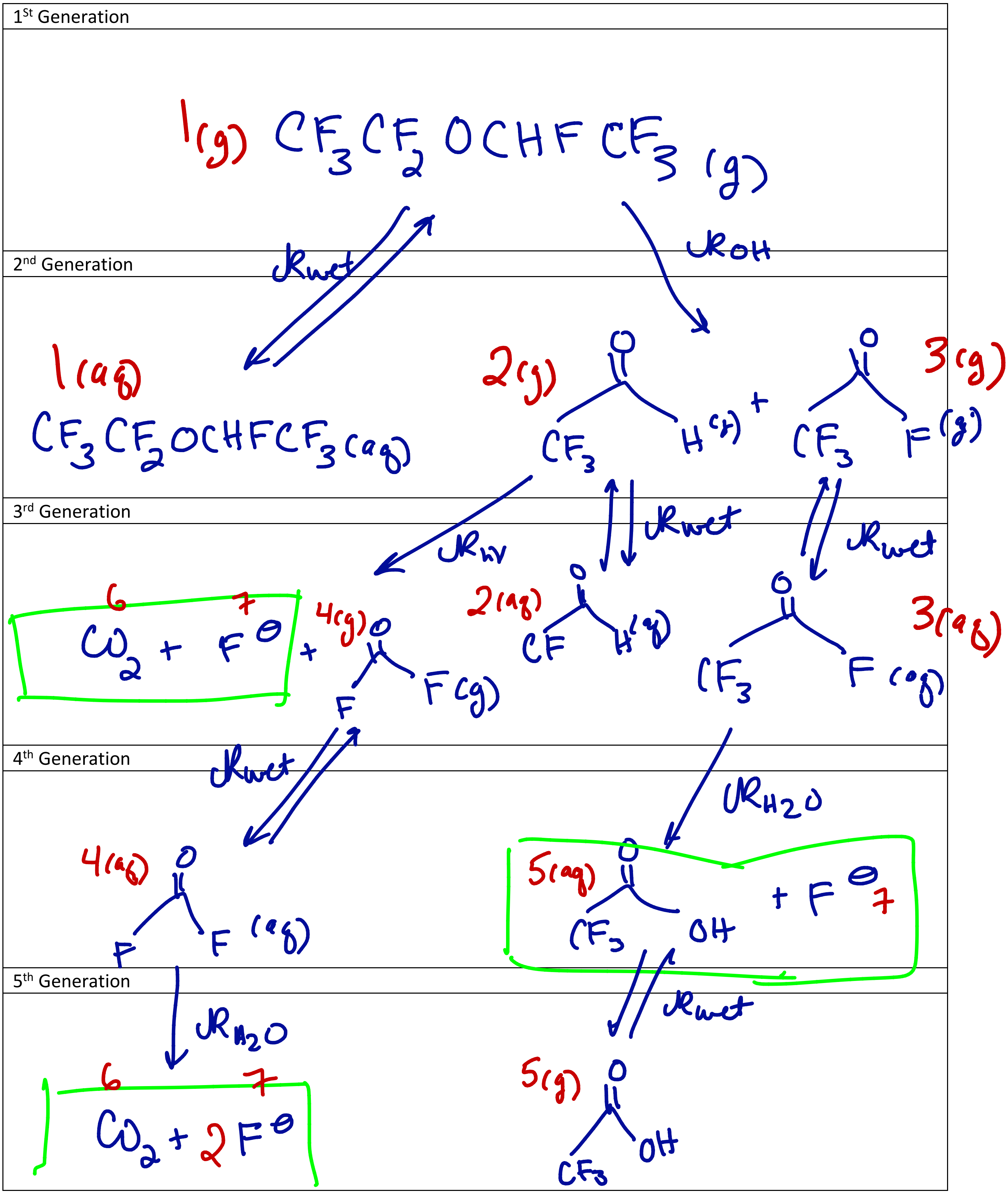

$CF_3COOH + 2CO_2 + 4F^{\ominus}$

Ref 2

Product Generation Scheme:

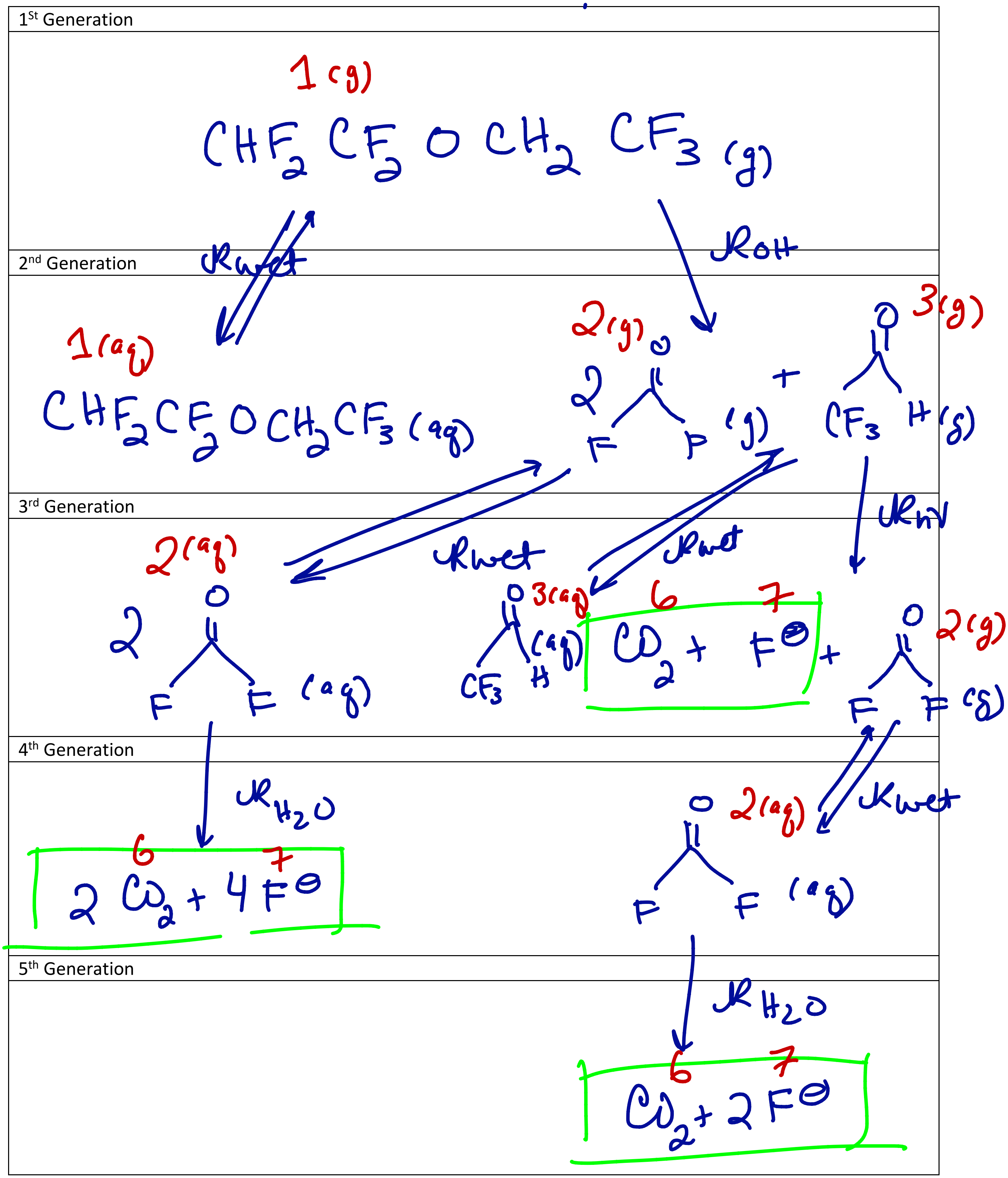

$4\ CO_2 + 7\ F^{\ominus}$

Ans 1

Product Generation Scheme:

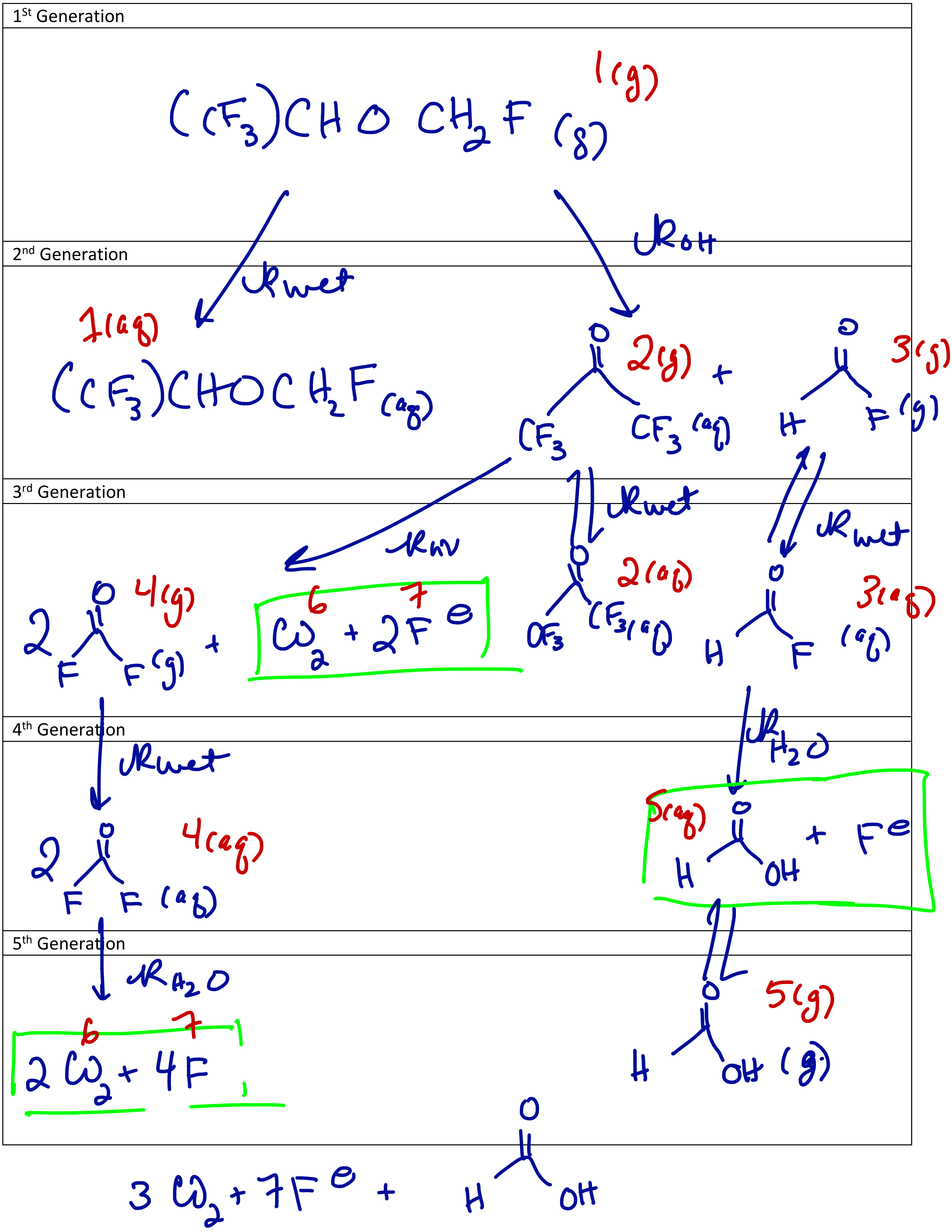

Anes 2

Product Generation Scheme:

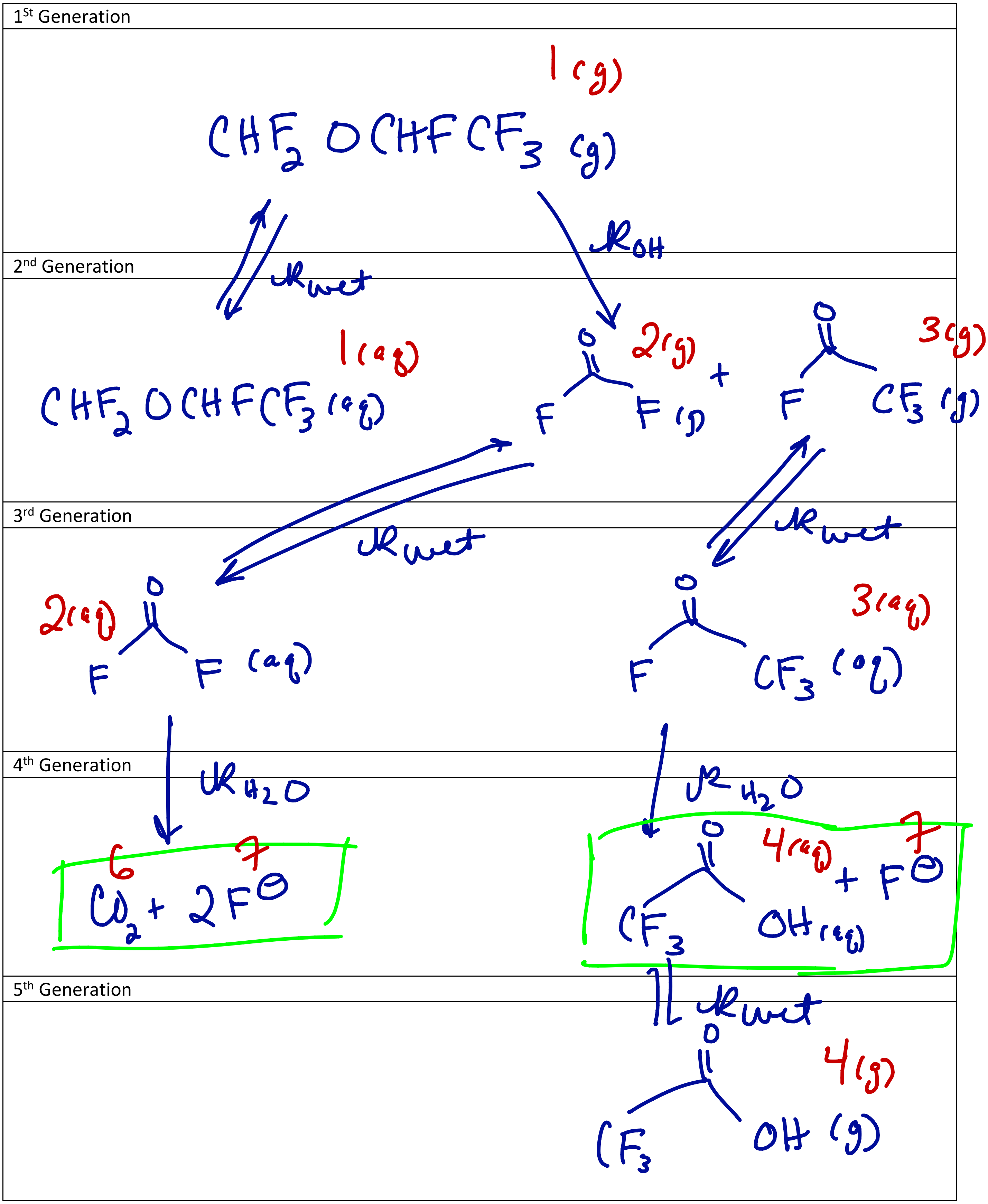

$$CF_3COOH + CO_2 + 3F^{\ominus}$$

Prop 1

Product Generation Scheme:

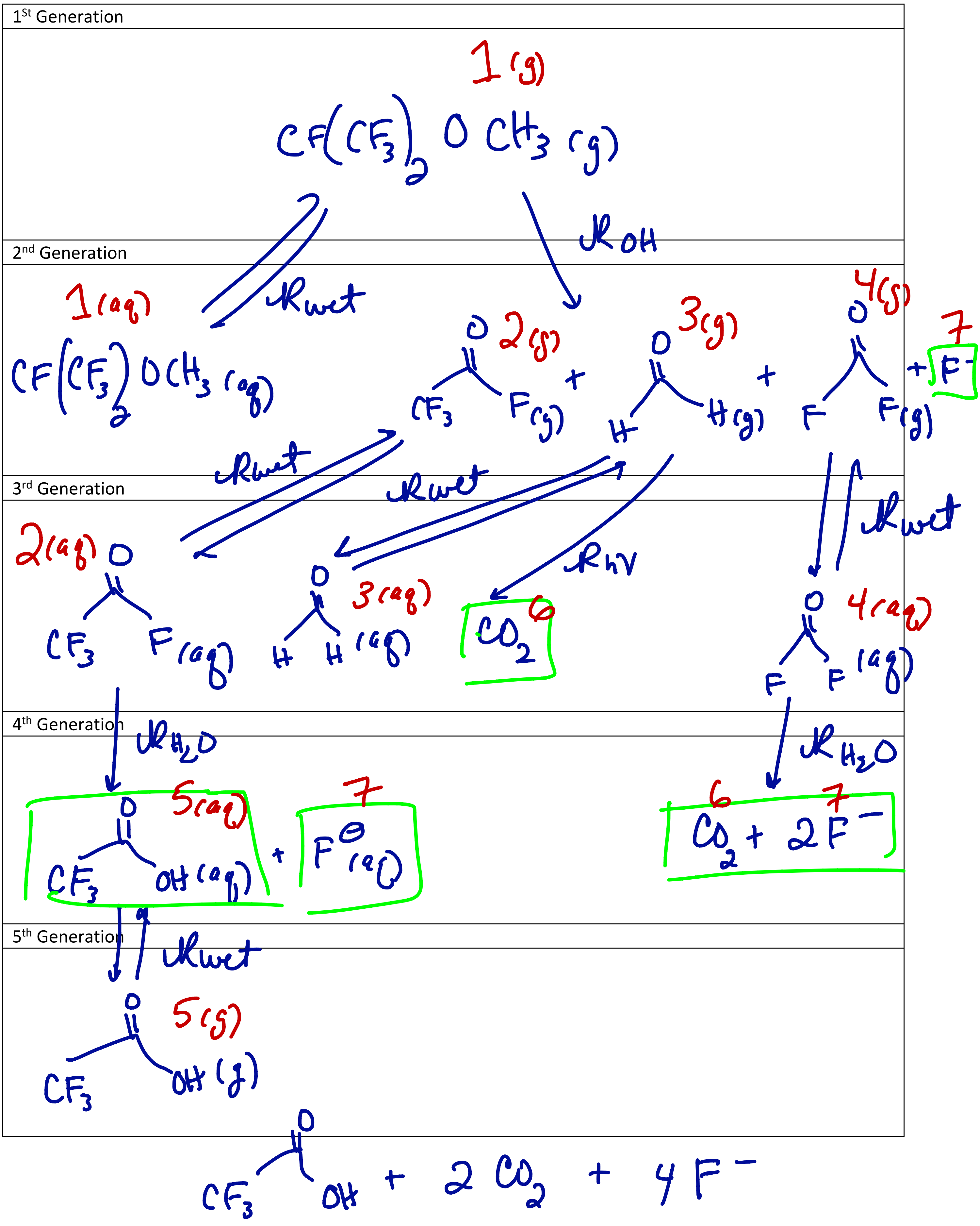

Prop 2

Product Generation Scheme:

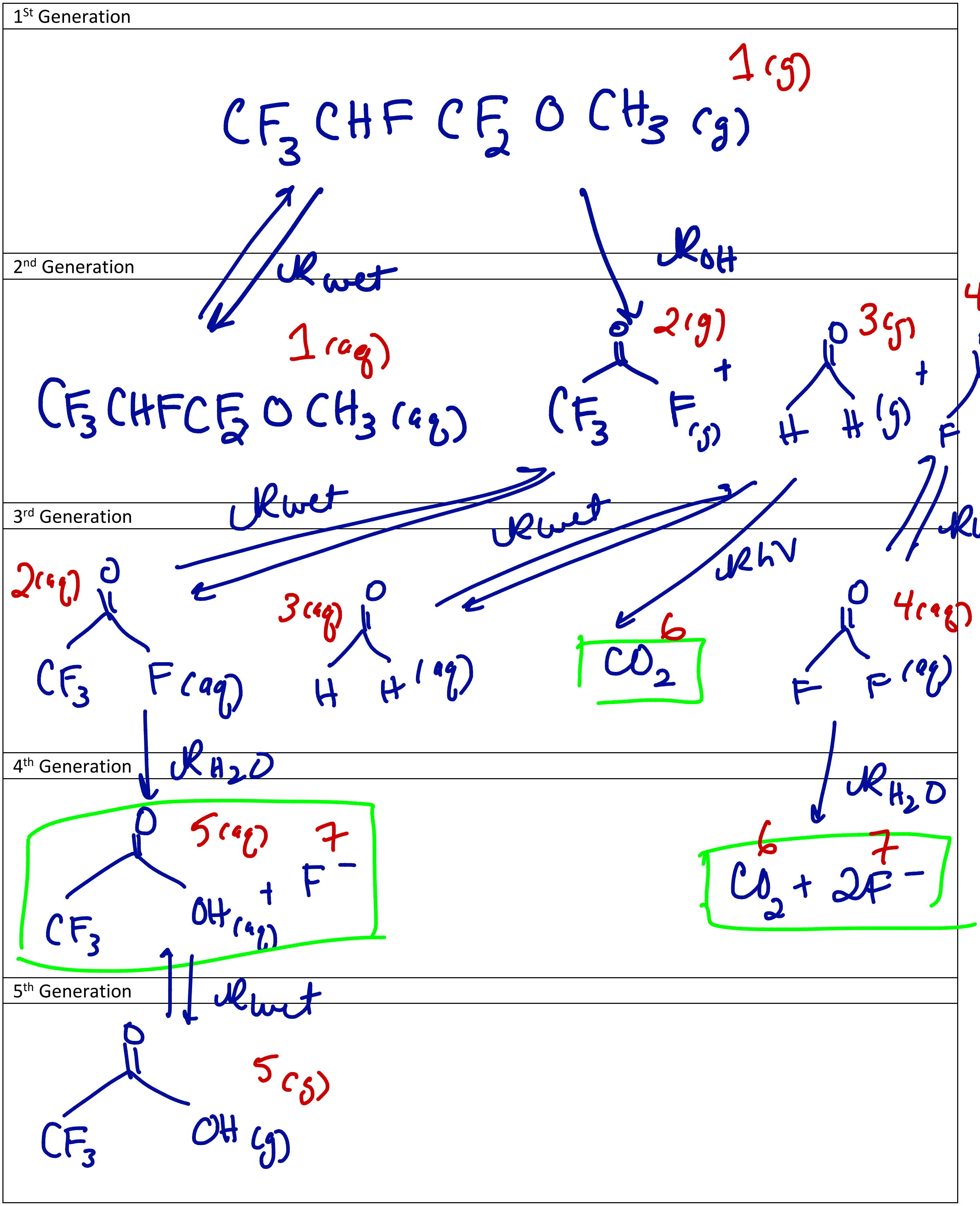

$CF_3COOH + 2CO_2 + 3F^{\ominus}$
