# Supplementary material for: Welcome to 310 Environmental Working Group! A Group Project That Places Students in the Role of Consultants Helping Businesses Choose the Most Climate Friendly Fluorinated Gas
Source: J Chem Educ. 2024 Sep 6;101(10):4203–13. doi: 10.1021/acs.jchemed.4c00479 (PMC11465463; doi:10.1021/acs.jchemed.4c00479)
Supplement: Supplementary file 1 — ed4c00479_si_001.zip [file ed4c00479_si_001.zip › Supporting Information/Assignment 3/310 EWG Assignment 3 Fall 2018 Product Generation Scheme.docx]

**Product Generation Scheme:**

| 1^St^ Generation |
| --- |
|  |
| 2^nd^ Generation |
|  |
| 3^rd^ Generation |
|  |
| 4^th^ Generation |
|  |
| 5^th^ Generation |
|  |
| Overall Reaction |
|  |
