# Supplementary material for: Welcome to 310 Environmental Working Group! A Group Project That Places Students in the Role of Consultants Helping Businesses Choose the Most Climate Friendly Fluorinated Gas
Source: J Chem Educ. 2024 Sep 6;101(10):4203–13. doi: 10.1021/acs.jchemed.4c00479 (PMC11465463; doi:10.1021/acs.jchemed.4c00479)
Supplement: Supplementary file 1 — ed4c00479_si_001.zip [file ed4c00479_si_001.zip › Supporting Information/Assignment 3/310 EWG Assignment 3 Fall 2018 Report Sheet.docx]

**Name:** **Student Number:** **Consulting Group:**

| **Questions** | **Chemical 1** | **Chemical 2** |
| --- | --- | --- |
| **Chemical Structure** |  |  |
| **Q1 (mechanism)** | Paste mechanism below | Paste mechanism below |
| **Q2 (Y/N)** |  |  |
| **Q3 (Explanation)** |  | |
| **Q4 (Generation scheme)** | Scheme Electronic or Hard Copy | Scheme Electronic or Hard Copy |
| **Q5 (Explanation)** |  |  |

**Question 1 Chemical 1 (paste ChemDraw mechanism):**

**Question 1 Chemical 2 (paste ChemDraw mechanism):**
