# Supplementary material for: Welcome to 310 Environmental Working Group! A Group Project That Places Students in the Role of Consultants Helping Businesses Choose the Most Climate Friendly Fluorinated Gas
Source: J Chem Educ. 2024 Sep 6;101(10):4203–13. doi: 10.1021/acs.jchemed.4c00479 (PMC11465463; doi:10.1021/acs.jchemed.4c00479)
Supplement: Supplementary file 1 — ed4c00479_si_001.zip [file ed4c00479_si_001.zip › Supporting Information/Assignment 3/ChemDraw Figure Answer Key.pdf]

Refrigerant 1

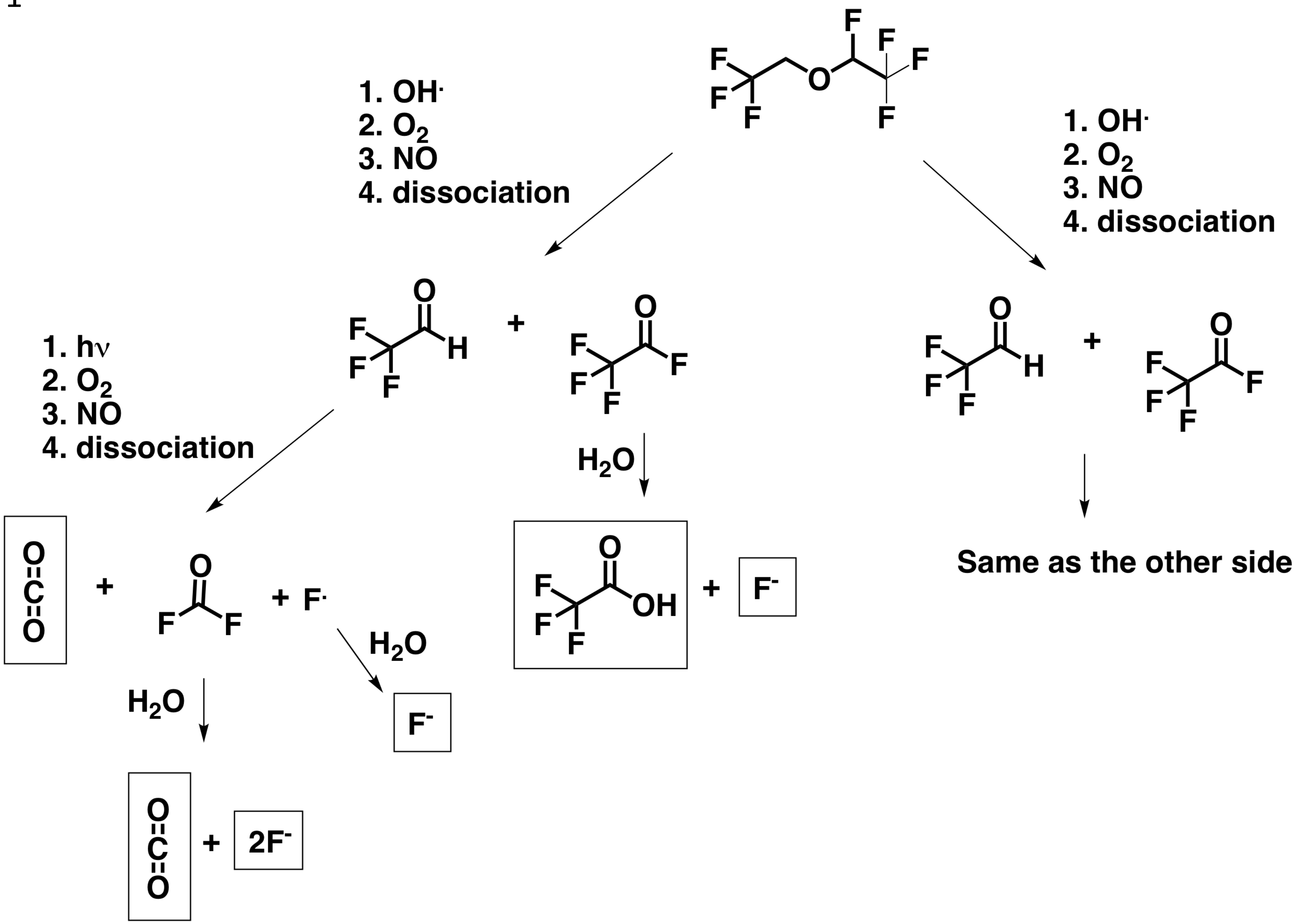

Refrigerant 2

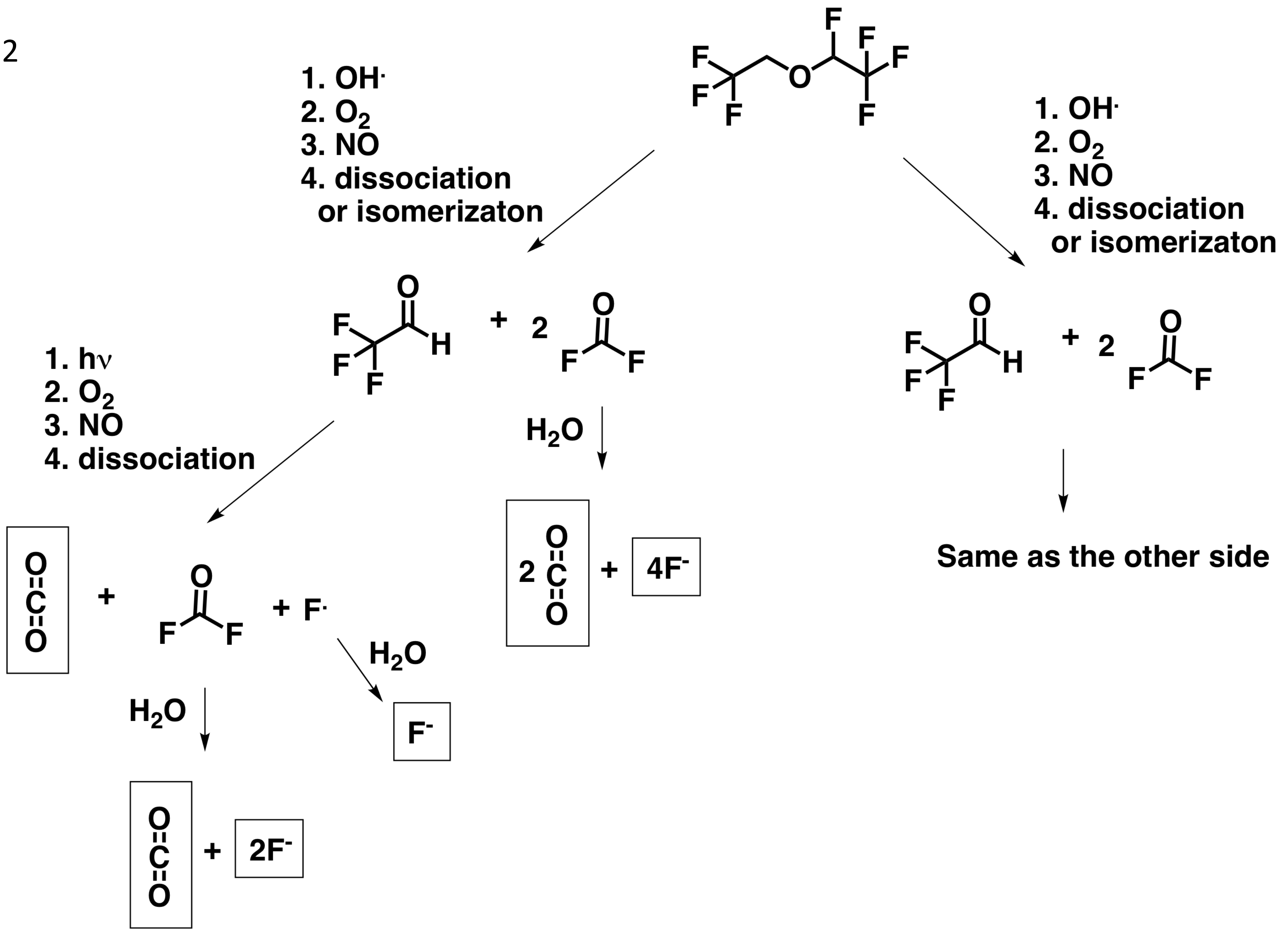

Anesthetic 1

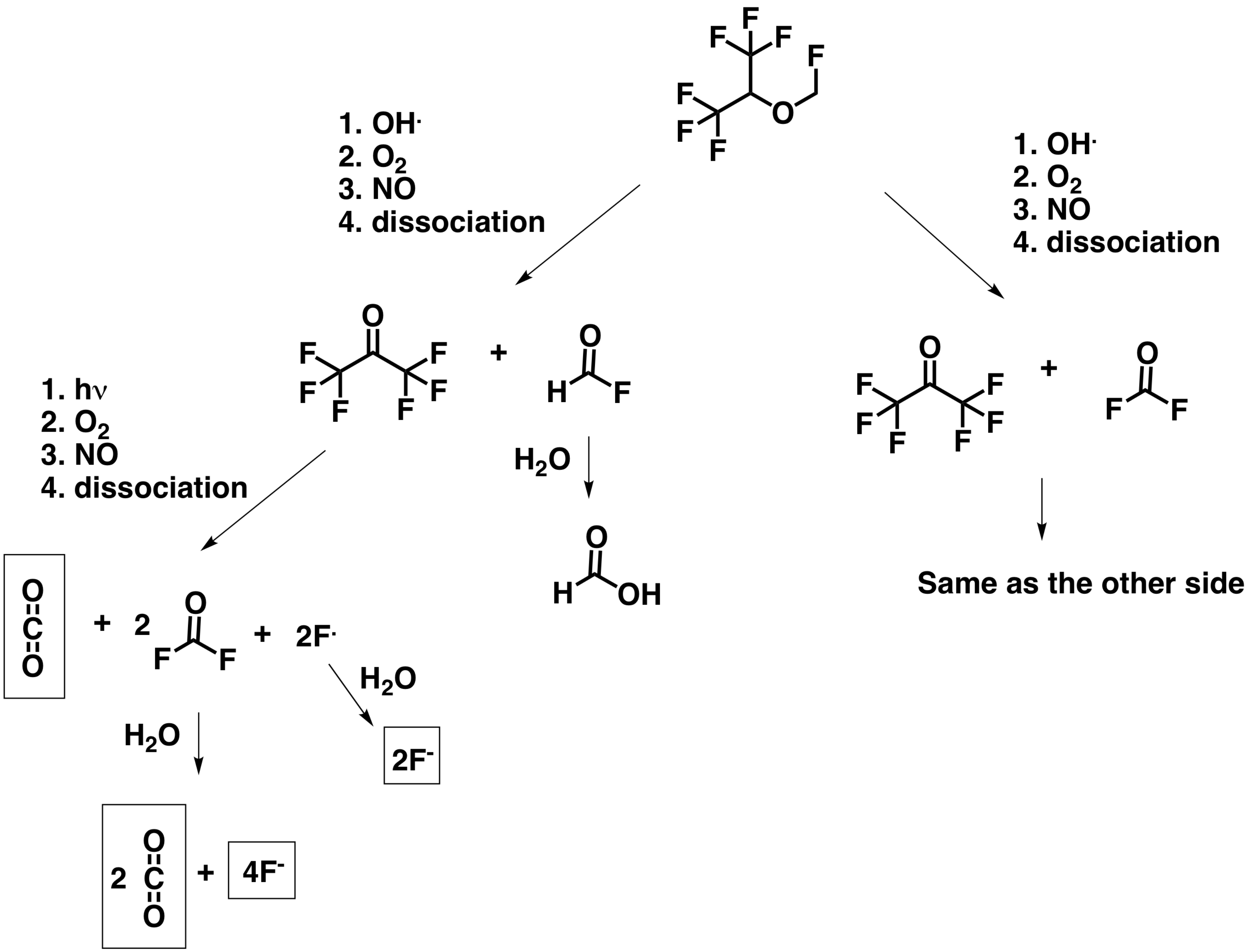

Anesthetic 2

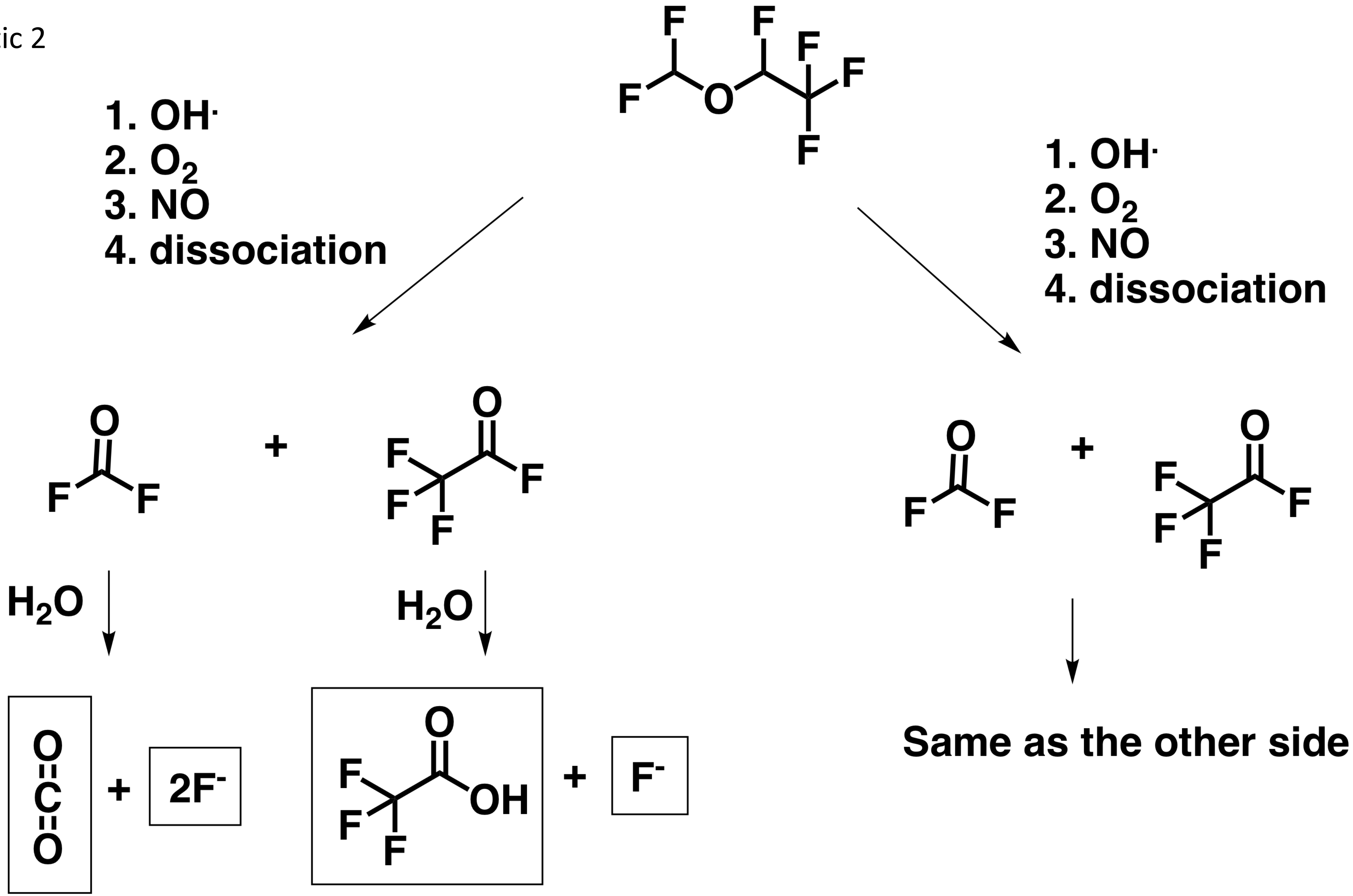

Propellant 1

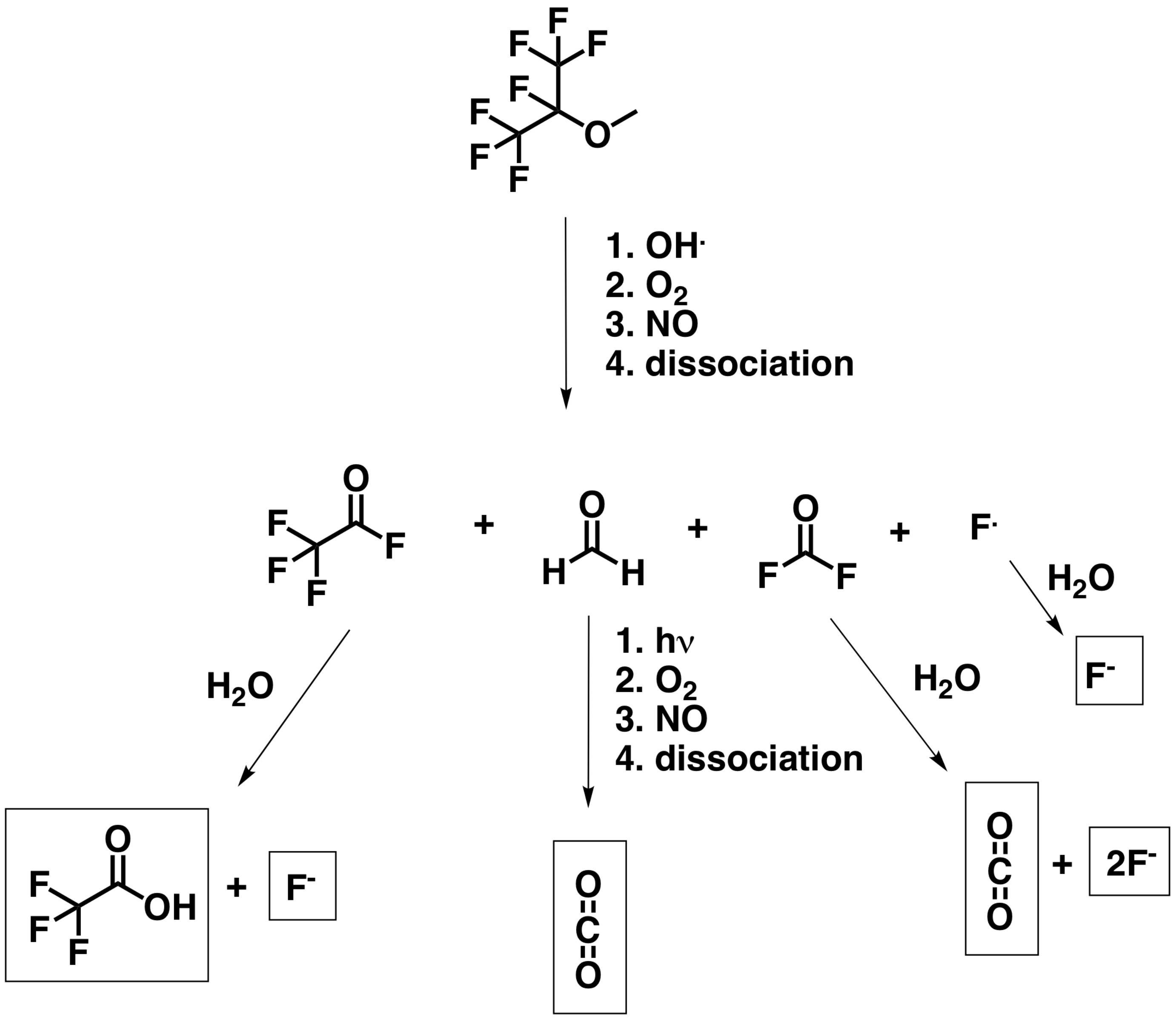

Propellant 2

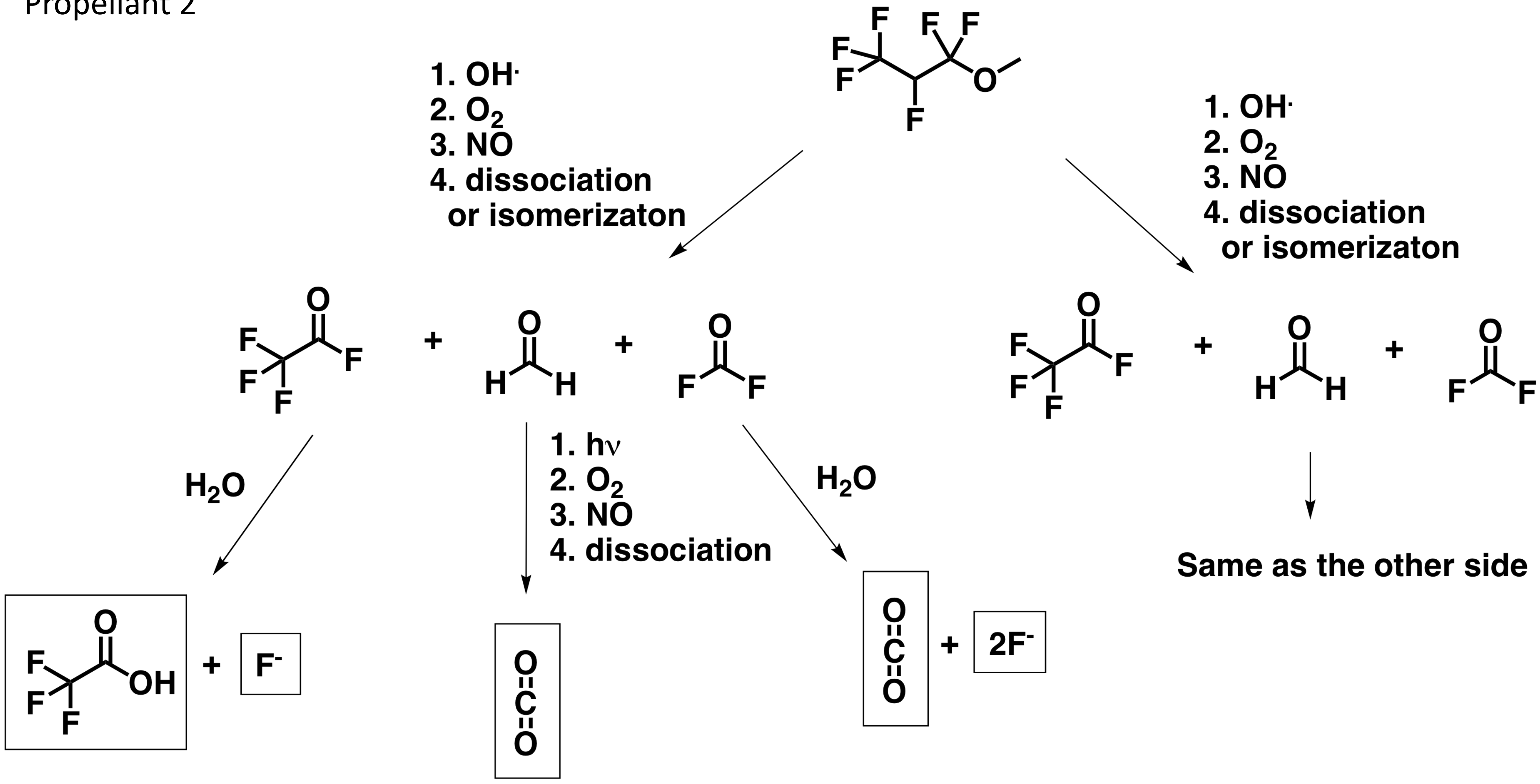

Note: for this compound there is the possibility of making formic acid instead of formaldehyde in this mechanism for an isomerization mechanism from the terminal methoxy.
