# Supplementary material for: Welcome to 310 Environmental Working Group! A Group Project That Places Students in the Role of Consultants Helping Businesses Choose the Most Climate Friendly Fluorinated Gas
Source: J Chem Educ. 2024 Sep 6;101(10):4203–13. doi: 10.1021/acs.jchemed.4c00479 (PMC11465463; doi:10.1021/acs.jchemed.4c00479)
Supplement: Supplementary file 1 — ed4c00479_si_001.zip [file ed4c00479_si_001.zip › Supporting Information/Assignment 2/310 EWG Assignment 2 Fall 2018 Report Sheet.docx]

**Name:** **Student Number:** **Consulting Group:**

| **Questions** | **Chemical 1** | **Chemical 2** | |
| --- | --- | --- | --- |
| **Chemical Structure** |  |  | |
| **Q1** | Submit spectra as a hardcopy in class | | |
| **Q2** | Completed Question 2 Table – Chemical 1 | Completed Question 2 Table – Chemical 1 | |
| **Q3(a) (frequency)** |  |  | |
| **Q3(b) (Y/N)** |  |  | |
| **Q3(c) (Description of vibration)** |  |  | |
| **Q3(d) (Explanation)** |  |  | |
| **Q4 (Explanation)** |  |  | |
| **Q5(a) (years)** |  |  | |
| **Q5(b) (seconds^-1^)** |  |  | |
| **Q5(c) (ppb)** |  |  | |
| **Q5(d)** | Submit plots as a hardcopy in class | | |
| **Q6 (W m^-2^)** |  | |  |
| **Q7** | Submit plots as a hardcopy in class | | |
| **Q8 (ppb)** |  | | |
| **Q9 (W m^-2^)** |  | | |
| **Q10 (GWP)** |  | |  |
| **Q11 (explanation)** |  | | |

**Question 2 Table – Chemical 1**

| **Frequency of the vibration (cm^-1^)** | **Intensity**  **(Km mol^-1^)** | **Absorbance Cross Sectional area**  **(cm molecule^-1^)** | **Radiative Efficiency per Unit Cross Sectional Area**  **(W m^-2^ ppbv^-1^ (cm molecule^-1^)^-1^)** | **Radiative Efficiency**  **(W m^-2^ ppbv^-1^)** |
| --- | --- | --- | --- | --- |
|  |  |  |  |  |
|  |  |  |  |  |
|  |  |  |  |  |
|  |  |  |  |  |
|  |  |  |  |  |
|  |  |  |  |  |
|  |  |  |  |  |
|  |  |  |  |  |
|  |  |  |  |  |
|  |  |  |  |  |
|  |  |  |  |  |
|  |  |  |  |  |
| **Total Radiative Efficiency*** | | | |  |

**Question 2 Table – Chemical 2**

| **Frequency of the vibration (cm^-1^)** | **Intensity**  **(Km mol^-1^)** | **Absorbance Cross Sectional area**  **(cm molecule^-1^)** | **Radiative Efficiency per Unit Cross Sectional Area**  **(W m^-2^ ppbv^-1^ (cm molecule^-1^)^-1^)** | **Radiative Efficiency**  **(W m^-2^ ppbv^-1^)** |
| --- | --- | --- | --- | --- |
|  |  |  |  |  |
|  |  |  |  |  |
|  |  |  |  |  |
|  |  |  |  |  |
|  |  |  |  |  |
|  |  |  |  |  |
|  |  |  |  |  |
|  |  |  |  |  |
|  |  |  |  |  |
|  |  |  |  |  |
|  |  |  |  |  |
|  |  |  |  |  |
| **Total Radiative Efficiency*** | | | |  |
