# Supplementary material for: Welcome to 310 Environmental Working Group! A Group Project That Places Students in the Role of Consultants Helping Businesses Choose the Most Climate Friendly Fluorinated Gas
Source: J Chem Educ. 2024 Sep 6;101(10):4203–13. doi: 10.1021/acs.jchemed.4c00479 (PMC11465463; doi:10.1021/acs.jchemed.4c00479)
Supplement: Supplementary file 1 — ed4c00479_si_001.zip [file ed4c00479_si_001.zip › Supporting Information/Assignment 2/310 EWG Assignment 2 Fall 2018.docx]

| 310 Environmental Working Group |  |
| --- | --- |

Assignment 2

Deliverables

**Calculating Radiative Efficiency**

The purpose of this exercise is to use the Gaussian 09 suite of software to calculate infrared spectra for your chemicals and then use that to estimate radiative efficiency (RE), which is the efficiency with which a chemical warm’s the atmosphere by absorbing radiation emitted by the surface of the Earth on its escape to space. This is an exercise that involves extrapolating values and making assumptions, making it semi-quantitative but still very useful. Radiative efficiency (RE) has units of W m^-2^ ppbv^-1^. The paper by Pinnock *et al.* (*J. Geophys. Res.* 1995, 100 (D11), 23,227-23,238) attached to this assignment describes a method to calculate radiative efficiency using IR spectra. The calculation will be explained below using perfluoroethane (CF_3_CF_3_) as an example.

The IR spectra for perfluoroethane is shown above. This spectrum contains two peaks at 1225 cm^-1^ and 1100 cm^-1^ with transmittance values of 0.10 and 0.45 (I divided % transmittance by 100 to get transmittance as a fraction). If you know the concentration (*c*) of perfluoroethane and the path length (*l*) used in the experiment you can calculate the absorption cross section (ν) for each peak in the spectrum.

$$\ln\left( T \right)=-\sigma cl$$

$$\sigma=-\frac{\ln\left( T \right)}{cl}$$

To calculate radiative efficiency, we need an area under the absorption cross section curve not just a maximum cross section value. For this example calculation, we will assume both peaks are 10 cm^-1^ wide and so we multiply the absorbance cross section (cm^2^ molecule^-1^) by 10 cm^-1^ to obtain an absorption cross sectional area (cm^2^ molecule^-1^ cm^-1^).

**Table 1.**  Frequency, transmittance, and example absorption cross section calculation for perfluorinated ethane (CF3CF3) assuming a 1 cm path length and a concentration of 1.5 x 10^17^ molecules cm^-3^.

| **Frequency**  **ν (cm^-1^)** | **Transmittance**  **(T)** | **Absorption cross section**  **σ (cm^2^ molecule^-1^)** | **Absorption Cross Sectional Area**  **(cm^2^ molecule^-1^ cm^-1^)** |
| --- | --- | --- | --- |
| 1100 | 0.45 | 5.32 x 10^-18^ | 5.32 x 10^-17^ |
| 1225 | 0.10 | 1.54 x 10^-17^ | 1.54 x 10^-16^ |

This absorption cross sectional area can then be multiplied by the radiative efficiency (RE) per unit cross sectional area provided in the excel spreadsheet that accompanies this assignment (310EWG_Assign_2_RE_values.xlsx) and is shown graphically in Figure 2. These RE values were obtained from the Pinnock *et al*. paper and are provided at 5 cm^-1^ intervals (simply use the value that are closest to your frequency values). To obtain the total radiative efficiency for your compound simply add the calculated radiative efficiencies for each absorption frequency (shown in Table 2).


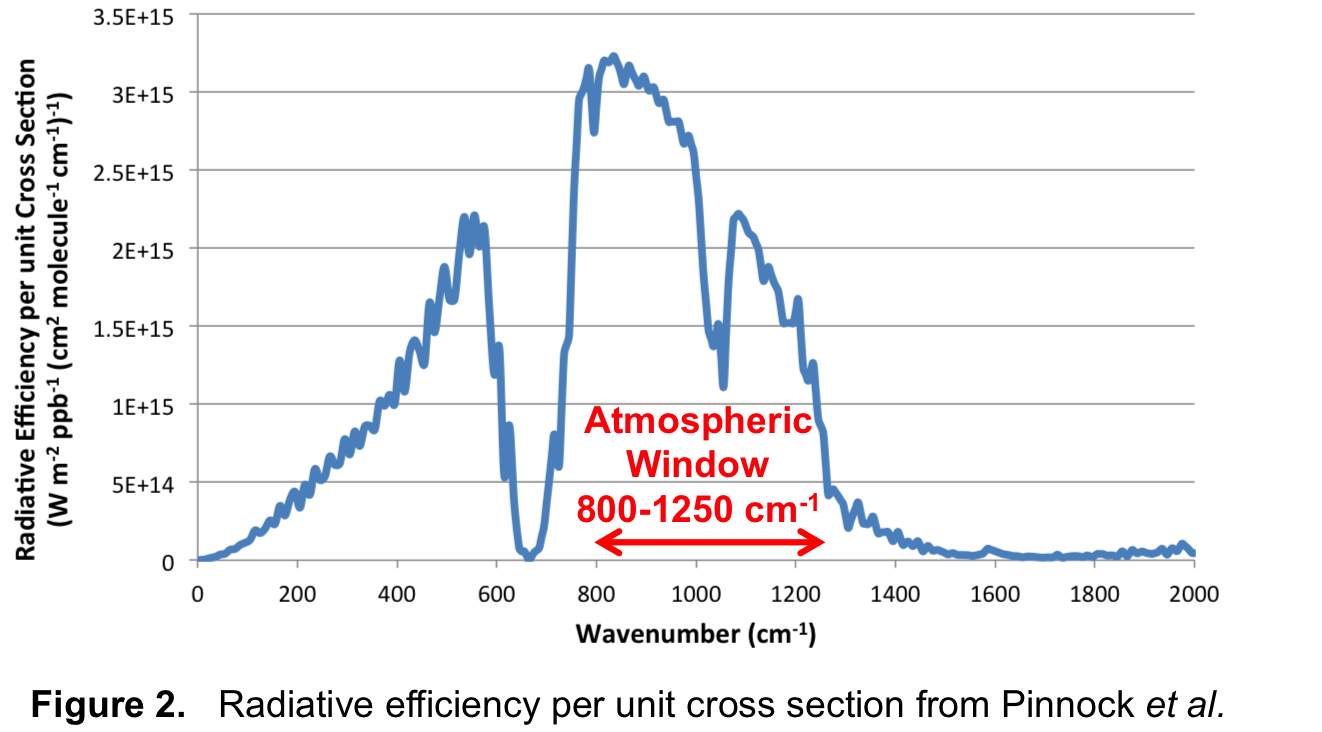


**Table 2.** Frequency, absorption cross sections, absorption cross sectional area, radiative efficiency per unit cross sectional area (from accompanying excel spreadsheet ICP_Assign_3_RE_values.xlsx), and radiative efficiency for perfluorinated ethane (CF3CF3).

| **Frequency ν (cm^-1^)** | **Absorption Cross Section, σ**  **(cm^2^ molecule^-1^)** | **Absorption Cross Sectional Area**  **(cm^2^ molecule^-1^ cm^-1^)** | **Radiative Efficiency per Unit Cross Sectional Area**  **(W m^-2^ ppbv^-1^ (cm^2^ molecule^-1^ cm^-1^)^-1^)** | **Radiative Efficiency**  **(W m^-2^ ppbv^-1^)** |
| --- | --- | --- | --- | --- |
| 1100 | 5.32 x 10^-18^ | 5.32 x 10^-17^ | 2.14 x 10^15^ | 0.114 |
| 1225 | 1.54 x 10^-17^ | 1.54 x 10^-16^ | 1.15 x 10^15^ | 0.176 |
|  |  |  | **Total Radiative Efficiency** | 0.29 |

Instructions for performing the Gaussian 09 calculations are provided in another document. In these calculations you will generate IR spectra with a defined peak width of 10 cm^-1^ you can then use to calculate radiative efficiency. Using the instructions provided you will generate an IR spectrum for both of your chemicals with a defined peak width of 10 cm^-1^. You will use the spectra to determine the peak absorbance for each vibrational frequency (don’t use the list of frequencies alone as the fine structure provided is too detailed). Unlike the IR spectrum shown in Figure 1, the y-axis of the calculated spectra is in units of intensity as opposed to % transmittance. These intensity values are actually absorption cross sectional area values for peaks with a width of 10 cm^-1^ (as defined in the instructions) and has units of Km mol^-1^. Use relevant conversion factors and Avogadro’s number to convert this intensity to an absorption cross sectional area in units of cm molecule^-1^ (this is a simplified version of the units of cm^2^ molecule^-1^ cm^-1^ used for absorption cross sectional area in the example above). You can directly apply the radiative efficiency per unit cross sectional area from the excel spreadsheet (310EWG_Assign_2_RE_values.xlsx) to this absorption cross sectional area with units of cm molecule^-1^ (no need to multiply by 10 cm^-1^ peak width).

**Calculating Global Warming Potential**

Global warming potential (GWP) is a thought experiment where 1 Kg of a compound is added to the atmosphere and its effect on the energy balance (also called radiative balance) of the Earth is calculated over a given time horizon, typically 100 years, relative to the same scenario for carbon dioxide.

$$GWP=\frac{\int_{t=0}^{t=100} {RF}_{compound}}{\int_{t=0}^{t=100} {RF}_{{CO}_{2}}}$$

This change in radiative balance is characterized by a compound’s radiative forcing (RF in W m^-2^), which is calculated by multiplying radiative efficiency (RE in W m^-2^ ppb^-1^) by atmospheric concentration (in ppb). We will determine a compound’s total forcing (RF) over 100 years by multiplying its radiative efficiency by its atmospheric concentration in one-year increments over 100 years, and then taking the sum of these forcing values.

**Questions**

This assignment includes both an online submission and an in-class hard copy. All solutions will be submitted online through Quercus by completing the word document entitled “310 EWG Assignment 2 Fall 2018 Report Sheet.docx”. Your submitted report sheet should be renamed as “Surname_FirstName_310EWG_Assign2.docx”. In addition to this report sheet you must submit a hard copy of the IR spectra (Question 1) and decay plots (Question 5(d)) for both of your chemicals, as well as the decay plots for CO_2_ (Question 7). In addition to the spectra and plots you may also include relevant calculations in your hard copy to receive any relevant partial credit. Be sure to include your name and student number on the in-class submission.

1. For each compound submit a copy of the IR spectra generated by the Gaussian 09 calculation. (May be a screenshot) (**1 mark**)
2. Complete the “Question 2 Table” for both Chemicals 1 and 2 by listing all vibrational frequencies with intensities greater than 25 in the generated IR spectra for each compound. Then complete the remaining columns of the table and calculate the overall radiative efficiency for both compounds. (**2 marks**)
3. Of the vibrational frequencies identified in Question 2 identify the vibration that was the largest contributor to the overall radiative efficiency ***for each chemical***.
   1. What is the frequency of this vibration?
   2. Is this frequency in the “atmospheric window”? (**0.5 marks**)
   3. Describe the vibrational mode by watching how the molecule moves in Gaussian 09 and assign it to one or combination of the following types of vibrations: Stretching (symmetrical or asymmetrical), Bending, Rocking, Scissoring, Twisting, Wagging. (**0.5 marks**)
   4. Is the importance of this vibration to the radiative efficiency of the chemical driven by its intensity or its frequency relative to the emission spectrum of the Earth? Explain your answer (**2 marks**)
4. All of the chemicals will have low intensity absorption frequencies between 3000-3500 cm^-1^. These frequencies result from the movement of the electron more than one vibrational energy level (e.g. ν = 0 🡪 ν = 2) and are called overtones. If the overtone bands were equal in intensity to the strongest absorption frequencies of the molecule would they be important contributors to the overall radiative efficiency of the chemicals? Explain your answer. (**2 marks**)
5. The concentration of a chemical that is lost via a first order reaction (or pseudo first order) or process can be calculated using the following relationship for exponential decay:

$\left[ compound \right]_{t}={[compound]}_{o}e^{-k_{total}t}$.

- 1. Provide the overall atmospheric lifetime for each of your chemicals that was calculated in Assignment 1 Question 6. (Note: If there was a mistake in your initial calculations, please include the revised value here)
  2. Using their overall atmospheric lifetime, calculate an overall rate constant (*k_total_*) for the rate of loss for each of your chemicals, and convert the units to seconds^-1^. (**1 mark**)
  3. Provide the concentration in ppb that was calculated in Assignment 1 for each of your chemicals after the 1 Kg bolus addition to the atmosphere (Question 10(b)). (Note: If there was a mistake in your initial calculations, please include the revised value here)
  4. Use the concentration (in ppb) and overall rate constant (*k_total_*) for each chemical to plot the loss of that chemical over 100 years after the 1 Kg bolus addition to the atmosphere. Attach these plots. (Note: your concentration units (in ppb) should be in scientific notation, the style and number of significant figures used in the worksheet will be that used on the graph axes). (**2 marks**)

1. For both of your chemicals use the loss curve after a 1 Kg bolus addition to the atmosphere from Question 5(d), and the radiative efficiency from Question 2 to calculate the total radiative forcing (RF in W m^-2^) over 100 years. Perform the calculation from 0-100 years in 1-year increments. (**2 marks**)
2. The IPCC reports the lifetime of carbon dioxide as a range of values (5-200 years). Consistent with IPCC recommendations (<https://www.ipcc.ch/publications_and_data/ar4/wg1/en/ch2s2-10-2.html>) in this calculation we are modeling the concentration of carbon dioxide over time as the sum of four different loss mechanisms:

$${[{CO}_{2}]}_{t}={[{CO}_{2}]}_{o}\left( 0.186 e^{\frac{- t}{1.186 years}}+0.338 e^{\frac{- t}{18.51 years}}+0.259 e^{\frac{- t}{172.9 years}}+0.217 \right)$$

Plot the fractional loss of CO_2_ over 100 years (starting at 0 in 1-year increments) for each of the four loss mechanisms described above, as well as the total fractional loss of carbon dioxide. Include all 5 plots on the same graph. (**2 marks**)

1. Assuming there are 1.7 x 10^20^ moles of gas in the atmosphere, what is the change in the concentration of CO_2_ (in units of ppb) in the atmosphere after a 1 Kg bolus addition? (Note: In this calculation we are only considering the fate of the added CO_2_ and not the current levels, and so do not include the current concentration of atmospheric CO_2_ in this calculation) (**1 mark**)
2. CO_2_ has a radiative efficiency of 1.4 x 10^-5^ W m^-2^ ppb^-1^. Use this value together with the concentration from Question 8 and the overall loss kinetics from Question 7 to calculate the total radiative forcing (RF in W m^-2^) of carbon dioxide over 100 years. Perform the calculation from 0-100 years in 1-year increments. (**1 mark**)
3. Use the total radiative forcing over 100 years for a 1 Kg bolus addition to the atmosphere (RF in W m^-2^) for CO_2_ from Question 9 and for both compounds from Question 6 to calculate a global warming potential for all your chemicals. (**1 mark**)
4. How similar are the GWPs calculated for your two chemicals? Are the observed differences driven by differences in radiative efficiency or kinetics? (**2 marks**)
