# Supplementary material for: Welcome to 310 Environmental Working Group! A Group Project That Places Students in the Role of Consultants Helping Businesses Choose the Most Climate Friendly Fluorinated Gas
Source: J Chem Educ. 2024 Sep 6;101(10):4203–13. doi: 10.1021/acs.jchemed.4c00479 (PMC11465463; doi:10.1021/acs.jchemed.4c00479)
Supplement: Supplementary file 2 — ed4c00479_si_002.pdf [file ed4c00479_si_002.pdf]

Supporting Information

Welcome to 310 Environmental Working Group! A Group Project That Places Students in the Role of Consultants Helping Businesses Choose the Most Climate Friendly Fluorinated Gas

Jessica C. D'eon[†]*, Sivani Baskaran[†Π], Jennifer A. Faust[¶], Mima Staikova[†], and Cora J. Young[§]

[†]University of Toronto, Department of Chemistry, 80 St. George Street, Toronto, ON, Canada M5S 3H6

[Π]Department of Environmental Engineering, Norwegian Geotechnical Institute (NGI), P.O. Box 3930, Ullevål Stadion, NO-0806 Oslo, Norway

[¶]College of Wooster, Department of Chemistry, 943 College Mall, Wooster, OH, 44691, USA

[§]York University, Department of Chemistry, 4700 Keele Street, Toronto, ON, Canada M3J 1P3

*Email: Jessica.deon@utoronto.ca

This document includes:

Additional Considerations about the Quantum Mechanical Calculations 1

Results of the chemical fate model 1

Notes on the different iterations of the project 2

Notes on modular ways of using the content 3

CHM310 Winter 2017 anonymous survey 4

CHM310 Winter 2018 anonymous survey 6

Additional Considerations about the Quantum Mechanical Calculations

Students in this class, and in chemistry courses in general, have experience acquiring and interpreting IR spectra using experimental methods. Calculating the IR spectrum in the projects was an additional learning experience showcasing the ability of quantum mechanical calculations to predict experimental properties. It also allowed students to visualize the vibrational modes of each IR band, deepening their appreciation of what is physically happening in the molecule. To support this learning, a brief presentation of the quantum mechanical principles used to calculate vibrational frequencies, and the advantages and limitations of the calculations, was delivered in class. Details of the calculations, including instructions to students and the presentation, are provided with the supporting information.

Results of the chemical fate model

Figure S1 shows the model output from the Excel VBA program from a student who was assigned the two inhaled anesthetics Sevoflurane (Figure S1(a)) and Desflurane (Figure S1(b)). The model outputs the amount of each chemical in moles, as concentration is complicated by the different volumes of the air and water compartments. The parent chemicals are visualized in red. This group calculated a lifetime of 0.78 years for Sevoflurane and 4.1 years for Desflurane, and this difference can be seen in the plots with the concentration of Sevoflurane not visible above the baseline in Figure S1(a) but a low steady-state concentration of Desflurane visible for the first 50 years in Figure S1(b). Both plots show the production of F^- (blue hashed line) and CO_2 (green solid line) as final products, only Desflurane (Figure S1(b)) also shows the production of TFA (purple solid and dashed lines). Figure S1(b) shows most of the TFA in the gas phase with some in the aqueous phase and no movement between these in the 50 years after emission stopped. This is not correct, and it seems the student likely didn't program the movement of TFA into the aqueous phase correctly, however the issue of the implication of the production of a persistent product like TFA to long term environmental contamination is still clear as TFA remains in the model even after emission of the parent chemical stopped at the 50-year mark.

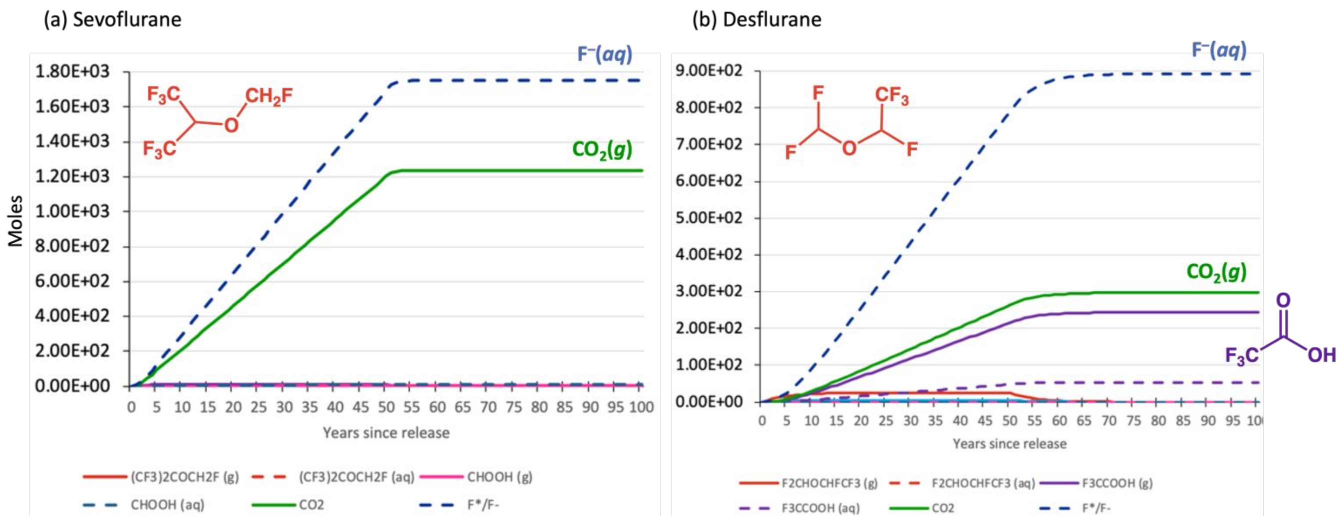

Figure S1. Student generated output from the Excel VBA chemical fate model for the two inhaled anesthetics (a) Sevoflurane and (b) Desflurane. These results show the production of final products over 100 years after 1 kg per year emission for the first 50 years. Chemicals in the gas phase are indicated by solid lines in the plot and those in the aqueous phase by dashed lines.

Notes on the different iterations of the project

The implementation of the project has changed over the years. In Winter 2016 students were given the role of scientific advisors to Environment Canada and tasked with comparing the environmental effects of two fluorinated gases. This first iteration did not include a group learning component or a presentation, these were added in the next iteration in Winter 2017 when the framing of the project was also moved to an industrial setting. These changes were made to diversify the assessment modalities and to introduce a peer-learning component to help students correct their answers between assignments. Before the fall 2018 iteration each student or each group was given a unique combination of two fluorinated gases. This was done to explore possible combinations and to decrease issues related to academic integrity. The long list of fluorinated gases used in these iterations can be found in the supporting information file titled “Candidate Compounds and Details.xlsx”. However, we found that providing students with an interesting comparison between chemicals mattered more than giving every student or group a different set to compare and so this is why we move to the three scenarios outlined in the paper in Fall 2018 and believe this setup worked best.

Notes on modular ways of using the content

The project as presented here was designed specifically for the content of CHM310 at the University of Toronto as taught between 2016 and 2018 and so it may be difficult to implement comprehensively in other settings. With this in mind, we suggest taking the content and modifying it to be done individually. This can be done with any of the assignments. As an example a discussion of the relationship between IR spectroscopy and the potency of a greenhouse gas is included in a “chemistry connections” slide presented in class to our first-year organic chemistry course (CHM136 Organic Chemistry I), see Figure S2.

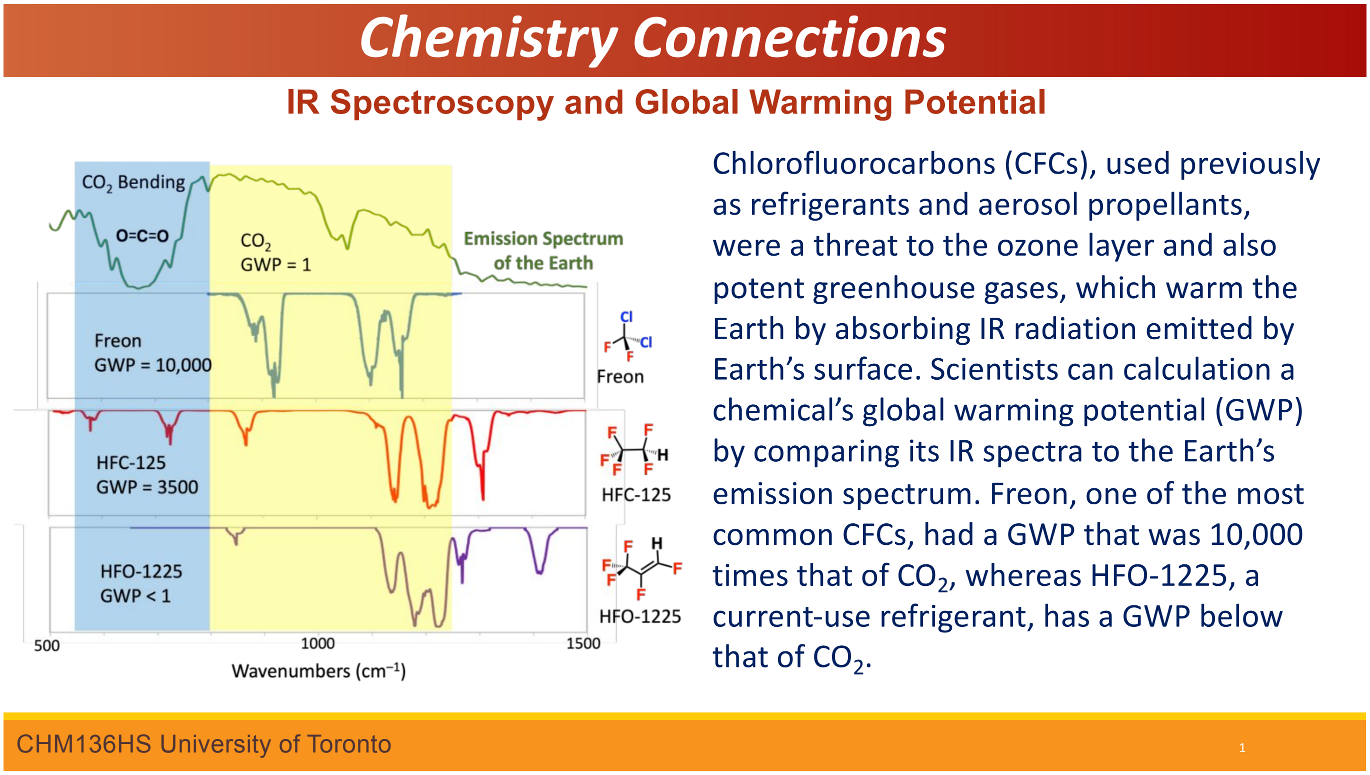

Figure S2. Slide used in our first-year organic chemistry class that describes the relationship between IR spectroscopy and the potency of greenhouse gases.

CHM310 Environmental Chemistry – Winter 2017 Student Survey
Industrial Consultants Project (ICP) Questionnaire

The purpose of this questionnaire is to obtain additional feedback from that collected by the course reviews through the Faculty of Arts and Science. Completion of this questionnaire is voluntary, anonymous and confidential

Note that student responses are shown below each question in blue.

1. The ICP assignments enhanced my learning in the course.

	Strongly agree	Agree	Neutral	Disagree	Strongly disagree
n = 69	20	45	1	1	2

2. The ICP assignments were an appropriate amount of work.

	Strongly agree	Agree	Neutral	Disagree	Strongly disagree
n = 68	9	40	14	3	2

3. I would have preferred unrelated problem sets instead of the ICP assignments.

	Strongly agree	Agree	Neutral	Disagree	Strongly disagree
n = 69	1	7	20	31	10

4. I feel as though the ICP assignments created a sense of continuity and purpose in CHM310.

	Strongly agree	Agree	Neutral	Disagree	Strongly disagree
n = 68	22	39	5	1	1

5. I feel as though meeting other students through the group work component of the ICP assignments helped my learning in CHM310 as a whole.

	Strongly agree	Agree	Neutral	Disagree	Strongly disagree
n = 68	25	26	11	3	3

6. How many of your group members did you know before taking this course?

	None	1	2	3	4
n = 69	40	16	7	6	0

7. What was the primary way your group communicated to come to a consensus value? (select all that apply)

In person	By email	By text or other messaging service	Facebook or other social media	Learning management system
28	8	8	43	6

8. Did all group members participate in choosing the consensus values?

	Yes	No
n = 68	63	5

9. Did you discuss or work on the ICP assignments with students outside of your group?

	Yes	No
n = 68	25	44

10. Did you meet with group members to study for this course unrelated to the ICP assignments?

	Yes	No
n = 61	21	40

11. Were the instructions for the presentation clear?

	Yes	No
n = 61	50	11

12. Are the instructions for the final writing assignment clear?

	Yes	No
n = 60	53	7

13. Did you meet with your group members to work on the assignments throughout the course? If so for about how long?

a. Assignment 1 (atmospheric oxidation)?

	Did not meet	< 1 hr	1-2 hrs	2-3 hrs	3-4 hrs	>4 hrs
n = 61	39	11	5	6	0	0

b. Assignment 2 (environmental fate)?

	Did not meet	< 1 hr	1-2 hrs	2-3 hrs	3-4 hrs	>4 hrs
n = 61	37	6	9	7	2	0

c. Assignment 3a (radiative forcing)?

	Did not meet	< 1 hr	1-2 hrs	2-3 hrs	3-4 hrs	>4 hrs
n = 61	27	12	12	9	1	0

d. Assignment 3b (partitioning)?

	Did not meet	< 1 hr	1-2 hrs	2-3 hrs	3-4 hrs	>4 hrs
n = 61	23	15	12	10	1	0

e. Assignment 4a (GWP)?

	Did not meet	< 1 hr	1-2 hrs	2-3 hrs	3-4 hrs	>4 hrs
n = 61	21	13	14	10	3	0

CHM310 Environmental Chemistry – Winter 2018 Student Survey
Industrial Consultants Project (ICP) Questionnaire

The purpose of this questionnaire is to obtain additional feedback from that collected by the course reviews through the Faculty of Arts and Science. Completion of this questionnaire is voluntary, anonymous and confidential.

Note that student responses are shown below each question in blue.

1. I feel the industrial consultant project appropriately assessed my understanding of the course content.

	Strongly agree	Agree	Neutral	Disagree	Strongly disagree
n = 62	11	40	8	3	2

2. I feel the industrial consultant project helped to prepare me for the class midterm.

	Strongly agree	Agree	Neutral	Disagree	Strongly disagree
n = 62	5	17	26	11	3

3. I feel the industrial consultant project assignments that involved Excel were useful in improving my computer skills.

	Strongly agree	Agree	Neutral	Disagree	Strongly disagree	I had sufficient experience with Excel prior to this class
n = 62	17	21	12	3	1	8

4. The group work component of the industrial consultant project improved my learning experience in CHM310 overall.

	Strongly agree	Agree	Neutral	Disagree	Strongly disagree
n = 62	11	33	12	4	2

5. Giving an oral presentation as part of the industrial consultant project was a useful experience in terms of practicing my oral communication skills.

	Strongly agree	Agree	Neutral	Disagree	Strongly disagree	Not Applicable
n = 62	17	34	9	0	0	2

6. Giving an oral presentation as part of the industrial consultant project was a useful experience in terms of learning the content for the CHM 310 exam.

	Strongly agree	Agree	Neutral	Disagree	Strongly disagree	Not Applicable
n = 62	10	26	16	5	2	3

7. I had given ______ oral presentations in my university career prior to delivering the group presentation in CHM310.

	no	1	2	3	4	5 or more
n = 62	10	14	11	9	2	16
